# Modifiable risk factors for asthma exacerbations during the COVID-19 pandemic: a population-based repeated cross-sectional study using the Research and Surveillance Centre primary care database

**DOI:** 10.1016/j.lanepe.2024.100938

**Published:** 2024-05-24

**Authors:** Mome Mukherjee, Cecilia Okusi, Gavin Jamie, Rachel Byford, Filipa Ferreira, Utkarsh Agarwal, David Weatherill, Monica Fletcher, Jennifer K. Quint, Mohammad Romel Bhuia, Simon de Lusignan, Sir Aziz Sheikh

**Affiliations:** a Asthma UK Centre for Applied Research, Usher Institute, The University of Edinburgh, Edinburgh, UK; b HDR UK Better Care, The University of Edinburgh, Edinburgh, UK; c HDR UK BREATHE Data Hub, The University of Edinburgh, Edinburgh, UK; d Centre for Clinical Brain Sciences, University of Edinburgh, Edinburgh, UK; e Clinical Informatics and Health Outcomes Research Group, Nuffield Department of Primary Care Health Sciences, University of Oxford, Eagle House, Walton Well Rd, Oxford, OX2 6ED, UK; f School of Public Health & National Heart and Lung Institute, Imperial College London, London, UK; g Department of Statistics, Shahjalal University of Science and Technology (SUST), Sylhet, 3114, Bangladesh; h Royal College of General Practitioners (RCGP), 30 Euston Square, London, NW1 2FB, UK; i Nuffield Department of Primary Care Health Sciences, University of Oxford, Eagle House, Walton Well Rd, Oxford, OX2 6ED, UK

**Keywords:** Asthma, Risk factors, Exacerbations, COVID-19, Prevalence, Cluster analysis

## Abstract

**Background:**

There were substantial reductions in asthma exacerbations during the COVID-19 pandemic for reasons that remain poorly understood. We investigated changes in modifiable risk factors which might help explain the reductions in asthma exacerbations.

**Methods:**

Multilevel generalised linear mixed models were fitted to examine changes in modifiable risk factors for asthma exacerbations during 2020–2022, compared to pre-pandemic year (2019), using observational, routine data from general practices in the Oxford-Royal College of General Practitioners Research and Surveillance Centre. Asthma exacerbations were defined as any of GP recorded: asthma exacerbations, prescriptions of prednisolone, accident and emergency department attendance or hospitalisation for asthma. Modifiable risk factors of interest were ownership of asthma self-management plan, asthma annual review, inhaled-corticosteroid (ICS) prescriptions, influenza vaccinations and respiratory-tract-infections (RTI).

**Findings:**

Compared with 2019 (n = 550,995), in 2020 (n = 565,956) and 2022 (n = 562,167) (p < 0.05): asthma exacerbations declined from 67.1% to 51.9% and 61.1%, the proportion of people who had: asthma exacerbations reduced from 20.4% to 15.1% and 18.5%, asthma self-management plans increased from 28.6% to 37.7% and 55.9%; ICS prescriptions increased from 69.9% to 72.0% and 71.1%; influenza vaccinations increased from 14.2% to 25.4% and 55.3%; current smoking declined from 15.0% to 14.5% and 14.7%; lower-RTI declined from 10.5% to 5.3% and 8.1%; upper-RTI reduced from 10.7% to 5.8% and 7.6%. There was cluster effect of GP practices on asthma exacerbations (p = 0.001). People with asthma were more likely (p < 0.05) to have exacerbations if they had LRTI (seven times(x)), had URTI and ILI (both twice), were current smokers (1.4x), PPV vaccinated (1.3x), seasonal flu vaccinated (1.01x), took ICS (1.3x), had asthma reviews (1.09x). People with asthma were less likely to have exacerbations if they had self-management plan (7%), and were partially (4%) than fully COVID-19 vaccinated.

**Interpretation:**

We have identified changes in modifiable risk factors for asthma exacerbation that need to be maintained in the post-pandemic era.

**Funding:**

10.13039/501100014335Asthma UK Centre for Applied Research and 10.13039/501100023699Health Data Research UK.

**Research in context**

**Evidence before this study**

Several countries have reported substantial reductions in asthma exacerbations during the COVD-19 pandemic, but the reasons underpinning these reductions remain poorly understood. We searched PubMed for observational studies, with no language restrictions, using the terms “asthma”, “attack”, “exacerbation”, “emergency”, “hospital admission”, “death”, “modifiable factors”, “risk factors”, “SARS-CoV-2”, “COVID-19”, published between January 1, 2020, and August 10, 2023. One of the 52 studies identified reported an association between reduced rates of respiratory syncytial virus infection and asthma exacerbation in children. Three other studies reported rebounds of asthma exacerbations during the latter stages of the pandemic, which were attributed to viral resurgence associated with increased social mixing and reductions in use of face masks.

**Added value of this study**

To our knowledge, this is the first study that has investigated changes in modifiable risk factors for asthma exacerbations before and during the pandemic using primary care data. Our large, population-based analysis identified beneficial changes in several modifiable risk factors for asthma exacerbations, which may be causally associated with the reductions in exacerbations seen over the study period.

**Implications of all the available evidence**

This national analysis has identified beneficial changes in modifiable risk factors during the pandemic. There is a need to find ways to maintain the beneficial changes in risk factor exposure for asthma exacerbations and outcomes seen during the pandemic.

## Introduction

A number of studies have reported substantial reductions in asthma exacerbations during the early pandemic years.^1^, ^2^, ^3^, ^4^, ^5^, ^6^, ^7^, ^8^, ^9^, ^10^, ^11^, ^12^, ^13^, ^14^, ^15^, ^16^ There were initially concerns that these reductions in presentation of asthma exacerbations to health systems may have reflected from changes in health seeking behaviour during lockdowns.^17^^,^^18^ But these concerns have been allayed by analyses showing improvements in a range of outcomes relating to asthma exacerbations, including use of oral steroids, and reductions in emergency department attendance and hospital admission for asthma,^14^^,^^19^^,^^20^ with no corresponding increases in asthma deaths.^2^

The reasons behind these unprecedented declines in asthma exacerbations remain poorly understood. The limited body of empirical evidence available suggests that the reduction in exposure to respiratory tract infections (RTIs) may have been important,^6^^,^^16^^,^^20^^,^^21^ particularly respiratory syncytial virus exposure in children.^8^

In the context of reports suggesting that asthma exacerbations are beginning to return to pre-pandemic levels,^21^^,^^22^ we sought to investigate changes in (potentially) modifiable risk factors for asthma exacerbations comparing the immediate pre-pandemic year (2019) to the first three years of the pandemic (2020–2022) with a view to identifying which if any of these risk factors might help explain the reductions in asthma exacerbations rates seen.

## Methods

### Study design

We undertook a repeated cross-sectional, observational study using routine primary care data.

### Setting

We used pseudonymised data from the primary care sentinel cohort of the Oxford-Royal College of General Practitioners (RCGP) Research and Surveillance Centre (RSC) in England, in the UK.^23^ Participating practices were recruited to be nationally representative and were involved in virological and serological surveillance.^24^ Accident and emergency (A&E) department attendance and hospital admission for asthma were obtained from GP records.

### Study period

Four years were studied—the year prior to the pandemic (i.e., January 1, to December 31, 2019) and the three subsequent pandemic calendar years (i.e., January 1, 2020 to December 31, 2022).

### Participants

Anyone who ever had a diagnosis of asthma by their GP and was treated with any asthma medication in the previous 12 months or had a hospital admission with asthma as their primary reason for admission was identified as having asthma. Records of people who had codes indicating they opted out of record sharing were not included in the analyses.^25^

### Outcomes

The outcomes of interest were counts of people who had GP-recorded:

i) Asthma exacerbations
ii) Prescriptions of prednisolone for asthma exacerbation
iii) A&E department attendance for asthma
iv) Hospital admission for asthma

and also counts of events (i, iii, iv) and prescriptions of prednisolone.

While other oral corticosteroids exist, such as dexamethasone and prednisone, these were not included as these were very rarely used for the community-based treatment of asthma in the UK.^26^^,^^27^ Since prednisolone may also have been prescribed on repeat to manage poorly controlled asthma, prescriptions recorded in RSC as repeats were excluded. To ensure we did not miss any hospital event for asthma due to sub-optimal coding of hospital events in primary care, we used additional hospital codes with ±7 days of the asthma exacerbation recorded by GPs or prednisolone prescriptions.

People with asthma exacerbations were defined as people who had either GP-recorded asthma exacerbations, prescriptions of prednisolone for an asthma exacerbation, A&E attendance or hospital admission for asthma. The number of asthma exacerbations were defined as events of GP-recorded asthma exacerbations, prescriptions of prednisolone for an asthma exacerbation, A&E attendance, or hospital admission for asthma.

### Potentially modifiable risk factors

The inclusion criterion was any person with asthma, irrespective of exacerbation or how long they were in the database, in whom there was a record of any of the variables of interest:

i) An asthma self-management plan being given
ii) An asthma review in last 12 months
iii) Prescriptions of ICS
iv) Number of ICS prescriptions
v) Being a current smoker
vi) Influenza vaccination
vii) Pneumococcal polysaccharide vaccination (PPV) in those aged ≥65 years
viii) Consultation for one or more of upper (URTI) or lower respiratory tract infection (LRTI) or influenza-like illness (ILI).

Smoking status was ascertained by looking at consultation-level for any old records of smoking in primary care records, and ‘current smoker’, ‘non-smoker’ or ‘ex-smoker’ was flagged for each of those dates. These were then ordered by the earliest to the latest smoking status for each calendar year. The next staging was a patient-level table which flagged the best-case smoking status per patient per day. The algorithm defined the smoking status end date column by checking the status start dates of historical smoking data and assigned a status end date of ‘9999-01-01’ for the most recent consultation date, and the day before the prior consultation date. If a patient's latest smoking status was a non-smoker but their worst status so far was either an ex-smoker or current smoker their adjusted smoking status became an ex-smoker. Only one row per patient with the latest smoking status start date at the end of the calendar year was included, with the corresponding status end date, smoking status, worst status so far, and adjusted smoking status.

In the UK, the pneumococcal vaccination programme was introduced in 2003 with PPV, for people 80 years and above. Since 2005, this was extended so that people 65 years and above also became eligible for the vaccine.^28^ In contrast, pneumococcal conjugate vaccine (PCV13) was used in the childhood immunisation programme for children two years and under and not routinely administered after two years of age.^28^ PCVs are centrally administered and the data not routinely collected in primary care. We used UK SNOMED code 4837, which captured high level (parent) codes for pneumococcal vaccination, including both PPV and PCV vaccines. For this analysis, we restricted pneumococcal vaccination to 65 years and above, thus essentially only capturing PPV administration.

Since the study period included the pandemic years, we also examined if people with asthma were vaccinated against COVID-19, and whether fully or partially.

### Data analyses

Only coded data were extracted, pseudonymising as close to sources as possible.^29^

Our primary analyses were on number of people and count of events or prescriptions. The definition we used for asthma prevalence was proportion of the registered people who consulted GP and were treated or were hospitalised for asthma. This definition included people with asthma who sought healthcare in either primary or secondary care, as recorded in RSC. For estimates other than prevalence, people with asthma was the denominator for analyses for respective years. Systematized Nomenclature of Medicine–Clinical Terms (SNOMED-CT) were used to extract the data (Supplement).^30^ Since asthma review, asthma self-management plan, influenza vaccination, PPV vaccination were reviewed and administered once a year, aggregated annual data were examined. A single ICS prescription may generally have been for two inhalers. Thus, people who had an ICS prescription had at least one ICS item.

Relative changes were measured in percentages for the years 2020-22 compared with 2019, by finding the difference in percentages and comparing to the respective percentage in 2019.

In our data, patients were clustered by GP practices. Thus, individual-level data of patients within each cluster (GP practice) were correlated. To account for this nested structure of data, we fitted multilevel generalised linear mixed model (GLMM) with GP practices as random effect. We included all the variables mentioned above, along with age, sex assigned at birth, urban/rural, socio-economic status measured by quintiles of Index of Multiple Deprivation (IMD) and ethnicity (White, Asian, Black, mixed, other, unknown) as fixed effects. GP practices were treated as random effect. Asthma exacerbation as a binary variable (yes/no) was the outcome variable of interest. We conducted a sensitivity analysis with the general practices that were available over the whole of the study period to account for the changes in GP practices participation in RSC during the study period. In the sensitivity analysis, we fitted the same multilevel GLMM with only the common GP practices which were there across all four years, to examine whether the findings of our study are sensitive to the changes in GP practices participation in RSC during the study period.

Likelihood ratio test (Chi-square test) was performed (in the form of analysis of variance (ANOVA) test), to assess whether the mixed models were an improvement in terms of goodness of fit. For sensitivity analysis, the ‘receiver operating characteristic’ (ROC) and ‘area under the curve’ (AUC) were examined of the actual exacerbations to the predicted exacerbations from the respective two mixed models. Effect sizes of the included variables in the models were reported as odds ratios (OR) and 95% confidence intervals (CI).

Since COVID-19 vaccinations started in the UK in December 2020, and these COVID-19 vaccinations data were mainly available in 2021 and 2022, we examined the association between COVID-19 vaccinations and asthma exacerbations in 2021 and 2022, respectively.

SQL was used to process data using Microsoft SQL Server Management Studio, version 15.0 and analyses were done in R using R Studio Server, version 2022.02.3.

### Ethics and permissions

As anonymised individual level data were interrogated, the Health Research Authority (HRA) decision tool was used,^31^ to confirm that NHS Research Ethics Committee (REC) was not required. Edinburgh Medical School Research Ethics Committee (EMREC) approval was obtained (ref: 21-EMREC-032).

### Reporting

Strengthening the Reporting of Observational studies in Epidemiology (STROBE) and REporting of studies Conducted using Observational Routinely-collected Data (RECORD) checklists were used to guide transparent reporting (Supplement).^32^^,^^33^

### Patient and public involvement (PPI)

PPI members were consulted twice, once before the study and with interim results before writing up. Based on the first PPI consultation, we also included people who had asthma as their primary reason for hospital admission as it was noted that some people with asthma may not see their GP and seek hospital care directly. PPI members provided additional insights on interpretation of findings, which have been incorporated into the discussion. One of the PPI members is a co-author.

### Role of the funding source

The funder had no role in the study design, any aspect of undertaking the study, interpretation of findings or the decision to publish.

## Results

In the years 2019–2022, the number of people who were registered in RSC were 8,355,263, 8,566,545, 7,978,750 and 7,634,642 respectively. Asthma prevalence in the respective years were 6.8% (95% CI 6.8–6.9), 6.9% (6.8–6.9), 7.3% (7.3–7.3) and 7.6% (7.6–7.7) (Table 1). Since these were the people with asthma who sought health care, we found an increase of 11.8% in 2022 than in 2019. Across all study years, in under-five-year-olds and five-14-year-olds male preponderance was found, which reversed to female preponderance in 15–44, 45–64 and 65-and-above year olds (Table 2).

**Table 1:** Numbers of people registered and with asthma, and prevalence of asthma, in Research and Surveillance Centre (RSC) primary care database.

	People registered	People with asthma	Asthma prevalence (95% CI)
2019	8,355,263	571,884	6.8 (6.8–6.9)
2020	8,566,545	587,302	6.8 (6.8–6.9)
2021	7,978,750	583,016	7.3 (7.3–7.3)
2022	7,634,642	583,519	7.6 (7.6–7.6)

**Table 2:** Percentages of females and males by age-groups in the calendar years in Research and Surveillance Centre (RSC) primary care database.

	<5 years	5–14 years	15–44 years	45–64 years	≥65 years	Total
2019						
Females	0.3	3.9	18.6	18.1	14.8	318,009
Males	0.4	5.8	15.3	13.4	9.5	253,875
2020						
Females	0.2	3.7	18.9	18.5	14.6	327,923
Males	0.3	5.5	15.4	13.6	9.4	259,379
2021						
Females	0.2	3.4	19	18.7	14.8	326,814
Males	0.3	5.2	15.3	13.7	9.5	256,202
2022						
Females	0.2	3.2	19	18.7	15.1	328,021
Males	0.3	4.9	15.2	13.6	9.7	255,498

**Fig 1:**
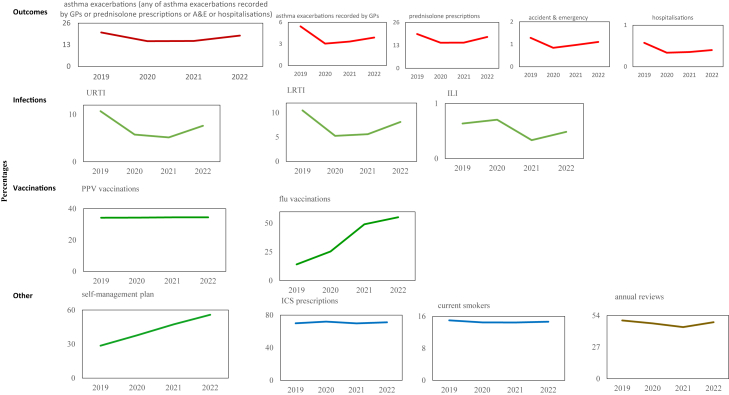
**Percentages of people with asthma modifiable factors, respiratory tract infections and outcomes in Research and Surveillance Centre (RSC) primary care database**.

The proportion of people who had asthma exacerbations in 2019 (20.4%, n = 116,481) reduced by 25.7% in 2020 (15.1%, n = 88,908), by 24.9% in 2021 (15.5% n = 89,161), and by 9.1% in 2022 (18.5%, n = 108,047) (Fig. 1, Table 3). Whereas compared with 2019, the proportion of asthma exacerbations declined by 22.7% in 2020 from 67.1% to 51.9%, by 18.7% in 2021 (54.6%), and by 9.0% in 2022 (61.1%) (Fig. 1 and Table 4).

**Table 3:** Number and percentages of people with asthma modifiable factors, respiratory tract infections and outcomes in Research and Surveillance Centre (RSC) primary care database.

People with	2019		2020		2021		2022	
	n	%	n	%	n	%	n	%
Active asthma–treated in last 12 months (denominator)	571,884	100	587,302	100	583,016	100	583,519	100
Asthma annual reviews	284,700	49.8	277,936	47.3	257,436	44.2	281,937	48.3
Asthma self-management plan	163,572	28.6	221,449	37.7	276,061	47.4	326,024	55.9
Influenza vaccinations	80,930	14.2	149,163	25.4	286,510	49.1	322,440	55.3
PPV vaccinations	195,944	34.3	201,530	34.3	201,099	34.5	201,209	34.5
Current smokers	85,854	15.0	85,018	14.5	84,313	14.5	85,523	14.7
ICS prescriptions	400,016	69.9	422,753	72.0	407,020	69.8	415,044	71.1
URTI	61,130	10.7	33,791	5.8	30,023	5.1	44,423	7.6
LRTI	59,903	10.5	30,868	5.3	32,594	5.6	47,365	8.1
ILI	3646	0.6	4143	0.7	1952	0.3	2833	0.5
Asthma exacerbations (any of asthma exacerbations recorded by GPs or prednisolone prescriptions or A&E or hospitalisations)	116,481	20.4	88,908	15.1	89,161	15.3	108,047	18.5
Asthma exacerbations recorded by GPs	31,107	5.4	17,908	3.0	19,413	3.3	22,671	3.9
Prednisolone prescriptions	111,286	19.5	85,202	14.5	84,876	14.6	103,703	17.8
A&E	7370	1.3	4961	0.8	5661	1.0	6456	1.1
Hospitalisations	3287	0.6	1974	0.3	2057	0.4	2333	0.4

**Table 4:** Number and percentages of events of asthma exacerbations in Research and Surveillance Centre (RSC) primary care database.

	2019		2020		2021		2022	
	n	%	n	%	n	%	n	%
Asthma exacerbations (any of asthma exacerbations recorded by GPs or prednisolone prescriptions or A&E or hospitalisations)	383,927	67.1	304,605	51.9	318,203	54.6	356,400	61.1
Asthma exacerbations recorded by GPs	42,210	7.4	24,396	4.2	26,454	4.5	30,185	5.2
Prednisolone prescriptions	211,170	36.9	166,948	28.4	161,523	27.7	193,185	33.1
A&E	137,051	24.0	111,104	18.9	127,654	21.9	133,719	22.9
Hospitalisations	37 417	6.5	29,195	5.0	31,036	5.3	30,835	5.3

Fig. 1 (and Table 3) show that compared with 2019, there were increases in the proportion of the population who had:

• Influenza vaccinations by 79.5% in 2020 (25.4% vs 14.2%), by 247.3% in 2021 (49.1%) and by 290.5% in 2022 (55.3%);
• Asthma self-management plans by 31.8% in 2020 (37.7% vs 28.6%), by 65.5% in 2021 (47.4%) and by 95.3% in 2022 (55.9%);
• ICS prescriptions by 2.9% in 2020 (72.0% vs 69.9%) and by 1.7% in 2022 (71.1%). The average number of ICS prescriptions, which might have had more than one ICS item, were 3.5, 3.7, 3.6 and 3.7, respectively;
• PPV vaccinations by 0.2% in 2020 (34.3%) compared to 2019 (34.3%), by 0.7% in 2021 and by 0.6% in 2022.

Compared with 2019 (Fig. 1 and Table 3), the proportion of people who:

• Had URTI reduced by 46.2% in 2020 (5.8% vs 10.7%), 51.8% in 2021 and 28.8% in 2022;
• Had LRTI reduced by 49.8% (5.3% vs 10.5), 46.6% in 2021 and 22.5% in 2022;
• Had ILI increased by 10.6% (0.7% vs 0.6%) in 2020 then decreased by 47.5% in 2021 and 23.8% in 2022;
• Were current smokers reduced by 3.9% (14.5% vs 15.0% (), by 4.1% in 2021 and by 2.9% in 2022.

In 2020, 0.02% people with asthma were fully and 2.3% were partially vaccinated against COVID-19 (Table 5). There were 78.4% people with asthma who were fully vaccinated against COVID-19 in 2022, compared to 74.8% in 2021. In 2022 there were 19.2% people with asthma who were not COVID-19 vaccinated.

**Table 5:** COVID-19 vaccination status in people with asthma, by years, in Research and Surveillance Centre (RSC) primary care database.

	Treated for active asthma in last 12 months		Fully COVID-19 vaccinated		Partially COVID-19 vaccinated		Not COVID-19 vaccinated	
	n	%	n	%	n	%	n	%
2019	571,884	100	0	0	0	0	571,884	100
2020	587,302	100	121	0	13,275	2.3	573,906	97.7
2021	583,016	100	435,950	74.8	26,041	4.5	121,025	20.8
2022	583,519	100	457,241	78.4	14,375	2.5	111,903	19.2

In the full mixed model analysis (Table 6) it was found the cluster effect of GP practices were significant at better than 0.001 level, and that people with asthma who:

i) had LRTI (OR: 6.59; 95% CI: 6.52–6.66) were seven times more likely to have exacerbations than people who did not have LRTI,
ii) had URTI (OR: 1.87; 95% CI: 1.85–1.90) and ILI (OR: 1.71; 95% CI: 1.64–1.78) were 2 times more likely to have exacerbations than people who didn't,
iii) were current smokers (OR: 1.39; 95% CI: 1.38–1.41) were 1.4 times more likely to have exacerbations than non-active smokers,
iv) were PPV vaccinated (OR: 1.29; 95% CI: 1.28–1.30) were 1.3 times more likely to have exacerbations than who were not vaccinated,
v) lived in urban areas (OR: 1.04; 95% CI: 1.03–1.06) were 1.04 times more likely to have exacerbations than who lived rurally,
vi) were seasonal flu vaccinated (OR: 1.011; 95% CI: 1.002–1.021) were 1.01 times more likely to have exacerbations than who were not vaccinated,
vii) took ICS (OR: 1.27; 95% CI: 1.25–1.28) were 1.3 times more likely to have exacerbations than who did not,
viii) had asthma reviews (OR: 1.09; 95% CI: 1.08–1.10) were 1.09 times likely to have exacerbations than who did not,
ix) had asthma self-management plan (OR: 0.93; 95% CI: 0.92–0.94) were 7% less likely to have exacerbations than who did not,
x) were partially (OR: 0.96; 95% CI: 0.94–0.99) COVID-19 vaccinated were 4% less likely to have exacerbations than who were fully COVID-19 vaccinated.

**Table 6:** Odds Ratio of asthma exacerbations with lower and upper limit of 95% CI of the multilevel model with GP practices as random effect in Research and Surveillance Centre (RSC) primary care database.

	Odds ratio	95% CI lower limit	95% CI upper limit
Age	1.003	1.002	1.003
Sex Male	0.72	0.71	0.73
Year 2020	0.79	0.78	0.79
Year 2021	0.80	0.78	0.81
Year 2022	0.94	0.92	0.95
Ethnicity Black	0.86	0.83	0.89
Ethnicity Mixed	0.92	0.89	0.96
Ethnicity Unknown	0.88	0.86	0.90
Ethnicity White	0.95	0.94	0.97
IMD 2	0.94	0.93	0.95
IMD 3	0.90	0.89	0.91
IMD 4	0.86	0.84	0.87
IMD 5 least deprived	0.81	0.79	0.82
Urban-Rural Urban	1.04	1.03	1.06
Asthma Review Yes	1.09	1.08	1.10
Asthma Self-Management Plan Yes	0.93	0.92	0.94
URLI Yes	1.87	1.85	1.90
LRTI Yes	6.59	6.52	6.66
ILI Yes	1.71	1.64	1.78
ICS Yes	1.27	1.25	1.28
Number of ICS prescription	1.07	1.07	1.07
PPV Yes	1.29	1.28	1.30
Seasonal flu vaccination Yes	1.011	1.002	1.021
Current Smoker Yes	1.39	1.38	1.41
COVID-19 vaccination Partially	0.96	0.94	0.99

GP practices (random effect): p < 0.0001.

References: Sex female, Year 2019, Ethnicity Asian, IMD 1 most deprived, Rural, Asthma Review No, Asthma Self-Management Plan No, URLI No, LRTI No, ILI No, ICS No, PPV No, Seasonal flu vaccination No, Current Smoker No, COVID-19 vaccination Fully.

The odds of asthma exacerbation were lower during the years 2020–2022 than in the year 2019. White, Black, Mixed or people with unknown ethnicity had reduced odds of asthma exacerbations as compared to Asian people. Compared to people with asthma in most deprived areas, people in areas of lesser deprivation had reduced exacerbations. The odds of asthma exacerbation increased 1.003 times if age increased by one year. Males with asthma were 28% less likely to have exacerbations than females. All the fixed effects were significant at better than 0.001 probability level, except seasonal flu vaccinated (p = 0.02).

### Sensitivity analysis

Over the years (2019–2022) there were 778, 773, 775 and 777 GP practices, respectively. In the sensitivity analysis focused on 768 GP practices which were common in all four years, it was found that the cluster effect of GP practices and the fixed effects were significant at better than 0.001 level, except seasonal flu vaccinated (p = 0.01). The AUC from this model was 0.7341, while of the model that included all the GP practices was 0.7342, which are almost same.

When the models were restricted to 2021 and 2022, cluster effect of GP practices and the fixed effects were significant at better than 0.001 level, except mixed ethnicity (0.01), white ethnicity (p = 0.02), other ethnicity (p = 0.04), and seasonal flu vaccinated (p = 0.06).

## Discussion

In this national observational study, we observed reductions in both the proportion of people who had asthma exacerbations and the proportion of asthma exacerbations, and corresponding changes in a number of potentially modifiable risk factors for asthma exacerbations. We found people with asthma were less likely to have exacerbations if they had asthma self-management plan (7% less likely), and were partially (4% less likely) than fully COVID-19 vaccinated. People with asthma who were PPV and seasonal flu vaccinated, who took ICS, had asthma reviews, were Asian, lived in urban areas and in more deprived areas, were more likely to have asthma exacerbations. Whilst causality cannot be inferred from observational study designs, our study demonstrated association of these observed changes in risk factors with the reductions in asthma exacerbations seen.

That people with asthma who were either PPV or seasonal flu vaccinated, or had asthma reviews were likely to have more exacerbations could be due to each of these being targeted to at-risk people in the UK, including people with asthma and the elderly, who are prone to exacerbations. Also, it could be due to individual's perception of risk, particularly if they are having exacerbations, to get themselves vaccinated and attend asthma reviews. Equally COVID-19 vaccination was targeted to similar group of people and we found that compared to fully vaccinated COVID-19 people, those who were not or partially vaccinated had lesser exacerbations. People with asthma who had ICS prescriptions during our study period were more likely to have asthma exacerbations perhaps because they had persistence of symptoms or had severity. RSC database does not have data on prescriptions dispensed. It seems people with asthma who were proactive and had asthma self-management plan could ward off exacerbations. Thus, people's behaviour might have played a role in their own asthma outcome. Furthermore, cluster effect of GP practices was found on asthma exacerbations. Thus, besides people's behaviour, it could be how GP practices managed care of people with asthma which affected their asthma outcome.

The directions of the estimates of model parameters and their inferences were almost same in our final fitted model and the model used in sensitivity analysis, along with the AUC values. That implies that the findings of our study are not sensitive to changes in GP practices participation in RSC during the study period.

At the onset of the pandemic, respiratory services were particularly impacted whereby there were cancellations of routine care by GPs and hospitals, and a reduction in face-to-face consultations.^34^ But a large study on over nine million consultations in primary care in England found there were 15% more consultations in 2021-22 than in 2018-19, whereby remote consultations were 155.3% higher and face-to-face consultations were 17.1% lower than in 2018-19.^35^ Although we did not calculate the number of GP consultations for asthma, the proportion of people who were treated for their asthma in primary and secondary care, as recorded in primary care, increased by 17.1% in 2022 when compared to 2019. We found a decline in asthma annual reviews by 4.9% in 2020 and 1.9% in 2022. This could be a result of NHS England changing the GP contract, which allowed GPs to curtail annual reviews during the pandemic so as to increase capacity for frontline services to respond to COVID-19.^36^ Despite the reductions in asthma annual reviews, we found a reduction in asthma exacerbations. Clinician-reported-diagnosed-treated asthma prevalence in Quality and Outcomes Framework (QoF), a GP incentivisation system, in England in 2019-20 for all ages and in 2020-21 for ages above-six-years was both 6.5%,^37^ which is close to our prevalence estimates. The asthma profile described here with a wide base of 0.6 million people, of whom a relatively small proportion (0.4%) experienced hospitalisation for their asthma is similar to a Scottish study.^38^

Key strengths of this work include the large primary care-based database interrogated with coded data on a number of potentially important risk factors.

Limitations include using a routine primary care database means there could be potential over- or under-diagnosis of asthma, and also bias in the collection of some of the exposure variables of interest.^39^ Analyses were limited to coded data that can also be subject to recording bias. That said, given that these electronic health record derived data represent the main clinical record, we anticipate that these would have been unlikely to have a major impact on our overall findings. Our estimates of exacerbations are likely to be underestimates for people who managed their exacerbations at home and did not report to GP. On the other hand, we did not put any restriction on number of days around an exacerbation and counted every event as an exacerbation if they met our criteria above. We might have under-estimated A&E and hospitalisation records since these may not be fully captured in primary care.

Community surveillance indicators in the UK reported the impact of social and physical distancing measures on COVID-19 activity within one week.^40^ Face masks and other non-pharmaceutical interventions (NPI) were in widespread use during the early stages of the pandemic and these may have contributed to reductions in the incidence of respiratory tract infections, which are important triggers for asthma exacerbations. Definitely establishing the effectiveness of NPIs in preventing asthma exacerbations would require a formal randomised controlled trial, but there is considerable uncertainty as to whether this could be successfully delivered in the UK context.

Our analysis now needs to be built on with studies investigating patient behaviours and how these may have changed during these early pandemic years through surveys and qualitative evaluations. There is also a need to consider which aspects of behaviour change may persist beyond the early pandemic years, which could be achieved both through extending the present analysis and in-depth work with patients to understand their behavioural patterns.

In summary, this national observational study found reductions in people who had asthma exacerbations. Over the same period, we noted increases in asthma self-management ownership, ICS prescriptions and influenza vaccination, and reductions in smoking and RTIs. There is a need to find ways of maintaining and building on these improvements in behaviours, care processes and exposures to achieve sustained improvements in asthma outcomes in the post-pandemic era.

## Contributors

AS, MM, SdeL conceived the study and contributed to the study design. SdeL was the director, guarantor for these data, assisted with clinical knowledge, system design and problem solving, and commented on the drafts. MM led the analysis plan, oversaw the analysis, analysed data, led the writing of the paper and edited the manuscripts. CO produced the base data tables and summary data. RB designed and developed much of the database structure. GJ was responsible for curation of clinical variables. UA advised on COVID-19 vaccinations. DW provided patient and public perspective. FF was responsible for project and contract management in RSC. MRB advised and oversaw the statistical analysis. MM, CO, GJ and SdeL have verified the data. AS, MRB, DW, GJ, JQ, MF, MM, CO, SdeL commented on the draft and revised the manuscript for important intellectual content. All authors had final responsibility for the decision to submit for publication.

## Data sharing statement

Access to confidential Oxford-RCGP RSC data can be requested by submitting a written protocol and data request form to Oxford-RCGP RSC.

## Declaration of interests

AS was a member of the Scottish Government Chief Medical Officer's COVID-19 Advisory Group and Astra-Zeneca's COVID-19 Thrombotic Thrombocytopenic Advisory Group and is a member of the Scottish Government's Standing Committee on Pandemic Preparedness; all roles are unremunerated. SdeL is the director of Royal College of GPs Research and Surveillance Centre (RSC) the primary care sentinel network, UK Health Security Agency funded. SdeL has had research funded by AstraZeneca, Moderna, GSK, Sanofi and Seqirus, has spoken at Seqirus and Roche funded events, was a member of advisory boards for AstraZeneca, Sanofi and Seqirus and had AstraZeneca funded attendance at a European conference, all fees for these events were paid to the University of Oxford.
