# Supplementary material for: Modifiable risk factors for asthma exacerbations during the COVID-19 pandemic: a population-based repeated cross-sectional study using the Research and Surveillance Centre primary care database
Source: Lancet Reg Health Eur. 2024 May 24;42:100938. doi: 10.1016/j.lanepe.2024.100938 (PMC11153226; doi:10.1016/j.lanepe.2024.100938)
Supplement: Supplement Tables [file mmc1.pdf]

**Supplement**

## Codes for asthma and modifiable factors, and RECORD and STROBE statements

## Table of contents


| | Pages |
|---|---|
| **Table S1: SNOMED CT codes for asthma** | **2-76** |
| **Table S2: SNOMED CT Expression Constraint Language (ECL) codes for asthma** | **77-80** |
| **Table S3: DM+D codes for prescriptions for asthma** | **80-128** |
| **Table S4: The RECORD statement – checklist of items, extended from the STROBE statement, for observational studies using routinely collected health data** | **129-132** |

**Table S1: SNOMED CT codes for asthma**

| Condition Name | Condition ID | Concept ID | Primary Term |
|---|---|---|---|
| AsthmaExacerbation | 4204 | 30352005 | Allergic-infective asthma |
| AsthmaExacerbation | 4204 | 57546000 | Asthma with status asthmaticus |
| AsthmaExacerbation | 4204 | 59327009 | Intrinsic asthma with status asthmaticus |
| AsthmaExacerbation | 4204 | 91340006 | Extrinsic asthma with status asthmaticus |
| AsthmaExacerbation | 4204 | 233681001 | Extrinsic asthma with asthma attack |
| AsthmaExacerbation | 4204 | 233685005 | Intrinsic asthma with asthma attack |
| AsthmaExacerbation | 4204 | 233686006 | Aspirin-sensitive asthma with nasal polyps |
| AsthmaExacerbation | 4204 | 266363006 | Status asthmaticus NOS |
| AsthmaExacerbation | 4204 | 266364000 | Asthma attack |
| AsthmaExacerbation | 4204 | 274105009 | Asthma attack NOS |
| AsthmaExacerbation | 4204 | 281239006 | Acute asthma |
| AsthmaExacerbation | 4204 | 304527002 | Acute asthma |
| AsthmaExacerbation | 4204 | 407674008 | Aspirin-induced asthma |
| AsthmaExacerbation | 4204 | 425969006 | Exacerbation of intermittent asthma |
| AsthmaExacerbation | 4204 | 427354000 | Exacerbation of persistent asthma |
| AsthmaExacerbation | 4204 | 442025000 | Acute exacerbation of chronic asthmatic bronchitis |
| AsthmaExacerbation | 4204 | 707445000 | Exacerbation of mild persistent asthma |
| AsthmaExacerbation | 4204 | 707446004 | Exacerbation of moderate persistent asthma |
| AsthmaExacerbation | 4204 | 707447008 | Exacerbation of severe persistent asthma |
| AsthmaExacerbation | 4204 | 707979007 | Acute severe exacerbation of severe persistent asthma |
| AsthmaExacerbation | 4204 | 707980005 | Acute severe exacerbation of moderate persistent asthma |
| AsthmaExacerbation | 4204 | 707981009 | Acute severe exacerbation of mild persistent asthma |
| AsthmaExacerbation | 4204 | 708038006 | Acute exacerbation of asthma |
| AsthmaExacerbation | 4204 | 708090002 | Acute severe exacerbation of asthma |
| AsthmaExacerbation | 4204 | 708093000 | Acute exacerbation of allergic asthma |
| AsthmaExacerbation | 4204 | 708094006 | Acute exacerbation of intrinsic asthma |
| AsthmaExacerbation | 4204 | 708095007 | Acute severe exacerbation of immunoglobin E-mediated allergic asthma |
| AsthmaExacerbation | 4204 | 708096008 | Acute severe exacerbation of intrinsic asthma |
| AsthmaExacerbation | 4204 | 733858005 | Acute severe refractory exacerbation of asthma |
| AsthmaExacerbation | 4204 | 734904007 | Life threatening acute exacerbation of asthma |
| AsthmaExacerbation | 4204 | 734905008 | Moderate acute exacerbation of asthma |
| AsthmaExacerbation | 4204 | 735587000 | Acute severe exacerbation of asthma co-occurrent and due to allergic asthma |
| AsthmaExacerbation | 4204 | 762521001 | Exacerbation of allergic asthma |
| AsthmaExacerbation | 4204 | 782513000 | Acute severe exacerbation of allergic asthma |
| AsthmaExacerbation | 4204 | 782520007 | Exacerbation of allergic asthma due to infection |
| AsthmaExacerbation | 4204 | 786836003 | Near fatal asthma |

| Condition Name | Condition ID | Concept ID | Primary Term |
|---|---|---|---|
| AsthmaExacerbation | 4204 | 829976001 | Thunderstorm asthma |
| AsthmaExacerbation | 4204 | 1751000119100 | Acute exacerbation of chronic obstructive airways disease with asthma |
| AsthmaExacerbation | 4204 | 99031000119107 | Acute exacerbation of asthma co-occurrent with allergic rhinitis |
| AsthmaExacerbation | 4204 | 135171000119106 | Acute exacerbation of moderate persistent asthma |
| AsthmaExacerbation | 4204 | 135181000119109 | Acute exacerbation of mild persistent asthma |
| AsthmaExacerbation | 4204 | 653751000000109 | Asthma attack NOS |
| AsthmaExacerbation | 4204 | 689421000000104 | Status asthmaticus NOS |
| AsthmaExacerbation | 4204 | 1064771000000103 | Acute severe exacerbation of asthma |
| AsthmaExacerbation | 4204 | 1064811000000103 | Moderate acute exacerbation of asthma |
| AsthmaExacerbation | 4204 | 1064821000000109 | Life threatening acute exacerbation of asthma |
| AsthmaExacerbation | 4204 | 1086701000000102 | Life threatening acute exacerbation of allergic asthma |
| AsthmaExacerbation | 4204 | 1086711000000100 | Life threatening acute exacerbation of intrinsic asthma |
| AsthmaExacerbation | 4204 | 10674711000119105 | Acute severe exacerbation of asthma co-occurrent with allergic rhinitis |
| AsthmaExacerbation | 4204 | 10675471000119109 | Acute severe exacerbation of severe persistent allergic asthma |
| AsthmaExacerbation | 4204 | 10675551000119104 | Acute severe exacerbation of severe persistent asthma co-occurrent with allergic rhinitis |
| AsthmaExacerbation | 4204 | 10675911000119109 | Acute severe exacerbation of mild persistent allergic asthma |
| AsthmaExacerbation | 4204 | 10675991000119100 | Acute severe exacerbation of mild persistent allergic asthma co-occurrent with allergic rhinitis |
| AsthmaExacerbation | 4204 | 10676431000119103 | Acute severe exacerbation of moderate persistent allergic asthma |
| AsthmaExacerbation | 4204 | 10676511000119109 | Acute severe exacerbation of moderate persistent asthma co-occurrent with allergic rhinitis |
| AsthmaExacerbation | 4204 | 10692681000119108 | Aspirin exacerbated respiratory disease |
| AsthmaExacerbation | 4204 | 10692721000119102 | Chronic obstructive asthma co-occurrent with acute exacerbation of asthma |
| AsthmaManagementNoPlan | 4210 | 169591000000104 | Does not have asthma management plan |
| AsthmaManagementNoPlan | 4210 | 174171000000100 | Does not have asthma management plan |
| AsthmaManagementNoPlan | 4210 | 176711000000100 | Does not have asthma management plan |
| AsthmaManagementNoPlan | 4210 | 892301000000100 | Asthma management plan declined |
| AsthmaManagementNoPlan | 4210 | 916481000000109 | Asthma management plan declined |

| Condition Name | Condition ID | Concept ID | Primary Term |
|---|---|---|---|
| AsthmaManagementPlan | 4209 | 390872009 | Change in asthma management plan |
| AsthmaManagementPlan | 4209 | 390877003 | Step up change in asthma management plan |
| AsthmaManagementPlan | 4209 | 390878008 | Step down change in asthma management plan |
| AsthmaManagementPlan | 4209 | 412775002 | Asthma clinical management plan |
| AsthmaManagementPlan | 4209 | 736056000 | Asthma clinical management plan |
| AsthmaManagementPlan | 4209 | 116961000000106 | Asthma clinical management plan |
| AsthmaManagementPlan | 4209 | 121701000000107 | Asthma clinical management plan |
| AsthmaManagementPlan | 4209 | 811921000000103 | Asthma self-management plan agreed |
| AsthmaManagementPlan | 4209 | 811931000000101 | Asthma self-management plan agreed |
| AsthmaReview | 4208 | 393030008 | Asthma annual review |
| AsthmaReview | 4208 | 393082005 | Asthma medication review |
| AsthmaReview | 4208 | 393986005 | Asthma annual review |
| AsthmaReview | 4208 | 394033000 | Asthma medication review |
| AsthmaReview | 4208 | 394700004 | Asthma annual review |
| AsthmaReview | 4208 | 394720003 | Asthma medication review |
| AsthmaReview | 4208 | 754061000000100 | Asthma review using Royal College of Physicians three questions |
| AsthmaReview | 4208 | 754071000000107 | Asthma review (three Royal College of Pysicisan questions) |
| HospitalAdmission | 7163 | 417005 | Hospital re-admission |
| HospitalAdmission | 7163 | 1505002 | Hospital admission for isolation |
| HospitalAdmission | 7163 | 2252009 | Hospital admission, urgent, 48 hours |
| HospitalAdmission | 7163 | 2876009 | Hospital admission, type unclassified, explain by report |
| HospitalAdmission | 7163 | 4563007 | Hospital admission, transfer from other hospital or health care facility |
| HospitalAdmission | 7163 | 5161006 | Speciality clinic admission |
| HospitalAdmission | 7163 | 8715000 | Hospital admission, elective |
| HospitalAdmission | 7163 | 10378005 | Hospital admission, emergency, from emergency room, accidental injury |
| HospitalAdmission | 7163 | 11545006 | Emergency room admission, dead on arrival (DOA) |
| HospitalAdmission | 7163 | 15584006 | Hospital admission, elective, with partial pre-admission work-up |
| HospitalAdmission | 7163 | 18083007 | Hospital admission, emergency, indirect |
| HospitalAdmission | 7163 | 19951005 | Hospital admission, emergency, from emergency room, medical nature |

| Condition Name | Condition ID | Concept ID | Primary Term |
|---|---|---|---|
| HospitalAdmission | 7163 | 23473000 | Hospital admission, for research investigation |
| HospitalAdmission | 7163 | 25986004 | Hospital admission, under police custody |
| HospitalAdmission | 7163 | 32485007 | Hospital admission |
| HospitalAdmission | 7163 | 36723004 | Hospital admission, pre-nursing home placement |
| HospitalAdmission | 7163 | 40274000 | General outpatient clinic admission |
| HospitalAdmission | 7163 | 45702004 | Hospital admission, precertified by medical audit action |
| HospitalAdmission | 7163 | 47348005 | Hospital admission, mother, for observation, delivered outside of hospital |
| HospitalAdmission | 7163 | 48183000 | Hospital admission, special |
| HospitalAdmission | 7163 | 50331008 | Emergency room admission, followed by release |
| HospitalAdmission | 7163 | 50699000 | Hospital admission, short-term |
| HospitalAdmission | 7163 | 50849002 | Emergency room admission |
| HospitalAdmission | 7163 | 51032003 | Hospital admission, donor for transplant organ |
| HospitalAdmission | 7163 | 51501005 | Hospital admission, parent, for in-hospital child care |
| HospitalAdmission | 7163 | 52748007 | Hospital admission, involuntary |
| HospitalAdmission | 7163 | 55402005 | Hospital admission, for laboratory work-up, radiography, etc. |
| HospitalAdmission | 7163 | 60059000 | Hospital admission, infant, for observation, delivered outside of hospital |
| HospitalAdmission | 7163 | 63551005 | Hospital admission, from remote area, by means of special transportation |
| HospitalAdmission | 7163 | 65043002 | Hospital admission, short-term, day care |
| HospitalAdmission | 7163 | 70755000 | Hospital admission, by legal authority (commitment) |
| HospitalAdmission | 7163 | 71290004 | Hospital admission, limited to designated procedures |
| HospitalAdmission | 7163 | 73607007 | Hospital admission, emergency, from emergency room |
| HospitalAdmission | 7163 | 74857009 | Hospital admission, short-term, 24 hours |
| HospitalAdmission | 7163 | 75004002 | Emergency room admission, died in emergency room |
| HospitalAdmission | 7163 | 76464004 | Hospital admission, for observation |
| HospitalAdmission | 7163 | 78680009 | Hospital admission, emergency, direct |
| HospitalAdmission | 7163 | 81672003 | Hospital admission, elective, without pre-admission work-up |
| HospitalAdmission | 7163 | 82942009 | Hospital admission, blood donor |
| HospitalAdmission | 7163 | 112689000 | Hospital admission, elective, with complete pre-admission work-up |
| HospitalAdmission | 7163 | 112690009 | Hospital admission, boarder, for social reasons |
| HospitalAdmission | 7163 | 183430001 | Holiday relief admission |
| HospitalAdmission | 7163 | 183452005 | Emergency hospital admission |

| Condition Name | Condition ID | Concept ID | Primary Term |
|---|---|---|---|
| HospitalAdmission | 7163 | 183453000 | Admit medical emergency unspecified |
| HospitalAdmission | 7163 | 183454006 | Admit surgical emergency unspecified |
| HospitalAdmission | 7163 | 183455007 | Psychiatric emergency hospital admission |
| HospitalAdmission | 7163 | 183456008 | Emergency psychiatric admission MHA |
| HospitalAdmission | 7163 | 183457004 | Geriatric emergency hospital admission |
| HospitalAdmission | 7163 | 183458009 | Paediatric emergency hospital admission |
| HospitalAdmission | 7163 | 183459001 | Gynaecological emergency hospital admission |
| HospitalAdmission | 7163 | 183460006 | Obstetric emergency hospital admission |
| HospitalAdmission | 7163 | 183461005 | Orthopaedic emergency hospital admission |
| HospitalAdmission | 7163 | 183462003 | ENT emergency hospital admission |
| HospitalAdmission | 7163 | 183463008 | Trauma emergency hospital admission |
| HospitalAdmission | 7163 | 183464002 | Ophthalmological emergency hospital admission |
| HospitalAdmission | 7163 | 183465001 | Rheumatology emergency hospital admission |
| HospitalAdmission | 7163 | 183466000 | Dermatology emergency hospital admission |
| HospitalAdmission | 7163 | 183467009 | Neurology emergency hospital admission |
| HospitalAdmission | 7163 | 183468004 | Urology emergency hospital admission |
| HospitalAdmission | 7163 | 183469007 | Radiotherapy emergency hospital admission |
| HospitalAdmission | 7163 | 183470008 | Haematology emergency hospital admission |
| HospitalAdmission | 7163 | 183471007 | Plastic surgery emergency hospital admission |
| HospitalAdmission | 7163 | 183472000 | Diabetic emergency hospital admission |
| HospitalAdmission | 7163 | 183473005 | Oral surgical emergency hospital admission |
| HospitalAdmission | 7163 | 183474004 | Psychogeriatric emergency hospital admission |
| HospitalAdmission | 7163 | 183475003 | Renal medicine emergency hospital admission |
| HospitalAdmission | 7163 | 183476002 | Neurosurgical emergency hospital admission |
| HospitalAdmission | 7163 | 183477006 | Cardiothoracic emergency hospital admission |
| HospitalAdmission | 7163 | 183478001 | Emergency hospital admission for asthma |
| HospitalAdmission | 7163 | 183480007 | Admit hospital emergency NOS |
| HospitalAdmission | 7163 | 183481006 | Non-urgent hospital admission |
| HospitalAdmission | 7163 | 183482004 | Non-urgent hospital admission unspecified |
| HospitalAdmission | 7163 | 183497001 | Non-urgent trauma admission |
| HospitalAdmission | 7163 | 183514007 | Other hospital admission NOS |
| HospitalAdmission | 7163 | 299964000 | [V] Admission for bladder training |
| HospitalAdmission | 7163 | 302187006 | [V]Admission for instruction of self-catheterisation |
| HospitalAdmission | 7163 | 302987007 | [V]Acquired absence of organs |
| HospitalAdmission | 7163 | 304568006 | Admission for respite care |
| HospitalAdmission | 7163 | 305337004 | Admission to community hospital |
| HospitalAdmission | 7163 | 305338009 | Admission to GP hospital |
| HospitalAdmission | 7163 | 305339001 | Admission to private hospital |
| HospitalAdmission | 7163 | 305340004 | Admission to long stay hospital |
| HospitalAdmission | 7163 | 305341000 | Admission to tertiary referral hospital |
| HospitalAdmission | 7163 | 305342007 | Admission to ward |

| Condition Name | Condition ID | Concept ID | Primary Term |
|---|---|---|---|
| HospitalAdmission | 7163 | 305343002 | Admission to day ward |
| HospitalAdmission | 7163 | 305344008 | Admission to day hospital |
| HospitalAdmission | 7163 | 305345009 | Admission to psychiatric day hospital |
| HospitalAdmission | 7163 | 305346005 | Admission to psychogeriatric day hospital |
| HospitalAdmission | 7163 | 305347001 | Admission to elderly severely mentally ill day hospital |
| HospitalAdmission | 7163 | 305348006 | Admission to care of the elderly day hospital |
| HospitalAdmission | 7163 | 308162006 | [V]Admission for respite care |
| HospitalAdmission | 7163 | 308163001 | [V]Problems in relationship with parents and in-laws |
| HospitalAdmission | 7163 | 308164007 | [V]Problem was normal state |
| HospitalAdmission | 7163 | 309561003 | [V]Procreative/fertility counselling |
| HospitalAdmission | 7163 | 309788005 | [V]Admission for instruction in the use of a nebuliser |
| HospitalAdmission | 7163 | 313265008 | [V]Procedure cancelled due to non-availability of anaesthetist |
| HospitalAdmission | 7163 | 313266009 | [V]Procedure cancelled due to lack of theatre time |
| HospitalAdmission | 7163 | 313285007 | [V]Admitted for conversion to insulin |
| HospitalAdmission | 7163 | 313286008 | [V]Admitted for commencement of insulin |
| HospitalAdmission | 7163 | 313385005 | Cardiology emergency hospital admission |
| HospitalAdmission | 7163 | 315949001 | [V]Genetic counselling |
| HospitalAdmission | 7163 | 316361005 | [V]Housing, household and economic circumstances as reason for encounter |
| HospitalAdmission | 7163 | 316362003 | [V]Lack of housing |
| HospitalAdmission | 7163 | 316363008 | [V]Inadequate housing |
| HospitalAdmission | 7163 | 316364002 | [V]Inadequate material resources |
| HospitalAdmission | 7163 | 316365001 | [V]Person living alone |
| HospitalAdmission | 7163 | 316366000 | [V]No able carer in household |
| HospitalAdmission | 7163 | 316367009 | [V]Holiday relief care |
| HospitalAdmission | 7163 | 316368004 | [V]Institution resident |
| HospitalAdmission | 7163 | 316369007 | [V]Sheltered housing |
| HospitalAdmission | 7163 | 316370008 | [V]Carer unable to cope |
| HospitalAdmission | 7163 | 316372000 | [V]Other social reason for encounter |
| HospitalAdmission | 7163 | 316373005 | [V]Unspecified social reason for encounter |
| HospitalAdmission | 7163 | 316374004 | [V]Other family reason for encounter |
| HospitalAdmission | 7163 | 316375003 | [V]Family disruption |
| HospitalAdmission | 7163 | 316376002 | [V]Marital problems |
| HospitalAdmission | 7163 | 316377006 | [V]Child abuse |
| HospitalAdmission | 7163 | 316378001 | [V]Other parent-child problems |
| HospitalAdmission | 7163 | 316379009 | [V]Aged parents or in-law problems |
| HospitalAdmission | 7163 | 316380007 | [V]Health problems in family |
| HospitalAdmission | 7163 | 316381006 | [V]Multiparity |
| HospitalAdmission | 7163 | 316382004 | [V]Illegitimacy |

| Condition Name | Condition ID | Concept ID | Primary Term |
|---|---|---|---|
| HospitalAdmission | 7163 | 316383009 | [V]Illegitimate pregnancy |
| HospitalAdmission | 7163 | 316385002 | [V]Other unwanted pregnancy |
| HospitalAdmission | 7163 | 316387005 | [V]Other specified family reason for encounter |
| HospitalAdmission | 7163 | 316388000 | [V]Unspecified family reason for encounter |
| HospitalAdmission | 7163 | 316408003 | [V]Procedure planned but not carried out |
| HospitalAdmission | 7163 | 316409006 | [V]Vaccination not carried out because of contraindication |
| HospitalAdmission | 7163 | 316410001 | [V]Surgical or other procedure not carried out because of contraindication |
| HospitalAdmission | 7163 | 316411002 | [V]Surgical or other procedure not carried out because of patient decision |
| HospitalAdmission | 7163 | 316412009 | [V]No procedure - not indicated |
| HospitalAdmission | 7163 | 316413004 | [V]Operation not carried out as condition resolved |
| HospitalAdmission | 7163 | 316414005 | [V]Immunisation not carried out because of patient's decision for reasons of belief or group pressure |
| HospitalAdmission | 7163 | 316415006 | [V]Immunisation not carried out because of patient's decision for other unspecified reasons |
| HospitalAdmission | 7163 | 316416007 | [V]Immunisation not carried out for other reasons |
| HospitalAdmission | 7163 | 316417003 | [V]Immunisation not carried out for unspecified reason |
| HospitalAdmission | 7163 | 316420006 | [V]Procedure not carried out for other specified reason |
| HospitalAdmission | 7163 | 316421005 | [V]Procedure not carried out for unspecified reason |
| HospitalAdmission | 7163 | 316427009 | [V]Other counselling NEC |
| HospitalAdmission | 7163 | 316428004 | [V]Person with feared complaint, no diagnosis made |
| HospitalAdmission | 7163 | 316435007 | [V]Other specified encounter with person who has no complaint or sickness |
| HospitalAdmission | 7163 | 316436008 | [V]Unspecified encounter with person who has no complaint or sickness |
| HospitalAdmission | 7163 | 316487006 | [V]Person consulting for counselling or advice |
| HospitalAdmission | 7163 | 316488001 | [V]Counselling related to sexual attitude |
| HospitalAdmission | 7163 | 316489009 | [V]Counselling related to patient's sexual behaviour and orientation |
| HospitalAdmission | 7163 | 316490000 | [V]Counselling related to sexual behaviour and orientation of third party |
| HospitalAdmission | 7163 | 316491001 | [V]Counselling related to combined concerns regarding sexual attitude, behaviour and orientation |
| HospitalAdmission | 7163 | 316492008 | [V]Human immunodefiency virus counselling |
| HospitalAdmission | 7163 | 316493003 | [V]Person consulting for explanation of investigation findings |

| Condition Name | Condition ID | Concept ID | Primary Term |
|---|---|---|---|
| HospitalAdmission | 7163 | 316494009 | [V]Alcohol abuse counselling and surveillance |
| HospitalAdmission | 7163 | 316495005 | [V]Drug abuse counselling and surveillance |
| HospitalAdmission | 7163 | 316496006 | [V]Tobacco abuse counselling |
| HospitalAdmission | 7163 | 316497002 | [V]Other sex counselling |
| HospitalAdmission | 7163 | 316500008 | [V]Problems related to medical facilities and other health care |
| HospitalAdmission | 7163 | 316501007 | [V]Person awaiting admission to adequate facility elsewhere |
| HospitalAdmission | 7163 | 316502000 | [V]Unavailability and inaccessibility of other helping agencies |
| HospitalAdmission | 7163 | 316503005 | [V]Other problems related to medical facilities and other health care |
| HospitalAdmission | 7163 | 316504004 | [V]Unspecified problem related to medical facilities and other health care |
| HospitalAdmission | 7163 | 316515009 | [V]Acquired absence of organs NEC |
| HospitalAdmission | 7163 | 316516005 | [V]Acquired absence of part of head and neck |
| HospitalAdmission | 7163 | 316517001 | [V]Acquired absence of breast(s) |
| HospitalAdmission | 7163 | 316518006 | [V]Acquired absence of part of lung |
| HospitalAdmission | 7163 | 316519003 | [V]Acquired absence of part of stomach |
| HospitalAdmission | 7163 | 316520009 | [V]Acquired absence of other parts of digestive tract |
| HospitalAdmission | 7163 | 316521008 | [V]Acquired absence of kidney |
| HospitalAdmission | 7163 | 316522001 | [V]Acquired absence of other organs of urinary tract |
| HospitalAdmission | 7163 | 316523006 | [V]Acquired absence of genital organ(s) |
| HospitalAdmission | 7163 | 316524000 | [V]Acquired absence of other organs |
| HospitalAdmission | 7163 | 405614004 | Unexpected hospital admission |
| HospitalAdmission | 7163 | 408489005 | Respiratory emergency hospital admission |
| HospitalAdmission | 7163 | 408501008 | Emergency hospital admission for chronic obstructive pulmonary disease |
| HospitalAdmission | 7163 | 416683003 | Emergency hospital admission for heart failure |
| HospitalAdmission | 7163 | 699122005 | Vascular surgery emergency hospital admission |
| HospitalAdmission | 7163 | 699245006 | Emergency hospital admission for ischaemic heart disease |
| HospitalAdmission | 7163 | 3241000175106 | Hospital admission from non-health care facility |
| HospitalAdmission | 7163 | 188151000000107 | Admit heart failure emergency |
| HospitalAdmission | 7163 | 191651000000108 | Admit heart failure emergency |
| HospitalAdmission | 7163 | 313891000000105 | Emergency voluntary psychiatric admission under Mental Health Act |
| HospitalAdmission | 7163 | 313901000000106 | Emergency informal psychiatric admission |
| HospitalAdmission | 7163 | 313911000000108 | Emergency informal psychiatric admission |
| HospitalAdmission | 7163 | 320351000000105 | Admit ischaemic heart disease emergency |
| HospitalAdmission | 7163 | 320361000000108 | Emergency admission - ischaemic heart disease |
| HospitalAdmission | 7163 | 320371000000101 | Emergency admission - ischaemic heart disease |

| Condition Name | Condition ID | Concept ID | Primary Term |
|---|---|---|---|
| HospitalAdmission | 7163 | 369261000000102 | Admission to community hospital |
| HospitalAdmission | 7163 | 370181000000103 | Admission to community hospital |
| HospitalAdmission | 7163 | 395661000000100 | Emergency psychiatric admission under Mental Health Act 1983 (England and Wales) |
| HospitalAdmission | 7163 | 397941000000102 | [V]Other reasons for encounter |
| HospitalAdmission | 7163 | 398041000000100 | [V]Admitted for commencement of insulin |
| HospitalAdmission | 7163 | 398341000000102 | [V]Lack of housing |
| HospitalAdmission | 7163 | 399421000000108 | [V]Inadequate material resources |
| HospitalAdmission | 7163 | 400151000000105 | [V]Other counselling NEC |
| HospitalAdmission | 7163 | 400161000000108 | [V]Person with feared complaint, no diagnosis made |
| HospitalAdmission | 7163 | 400791000000103 | [V]Other parent-child problems |
| HospitalAdmission | 7163 | 401051000000100 | [V]Other social reason for encounter |
| HospitalAdmission | 7163 | 401171000000108 | [V] Admission for bladder training |
| HospitalAdmission | 7163 | 401651000000107 | [V]Procedure cancelled due to lack of theatre time |
| HospitalAdmission | 7163 | 402131000000102 | [V]Other unwanted pregnancy |
| HospitalAdmission | 7163 | 402291000000106 | [V]No able carer in household |
| HospitalAdmission | 7163 | 409891000000104 | [V]Other reasons for encounter OS |
| HospitalAdmission | 7163 | 410171000000104 | [V]Procedure cancelled due to non-availability of anaesthetist |
| HospitalAdmission | 7163 | 410371000000101 | [V]Housing, household and economic circumstances as reason for encounter |
| HospitalAdmission | 7163 | 410391000000102 | [V]Admission for respite care |
| HospitalAdmission | 7163 | 410801000000102 | [V]Illegitimate pregnancy |
| HospitalAdmission | 7163 | 411151000000102 | [V]Unspecified encounter with person who has no complaint or sickness |
| HospitalAdmission | 7163 | 411851000000109 | [X]Other boarder in health care facility |
| HospitalAdmission | 7163 | 412211000000100 | [V]Acquired absence of other parts of digestive tract |
| HospitalAdmission | 7163 | 413571000000101 | [V]Problems in relationship with parents and in-laws |
| HospitalAdmission | 7163 | 413761000000102 | [V]No procedure - not indicated |
| HospitalAdmission | 7163 | 414181000000105 | [V]Vaccination not carried out because of contraindication |
| HospitalAdmission | 7163 | 416411000000100 | [V]Procedure planned but not carried out |
| HospitalAdmission | 7163 | 416681000000109 | [V]Other specified reasons for encounter |
| HospitalAdmission | 7163 | 418621000000107 | [V]Admitted for commencement of insulin |
| HospitalAdmission | 7163 | 418871000000107 | [V]Institution resident |
| HospitalAdmission | 7163 | 418881000000109 | [V]Other sex counselling |
| HospitalAdmission | 7163 | 419371000000105 | [V]Person living alone |
| HospitalAdmission | 7163 | 420171000000106 | [V]Unspecified problem related to medical facilities and other health care |
| HospitalAdmission | 7163 | 426401000000108 | [V]Counselling related to patient's sexual behaviour and orientation |

| Condition Name | Condition ID | Concept ID | Primary Term |
|---|---|---|---|
| HospitalAdmission | 7163 | 427471000000102 | [V]Admission for instruction of self-catheterisation |
| HospitalAdmission | 7163 | 427601000000108 | [V]Procedure not carried out for unspecified reason |
| HospitalAdmission | 7163 | 427611000000105 | [V]Encounter with person who has no complaint or sickness |
| HospitalAdmission | 7163 | 427981000000109 | [V]Acquired absence of organs |
| HospitalAdmission | 7163 | 428231000000104 | [V]Other boarder in health-care facility |
| HospitalAdmission | 7163 | 428401000000109 | [V]Problems in relationship with parents and in-laws |
| HospitalAdmission | 7163 | 428511000000105 | [V]Illegitimacy |
| HospitalAdmission | 7163 | 429351000000109 | [V]Inadequate housing |
| HospitalAdmission | 7163 | 429841000000103 | [V]Acquired absence of other organs of urinary tract |
| HospitalAdmission | 7163 | 430251000000105 | [V]Unspecified reasons for encounter |
| HospitalAdmission | 7163 | 430531000000101 | [V]Child abuse |
| HospitalAdmission | 7163 | 430541000000105 | [V]Immunisation not carried out for unspecified reason |
| HospitalAdmission | 7163 | 430751000000103 | [V]Other family reason for encounter |
| HospitalAdmission | 7163 | 431101000000109 | [V]Admission for instruction in the use of a nebuliser |
| HospitalAdmission | 7163 | 431521000000106 | [V]Sheltered housing |
| HospitalAdmission | 7163 | 431921000000103 | [V]Unspecified family reason for encounter |
| HospitalAdmission | 7163 | 432621000124105 | Hospital admission from dialysis facility |
| HospitalAdmission | 7163 | 432661000000106 | [V]Marital problems |
| HospitalAdmission | 7163 | 440111000000104 | [V]Person consulting for counselling or advice |
| HospitalAdmission | 7163 | 440431000000103 | [V]Person with other specified health problems |
| HospitalAdmission | 7163 | 440731000000106 | [V]Health problems in family |
| HospitalAdmission | 7163 | 440741000000102 | [V]Healthy person accompanying sick person |
| HospitalAdmission | 7163 | 441901000000108 | [V]Dietary surveillance and counselling |
| HospitalAdmission | 7163 | 442701000000101 | [V]Unspecified social reason for encounter |
| HospitalAdmission | 7163 | 442711000000104 | [V]Immunisation not carried out because of patient's decision for reasons of belief or group pressure |
| HospitalAdmission | 7163 | 442721000000105 | [V]Problems related to medical facilities and other health care |
| HospitalAdmission | 7163 | 443421000000107 | [V]Carer unable to cope |
| HospitalAdmission | 7163 | 443861000000102 | [V]Holiday relief care |
| HospitalAdmission | 7163 | 443871000000109 | [V]Surgical or other procedure not carried out because of contraindication |
| HospitalAdmission | 7163 | 443901000000109 | [V]Drug abuse counselling and surveillance |
| HospitalAdmission | 7163 | 444741000000107 | [V]Family disruption |
| HospitalAdmission | 7163 | 451501000000101 | [V]Counselling related to sexual attitude |
| HospitalAdmission | 7163 | 451961000000105 | [V]Other reasons for encounter NOS |

| Condition Name | Condition ID | Concept ID | Primary Term |
|---|---|---|---|
| HospitalAdmission | 7163 | 451991000000104 | [V]Other specified encounter with person who has no complaint or sickness |
| HospitalAdmission | 7163 | 452011000000104 | [V]Acquired absence of other organs |
| HospitalAdmission | 7163 | 452111000000103 | [V]Problem was normal state |
| HospitalAdmission | 7163 | 452221000000100 | [V]Multiparity |
| HospitalAdmission | 7163 | 452441000000106 | [V]Procedure cancelled due to lack of theatre time |
| HospitalAdmission | 7163 | 452491000000101 | [V]Aged parents or in-law problems |
| HospitalAdmission | 7163 | 452561000000106 | [V]Genetic counselling |
| HospitalAdmission | 7163 | 453041000000106 | [V] Admission for bladder training |
| HospitalAdmission | 7163 | 454911000000106 | [V]Problem was normal state |
| HospitalAdmission | 7163 | 455051000000105 | [V]Admitted for conversion to insulin |
| HospitalAdmission | 7163 | 455711000000108 | [V]Acquired absence of organs NEC |
| HospitalAdmission | 7163 | 456641000000105 | [V]Other problems related to medical facilities and other health care |
| HospitalAdmission | 7163 | 463631000000103 | [V]Admitted for conversion to insulin |
| HospitalAdmission | 7163 | 464391000000106 | [V]Unspecified health problems |
| HospitalAdmission | 7163 | 464741000000102 | [V]Person consulting for explanation of investigation findings |
| HospitalAdmission | 7163 | 465741000000103 | [V]Admission for respite care |
| HospitalAdmission | 7163 | 465751000000100 | [V]Procedure cancelled due to non-availability of anaesthetist |
| HospitalAdmission | 7163 | 467311000000108 | [V]Acquired absence of part of head and neck |
| HospitalAdmission | 7163 | 469761000000100 | [V]Admission for instruction of self-catheterisation |
| HospitalAdmission | 7163 | 469991000000109 | [V]Procedure not carried out for other specified reason |
| HospitalAdmission | 7163 | 470201000000106 | [V]Operation not carried out as condition resolved |
| HospitalAdmission | 7163 | 470891000000100 | [V]Surgical or other procedure not carried out because of patient decision |
| HospitalAdmission | 7163 | 471181000000101 | [V]Other specified family reason for encounter |
| HospitalAdmission | 7163 | 472141000000100 | [V]Immunisation not carried out because of patient's decision for other unspecified reasons |
| HospitalAdmission | 7163 | 472151000000102 | [V]Immunisation not carried out for other reasons |
| HospitalAdmission | 7163 | 479091000000102 | [X]Persons encountering health services in other specified circumstances |
| HospitalAdmission | 7163 | 479851000000104 | [V]Counselling related to sexual behaviour and orientation of third party |
| HospitalAdmission | 7163 | 479861000000101 | [V]Admission for instruction in the use of a nebuliser |
| HospitalAdmission | 7163 | 480041000000107 | [V]Counselling related to combined concerns regarding sexual attitude, behaviour and orientation |

| Condition Name | Condition ID | Concept ID | Primary Term |
|---|---|---|---|
| HospitalAdmission | 7163 | 511661000000100 | Admit vascular surgery emergency |
| HospitalAdmission | 7163 | 511671000000107 | Admit vascular surgery emergency |
| HospitalAdmission | 7163 | 623231000000108 | Other hospital admission NOS |
| HospitalAdmission | 7163 | 629551000000101 | Admit surgical emergency unspecified |
| HospitalAdmission | 7163 | 646311000000102 | Admit hospital emergency NOS |
| HospitalAdmission | 7163 | 646321000000108 | Non-urgent hospital admission unspecified |
| HospitalAdmission | 7163 | 656151000000103 | Admit medical emergency unspecified |
| HospitalAdmission | 7163 | 780831000000103 | Admission to accident and emergency department |
| HospitalAdmission | 7163 | 814141000000105 | Hospital re-admission |
| HospitalAdmission | 7163 | 814151000000108 | Hospital re-admission |
| HospitalAdmission | 7163 | 831421000000102 | Admission to day ward |
| HospitalAdmission | 7163 | 842951000000109 | Emergency hospital admission from walk-in centre |
| HospitalAdmission | 7163 | 842961000000107 | Emergency admission from walk in centre |
| HospitalAdmission | 7163 | 848101000000108 | Admit anticoagulation emergency |
| HospitalAdmission | 7163 | 848111000000105 | Admit anticoagulation emergency |
| HospitalAdmission | 7163 | 851901000006112 | Elderly psychiatric emergency admission |
| HospitalAdmission | 7163 | 894881000000108 | Admission to observation ward |
| HospitalAdmission | 7163 | 894891000000105 | Admission to observation ward |
| HospitalAdmission | 7163 | 1077941000000106 | Emergency hospital admission to maxillofacial surgery service |
| HospitalAdmission | 7163 | 1077981000000103 | Emergency hospital admission to cardiac surgery service |
| HospitalAdmission | 7163 | 1077991000000101 | Emergency hospital admission to thoracic surgery service |
| HospitalAdmission | 7163 | 1078031000000104 | Emergency hospital admission to community paediatric service |
| HospitalAdmission | 7163 | 1078041000000108 | Emergency hospital admission to general surgical service |
| HospitalAdmission | 7163 | 1078051000000106 | Emergency hospital admission to transplantation surgery service |
| HospitalAdmission | 7163 | 1078061000000109 | Emergency hospital admission to breast surgery service |
| HospitalAdmission | 7163 | 1078071000000102 | Emergency hospital admission to colorectal surgery service |
| HospitalAdmission | 7163 | 1078081000000100 | Emergency hospital admission to cardiothoracic transplantation surgery service |
| HospitalAdmission | 7163 | 1078091000000103 | Emergency hospital admission to pain management service |
| HospitalAdmission | 7163 | 1078101000000106 | Emergency hospital admission to paediatric urology service |
| HospitalAdmission | 7163 | 1078111000000108 | Emergency hospital admission to paediatric gastrointestinal surgery service |

| Condition Name | Condition ID | Concept ID | Primary Term |
|---|---|---|---|
| HospitalAdmission | 7163 | 1078121000000102 | Emergency hospital admission to paediatric ear, nose and throat service |
| HospitalAdmission | 7163 | 1078131000000100 | Emergency hospital admission to paediatric ophthalmology service |
| HospitalAdmission | 7163 | 1078141000000109 | Emergency hospital admission to paediatric maxillofacial surgery service |
| HospitalAdmission | 7163 | 1078151000000107 | Emergency hospital admission to paediatric neurosurgery service |
| HospitalAdmission | 7163 | 1078161000000105 | Emergency hospital admission to paediatric plastic surgery service |
| HospitalAdmission | 7163 | 1078171000000103 | Emergency hospital admission to paediatric cardiac surgery service |
| HospitalAdmission | 7163 | 1078181000000101 | Emergency hospital admission to paediatric thoracic surgery service |
| HospitalAdmission | 7163 | 1078191000000104 | Emergency hospital admission to paediatric pain management service |
| HospitalAdmission | 7163 | 1078201000000102 | Emergency hospital admission to paediatric gastroenterology service |
| HospitalAdmission | 7163 | 1078211000000100 | Emergency hospital admission to paediatric endocrinology service |
| HospitalAdmission | 7163 | 1078221000000106 | Emergency hospital admission to paediatric dermatology service |
| HospitalAdmission | 7163 | 1078231000000108 | Emergency hospital admission to paediatric respiratory medicine service |
| HospitalAdmission | 7163 | 1078241000000104 | Emergency hospital admission to paediatric nephrology service |
| HospitalAdmission | 7163 | 1078251000000101 | Emergency hospital admission to paediatric rheumatology service |
| HospitalAdmission | 7163 | 1078261000000103 | Emergency hospital admission to gastroenterology service |
| HospitalAdmission | 7163 | 1078271000000105 | Emergency hospital admission to endocrinology service |
| HospitalAdmission | 7163 | 1078281000000107 | Emergency hospital admission to critical care medicine service |
| HospitalAdmission | 7163 | 1078291000000109 | Emergency hospital admission to general medical service |
| HospitalAdmission | 7163 | 1078301000000108 | Emergency hospital admission to hepatology service |
| HospitalAdmission | 7163 | 1078311000000105 | Emergency hospital admission to palliative medicine service |
| HospitalAdmission | 7163 | 1078321000000104 | Emergency hospital admission to respite care service |
| HospitalAdmission | 7163 | 1078331000000102 | Emergency hospital admission to intermediate care service |
| HospitalAdmission | 7163 | 1078341000000106 | Emergency hospital admission to paediatric cardiology service |

| Condition Name | Condition ID | Concept ID | Primary Term |
|---|---|---|---|
| HospitalAdmission | 7163 | 1078351000000109 | Emergency hospital admission to cardiac rehabilitation service |
| HospitalAdmission | 7163 | 1078531000000105 | Emergency hospital admission to paediatric dentistry service |
| HospitalAdmission | 7163 | 1078541000000101 | Emergency hospital admission to orthodontics service |
| HospitalAdmission | 7163 | 1078551000000103 | Emergency hospital admission to paediatric surgical service |
| HospitalAdmission | 7163 | 1078561000000100 | Emergency hospital admission to paediatric intensive care service |
| HospitalAdmission | 7163 | 1078571000000107 | Emergency hospital admission to trauma and orthopaedics service |
| HospitalAdmission | 7163 | 1078581000000109 | Emergency hospital admission to paediatric transplantation surgery service |
| HospitalAdmission | 7163 | 1078591000000106 | Emergency hospital admission to paediatric trauma and orthopaedics surgery service |
| HospitalAdmission | 7163 | 1078601000000100 | Emergency hospital admission to paediatric medical oncology service |
| HospitalAdmission | 7163 | 1078611000000103 | Emergency hospital admission to paediatric diabetes service |
| HospitalAdmission | 7163 | 1078621000000109 | Emergency hospital admission to paediatric interventional radiology service |
| HospitalAdmission | 7163 | 1078631000000106 | Emergency hospital admission to paediatric neurodisability service |
| HospitalAdmission | 7163 | 1078641000000102 | Emergency hospital admission to clinical pharmacology service |
| HospitalAdmission | 7163 | 1078651000000104 | Emergency hospital admission to haemophilia service |
| HospitalAdmission | 7163 | 1078661000000101 | Emergency hospital admission to clinical immunology and allergy service |
| HospitalAdmission | 7163 | 1078671000000108 | Emergency hospital admission to stroke service |
| HospitalAdmission | 7163 | 1078681000000105 | Emergency hospital admission to genitourinary medicine service |
| HospitalAdmission | 7163 | 1078691000000107 | Emergency hospital admission to clinical neurophysiology service |
| HospitalAdmission | 7163 | 1078701000000107 | Emergency hospital admission to podiatric surgery service |
| HospitalAdmission | 7163 | 1078711000000109 | Emergency hospital admission to midwifery service |
| HospitalAdmission | 7163 | 1078721000000103 | Emergency hospital admission to forensic psychiatry service |
| HospitalAdmission | 7163 | 1078731000000101 | Emergency hospital admission to eating disorders service |
| HospitalAdmission | 7163 | 1078741000000105 | Emergency hospital admission to clinical oncology service |
| HospitalAdmission | 7163 | 1078751000000108 | Emergency hospital admission to interventional radiology service |

| Condition Name | Condition ID | Concept ID | Primary Term |
|---|---|---|---|
| HospitalAdmission | 7163 | 1078761000000106 | Emergency hospital admission to upper gastrointestinal surgery service |
| HospitalAdmission | 7163 | 1078771000000104 | Emergency hospital admission to restorative dentistry service |
| HospitalAdmission | 7163 | 1078781000000102 | Emergency hospital admission to tropical medicine service |
| HospitalAdmission | 7163 | 1078791000000100 | Emergency hospital admission to medical oncology service |
| HospitalAdmission | 7163 | 1078801000000101 | Emergency hospital admission to nuclear medicine service |
| HospitalAdmission | 7163 | 1078811000000104 | Emergency hospital admission to psychiatric intensive care service |
| HospitalAdmission | 7163 | 1078821000000105 | Emergency hospital admission to child and adolescent psychiatry service |
| HospitalAdmission | 7163 | 1078831000000107 | Emergency hospital admission to gynaecological oncology service |
| HospitalAdmission | 7163 | 1078841000000103 | Emergency hospital admission to medical ophthalmology service |
| HospitalAdmission | 7163 | 1078851000000100 | Emergency hospital admission to paediatric burn care service |
| HospitalAdmission | 7163 | 1078861000000102 | Emergency hospital admission to clinical immunology service |
| HospitalAdmission | 7163 | 1078871000000109 | Emergency hospital admission to allergy service |
| HospitalAdmission | 7163 | 1078881000000106 | Emergency hospital admission to spinal injuries service |
| HospitalAdmission | 7163 | 1078891000000108 | Emergency hospital admission to respiratory physiology service |
| HospitalAdmission | 7163 | 1079141000000103 | Emergency hospital admission to addiction service |
| HospitalAdmission | 7163 | 1079151000000100 | Emergency hospital admission to burn care service |
| HospitalAdmission | 7163 | 1079161000000102 | Emergency hospital admission to paediatric clinical haematology service |
| HospitalAdmission | 7163 | 1079171000000109 | Emergency hospital admission to paediatric infectious diseases service |
| HospitalAdmission | 7163 | 1079181000000106 | Emergency hospital admission to paediatric metabolic disease service |
| HospitalAdmission | 7163 | 1079191000000108 | Emergency hospital admission to paediatric cystic fibrosis service |
| HospitalAdmission | 7163 | 1079201000000105 | Emergency hospital admission to transient ischaemic attack service |
| HospitalAdmission | 7163 | 1079211000000107 | Emergency hospital admission to adult cystic fibrosis service |
| HospitalAdmission | 7163 | 1079221000000101 | Emergency hospital admission to infectious diseases service |
| HospitalAdmission | 7163 | 1079231000000104 | Emergency hospital admission to paediatric neurology service |

| Condition Name | Condition ID | Concept ID | Primary Term |
|---|---|---|---|
| HospitalAdmission | 7163 | 1079241000000108 | Emergency hospital admission to neonatal critical care service |
| HospitalAdmission | 7163 | 1079251000000106 | Emergency hospital admission to psychotherapy service |
| HospitalAdmission | 7163 | 1079261000000109 | Emergency hospital admission to perinatal psychiatry service |
| HospitalAdmission | 7163 | 1079271000000102 | Emergency hospital admission to hepatobiliary and pancreatic surgery service |
| HospitalAdmission | 7163 | 1079281000000100 | Emergency hospital admission to spinal surgery service |
| HospitalAdmission | 7163 | 1079291000000103 | Emergency hospital admission to paediatric epilepsy service |
| HospitalAdmission | 7163 | 1079301000000104 | Emergency hospital admission to paediatric audiological medicine service |
| HospitalAdmission | 7163 | 1079311000000102 | Emergency hospital admission to paediatric clinical immunology and allergy service |
| HospitalAdmission | 7163 | 1079321000000108 | Emergency hospital admission to blood and marrow transplantation service |
| HospitalAdmission | 7163 | 1079331000000105 | Emergency hospital admission to audiological medicine service |
| HospitalAdmission | 7163 | 1079341000000101 | Emergency hospital admission to clinical genetics service |
| HospitalAdmission | 7163 | 1079351000000103 | Emergency hospital admission to rehabilitation service |
| HospitalAdmission | 7163 | 1079361000000100 | Emergency hospital admission to sport and exercise medicine service |
| HospitalAdmission | 7163 | 1079371000000107 | Emergency hospital admission to congenital heart disease service |
| HospitalAdmission | 7163 | 1082351000000104 | Emergency hospital admission to adult mental health service |
| HospitalAdmission | 7163 | 1082361000000101 | Emergency hospital admission to complex specialised rehabilitation service |
| HospitalAdmission | 7163 | 1082371000000108 | Emergency hospital admission to dental medicine specialties service |
| HospitalAdmission | 7163 | 1082381000000105 | Emergency hospital admission to learning disability service |
| HospitalAdmission | 7163 | 1082391000000107 | Emergency hospital admission to local specialist rehabilitation service |
| HospitalAdmission | 7163 | 1082401000000105 | Emergency hospital admission to programmed pulmonary rehabilitation service |
| HospitalAdmission | 7163 | 1082411000000107 | Emergency hospital admission to well babies specialty |
| HospitalAdmission | 7163 | 1082421000000101 | Emergency hospital admission to accident and emergency service |
| HospitalAdmission | 7163 | 1823531000006111 | Hospital admission, emergency, from walk-in centre |
| HospitalAdmission | 7163 | 1823541000006118 | Admission by own GP |

| Condition Name | Condition ID | Concept ID | Primary Term |
|---|---|---|---|
| HospitalAdmission | 7163 | 1823551000006116 | Admission by GP partner |
| HospitalAdmission | 7163 | 1880401000006119 | Admission to observation ward |
| Influenza-likeIllness-WRpt | 1020 | 6142004 | Influenza |
| Influenza-likeIllness-WRpt | 1020 | 24662006 | Influenza due to Influenza B virus |
| Influenza-likeIllness-WRpt | 1020 | 41269000 | Influenzal bronchopneumonia |
| Influenza-likeIllness-WRpt | 1020 | 42964004 | Influenza with pneumonia |
| Influenza-likeIllness-WRpt | 1020 | 43692000 | Influenzal acute upper respiratory infection |
| Influenza-likeIllness-WRpt | 1020 | 46171006 | Influenza due to Influenza virus, type A, porcine |
| Influenza-likeIllness-WRpt | 1020 | 55604004 | Avian influenza |
| Influenza-likeIllness-WRpt | 1020 | 61700007 | Influenza with non-respiratory manifestation |
| Influenza-likeIllness-WRpt | 1020 | 63039003 | Influenza with respiratory manifestation other than pneumonia |
| Influenza-likeIllness-WRpt | 1020 | 74644004 | Influenza with encephalopathy |
| Influenza-likeIllness-WRpt | 1020 | 78046005 | Myocarditis due to influenza virus |
| Influenza-likeIllness-WRpt | 1020 | 78431007 | Influenza due to Influenza virus, type A, human |
| Influenza-likeIllness-WRpt | 1020 | 81524006 | Influenza due to Influenza C virus |
| Influenza-likeIllness-WRpt | 1020 | 84037004 | Swine influenza |
| Influenza-likeIllness-WRpt | 1020 | 95891005 | Influenza-like illness |
| Influenza-likeIllness-WRpt | 1020 | 139168000 | Influenza-like symptoms |
| Influenza-likeIllness-WRpt | 1020 | 161913008 | Influenza-like symptoms |
| Influenza-likeIllness-WRpt | 1020 | 194946005 | Acute myocarditis - influenzal |
| Influenza-likeIllness-WRpt | 1020 | 195878008 | Pneumonia and influenza |
| Influenza-likeIllness-WRpt | 1020 | 195920000 | Influenza with pneumonia, influenza virus identified |
| Influenza-likeIllness-WRpt | 1020 | 195921001 | Influenza with pneumonia NOS |
| Influenza-likeIllness-WRpt | 1020 | 195922008 | Influenza with other respiratory manifestation |

| Condition Name | Condition ID | Concept ID | Primary Term |
|---|---|---|---|
| Influenza-likeIllness-WRpt | 1020 | 195923003 | Influenza with laryngitis |
| Influenza-likeIllness-WRpt | 1020 | 195924009 | Influenza with pharyngitis |
| Influenza-likeIllness-WRpt | 1020 | 195925005 | Influenza with respiratory manifestations NOS |
| Influenza-likeIllness-WRpt | 1020 | 195927002 | Influenza with other manifestations |
| Influenza-likeIllness-WRpt | 1020 | 195929004 | Influenza with gastrointestinal tract involvement |
| Influenza-likeIllness-WRpt | 1020 | 195930009 | Influenza with other manifestations NOS |
| Influenza-likeIllness-WRpt | 1020 | 195933006 | Other specified pneumonia or influenza |
| Influenza-likeIllness-WRpt | 1020 | 195934000 | Pneumonia or influenza NOS |
| Influenza-likeIllness-WRpt | 1020 | 196200002 | [X]Influenza with other respiratory manifestations, influenza virus identified |
| Influenza-likeIllness-WRpt | 1020 | 196201003 | [X]Influenza with other manifestations, influenza virus identified |
| Influenza-likeIllness-WRpt | 1020 | 196202005 | [X]Influenza with other respiratory manifestations, virus not identified |
| Influenza-likeIllness-WRpt | 1020 | 196203000 | [X]Influenza with other manifestations, virus not identified |
| Influenza-likeIllness-WRpt | 1020 | 266353003 | Influenza NOS |
| Influenza-likeIllness-WRpt | 1020 | 274104008 | Flu-like illness NOS |
| Influenza-likeIllness-WRpt | 1020 | 309789002 | Encephalitis due to influenza |
| Influenza-likeIllness-WRpt | 1020 | 309806000 | Encephalitis due to influenza-virus identified |
| Influenza-likeIllness-WRpt | 1020 | 313251006 | Encephalitis due to influenza-specific virus not identified |
| Influenza-likeIllness-WRpt | 1020 | 315642008 | Influenza-like symptoms |
| Influenza-likeIllness-WRpt | 1020 | 408687004 | Healthcare associated influenza disease |
| Influenza-likeIllness-WRpt | 1020 | 427873006 | Influenza due to influenza virus type A, avian, H5N1 strain |
| Influenza-likeIllness-WRpt | 1020 | 442438000 | Influenza due to Influenza A virus |
| Influenza-likeIllness-WRpt | 1020 | 442696006 | Influenza due to Influenza A virus subtype H1N1 |
| Influenza-likeIllness-WRpt | 1020 | 450715004 | Influenza due to Influenza A virus subtype H7 |

| Condition Name | Condition ID | Concept ID | Primary Term |
|---|---|---|---|
| Influenza-likeIllness-WRpt | 1020 | 450716003 | Influenza due to Influenza A virus subtype H9 |
| Influenza-likeIllness-WRpt | 1020 | 707448003 | Influenza due to Influenza A virus subtype H7N9 |
| Influenza-likeIllness-WRpt | 1020 | 711128004 | Influenza due to influenza virus type A, avian, H3N2 strain |
| Influenza-likeIllness-WRpt | 1020 | 713083002 | Influenza caused by Influenza A virus subtype H5 |
| Influenza-likeIllness-WRpt | 1020 | 719590007 | Influenza caused by seasonal influenza virus |
| Influenza-likeIllness-WRpt | 1020 | 719865001 | Influenza caused by pandemic influenza virus |
| Influenza-likeIllness-WRpt | 1020 | 772810003 | Influenza caused by Influenza A virus subtype H3N2 |
| Influenza-likeIllness-WRpt | 1020 | 772828001 | Influenza caused by Influenza A virus subtype H5N1 |
| Influenza-likeIllness-WRpt | 1020 | 772839003 | Pneumonia caused by Influenza A virus |
| Influenza-likeIllness-WRpt | 1020 | 1149091008 | Influenza caused by Influenza A virus subtype H2 |
| Influenza-likeIllness-WRpt | 1020 | 16311000119108 | Pneumonia due to influenza |
| Influenza-likeIllness-WRpt | 1020 | 142921000119103 | Upper respiratory tract infection due to avian influenza |
| Influenza-likeIllness-WRpt | 1020 | 142931000119100 | Pneumonia due to H1N1 influenza |
| Influenza-likeIllness-WRpt | 1020 | 142941000119109 | Upper respiratory tract infection due to H1N1 influenza |
| Influenza-likeIllness-WRpt | 1020 | 142951000119106 | Myocarditis due to Influenza A virus subtype H1N1 |
| Influenza-likeIllness-WRpt | 1020 | 142961000119108 | Gastroenteritis due to H1N1 influenza |
| Influenza-likeIllness-WRpt | 1020 | 142981000119104 | Myocarditis due to avian influenza |
| Influenza-likeIllness-WRpt | 1020 | 142991000119101 | Gastroenteritis due to avian influenza |
| Influenza-likeIllness-WRpt | 1020 | 143111000119103 | Pneumonia due to avian influenza |
| Influenza-likeIllness-WRpt | 1020 | 280331000000102 | Avian influenza |
| Influenza-likeIllness-WRpt | 1020 | 292631000000106 | Avian influenza |
| Influenza-likeIllness-WRpt | 1020 | 328531000119104 | Upper respiratory tract infection due to Influenza A |
| Influenza-likeIllness-WRpt | 1020 | 418181000000104 | [X]Influenza with other respiratory manifestations, influenza virus identified |

| Condition Name | Condition ID | Concept ID | Primary Term |
|---|---|---|---|
| Influenza-likeIllness-WRpt | 1020 | 418191000000102 | [X]Influenza with other manifestations, influenza virus identified |
| Influenza-likeIllness-WRpt | 1020 | 430891000000103 | [X]Influenza with other respiratory manifestations, virus not identified |
| Influenza-likeIllness-WRpt | 1020 | 441131000000104 | [X]Influenza with other manifestations, virus not identified |
| Influenza-likeIllness-WRpt | 1020 | 505131000000104 | Influenza due to Influenza A virus subtype H1N1 |
| Influenza-likeIllness-WRpt | 1020 | 510671000000104 | Influenza due to Influenza A virus subtype H1N1 |
| Influenza-likeIllness-WRpt | 1020 | 540121000000103 | Influenza with other manifestations |
| Influenza-likeIllness-WRpt | 1020 | 540131000000101 | Influenza with other manifestations NOS |
| Influenza-likeIllness-WRpt | 1020 | 540141000000105 | Other specified pneumonia or influenza |
| Influenza-likeIllness-WRpt | 1020 | 540151000000108 | Pneumonia or influenza NOS |
| Influenza-likeIllness-WRpt | 1020 | 616161000000107 | Influenza with pneumonia NOS |
| Influenza-likeIllness-WRpt | 1020 | 616171000000100 | Influenza with other respiratory manifestation |
| Influenza-likeIllness-WRpt | 1020 | 616181000000103 | Influenza with respiratory manifestations NOS |
| Influenza-likeIllness-WRpt | 1020 | 670551000000108 | Influenza NOS |
| Influenza-likeIllness-WRpt | 1020 | 677811000000106 | Flu-like illness NOS |
| Influenza-likeIllness-WRpt | 1020 | 856211000006111 | Post influenzal debility |
| Influenza-likeIllness-WRpt | 1020 | 1033051000000101 | Influenza due to zoonotic influenza virus |
| Influenza-likeIllness-WRpt | 1020 | 1033061000000103 | Influenza due to zoonotic influenza virus |
| Influenza-likeIllness-WRpt | 1020 | 1033071000000105 | Influenza due to pandemic influenza virus |
| Influenza-likeIllness-WRpt | 1020 | 1033081000000107 | Influenza due to pandemic influenza virus |
| Influenza-likeIllness-WRpt | 1020 | 1033091000000109 | Influenza due to seasonal influenza virus |
| Influenza-likeIllness-WRpt | 1020 | 1033101000000101 | Influenza due to seasonal influenza virus |
| Influenza-likeIllness-WRpt | 1020 | 1033111000000104 | Influenza with pneumonia due to seasonal influenza virus |
| Influenza-likeIllness-WRpt | 1020 | 1033121000000105 | Influenzal bronchopneumonia due to seasonal influenza virus |

| Condition Name | Condition ID | Concept ID | Primary Term |
|---|---|---|---|
| Influenza-likeIllness-WRpt | 1020 | 1050601000000101 | Influenza due to seasonal influenza virus |
| Influenza-likeIllness-WRpt | 1020 | 1050981000000100 | Influenza due to seasonal influenza virus |
| Influenza-likeIllness-WRpt | 1020 | 1787121000006116 | Community acquired pneumonia |
| Influenza-likeIllness-WRpt | 1020 | 1787131000006118 | Hospital acquired pneumonia |
| Influenza-likeIllness-WRpt | 1020 | 10628871000119101 | Gastroenteritis due to influenza |
| Influenza-likeIllness-WRpt | 1020 | 10628911000119103 | Gastroenteritis due to Influenza A virus |
| Influenza-likeIllness-WRpt | 1020 | 10629191000119100 | Bronchiolitis caused by influenza virus |
| Influenza-likeIllness-WRpt | 1020 | 10629351000119108 | Myocarditis due to Influenza A virus |
| Influenza-likeIllness-WRpt | 1020 | 10685111000119102 | Upper respiratory tract infection due to Influenza |
| LowerRespiratoryTract Infection-WRpt | 1055 | 181007 | Haemorrhagic bronchopneumonia |
| LowerRespiratoryTract Infection-WRpt | 1055 | 718004 | Acute bronchiolitis with obstruction |
| LowerRespiratoryTract Infection-WRpt | 1055 | 2087000 | Pulmonary nocardiosis |
| LowerRespiratoryTract Infection-WRpt | 1055 | 2523007 | Salmonella pneumonia |
| LowerRespiratoryTract Infection-WRpt | 1055 | 2585002 | Pneumococcal pleurisy |
| LowerRespiratoryTract Infection-WRpt | 1055 | 3144005 | Staphylococcal pleurisy |
| LowerRespiratoryTract Infection-WRpt | 1055 | 3214003 | Invasive pulmonary aspergillosis |
| LowerRespiratoryTract Infection-WRpt | 1055 | 3487004 | Candidiasis of lung |
| LowerRespiratoryTract Infection-WRpt | 1055 | 4120002 | Bronchiolitis |
| LowerRespiratoryTract Infection-WRpt | 1055 | 5505005 | Acute bronchiolitis |
| LowerRespiratoryTract Infection-WRpt | 1055 | 5875001 | Acute bronchitis with obstruction |
| LowerRespiratoryTract Infection-WRpt | 1055 | 6042001 | Pulmonary aspergillosis |
| LowerRespiratoryTract Infection-WRpt | 1055 | 7063008 | Gangrenous pneumonia |
| LowerRespiratoryTract Infection-WRpt | 1055 | 7097001 | Streptococcal pleurisy with effusion |

| Condition Name | Condition ID | Concept ID | Primary Term |
|---|---|---|---|
| LowerRespiratoryTract Infection-WRpt | 1055 | 7238003 | Jaagziekte |
| LowerRespiratoryTract Infection-WRpt | 1055 | 7548000 | Rheumatic pneumonia |
| LowerRespiratoryTract Infection-WRpt | 1055 | 7678002 | Cytomegaloviral pneumonia |
| LowerRespiratoryTract Infection-WRpt | 1055 | 8555001 | Syphilis of lung |
| LowerRespiratoryTract Infection-WRpt | 1055 | 9095002 | Adiaspiromycosis due to Emmonsia crescens |
| LowerRespiratoryTract Infection-WRpt | 1055 | 9228003 | Pulmonary schistosomiasis |
| LowerRespiratoryTract Infection-WRpt | 1055 | 9505000 | Infestation by Pneumonyssus simicola |
| LowerRespiratoryTract Infection-WRpt | 1055 | 10446001 | Aspergilloma |
| LowerRespiratoryTract Infection-WRpt | 1055 | 10509002 | Acute bronchitis |
| LowerRespiratoryTract Infection-WRpt | 1055 | 11389007 | Inhalational anthrax |
| LowerRespiratoryTract Infection-WRpt | 1055 | 11885007 | Corynebacterial pneumonia of foals |
| LowerRespiratoryTract Infection-WRpt | 1055 | 13089009 | Adenoviral bronchiolitis |
| LowerRespiratoryTract Infection-WRpt | 1055 | 14527007 | Tuberculous empyema |
| LowerRespiratoryTract Infection-WRpt | 1055 | 15199004 | Acute bronchiolitis with bronchospasm |
| LowerRespiratoryTract Infection-WRpt | 1055 | 15341009 | Atypical interstitial pneumonia of cattle |
| LowerRespiratoryTract Infection-WRpt | 1055 | 16146001 | Viral bronchitis |
| LowerRespiratoryTract Infection-WRpt | 1055 | 16810008 | AIDS with viral pneumonia |
| LowerRespiratoryTract Infection-WRpt | 1055 | 18988001 | Septic pleurisy |
| LowerRespiratoryTract Infection-WRpt | 1055 | 19287005 | North American pulmonary blastomycosis |
| LowerRespiratoryTract Infection-WRpt | 1055 | 20953001 | Pulmonary cryptococcosis |
| LowerRespiratoryTract Infection-WRpt | 1055 | 21846001 | Pulmonary actinomycosis |
| LowerRespiratoryTract Infection-WRpt | 1055 | 22754005 | Staphylococcal pneumonia |
| LowerRespiratoryTract Infection-WRpt | 1055 | 23698009 | Staphylococcal pleurisy with effusion |

| Condition Name | Condition ID | Concept ID | Primary Term |
|---|---|---|---|
| LowerRespiratoryTract Infection-WRpt | 1055 | 24235005 | Tuberculous hydrothorax |
| LowerRespiratoryTract Infection-WRpt | 1055 | 24302002 | Pneumococcal pleurisy with effusion |
| LowerRespiratoryTract Infection-WRpt | 1055 | 25042006 | Tracheostomy sepsis |
| LowerRespiratoryTract Infection-WRpt | 1055 | 26427008 | Chronic pulmonary histoplasmosis |
| LowerRespiratoryTract Infection-WRpt | 1055 | 27475006 | Parainfluenza virus bronchitis |
| LowerRespiratoryTract Infection-WRpt | 1055 | 27757009 | Encysted pleurisy |
| LowerRespiratoryTract Infection-WRpt | 1055 | 27836007 | Pertussis |
| LowerRespiratoryTract Infection-WRpt | 1055 | 28085001 | Empyema with bronchopleural fistula |
| LowerRespiratoryTract Infection-WRpt | 1055 | 29591002 | Purulent bronchitis |
| LowerRespiratoryTract Infection-WRpt | 1055 | 29731002 | Tuberculous pneumothorax |
| LowerRespiratoryTract Infection-WRpt | 1055 | 30437004 | Empyema with bronchocutaneous fistula |
| LowerRespiratoryTract Infection-WRpt | 1055 | 31561003 | Hypostatic bronchopneumonia |
| LowerRespiratoryTract Infection-WRpt | 1055 | 31920006 | Haemorrhagic varicella pneumonitis |
| LowerRespiratoryTract Infection-WRpt | 1055 | 32204007 | Pulmonary actinobacillosis |
| LowerRespiratoryTract Infection-WRpt | 1055 | 32286006 | Pneumonia in Q fever |
| LowerRespiratoryTract Infection-WRpt | 1055 | 33631007 | Pyopneumothorax |
| LowerRespiratoryTract Infection-WRpt | 1055 | 34020007 | Pneumonia due to Streptococcus |
| LowerRespiratoryTract Infection-WRpt | 1055 | 34286007 | Empyema with hepatopleural fistula |
| LowerRespiratoryTract Infection-WRpt | 1055 | 34290009 | Pulmonary nematodiasis |
| LowerRespiratoryTract Infection-WRpt | 1055 | 35037009 | Primary atypical interstitial pneumonia |
| LowerRespiratoryTract Infection-WRpt | 1055 | 35339003 | Primary pneumonic plague |
| LowerRespiratoryTract Infection-WRpt | 1055 | 36426008 | Subacute bronchitis |
| LowerRespiratoryTract Infection-WRpt | 1055 | 37721008 | AIDS with pneumococcal pneumonia |

| Condition Name | Condition ID | Concept ID | Primary Term |
|---|---|---|---|
| LowerRespiratoryTract Infection-WRpt | 1055 | 38699009 | Pneumonia due to Histoplasma capsulatum |
| LowerRespiratoryTract Infection-WRpt | 1055 | 38976008 | Pneumonic plague |
| LowerRespiratoryTract Infection-WRpt | 1055 | 39172002 | Pneumonia due to Proteus mirabilis |
| LowerRespiratoryTract Infection-WRpt | 1055 | 40600002 | Pneumococcal bronchitis |
| LowerRespiratoryTract Infection-WRpt | 1055 | 41207000 | Adenoviral pneumonia |
| LowerRespiratoryTract Infection-WRpt | 1055 | 41269000 | Influenzal bronchopneumonia |
| LowerRespiratoryTract Infection-WRpt | 1055 | 41381004 | Pneumonia due to Pseudomonas |
| LowerRespiratoryTract Infection-WRpt | 1055 | 42002000 | Avian infectious bronchitis |
| LowerRespiratoryTract Infection-WRpt | 1055 | 45263007 | Pulmonary sporotrichosis |
| LowerRespiratoryTract Infection-WRpt | 1055 | 45312009 | Pneumonia in typhoid fever |
| LowerRespiratoryTract Infection-WRpt | 1055 | 45556008 | Pulmonary tularaemia |
| LowerRespiratoryTract Infection-WRpt | 1055 | 46207001 | Pneumonitis due to acquired toxoplasmosis |
| LowerRespiratoryTract Infection-WRpt | 1055 | 46970008 | Mycoplasma pneumonia |
| LowerRespiratoryTract Infection-WRpt | 1055 | 47082005 | Congenital rubella pneumonitis |
| LowerRespiratoryTract Infection-WRpt | 1055 | 48722001 | Progressive pneumonia of sheep |
| LowerRespiratoryTract Infection-WRpt | 1055 | 50417007 | Lower respiratory tract infection |
| LowerRespiratoryTract Infection-WRpt | 1055 | 50648007 | Tropical eosinophilia |
| LowerRespiratoryTract Infection-WRpt | 1055 | 50694005 | Empyema with mediastinal fistula |
| LowerRespiratoryTract Infection-WRpt | 1055 | 50997000 | Pulmonary dirofilariasis |
| LowerRespiratoryTract Infection-WRpt | 1055 | 51530003 | Pneumonia due to Escherichia coli |
| LowerRespiratoryTract Infection-WRpt | 1055 | 52409006 | Bronchiolitis exudativa |
| LowerRespiratoryTract Infection-WRpt | 1055 | 53084003 | Bacterial pneumonia |
| LowerRespiratoryTract Infection-WRpt | 1055 | 56507008 | Tea-tasters' disease |

| Condition Name | Condition ID | Concept ID | Primary Term |
|---|---|---|---|
| LowerRespiratoryTract Infection-WRpt | 1055 | 57086000 | Enzootic pneumonia of calves |
| LowerRespiratoryTract Infection-WRpt | 1055 | 57089007 | Respiratory syncytial virus bronchiolitis |
| LowerRespiratoryTract Infection-WRpt | 1055 | 57541005 | Pulmonary pneumocystosis |
| LowerRespiratoryTract Infection-WRpt | 1055 | 57716000 | Brooder pneumonia |
| LowerRespiratoryTract Infection-WRpt | 1055 | 58524006 | Acute pulmonary histoplasmosis |
| LowerRespiratoryTract Infection-WRpt | 1055 | 58554001 | Empyema of pleura |
| LowerRespiratoryTract Infection-WRpt | 1055 | 58890000 | Adenoviral bronchopneumonia |
| LowerRespiratoryTract Infection-WRpt | 1055 | 59475000 | Pneumonia in pertussis |
| LowerRespiratoryTract Infection-WRpt | 1055 | 60363000 | Pneumonia |
| LowerRespiratoryTract Infection-WRpt | 1055 | 60485005 | Pleurobronchopneumonia |
| LowerRespiratoryTract Infection-WRpt | 1055 | 60696005 | AIDS with bacterial pneumonia |
| LowerRespiratoryTract Infection-WRpt | 1055 | 60837001 | Contagious bovine pleuropneumonia |
| LowerRespiratoryTract Infection-WRpt | 1055 | 60916008 | Cardiopulmonary schistosomiasis |
| LowerRespiratoryTract Infection-WRpt | 1055 | 61532009 | Enzootic pneumonia of sheep |
| LowerRespiratoryTract Infection-WRpt | 1055 | 61884008 | Achromobacter pneumonia |
| LowerRespiratoryTract Infection-WRpt | 1055 | 63741006 | Fungal infection of lung |
| LowerRespiratoryTract Infection-WRpt | 1055 | 64479007 | Pneumonia due to Klebsiella pneumoniae |
| LowerRespiratoryTract Infection-WRpt | 1055 | 64667001 | Interstitial pneumonia |
| LowerRespiratoryTract Infection-WRpt | 1055 | 64703005 | Terminal bronchopneumonia |
| LowerRespiratoryTract Infection-WRpt | 1055 | 64880000 | Parainfluenza virus bronchopneumonia |
| LowerRespiratoryTract Infection-WRpt | 1055 | 64912000 | Enzootic mycoplasmal pneumonia of swine |
| LowerRespiratoryTract Infection-WRpt | 1055 | 64917006 | Parainfluenza virus pneumonia |
| LowerRespiratoryTract Infection-WRpt | 1055 | 65095005 | Amoebic lung abscess |

| Condition Name | Condition ID | Concept ID | Primary Term |
|---|---|---|---|
| LowerRespiratoryTract Infection-WRpt | 1055 | 65102004 | Adiaspiromycosis due to Emmonsia parva |
| LowerRespiratoryTract Infection-WRpt | 1055 | 65878001 | Septic bronchitis |
| LowerRespiratoryTract Infection-WRpt | 1055 | 66429007 | Unresolved lobar pneumonia |
| LowerRespiratoryTract Infection-WRpt | 1055 | 67525007 | Secondary pneumonic plague |
| LowerRespiratoryTract Infection-WRpt | 1055 | 70036007 | Haemophilus influenzae pneumonia |
| LowerRespiratoryTract Infection-WRpt | 1055 | 71186008 | Croup |
| LowerRespiratoryTract Infection-WRpt | 1055 | 71255007 | Adenoviral laryngotracheobronchitis |
| LowerRespiratoryTract Infection-WRpt | 1055 | 71435009 | Chronic obstructive bronchitis |
| LowerRespiratoryTract Infection-WRpt | 1055 | 71926009 | Infective pneumonia acquired prenatally |
| LowerRespiratoryTract Infection-WRpt | 1055 | 73198007 | Bacterial pleurisy |
| LowerRespiratoryTract Infection-WRpt | 1055 | 73414003 | Haemophilus influenzae laryngotracheobronchitis |
| LowerRespiratoryTract Infection-WRpt | 1055 | 73452002 | Abscess of lung |
| LowerRespiratoryTract Infection-WRpt | 1055 | 73995006 | Pulmonary paracoccidioidomycosis |
| LowerRespiratoryTract Infection-WRpt | 1055 | 74387008 | Tuberculosis of hilar lymph nodes |
| LowerRespiratoryTract Infection-WRpt | 1055 | 74417001 | Mucopurulent chronic bronchitis |
| LowerRespiratoryTract Infection-WRpt | 1055 | 75388006 | Echinococcus granulosus infection of lung |
| LowerRespiratoryTract Infection-WRpt | 1055 | 75570004 | Viral pneumonia |
| LowerRespiratoryTract Infection-WRpt | 1055 | 75642009 | Bovine pneumonic pasteurellosis |
| LowerRespiratoryTract Infection-WRpt | 1055 | 76090006 | Pittsburgh pneumonia |
| LowerRespiratoryTract Infection-WRpt | 1055 | 76630008 | Fibrinopurulent pleurisy |
| LowerRespiratoryTract Infection-WRpt | 1055 | 78887005 | Seropurulent pleurisy |
| LowerRespiratoryTract Infection-WRpt | 1055 | 78895009 | Congenital pneumonia |
| LowerRespiratoryTract Infection-WRpt | 1055 | 79479005 | Respiratory syncytial virus bronchitis |

| Condition Name | Condition ID | Concept ID | Primary Term |
|---|---|---|---|
| LowerRespiratoryTract Infection-WRpt | 1055 | 80003002 | Tuberculous pneumonia |
| LowerRespiratoryTract Infection-WRpt | 1055 | 80010008 | Isolated bronchial tuberculosis |
| LowerRespiratoryTract Infection-WRpt | 1055 | 80257001 | Acute bronchitis with bronchospasm |
| LowerRespiratoryTract Infection-WRpt | 1055 | 80602006 | Nodular tuberculosis of lung |
| LowerRespiratoryTract Infection-WRpt | 1055 | 81164001 | Ornithosis with pneumonia |
| LowerRespiratoryTract Infection-WRpt | 1055 | 81554001 | Tuberculosis of lung with involvement of bronchus |
| LowerRespiratoryTract Infection-WRpt | 1055 | 81638006 | Contagious caprine pleuropneumonia |
| LowerRespiratoryTract Infection-WRpt | 1055 | 82670009 | Whooping cough due to organism other than Bordetella pertussis |
| LowerRespiratoryTract Infection-WRpt | 1055 | 84353005 | Pulmonary disease due to Mycobacteria |
| LowerRespiratoryTract Infection-WRpt | 1055 | 84659003 | Feline pneumonitis |
| LowerRespiratoryTract Infection-WRpt | 1055 | 84753008 | Pneumonia in systemic mycosis |
| LowerRespiratoryTract Infection-WRpt | 1055 | 85420008 | Streptococcal pleurisy |
| LowerRespiratoryTract Infection-WRpt | 1055 | 85426002 | Bacterial pleurisy with effusion |
| LowerRespiratoryTract Infection-WRpt | 1055 | 85469005 | Hypostatic pneumonia |
| LowerRespiratoryTract Infection-WRpt | 1055 | 85915003 | Laryngotracheobronchitis |
| LowerRespiratoryTract Infection-WRpt | 1055 | 86853002 | Feline infectious peritonitis AND pleuritis |
| LowerRespiratoryTract Infection-WRpt | 1055 | 87695000 | Necrotising bronchiolitis |
| LowerRespiratoryTract Infection-WRpt | 1055 | 88036000 | Primary pulmonary coccidioidomycosis |
| LowerRespiratoryTract Infection-WRpt | 1055 | 88693009 | AIDS with candidiasis of lung |
| LowerRespiratoryTract Infection-WRpt | 1055 | 89087009 | Porcine contagious pleuropneumonia |
| LowerRespiratoryTract Infection-WRpt | 1055 | 90117007 | Tuberculous fibrosis of lung |
| LowerRespiratoryTract Infection-WRpt | 1055 | 111849006 | Adenoviral bronchitis |
| LowerRespiratoryTract Infection-WRpt | 1055 | 111900000 | Pneumonia in aspergillosis |

| Condition Name | Condition ID | Concept ID | Primary Term |
|---|---|---|---|
| LowerRespiratoryTract Infection-WRpt | 1055 | 120639003 | Hantavirus pulmonary syndrome |
| LowerRespiratoryTract Infection-WRpt | 1055 | 123587001 | Acute bronchopneumonia |
| LowerRespiratoryTract Infection-WRpt | 1055 | 123588006 | Confluent bronchopneumonia with abscess formation |
| LowerRespiratoryTract Infection-WRpt | 1055 | 123589003 | Necrotising bronchopneumonia |
| LowerRespiratoryTract Infection-WRpt | 1055 | 123620007 | Hantanvirus pulmonary syndrome |
| LowerRespiratoryTract Infection-WRpt | 1055 | 128601007 | Infectious disease of lung |
| LowerRespiratoryTract Infection-WRpt | 1055 | 128940004 | Parasitic infection of lung |
| LowerRespiratoryTract Infection-WRpt | 1055 | 154283005 | Pulmonary tuberculosis |
| LowerRespiratoryTract Infection-WRpt | 1055 | 155510007 | Acute laryngotracheobronchitis |
| LowerRespiratoryTract Infection-WRpt | 1055 | 155548002 | Pneumonia and influenza &/or pneumonia |
| LowerRespiratoryTract Infection-WRpt | 1055 | 155552002 | Pneumonia NOS |
| LowerRespiratoryTract Infection-WRpt | 1055 | 155558003 | Pneumonia NOS |
| LowerRespiratoryTract Infection-WRpt | 1055 | 155570004 | Acute exacerbation of chronic obstructive airways disease |
| LowerRespiratoryTract Infection-WRpt | 1055 | 155571000 | Acute exacerbation of chronic obstructive airways disease |
| LowerRespiratoryTract Infection-WRpt | 1055 | 171699006 | Tuberculous pleuritis |
| LowerRespiratoryTract Infection-WRpt | 1055 | 186172004 | Tuberculous pleurisy in primary progressive tuberculosis |
| LowerRespiratoryTract Infection-WRpt | 1055 | 186175002 | Infiltrative lung tuberculosis |
| LowerRespiratoryTract Infection-WRpt | 1055 | 186177005 | Tuberculosis of lung with cavitation |
| LowerRespiratoryTract Infection-WRpt | 1055 | 186178000 | Tuberculosis of bronchus |
| LowerRespiratoryTract Infection-WRpt | 1055 | 186179008 | Other specified pulmonary tuberculosis |
| LowerRespiratoryTract Infection-WRpt | 1055 | 186180006 | Pulmonary tuberculosis NOS |
| LowerRespiratoryTract Infection-WRpt | 1055 | 186181005 | Other respiratory tuberculosis |
| LowerRespiratoryTract Infection-WRpt | 1055 | 186182003 | Tuberculosis of pleura |

| Condition Name | Condition ID | Concept ID | Primary Term |
|---|---|---|---|
| LowerRespiratoryTract Infection-WRpt | 1055 | 186186000 | Tuberculous pleurisy NOS |
| LowerRespiratoryTract Infection-WRpt | 1055 | 186188004 | Isolated tracheal or bronchial tuberculosis |
| LowerRespiratoryTract Infection-WRpt | 1055 | 186191004 | Isolated tracheal or bronchial tuberculosis NOS |
| LowerRespiratoryTract Infection-WRpt | 1055 | 186193001 | Tuberculosis of lung, confirmed by sputum microscopy with or without culture |
| LowerRespiratoryTract Infection-WRpt | 1055 | 186194007 | Tuberculosis of lung, confirmed by culture only |
| LowerRespiratoryTract Infection-WRpt | 1055 | 186195008 | Tuberculosis of lung, confirmed histologically |
| LowerRespiratoryTract Infection-WRpt | 1055 | 186197000 | Tuberculosis of lung, confirmed by unspecified means |
| LowerRespiratoryTract Infection-WRpt | 1055 | 186200004 | Tuberculous pleurisy, confirmed bacteriologically and histologically |
| LowerRespiratoryTract Infection-WRpt | 1055 | 186203002 | Tuberculosis of lung, bacteriologically and histologically negative |
| LowerRespiratoryTract Infection-WRpt | 1055 | 186204008 | Tuberculosis of lung, bacteriological and histological examination not done |
| LowerRespiratoryTract Infection-WRpt | 1055 | 186207001 | Other specified respiratory tuberculosis |
| LowerRespiratoryTract Infection-WRpt | 1055 | 186212000 | Other specified respiratory tuberculosis NOS |
| LowerRespiratoryTract Infection-WRpt | 1055 | 186283004 | Pneumonic plague, unspecified |
| LowerRespiratoryTract Infection-WRpt | 1055 | 186342000 | Pulmonary Mycobacterium avium complex infection |
| LowerRespiratoryTract Infection-WRpt | 1055 | 186353006 | Whooping cough - other specified organism |
| LowerRespiratoryTract Infection-WRpt | 1055 | 186355004 | Other whooping cough NOS |
| LowerRespiratoryTract Infection-WRpt | 1055 | 186356003 | Whooping cough NOS |
| LowerRespiratoryTract Infection-WRpt | 1055 | 187027001 | Acute pulmonary coccidioidomycosis |
| LowerRespiratoryTract Infection-WRpt | 1055 | 187042003 | Histoplasma capsulatum with pneumonia |
| LowerRespiratoryTract Infection-WRpt | 1055 | 187052004 | Pulmonary African histoplasmosis |
| LowerRespiratoryTract Infection-WRpt | 1055 | 187054003 | Pulmonary histoplasmosis |
| LowerRespiratoryTract Infection-WRpt | 1055 | 187061004 | Histoplasmosis with pneumonia |
| LowerRespiratoryTract Infection-WRpt | 1055 | 187066009 | Primary pulmonary blastomycosis |

| Condition Name | Condition ID | Concept ID | Primary Term |
|---|---|---|---|
| LowerRespiratoryTract Infection-WRpt | 1055 | 187069002 | Acute pulmonary blastomycosis |
| LowerRespiratoryTract Infection-WRpt | 1055 | 187134002 | Lung echinococcus granulosus |
| LowerRespiratoryTract Infection-WRpt | 1055 | 187196002 | Toxoplasma pneumonitis |
| LowerRespiratoryTract Infection-WRpt | 1055 | 187324001 | [X]Whooping cough due to other Bordetella species |
| LowerRespiratoryTract Infection-WRpt | 1055 | 187325000 | [X]Whooping cough, unspecified |
| LowerRespiratoryTract Infection-WRpt | 1055 | 187499004 | [X]Pulmonary histoplasmosis capsulati, unspecified |
| LowerRespiratoryTract Infection-WRpt | 1055 | 187500008 | [X]Other pulmonary aspergillosis |
| LowerRespiratoryTract Infection-WRpt | 1055 | 195702002 | Acute laryngotracheobronchitis |
| LowerRespiratoryTract Infection-WRpt | 1055 | 195712009 | Acute bronchitis and/or bronchiolitis |
| LowerRespiratoryTract Infection-WRpt | 1055 | 195714005 | Acute fibrinous bronchitis |
| LowerRespiratoryTract Infection-WRpt | 1055 | 195715006 | Acute membranous bronchitis |
| LowerRespiratoryTract Infection-WRpt | 1055 | 195717003 | Acute purulent bronchitis |
| LowerRespiratoryTract Infection-WRpt | 1055 | 195719000 | Acute pneumococcal bronchitis |
| LowerRespiratoryTract Infection-WRpt | 1055 | 195720006 | Acute streptococcal bronchitis |
| LowerRespiratoryTract Infection-WRpt | 1055 | 195721005 | Acute bronchitis caused by Haemophilus influenzae |
| LowerRespiratoryTract Infection-WRpt | 1055 | 195722003 | Acute Moraxella catarrhalis bronchitis |
| LowerRespiratoryTract Infection-WRpt | 1055 | 195725001 | Acute coxsackievirus bronchitis |
| LowerRespiratoryTract Infection-WRpt | 1055 | 195726000 | Acute parainfluenza virus bronchitis |
| LowerRespiratoryTract Infection-WRpt | 1055 | 195727009 | Acute respiratory syncytial virus bronchitis |
| LowerRespiratoryTract Infection-WRpt | 1055 | 195728004 | Acute bronchitis due to rhinovirus |
| LowerRespiratoryTract Infection-WRpt | 1055 | 195729007 | Acute echovirus bronchitis |
| LowerRespiratoryTract Infection-WRpt | 1055 | 195730002 | Subacute bronchitis unspecified |
| LowerRespiratoryTract Infection-WRpt | 1055 | 195731003 | Acute viral bronchitis unspecified |

| Condition Name | Condition ID | Concept ID | Primary Term |
|---|---|---|---|
| LowerRespiratoryTract Infection-WRpt | 1055 | 195732005 | Acute bacterial bronchitis unspecified |
| LowerRespiratoryTract Infection-WRpt | 1055 | 195733000 | Acute bronchitis NOS |
| LowerRespiratoryTract Infection-WRpt | 1055 | 195737004 | Acute exudative bronchiolitis |
| LowerRespiratoryTract Infection-WRpt | 1055 | 195739001 | Acute bronchiolitis due to respiratory syncytial virus |
| LowerRespiratoryTract Infection-WRpt | 1055 | 195740004 | Acute bronchiolitis due to other specified organisms |
| LowerRespiratoryTract Infection-WRpt | 1055 | 195741000 | Acute bronchiolitis NOS |
| LowerRespiratoryTract Infection-WRpt | 1055 | 195742007 | Acute lower respiratory tract infection |
| LowerRespiratoryTract Infection-WRpt | 1055 | 195743002 | Acute bronchitis or bronchiolitis NOS |
| LowerRespiratoryTract Infection-WRpt | 1055 | 195747001 | Chest cold |
| LowerRespiratoryTract Infection-WRpt | 1055 | 195881003 | Pneumonia due to respiratory syncytial virus |
| LowerRespiratoryTract Infection-WRpt | 1055 | 195882005 | Viral pneumonia NEC |
| LowerRespiratoryTract Infection-WRpt | 1055 | 195883000 | Viral pneumonia NOS |
| LowerRespiratoryTract Infection-WRpt | 1055 | 195885007 | Other bacterial pneumonia |
| LowerRespiratoryTract Infection-WRpt | 1055 | 195886008 | Group B streptococcal pneumonia |
| LowerRespiratoryTract Infection-WRpt | 1055 | 195887004 | Pneumonia due to other specified bacteria |
| LowerRespiratoryTract Infection-WRpt | 1055 | 195888009 | Proteus pneumonia |
| LowerRespiratoryTract Infection-WRpt | 1055 | 195889001 | Legionella pneumonia |
| LowerRespiratoryTract Infection-WRpt | 1055 | 195891009 | Pneumonia due to bacteria NOS |
| LowerRespiratoryTract Infection-WRpt | 1055 | 195892002 | Bacterial pneumonia NOS |
| LowerRespiratoryTract Infection-WRpt | 1055 | 195893007 | Pneumonia due to other specified organisms |
| LowerRespiratoryTract Infection-WRpt | 1055 | 195896004 | Pneumonia due to pleuropneumonia-like organism |
| LowerRespiratoryTract Infection-WRpt | 1055 | 195898003 | Pneumonia due to specified organism NOS |
| LowerRespiratoryTract Infection-WRpt | 1055 | 195900001 | Pneumonia due to measles |

| Condition Name | Condition ID | Concept ID | Primary Term |
|---|---|---|---|
| LowerRespiratoryTract Infection-WRpt | 1055 | 195902009 | Anthrax pneumonia |
| LowerRespiratoryTract Infection-WRpt | 1055 | 195903004 | Pneumonia with other systemic mycoses |
| LowerRespiratoryTract Infection-WRpt | 1055 | 195904005 | Pneumonia with coccidioidomycosis |
| LowerRespiratoryTract Infection-WRpt | 1055 | 195905006 | Pneumonia with histoplasmosis |
| LowerRespiratoryTract Infection-WRpt | 1055 | 195906007 | Pneumonia with systemic mycosis NOS |
| LowerRespiratoryTract Infection-WRpt | 1055 | 195907003 | Pneumonia with other infectious diseases EC |
| LowerRespiratoryTract Infection-WRpt | 1055 | 195908008 | Actinomycotic pneumonia |
| LowerRespiratoryTract Infection-WRpt | 1055 | 195909000 | Nocardial pneumonia |
| LowerRespiratoryTract Infection-WRpt | 1055 | 195911009 | Chickenpox pneumonia |
| LowerRespiratoryTract Infection-WRpt | 1055 | 195912002 | Pneumonia with other infectious diseases EC NOS |
| LowerRespiratoryTract Infection-WRpt | 1055 | 195913007 | Pneumonia with infectious diseases EC NOS |
| LowerRespiratoryTract Infection-WRpt | 1055 | 195915000 | Pneumonia due to unspecified organism |
| LowerRespiratoryTract Infection-WRpt | 1055 | 195916004 | Lobar pneumonia due to unspecified organism |
| LowerRespiratoryTract Infection-WRpt | 1055 | 195919006 | Postoperative pneumonia |
| LowerRespiratoryTract Infection-WRpt | 1055 | 195936003 | Bronchitis: [unspecified (& chest infection)] or [recurrent wheezy] |
| LowerRespiratoryTract Infection-WRpt | 1055 | 195938002 | Laryngotracheobronchitis |
| LowerRespiratoryTract Infection-WRpt | 1055 | 195947005 | Mucopurulent chronic bronchitis NOS |
| LowerRespiratoryTract Infection-WRpt | 1055 | 195949008 | Chronic asthmatic bronchitis |
| LowerRespiratoryTract Infection-WRpt | 1055 | 195951007 | Acute exacerbation of chronic obstructive airways disease |
| LowerRespiratoryTract Infection-WRpt | 1055 | 196001008 | Chronic obstructive pulmonary disease with acute lower respiratory infection |
| LowerRespiratoryTract Infection-WRpt | 1055 | 196002001 | Chronic obstructive pulmonary disease with acute exacerbation, unspecified |
| LowerRespiratoryTract Infection-WRpt | 1055 | 196063008 | Empyema with pleural fistula NOS |
| LowerRespiratoryTract Infection-WRpt | 1055 | 196064002 | Empyema with thoracic fistula NOS |

| Condition Name | Condition ID | Concept ID | Primary Term |
|---|---|---|---|
| LowerRespiratoryTract Infection-WRpt | 1055 | 196067009 | Loculated empyema |
| LowerRespiratoryTract Infection-WRpt | 1055 | 196070008 | Lung empyema NOS |
| LowerRespiratoryTract Infection-WRpt | 1055 | 196090004 | Bacterial pleurisy with effusion NOS |
| LowerRespiratoryTract Infection-WRpt | 1055 | 196092007 | Encysted pleurisy |
| LowerRespiratoryTract Infection-WRpt | 1055 | 196107009 | Abscess of lung and mediastinum |
| LowerRespiratoryTract Infection-WRpt | 1055 | 196108004 | Single lung abscess |
| LowerRespiratoryTract Infection-WRpt | 1055 | 196109007 | Multiple lung abscesses |
| LowerRespiratoryTract Infection-WRpt | 1055 | 196110002 | Gangrenous pneumonia |
| LowerRespiratoryTract Infection-WRpt | 1055 | 196112005 | Abscess of lung with pneumonia |
| LowerRespiratoryTract Infection-WRpt | 1055 | 196113000 | Abscess of lung NOS |
| LowerRespiratoryTract Infection-WRpt | 1055 | 196114006 | Abscess of lung and mediastinum NOS |
| LowerRespiratoryTract Infection-WRpt | 1055 | 196204006 | [X]Other viral pneumonia |
| LowerRespiratoryTract Infection-WRpt | 1055 | 196205007 | [X]Pneumonia due to other aerobic gram-negative bacteria |
| LowerRespiratoryTract Infection-WRpt | 1055 | 196206008 | [X]Other bacterial pneumonia |
| LowerRespiratoryTract Infection-WRpt | 1055 | 196207004 | [X]Pneumonia due to other specified infectious organisms |
| LowerRespiratoryTract Infection-WRpt | 1055 | 196208009 | [X]Pneumonia in bacterial diseases classified elsewhere |
| LowerRespiratoryTract Infection-WRpt | 1055 | 196209001 | [X]Pneumonia in viral diseases classified elsewhere |
| LowerRespiratoryTract Infection-WRpt | 1055 | 196210006 | [X]Pneumonia in mycoses classified elsewhere |
| LowerRespiratoryTract Infection-WRpt | 1055 | 196211005 | [X]Pneumonia in parasitic diseases classified elsewhere |
| LowerRespiratoryTract Infection-WRpt | 1055 | 196212003 | [X]Pneumonia in other diseases classified elsewhere |
| LowerRespiratoryTract Infection-WRpt | 1055 | 196213008 | [X]Other pneumonia, organism unspecified |
| LowerRespiratoryTract Infection-WRpt | 1055 | 196214002 | [X]Other acute lower respiratory infections |
| LowerRespiratoryTract Infection-WRpt | 1055 | 196215001 | [X]Acute bronchitis due to other specified organisms |

| Condition Name | Condition ID | Concept ID | Primary Term |
|---|---|---|---|
| LowerRespiratoryTract Infection-WRpt | 1055 | 196216000 | [X]Acute bronchiolitis due to other specified organisms |
| LowerRespiratoryTract Infection-WRpt | 1055 | 196248001 | [X]Suppurative and necrotic conditions of the lower respiratory tract |
| LowerRespiratoryTract Infection-WRpt | 1055 | 206283000 | Congenital staphylococcal pneumonia |
| LowerRespiratoryTract Infection-WRpt | 1055 | 206284006 | Congenital group A haemolytic streptococcal pneumonia |
| LowerRespiratoryTract Infection-WRpt | 1055 | 206285007 | Congenital group B haemolytic streptococcal pneumonia |
| LowerRespiratoryTract Infection-WRpt | 1055 | 206286008 | Congenital Escherichia coli pneumonia |
| LowerRespiratoryTract Infection-WRpt | 1055 | 206287004 | Congenital pseudomonal pneumonia |
| LowerRespiratoryTract Infection-WRpt | 1055 | 206289001 | Congenital chlamydial pneumonia |
| LowerRespiratoryTract Infection-WRpt | 1055 | 206290005 | Other specified congenital pneumonia |
| LowerRespiratoryTract Infection-WRpt | 1055 | 206291009 | Congenital pneumonia NOS |
| LowerRespiratoryTract Infection-WRpt | 1055 | 206359006 | Neonatal candidiasis of lung |
| LowerRespiratoryTract Infection-WRpt | 1055 | 206634009 | [X]Congenital pneumonia due to other bacterial agents |
| LowerRespiratoryTract Infection-WRpt | 1055 | 206635005 | [X]Congenital pneumonia due to other organisms |
| LowerRespiratoryTract Infection-WRpt | 1055 | 213225005 | Postoperative chest infection |
| LowerRespiratoryTract Infection-WRpt | 1055 | 233597004 | Chest infection - unspecified bronchitis |
| LowerRespiratoryTract Infection-WRpt | 1055 | 233598009 | Acute bacterial bronchitis |
| LowerRespiratoryTract Infection-WRpt | 1055 | 233599001 | Acute mycoplasmal bronchitis |
| LowerRespiratoryTract Infection-WRpt | 1055 | 233600003 | Acute chlamydial bronchitis |
| LowerRespiratoryTract Infection-WRpt | 1055 | 233601004 | Acute viral bronchitis |
| LowerRespiratoryTract Infection-WRpt | 1055 | 233602006 | Acute viral bronchiolitis |
| LowerRespiratoryTract Infection-WRpt | 1055 | 233603001 | Acute bronchiolitis due to adenovirus |
| LowerRespiratoryTract Infection-WRpt | 1055 | 233604007 | Pneumonia |
| LowerRespiratoryTract Infection-WRpt | 1055 | 233606009 | Atypical pneumonia |

| Condition Name | Condition ID | Concept ID | Primary Term |
|---|---|---|---|
| LowerRespiratoryTract Infection-WRpt | 1055 | 233607000 | Pneumococcal pneumonia |
| LowerRespiratoryTract Infection-WRpt | 1055 | 233608005 | Meningococcal pneumonia |
| LowerRespiratoryTract Infection-WRpt | 1055 | 233609002 | Chlamydial pneumonia |
| LowerRespiratoryTract Infection-WRpt | 1055 | 233610007 | Neonatal chlamydial pneumonia |
| LowerRespiratoryTract Infection-WRpt | 1055 | 233613009 | Fungal pneumonia |
| LowerRespiratoryTract Infection-WRpt | 1055 | 233614003 | Pulmonary mucormycosis |
| LowerRespiratoryTract Infection-WRpt | 1055 | 233615002 | Chronic pulmonary coccidioidomycosis |
| LowerRespiratoryTract Infection-WRpt | 1055 | 233616001 | Pulmonary blastomycosis |
| LowerRespiratoryTract Infection-WRpt | 1055 | 233617005 | Haemorrhagic pneumonia |
| LowerRespiratoryTract Infection-WRpt | 1055 | 233618000 | Mycobacterial pneumonia |
| LowerRespiratoryTract Infection-WRpt | 1055 | 233619008 | Neonatal pneumonia |
| LowerRespiratoryTract Infection-WRpt | 1055 | 233620002 | Pneumonia due to parasitic infestation |
| LowerRespiratoryTract Infection-WRpt | 1055 | 233621003 | Rickettsial pneumonia |
| LowerRespiratoryTract Infection-WRpt | 1055 | 233622005 | Infectious mononucleosis pneumonia |
| LowerRespiratoryTract Infection-WRpt | 1055 | 233623000 | Mononuclear interstitial pneumonia |
| LowerRespiratoryTract Infection-WRpt | 1055 | 233624006 | Herpes simplex pneumonia |
| LowerRespiratoryTract Infection-WRpt | 1055 | 233625007 | Giant cell pneumonia |
| LowerRespiratoryTract Infection-WRpt | 1055 | 233626008 | Chronic bronchial sepsis |
| LowerRespiratoryTract Infection-WRpt | 1055 | 233649005 | Tuberculous chylothorax |
| LowerRespiratoryTract Infection-WRpt | 1055 | 233671000 | Chronic obstructive pulmonary disease with acute exacerbation, unspecified |
| LowerRespiratoryTract Infection-WRpt | 1055 | 233785003 | Infectious disorder of trachea |
| LowerRespiratoryTract Infection-WRpt | 1055 | 233794009 | Tuberculous bronchopleural fistula |
| LowerRespiratoryTract Infection-WRpt | 1055 | 233795005 | Infectious disorder of bronchus |

| Condition Name | Condition ID | Concept ID | Primary Term |
|---|---|---|---|
| LowerRespiratoryTract Infection-WRpt | 1055 | 233797002 | Infected bronchogenic cyst |
| LowerRespiratoryTract Infection-WRpt | 1055 | 240387006 | Pulmonary glanders |
| LowerRespiratoryTract Infection-WRpt | 1055 | 240391001 | Pulmonary melioidosis |
| LowerRespiratoryTract Infection-WRpt | 1055 | 240422004 | Tracheobronchial diphtheria |
| LowerRespiratoryTract Infection-WRpt | 1055 | 240629003 | Malarial shock lung |
| LowerRespiratoryTract Infection-WRpt | 1055 | 240635003 | Leishmanial pneumonia |
| LowerRespiratoryTract Infection-WRpt | 1055 | 240702004 | Chronic necrotising pulmonary aspergillosis |
| LowerRespiratoryTract Infection-WRpt | 1055 | 240705002 | Candidiasis of trachea |
| LowerRespiratoryTract Infection-WRpt | 1055 | 240741002 | Acute pulmonary African histoplasmosis |
| LowerRespiratoryTract Infection-WRpt | 1055 | 240742009 | Chronic pulmonary African histoplasmosis |
| LowerRespiratoryTract Infection-WRpt | 1055 | 240747003 | Chronic pulmonary blastomycosis |
| LowerRespiratoryTract Infection-WRpt | 1055 | 266339003 | Chest infection NOS |
| LowerRespiratoryTract Infection-WRpt | 1055 | 266350000 | Pneumococcal lobar pneumonia |
| LowerRespiratoryTract Infection-WRpt | 1055 | 266351001 | Pneumonia with infectious diseases EC |
| LowerRespiratoryTract Infection-WRpt | 1055 | 266352008 | Bronchopneumonia due to unspecified organism |
| LowerRespiratoryTract Infection-WRpt | 1055 | 266391003 | Pneumonia and influenza &/or pneumonia |
| LowerRespiratoryTract Infection-WRpt | 1055 | 271503005 | Empyema with fistula |
| LowerRespiratoryTract Infection-WRpt | 1055 | 271504004 | Pleural empyema with no fistula |
| LowerRespiratoryTract Infection-WRpt | 1055 | 271506002 | Pleural empyema NOS |
| LowerRespiratoryTract Infection-WRpt | 1055 | 271567008 | Whooping cough-like syndrome |
| LowerRespiratoryTract Infection-WRpt | 1055 | 274103002 | Pneumonia NOS |
| LowerRespiratoryTract Infection-WRpt | 1055 | 275497007 | Infection of lower respiratory tract and mediastinum |
| LowerRespiratoryTract Infection-WRpt | 1055 | 275499005 | Acute wheezy bronchitis |

| Condition Name | Condition ID | Concept ID | Primary Term |
|---|---|---|---|
| LowerRespiratoryTract Infection-WRpt | 1055 | 276692000 | Congenital viral pneumonia |
| LowerRespiratoryTract Infection-WRpt | 1055 | 276693005 | Congenital bacterial pneumonia |
| LowerRespiratoryTract Infection-WRpt | 1055 | 276694004 | Acquired neonatal pneumonia |
| LowerRespiratoryTract Infection-WRpt | 1055 | 277869007 | Non-tuberculous mycobacterial pneumonia |
| LowerRespiratoryTract Infection-WRpt | 1055 | 278484009 | Tropical pulmonary eosinophilia |
| LowerRespiratoryTract Infection-WRpt | 1055 | 278516003 | Lobar pneumonia |
| LowerRespiratoryTract Infection-WRpt | 1055 | 285381006 | Acute infective exacerbation of chronic obstructive airways disease |
| LowerRespiratoryTract Infection-WRpt | 1055 | 300999006 | Basal pneumonia |
| LowerRespiratoryTract Infection-WRpt | 1055 | 301000005 | Left lower zone pneumonia |
| LowerRespiratoryTract Infection-WRpt | 1055 | 301001009 | Right lower zone pneumonia |
| LowerRespiratoryTract Infection-WRpt | 1055 | 301002002 | Left upper zone pneumonia |
| LowerRespiratoryTract Infection-WRpt | 1055 | 301003007 | Right middle zone pneumonia |
| LowerRespiratoryTract Infection-WRpt | 1055 | 301004001 | Right upper zone pneumonia |
| LowerRespiratoryTract Infection-WRpt | 1055 | 307763005 | Basal pneumonia due to unspecified organism |
| LowerRespiratoryTract Infection-WRpt | 1055 | 308130008 | Recurrent chest infection |
| LowerRespiratoryTract Infection-WRpt | 1055 | 308906005 | Secondary bacterial pneumonia |
| LowerRespiratoryTract Infection-WRpt | 1055 | 312119006 | Bacterial lower respiratory infection |
| LowerRespiratoryTract Infection-WRpt | 1055 | 312134000 | Viral lower respiratory infection |
| LowerRespiratoryTract Infection-WRpt | 1055 | 312342009 | Infective pneumonia |
| LowerRespiratoryTract Infection-WRpt | 1055 | 312371005 | Acute infective bronchitis |
| LowerRespiratoryTract Infection-WRpt | 1055 | 312403005 | Legionnaire's disease |
| LowerRespiratoryTract Infection-WRpt | 1055 | 313433007 | Tuberculous cavity of lung |
| LowerRespiratoryTract Infection-WRpt | 1055 | 314042000 | Infective pleurisy |

| Condition Name | Condition ID | Concept ID | Primary Term |
|---|---|---|---|
| LowerRespiratoryTract Infection-WRpt | 1055 | 314043005 | Viral pleurisy |
| LowerRespiratoryTract Infection-WRpt | 1055 | 314978007 | Postoperative pneumonia |
| LowerRespiratoryTract Infection-WRpt | 1055 | 373435003 | Battey disease |
| LowerRespiratoryTract Infection-WRpt | 1055 | 385093006 | Community acquired pneumonia |
| LowerRespiratoryTract Infection-WRpt | 1055 | 385479009 | Follicular bronchiolitis |
| LowerRespiratoryTract Infection-WRpt | 1055 | 389075004 | Tuberculous pneumonia of humans |
| LowerRespiratoryTract Infection-WRpt | 1055 | 389077007 | Tuberculous pneumonia of animals |
| LowerRespiratoryTract Infection-WRpt | 1055 | 396284006 | Lobular pneumonia |
| LowerRespiratoryTract Infection-WRpt | 1055 | 396285007 | Bronchopneumonia |
| LowerRespiratoryTract Infection-WRpt | 1055 | 396286008 | Bilateral bronchopneumonia |
| LowerRespiratoryTract Infection-WRpt | 1055 | 397190009 | Respiratory tuberculosis |
| LowerRespiratoryTract Infection-WRpt | 1055 | 398447004 | Severe acute respiratory syndrome |
| LowerRespiratoryTract Infection-WRpt | 1055 | 407671000 | Bilateral pneumonia |
| LowerRespiratoryTract Infection-WRpt | 1055 | 408679000 | Healthcare associated pneumonia |
| LowerRespiratoryTract Infection-WRpt | 1055 | 408680002 | Healthcare associated bacterial pneumonia |
| LowerRespiratoryTract Infection-WRpt | 1055 | 408681003 | Healthcare associated legionnaire's disease |
| LowerRespiratoryTract Infection-WRpt | 1055 | 408682005 | Healthcare associated pertussis |
| LowerRespiratoryTract Infection-WRpt | 1055 | 408683000 | Healthcare associated pulmonary aspergillosis |
| LowerRespiratoryTract Infection-WRpt | 1055 | 408688009 | Healthcare associated severe acute respiratory syndrome |
| LowerRespiratoryTract Infection-WRpt | 1055 | 409664000 | Pneumonia due to anaerobic bacteria |
| LowerRespiratoryTract Infection-WRpt | 1055 | 409665004 | Pneumonia due to aerobic bacteria |
| LowerRespiratoryTract Infection-WRpt | 1055 | 415125002 | Pneumocystosis pneumonia |
| LowerRespiratoryTract Infection-WRpt | 1055 | 417018008 | Pulmonary coccidioidomycosis |

| Condition Name | Condition ID | Concept ID | Primary Term |
|---|---|---|---|
| LowerRespiratoryTract Infection-WRpt | 1055 | 417688002 | Chronic progressive coccidioidal pneumonia |
| LowerRespiratoryTract Infection-WRpt | 1055 | 418122003 | Bronchomoniliasis |
| LowerRespiratoryTract Infection-WRpt | 1055 | 419502003 | Chest infection |
| LowerRespiratoryTract Infection-WRpt | 1055 | 420245002 | Pneumonia due to Mannheimia haemolytica |
| LowerRespiratoryTract Infection-WRpt | 1055 | 420544002 | Bacterial pneumonia with AIDS (acquired immunodeficiency syndrome) |
| LowerRespiratoryTract Infection-WRpt | 1055 | 420787001 | Pneumococcal pneumonia with AIDS (acquired immunodeficiency syndrome) |
| LowerRespiratoryTract Infection-WRpt | 1055 | 421047005 | Candidiasis of lung with AIDS (acquired immunodeficiency syndrome) |
| LowerRespiratoryTract Infection-WRpt | 1055 | 421217001 | Enzootic pneumonia of sheep |
| LowerRespiratoryTract Infection-WRpt | 1055 | 421508002 | Viral pneumonia with AIDS (acquired immunodeficiency syndrome) |
| LowerRespiratoryTract Infection-WRpt | 1055 | 421724004 | Bovine respiratory disease complex |
| LowerRespiratoryTract Infection-WRpt | 1055 | 425464007 | Hospital acquired pneumonia |
| LowerRespiratoryTract Infection-WRpt | 1055 | 425996009 | Bilateral basal pneumonia |
| LowerRespiratoryTract Infection-WRpt | 1055 | 426696003 | Lingular pneumonia |
| LowerRespiratoryTract Infection-WRpt | 1055 | 428697002 | Inactive tuberculosis of lung |
| LowerRespiratoryTract Infection-WRpt | 1055 | 430395005 | Pneumonia due to Gram negative bacteria |
| LowerRespiratoryTract Infection-WRpt | 1055 | 430476004 | Diffuse panbronchiolitis |
| LowerRespiratoryTract Infection-WRpt | 1055 | 438764004 | Postoperative aspiration pneumonia |
| LowerRespiratoryTract Infection-WRpt | 1055 | 441590008 | Pneumonia due to Severe acute respiratory syndrome coronavirus |
| LowerRespiratoryTract Infection-WRpt | 1055 | 441658007 | Pneumonia due to Staphylococcus aureus |
| LowerRespiratoryTract Infection-WRpt | 1055 | 441942006 | Pneumonia due to infection by Streptococcus pyogenes |
| LowerRespiratoryTract Infection-WRpt | 1055 | 442094008 | Pneumonia due to Histoplasma |
| LowerRespiratoryTract Infection-WRpt | 1055 | 443378001 | Lady Windermere syndrome |
| LowerRespiratoryTract Infection-WRpt | 1055 | 445058002 | Aspergillus bronchitis |

| Condition Name | Condition ID | Concept ID | Primary Term |
|---|---|---|---|
| LowerRespiratoryTract Infection-WRpt | 1055 | 445096001 | Pneumonia due to Human metapneumovirus |
| LowerRespiratoryTract Infection-WRpt | 1055 | 445102008 | Bronchiolitis due to Human metapneumovirus |
| LowerRespiratoryTract Infection-WRpt | 1055 | 446543007 | Tuberculous abscess of lung |
| LowerRespiratoryTract Infection-WRpt | 1055 | 446946005 | Reinfection pulmonary tuberculosis |
| LowerRespiratoryTract Infection-WRpt | 1055 | 446986002 | Tuberculous pleural effusion |
| LowerRespiratoryTract Infection-WRpt | 1055 | 447006007 | Relapse pulmonary tuberculosis |
| LowerRespiratoryTract Infection-WRpt | 1055 | 448719004 | Postoperative lower respiratory tract infection |
| LowerRespiratoryTract Infection-WRpt | 1055 | 448739000 | Recurrent lower respiratory tract infection |
| LowerRespiratoryTract Infection-WRpt | 1055 | 700273003 | Isolated tracheobronchial tuberculosis |
| LowerRespiratoryTract Infection-WRpt | 1055 | 704345008 | Chronic interstitial pneumonia |
| LowerRespiratoryTract Infection-WRpt | 1055 | 707503004 | Pneumonia due to Schistosoma mansoni |
| LowerRespiratoryTract Infection-WRpt | 1055 | 707507003 | Pneumonia due to Schistosoma japonicum |
| LowerRespiratoryTract Infection-WRpt | 1055 | 707508008 | Pneumonia due to Schistosoma haematobium |
| LowerRespiratoryTract Infection-WRpt | 1055 | 708025003 | Pyopneumothorax following infection by Coccidioides |
| LowerRespiratoryTract Infection-WRpt | 1055 | 713084008 | Pneumonia caused by Human coronavirus |
| LowerRespiratoryTract Infection-WRpt | 1055 | 713525001 | Recurrent bacterial pneumonia |
| LowerRespiratoryTract Infection-WRpt | 1055 | 713526000 | Recurrent bacterial pneumonia co-occurrent with human immunodeficiency virus infection |
| LowerRespiratoryTract Infection-WRpt | 1055 | 713544008 | Bacterial pneumonia co-occurrent with human immunodeficiency virus infection |
| LowerRespiratoryTract Infection-WRpt | 1055 | 714203003 | Acute bronchitis co-occurrent with bronchiectasis |
| LowerRespiratoryTract Infection-WRpt | 1055 | 715882005 | Severe acute respiratory syndrome of upper respiratory tract |
| LowerRespiratoryTract Infection-WRpt | 1055 | 721804002 | Infection of lung caused by Pneumocystis |
| LowerRespiratoryTract Infection-WRpt | 1055 | 724498004 | Pneumonia caused by Chlamydia pneumoniae |
| LowerRespiratoryTract Infection-WRpt | 1055 | 733051000 | Pneumonia caused by Gram positive bacteria |

| Condition Name | Condition ID | Concept ID | Primary Term |
|---|---|---|---|
| LowerRespiratoryTract Infection-WRpt | 1055 | 733171006 | Chronic pulmonary aspergillosis |
| LowerRespiratoryTract Infection-WRpt | 1055 | 733497009 | Chronic suppuration of bronchus |
| LowerRespiratoryTract Infection-WRpt | 1055 | 735464006 | Acute noninfective bronchitis |
| LowerRespiratoryTract Infection-WRpt | 1055 | 735465007 | Protracted bronchitis caused by bacterium |
| LowerRespiratoryTract Infection-WRpt | 1055 | 735532001 | Infection of lung caused by Echinococcus |
| LowerRespiratoryTract Infection-WRpt | 1055 | 737180005 | Chronic bronchiolitis |
| LowerRespiratoryTract Infection-WRpt | 1055 | 763888005 | Necrotising pneumonia caused by Panton-Valentine leukocidin producing Staphylococcus aureus |
| LowerRespiratoryTract Infection-WRpt | 1055 | 770674007 | Ghon complex |
| LowerRespiratoryTract Infection-WRpt | 1055 | 772839003 | Pneumonia caused by Influenza A virus |
| LowerRespiratoryTract Infection-WRpt | 1055 | 782761005 | Subacute invasive pulmonary aspergillosis |
| LowerRespiratoryTract Infection-WRpt | 1055 | 785745000 | Acute bronchitis co-occurrent with wheeze |
| LowerRespiratoryTract Infection-WRpt | 1055 | 788997004 | Obstructing Aspergillus tracheobronchitis |
| LowerRespiratoryTract Infection-WRpt | 1055 | 870573008 | Interstitial pneumonia with autoimmune features |
| LowerRespiratoryTract Infection-WRpt | 1055 | 1010615002 | Late syphilis of lung |
| LowerRespiratoryTract Infection-WRpt | 1055 | 1010620002 | Infection of lung caused by Mycobacterium malmoense |
| LowerRespiratoryTract Infection-WRpt | 1055 | 1010622005 | Infection of lung caused by Mycobacterium xenopi |
| LowerRespiratoryTract Infection-WRpt | 1055 | 1010634002 | Pneumonia caused by Acinetobacter |
| LowerRespiratoryTract Infection-WRpt | 1055 | 1010662009 | Infection of lung caused by Mycobacterium kansasii |
| LowerRespiratoryTract Infection-WRpt | 1055 | 1149093006 | Pneumonia caused by vancomycin resistant Enterococcus |
| LowerRespiratoryTract Infection-WRpt | 1055 | 1163147006 | Chronic fibrosing pulmonary aspergillosis |
| LowerRespiratoryTract Infection-WRpt | 1055 | 1163150009 | Chronic cavitary pulmonary aspergillosis |
| LowerRespiratoryTract Infection-WRpt | 1055 | 1163489008 | Human metapneumovirus bronchitis |
| LowerRespiratoryTract Infection-WRpt | 1055 | 1176988004 | Enterobacter pneumonia |

| Condition Name | Condition ID | Concept ID | Primary Term |
|---|---|---|---|
| LowerRespiratoryTract Infection-WRpt | 1055 | 1177000004 | Pulmonary nodule caused by Pneumocystis |
| LowerRespiratoryTract Infection-WRpt | 1055 | 1177059007 | Pneumocystis jirovecii lung cyst |
| LowerRespiratoryTract Infection-WRpt | 1055 | 1187256004 | Viral pneumonia due to Epstein-Barr virus infectious mononucleosis |
| LowerRespiratoryTract Infection-WRpt | 1055 | 1208602000 | Pneumonia caused by Pseudomonas aeruginosa |
| LowerRespiratoryTract Infection-WRpt | 1055 | 1731000119106 | Atypical mycobacterial infection of lung |
| LowerRespiratoryTract Infection-WRpt | 1055 | 12571000132104 | Pneumonitis due to Herpes zoster |
| LowerRespiratoryTract Infection-WRpt | 1055 | 16311000119108 | Pneumonia due to influenza |
| LowerRespiratoryTract Infection-WRpt | 1055 | 28791000119105 | Chronic coccidioidomycotic pneumonia |
| LowerRespiratoryTract Infection-WRpt | 1055 | 33601000087105 | Acute bronchiolitis caused by chemical fumes |
| LowerRespiratoryTract Infection-WRpt | 1055 | 106001000119101 | Chronic obstructive lung disease co-occurrent with acute bronchitis |
| LowerRespiratoryTract Infection-WRpt | 1055 | 124691000119101 | Pneumonia due to methicillin resistant Staphylococcus aureus |
| LowerRespiratoryTract Infection-WRpt | 1055 | 128711000119106 | Pneumonia due to methicillin susceptible Staphylococcus aureus |
| LowerRespiratoryTract Infection-WRpt | 1055 | 142931000119100 | Pneumonia due to H1N1 influenza |
| LowerRespiratoryTract Infection-WRpt | 1055 | 143111000119103 | Pneumonia due to avian influenza |
| LowerRespiratoryTract Infection-WRpt | 1055 | 184431000119108 | Acute pneumonia due to coccidioidomycosis |
| LowerRespiratoryTract Infection-WRpt | 1055 | 243981000119109 | Pulmonary filariasis |
| LowerRespiratoryTract Infection-WRpt | 1055 | 309421000000105 | Pulmonary blastomycosis |
| LowerRespiratoryTract Infection-WRpt | 1055 | 400631000000109 | [X]Other pneumonia, organism unspecified |
| LowerRespiratoryTract Infection-WRpt | 1055 | 402451000000104 | [X]Pulmonary histoplasmosis capsulati, unspecified |
| LowerRespiratoryTract Infection-WRpt | 1055 | 402461000000101 | [X]Other pulmonary aspergillosis |
| LowerRespiratoryTract Infection-WRpt | 1055 | 409081000000107 | [X]Congenital pneumonia due to other bacterial agents |
| LowerRespiratoryTract Infection-WRpt | 1055 | 412671000000108 | [X]Other acute lower respiratory infections |
| LowerRespiratoryTract Infection-WRpt | 1055 | 414341000000100 | [X]Pneumonia in viral diseases classified elsewhere |

| Condition Name | Condition ID | Concept ID | Primary Term |
|---|---|---|---|
| LowerRespiratoryTract Infection-WRpt | 1055 | 418961000000106 | [X]Acute bronchiolitis due to other specified organisms |
| LowerRespiratoryTract Infection-WRpt | 1055 | 429381000000103 | [X]Other viral pneumonia |
| LowerRespiratoryTract Infection-WRpt | 1055 | 431681000000108 | [X]Acute bronchitis due to other specified organisms |
| LowerRespiratoryTract Infection-WRpt | 1055 | 432151000000100 | [X]Pneumonia in bacterial diseases classified elsewhere |
| LowerRespiratoryTract Infection-WRpt | 1055 | 441141000000108 | [X]Pneumonia due to other aerobic gram-negative bacteria |
| LowerRespiratoryTract Infection-WRpt | 1055 | 442341000000102 | [X]Pneumonia in other diseases classified elsewhere |
| LowerRespiratoryTract Infection-WRpt | 1055 | 444061000000106 | [X]Whooping cough due to other Bordetella species |
| LowerRespiratoryTract Infection-WRpt | 1055 | 454431000000109 | [X]Pneumonia due to other aerobic gram-negative bacteria |
| LowerRespiratoryTract Infection-WRpt | 1055 | 456031000000108 | [X]Pneumonia in mycoses classified elsewhere |
| LowerRespiratoryTract Infection-WRpt | 1055 | 456041000000104 | [X]Whooping cough, unspecified |
| LowerRespiratoryTract Infection-WRpt | 1055 | 468671000000107 | [X]Other bacterial pneumonia |
| LowerRespiratoryTract Infection-WRpt | 1055 | 469801000000105 | [X]Pneumonia in parasitic diseases classified elsewhere |
| LowerRespiratoryTract Infection-WRpt | 1055 | 471471000000106 | [X]Pneumonia due to other specified infectious organisms |
| LowerRespiratoryTract Infection-WRpt | 1055 | 478351000000102 | [X]Congenital pneumonia due to other organisms |
| LowerRespiratoryTract Infection-WRpt | 1055 | 526041000000105 | Aspergillus bronchitis |
| LowerRespiratoryTract Infection-WRpt | 1055 | 532291000000105 | Pneumonia with infectious diseases EC |
| LowerRespiratoryTract Infection-WRpt | 1055 | 546371000000104 | Whooping cough NOS |
| LowerRespiratoryTract Infection-WRpt | 1055 | 554071000000106 | Isolated tracheal or bronchial tuberculosis NOS |
| LowerRespiratoryTract Infection-WRpt | 1055 | 554081000000108 | Tuberculosis of lung, confirmed by unspecified means |
| LowerRespiratoryTract Infection-WRpt | 1055 | 555361000000102 | Other whooping cough NOS |
| LowerRespiratoryTract Infection-WRpt | 1055 | 564541000000102 | Other specified pulmonary tuberculosis |
| LowerRespiratoryTract Infection-WRpt | 1055 | 564611000000104 | Other specified respiratory tuberculosis |
| LowerRespiratoryTract Infection-WRpt | 1055 | 564621000000105 | Other specified respiratory tuberculosis NOS |

| Condition Name | Condition ID | Concept ID | Primary Term |
|---|---|---|---|
| LowerRespiratoryTract Infection-WRpt | 1055 | 566801000000107 | Other specified congenital pneumonia |
| LowerRespiratoryTract Infection-WRpt | 1055 | 570791000000100 | Whooping cough - other specified organism |
| LowerRespiratoryTract Infection-WRpt | 1055 | 579411000000108 | Pulmonary tuberculosis NOS |
| LowerRespiratoryTract Infection-WRpt | 1055 | 579421000000102 | Other respiratory tuberculosis |
| LowerRespiratoryTract Infection-WRpt | 1055 | 579431000000100 | Tuberculous pleurisy NOS |
| LowerRespiratoryTract Infection-WRpt | 1055 | 603071000000106 | Pneumonia due to specified organism NOS |
| LowerRespiratoryTract Infection-WRpt | 1055 | 603111000000100 | Pneumonia with other infectious diseases EC |
| LowerRespiratoryTract Infection-WRpt | 1055 | 603201000000106 | Congenital pneumonia NOS |
| LowerRespiratoryTract Infection-WRpt | 1055 | 616081000000102 | Pneumonia due to other specified organisms |
| LowerRespiratoryTract Infection-WRpt | 1055 | 616091000000100 | Pneumonia with other systemic mycoses |
| LowerRespiratoryTract Infection-WRpt | 1055 | 616101000000108 | Pneumonia with systemic mycosis NOS |
| LowerRespiratoryTract Infection-WRpt | 1055 | 616111000000105 | Pneumonia with other infectious diseases EC NOS |
| LowerRespiratoryTract Infection-WRpt | 1055 | 616121000000104 | Lung empyema NOS |
| LowerRespiratoryTract Infection-WRpt | 1055 | 616131000000102 | Pneumonia with infectious diseases EC NOS |
| LowerRespiratoryTract Infection-WRpt | 1055 | 616141000000106 | Pneumonia due to unspecified organism |
| LowerRespiratoryTract Infection-WRpt | 1055 | 617941000000107 | Viral pneumonia NEC |
| LowerRespiratoryTract Infection-WRpt | 1055 | 617951000000105 | Viral pneumonia NOS |
| LowerRespiratoryTract Infection-WRpt | 1055 | 617961000000108 | Pneumonia due to other specified bacteria |
| LowerRespiratoryTract Infection-WRpt | 1055 | 617971000000101 | Pneumonia due to bacteria NOS |
| LowerRespiratoryTract Infection-WRpt | 1055 | 617981000000104 | Bacterial pneumonia NOS |
| LowerRespiratoryTract Infection-WRpt | 1055 | 621011000000106 | Acute bronchitis NOS |
| LowerRespiratoryTract Infection-WRpt | 1055 | 621021000000100 | Acute bronchiolitis due to other specified organisms |
| LowerRespiratoryTract Infection-WRpt | 1055 | 621031000000103 | Acute bronchiolitis NOS |

| Condition Name | Condition ID | Concept ID | Primary Term |
|---|---|---|---|
| LowerRespiratoryTract Infection-WRpt | 1055 | 623451000000102 | Other bacterial pneumonia |
| LowerRespiratoryTract Infection-WRpt | 1055 | 627061000000102 | Empyema with pleural fistula NOS |
| LowerRespiratoryTract Infection-WRpt | 1055 | 627071000000109 | Empyema with thoracic fistula NOS |
| LowerRespiratoryTract Infection-WRpt | 1055 | 627101000000100 | Bacterial pleurisy with effusion NOS |
| LowerRespiratoryTract Infection-WRpt | 1055 | 644261000000103 | Chest infection - unspecified bronchitis |
| LowerRespiratoryTract Infection-WRpt | 1055 | 644271000000105 | Chronic obstructive pulmonary disease with acute exacerbation, unspecified |
| LowerRespiratoryTract Infection-WRpt | 1055 | 649141000000105 | Mucopurulent chronic bronchitis NOS |
| LowerRespiratoryTract Infection-WRpt | 1055 | 651931000000103 | Subacute bronchitis unspecified |
| LowerRespiratoryTract Infection-WRpt | 1055 | 651941000000107 | Acute viral bronchitis unspecified |
| LowerRespiratoryTract Infection-WRpt | 1055 | 651951000000105 | Acute bacterial bronchitis unspecified |
| LowerRespiratoryTract Infection-WRpt | 1055 | 652331000000107 | Basal pneumonia due to unspecified organism |
| LowerRespiratoryTract Infection-WRpt | 1055 | 654461000000102 | Pneumonic plague, unspecified |
| LowerRespiratoryTract Infection-WRpt | 1055 | 656891000000102 | Lobar pneumonia due to unspecified organism |
| LowerRespiratoryTract Infection-WRpt | 1055 | 677801000000109 | Pneumonia NOS |
| LowerRespiratoryTract Infection-WRpt | 1055 | 686531000000105 | Abscess of lung NOS |
| LowerRespiratoryTract Infection-WRpt | 1055 | 686541000000101 | Abscess of lung and mediastinum NOS |
| LowerRespiratoryTract Infection-WRpt | 1055 | 689401000000108 | Bronchopneumonia due to unspecified organism |
| LowerRespiratoryTract Infection-WRpt | 1055 | 701361000000105 | Chest infection NOS |
| LowerRespiratoryTract Infection-WRpt | 1055 | 701461000000102 | Pleural empyema NOS |
| LowerRespiratoryTract Infection-WRpt | 1055 | 705871000000109 | Acute bronchitis or bronchiolitis NOS |
| LowerRespiratoryTract Infection-WRpt | 1055 | 706961000000100 | Aspergillus bronchitis |
| LowerRespiratoryTract Infection-WRpt | 1055 | 707151000000103 | Aspergilloma |
| LowerRespiratoryTract Infection-WRpt | 1055 | 810711000000102 | Acute bronchiolitis due to human metapneumovirus |

| Condition Name | Condition ID | Concept ID | Primary Term |
|---|---|---|---|
| LowerRespiratoryTract Infection-WRpt | 1055 | 810721000000108 | Pneumonia due to Human metapneumovirus |
| LowerRespiratoryTract Infection-WRpt | 1055 | 812311000000102 | Hantavirus pulmonary syndrome |
| LowerRespiratoryTract Infection-WRpt | 1055 | 823141000000101 | Community acquired pneumonia |
| LowerRespiratoryTract Infection-WRpt | 1055 | 823151000000103 | Hospital acquired pneumonia |
| LowerRespiratoryTract Infection-WRpt | 1055 | 831801000000108 | Acute bronchiolitis due to human metapneumovirus |
| LowerRespiratoryTract Infection-WRpt | 1055 | 831811000000105 | Pneumonia due to human metapneumovirus |
| LowerRespiratoryTract Infection-WRpt | 1055 | 832441000000100 | Hantavirus pulmonary syndrome |
| LowerRespiratoryTract Infection-WRpt | 1055 | 834851000000106 | Community acquired pneumonia |
| LowerRespiratoryTract Infection-WRpt | 1055 | 834861000000109 | Hospital acquired pneumonia |
| LowerRespiratoryTract Infection-WRpt | 1055 | 1033131000000107 | Pneumonia due to Gram negative bacteria |
| LowerRespiratoryTract Infection-WRpt | 1055 | 1033141000000103 | Pneumonia due to Gram negative bacteria |
| LowerRespiratoryTract Infection-WRpt | 1055 | 1082721000119101 | Pneumonia due to Ascaris |
| LowerRespiratoryTract Infection-WRpt | 1055 | 1087061000119106 | Gonococcal pneumonia |
| LowerRespiratoryTract Infection-WRpt | 1055 | 1092361000119109 | Rubella pneumonia |
| LowerRespiratoryTract Infection-WRpt | 1055 | 1092951000119106 | Pneumonia due to Bordetella parapertussis |
| LowerRespiratoryTract Infection-WRpt | 1055 | 1240541000000107 | Upper respiratory tract infection caused by SARS-CoV-2 (severe acute respiratory syndrome coronavirus 2) |
| LowerRespiratoryTract Infection-WRpt | 1055 | 1240551000000105 | Pneumonia caused by SARS-CoV-2 (severe acute respiratory syndrome coronavirus 2) |
| LowerRespiratoryTract Infection-WRpt | 1055 | 10624991000119103 | Bronchopneumonia due to Achromobacter |
| LowerRespiratoryTract Infection-WRpt | 1055 | 10625031000119102 | Bronchopneumonia due to anaerobic bacteria |
| LowerRespiratoryTract Infection-WRpt | 1055 | 10625071000119104 | Bronchopneumonia due to bacteria |
| LowerRespiratoryTract Infection-WRpt | 1055 | 10625111000119106 | Bronchopneumonia due to Escherichia coli |
| LowerRespiratoryTract Infection-WRpt | 1055 | 10625151000119107 | Bronchopneumonia due to Group A Streptococcus |
| LowerRespiratoryTract Infection-WRpt | 1055 | 10625191000119102 | Bronchopneumonia due to Group B Streptococcus |

| Condition Name | Condition ID | Concept ID | Primary Term |
|---|---|---|---|
| LowerRespiratoryTract Infection-WRpt | 1055 | 10625231000119106 | Bronchopneumonia due to Haemophilus influenzae |
| LowerRespiratoryTract Infection-WRpt | 1055 | 10625271000119109 | Bronchopneumonia due to Human metapneumovirus |
| LowerRespiratoryTract Infection-WRpt | 1055 | 10625311000119109 | Bronchopneumonia due to Klebsiella pneumoniae |
| LowerRespiratoryTract Infection-WRpt | 1055 | 10625351000119105 | Bronchopneumonia due to methicillin resistant Staphylococcus aureus |
| LowerRespiratoryTract Infection-WRpt | 1055 | 10625391000119100 | Bronchopneumonia due to methicillin susceptible Staphylococcus aureus |
| LowerRespiratoryTract Infection-WRpt | 1055 | 10625431000119105 | Bronchopneumonia due to Mycoplasma pneumoniae |
| LowerRespiratoryTract Infection-WRpt | 1055 | 10625471000119108 | Bronchopneumonia due to Proteus mirabilis |
| LowerRespiratoryTract Infection-WRpt | 1055 | 10625511000119104 | Bronchopneumonia due to Pseudomonas |
| LowerRespiratoryTract Infection-WRpt | 1055 | 10625551000119103 | Bronchopneumonia due to respiratory syncytial virus |
| LowerRespiratoryTract Infection-WRpt | 1055 | 10625591000119108 | Bronchopneumonia due to Staphylococcus |
| LowerRespiratoryTract Infection-WRpt | 1055 | 10625631000119108 | Bronchopneumonia due to Staphylococcus aureus |
| LowerRespiratoryTract Infection-WRpt | 1055 | 10625671000119106 | Bronchopneumonia due to Streptococcus |
| LowerRespiratoryTract Infection-WRpt | 1055 | 10625711000119105 | Bronchopneumonia due to Streptococcus pneumoniae |
| LowerRespiratoryTract Infection-WRpt | 1055 | 10625751000119106 | Bronchopneumonia due to virus |
| LowerRespiratoryTract Infection-WRpt | 1055 | 10629151000119105 | Acute bronchitis caused by coxsackievirus |
| LowerRespiratoryTract Infection-WRpt | 1055 | 10629191000119100 | Bronchiolitis caused by influenza virus |
| LowerRespiratoryTract Infection-WRpt | 1055 | 138389411000119105 | Acute bronchitis caused by SARS-CoV-2 (severe acute respiratory syndrome coronavirus 2) |
| LowerRespiratoryTract Infection-WRpt | 1055 | 880529761000119102 | Lower respiratory infection caused by SARS-CoV-2 (severe acute respiratory syndrome coronavirus 2) |
| LowerRespiratoryTract Infection-WRpt | 1055 | 882784691000119100 | Pneumonia caused by SARS-CoV-2 (severe acute respiratory syndrome coronavirus 2) |
| SeenInHospitalCasualty | 7183 | 4525004 | Emergency department patient visit |
| SeenInHospitalCasualty | 7183 | 11545006 | Emergency room admission, dead on arrival (DOA) |
| SeenInHospitalCasualty | 7183 | 12586001 | Physician direction of emergency medical systems |
| SeenInHospitalCasualty | 7183 | 50331008 | Emergency room admission, followed by release |

| Condition Name | Condition ID | Concept ID | Primary Term |
|---|---|---|---|
| SeenInHospitalCasualty | 7183 | 50849002 | Emergency room admission |
| SeenInHospitalCasualty | 7183 | 75004002 | Emergency room admission, died in emergency room |
| SeenInHospitalCasualty | 7183 | 183497001 | Non-urgent trauma admission |
| SeenInHospitalCasualty | 7183 | 185210004 | Seen in hospital casualty |
| SeenInHospitalCasualty | 7183 | 305226003 | Admission by Accident and Emergency doctor |
| SeenInHospitalCasualty | 7183 | 305451000 | Under care of Accident and Emergency doctor |
| SeenInHospitalCasualty | 7183 | 305633005 | Seen by Accident and Emergency doctor |
| SeenInHospitalCasualty | 7183 | 305925007 | Referral by Accident and Emergency doctor |
| SeenInHospitalCasualty | 7183 | 306390007 | Discharge by Accident and Emergency doctor |
| SeenInHospitalCasualty | 7183 | 306563004 | Discharge from Accident and Emergency service |
| SeenInHospitalCasualty | 7183 | 397721007 | Referral by Accident and Emergency |
| SeenInHospitalCasualty | 7183 | 413845009 | Chronic obstructive pulmonary disease accident and emergency attendance since last visit |
| SeenInHospitalCasualty | 7183 | 417119002 | Discharged from accident and emergency |
| SeenInHospitalCasualty | 7183 | 92291000000100 | Referral by accident and emergency |
| SeenInHospitalCasualty | 7183 | 169631000000104 | Chronic obstructive pulmonary disease accident and emergency attendance since last visit |
| SeenInHospitalCasualty | 7183 | 174211000000102 | Chronic obstructive pulmonary disease accident and emergency attendance since last visit |
| SeenInHospitalCasualty | 7183 | 188161000000105 | Discharged from accident and emergency |
| SeenInHospitalCasualty | 7183 | 191661000000106 | Discharged from accident and emergency |
| SeenInHospitalCasualty | 7183 | 353401000000109 | Seen in eye casualty department |
| SeenInHospitalCasualty | 7183 | 353411000000106 | Seen in eye casualty department |
| SeenInHospitalCasualty | 7183 | 353421000000100 | Seen in eye casualty department |
| SeenInHospitalCasualty | 7183 | 507291000000100 | Self-referral to accident and emergency department |
| SeenInHospitalCasualty | 7183 | 510051000000101 | Self-referral to accident and emergency department |

| Condition Name | Condition ID | Concept ID | Primary Term |
|---|---|---|---|
| SeenInHospitalCasualty | 7183 | 510061000000103 | Self-referral to accident and emergency department |
| SeenInHospitalCasualty | 7183 | 780781000000108 | Admission by accident and emergency doctor |
| SeenInHospitalCasualty | 7183 | 780831000000103 | Admission to accident and emergency department |
| SeenInHospitalCasualty | 7183 | 812481000000104 | Dead on arrival in accident and emergency department |
| SeenInHospitalCasualty | 7183 | 812491000000102 | Died in accident and emergency department |
| SeenInHospitalCasualty | 7183 | 826931000000104 | Frequent attender of accident and emergency department |
| SeenInHospitalCasualty | 7183 | 826941000000108 | Frequent attendance of accident and emergency |
| SeenInHospitalCasualty | 7183 | 831631000000103 | Referral by accident and emergency |
| SeenInHospitalCasualty | 7183 | 963261000000108 | Emergency department attendance related to personal alcohol consumption |
| SeenInHospitalCasualty | 7183 | 963271000000101 | Emergency department attendance related to personal alcohol consumption |
| SeenInHospitalCasualty | 7183 | 980491000000106 | Removed from Accident and Emergency department by force |
| SeenInHospitalCasualty | 7183 | 989501000000106 | Discharge from Accident and Emergency service with advice for follow up treatment by general practitioner |
| SeenInHospitalCasualty | 7183 | 989531000000100 | Left Accident and Emergency department having refused treatment |
| SeenInHospitalCasualty | 7183 | 1066331000000109 | Emergency department discharge to emergency department short stay ward |
| SeenInHospitalCasualty | 7183 | 1066341000000100 | Emergency department discharge to ambulatory emergency care service |
| SeenInHospitalCasualty | 7183 | 1066361000000104 | Emergency department discharge to high dependency unit |
| SeenInHospitalCasualty | 7183 | 1066371000000106 | Emergency department discharge to coronary care unit |
| SeenInHospitalCasualty | 7183 | 1066381000000108 | Emergency department discharge to special care baby unit |
| SeenInHospitalCasualty | 7183 | 1066391000000105 | Emergency department discharge to intensive care unit |
| SeenInHospitalCasualty | 7183 | 1066401000000108 | Emergency department discharge to neonatal intensive care unit |
| SeenInHospitalCasualty | 7183 | 1077011000000106 | Streamed from emergency department following initial assessment |
| SeenInHospitalCasualty | 7183 | 1077021000000100 | Streamed from emergency department to general practitioner following initial assessment |
| SeenInHospitalCasualty | 7183 | 1077031000000103 | Streamed from emergency department to urgent care service following initial assessment |

| Condition Name | Condition ID | Concept ID | Primary Term |
|---|---|---|---|
| SeenInHospitalCasualty | 7183 | 1077041000000107 | Streamed from emergency department to mental health service following initial assessment |
| SeenInHospitalCasualty | 7183 | 1077051000000105 | Streamed from emergency department to dental service following initial assessment |
| SeenInHospitalCasualty | 7183 | 1077061000000108 | Streamed from emergency department to ophthalmology service following initial assessment |
| SeenInHospitalCasualty | 7183 | 1077071000000101 | Streamed from emergency department to pharmacy service following initial assessment |
| SeenInHospitalCasualty | 7183 | 1077081000000104 | Streamed from emergency department to ambulatory emergency care service following initial assessment |
| SeenInHospitalCasualty | 7183 | 1077091000000102 | Streamed from emergency department to falls service following initial assessment |
| SeenInHospitalCasualty | 7183 | 1077101000000105 | Streamed from emergency department to frailty service following initial assessment |
| SeenInHospitalCasualty | 7183 | 1082421000000101 | Emergency hospital admission to accident and emergency service |
| SeenInHospitalCasualty | 7183 | 1324201000000109 | Streamed from emergency department to inpatient unit following initial assessment |
| SeenInHospitalCasualty | 7183 | 1656561000006119 | Seen in eye casualty |
| SeenInHospitalCasualty | 7183 | 1779101000006110 | Seen in gynaecology accident and emergency department |
| SeenInHospitalCasualty | 7183 | 1779111000006113 | Seen in general medicine accident and emergency department |
| SeenInHospitalCasualty | 7183 | 1779121000006117 | Seen in ENT accident and emergency department |
| SeenInHospitalCasualty | 7183 | 1779131000006119 | Seen in paediatric accident and emergency department |
| SeenInHospitalCasualty | 7183 | 1779141000006112 | Seen in surgical accident and emergency department |
| SeenInHospitalCasualty | 7183 | 1779151000006114 | Seen in orthopaedic accident and emergency department |
| UpperRespiratoryInfection-WRpt | 1007 | 140004 | Chronic pharyngitis |
| UpperRespiratoryInfection-WRpt | 1007 | 222008 | Acute epiglottitis with obstruction |
| UpperRespiratoryInfection-WRpt | 1007 | 297009 | Acute myringitis |
| UpperRespiratoryInfection-WRpt | 1007 | 652005 | Gangrenous tonsillitis |
| UpperRespiratoryInfection-WRpt | 1007 | 1532007 | Viral pharyngitis |
| UpperRespiratoryInfection-WRpt | 1007 | 1980003 | Seromucinous otitis media |

| Condition Name | Condition ID | Concept ID | Primary Term |
|---|---|---|---|
| UpperRespiratoryInfection-WRpt | 1007 | 2365002 | Chronic granular pharyngitis |
| UpperRespiratoryInfection-WRpt | 1007 | 3110003 | Acute otitis media |
| UpperRespiratoryInfection-WRpt | 1007 | 4225003 | Tuberculosis of nose |
| UpperRespiratoryInfection-WRpt | 1007 | 5028002 | Acute pansinusitis |
| UpperRespiratoryInfection-WRpt | 1007 | 6142004 | Influenza |
| UpperRespiratoryInfection-WRpt | 1007 | 6655004 | Acute laryngitis |
| UpperRespiratoryInfection-WRpt | 1007 | 7457009 | Chronic tracheitis |
| UpperRespiratoryInfection-WRpt | 1007 | 8304007 | Acute seromucinous otitis media |
| UpperRespiratoryInfection-WRpt | 1007 | 8326008 | Acute allergic mucoid otitis media |
| UpperRespiratoryInfection-WRpt | 1007 | 8442000 | Purulent rhinitis |
| UpperRespiratoryInfection-WRpt | 1007 | 8519009 | Acute tracheitis with obstruction |
| UpperRespiratoryInfection-WRpt | 1007 | 9312005 | Necrotic rhinitis |
| UpperRespiratoryInfection-WRpt | 1007 | 10345003 | Primary syphilis of tonsils |
| UpperRespiratoryInfection-WRpt | 1007 | 10351008 | Suppurative tonsillitis |
| UpperRespiratoryInfection-WRpt | 1007 | 10809006 | Parainfluenza virus laryngotracheitis |
| UpperRespiratoryInfection-WRpt | 1007 | 11134001 | Acute suppuration of sphenoidal sinus |
| UpperRespiratoryInfection-WRpt | 1007 | 11316005 | Granuloma of vocal cords |
| UpperRespiratoryInfection-WRpt | 1007 | 11461005 | Staphylococcal tonsillitis |
| UpperRespiratoryInfection-WRpt | 1007 | 13177009 | Cellulitis of nasopharynx |
| UpperRespiratoryInfection-WRpt | 1007 | 13266007 | Sphenoidal sinusitis |
| UpperRespiratoryInfection-WRpt | 1007 | 13420004 | Post measles otitis media |
| UpperRespiratoryInfection-WRpt | 1007 | 13617004 | Tracheobronchitis |
| UpperRespiratoryInfection-WRpt | 1007 | 13933003 | Feline viral rhinotracheitis |

| Condition Name | Condition ID | Concept ID | Primary Term |
| --- | --- | --- | --- |
| UpperRespiratoryInfection-WRpt | 1007 | 14310000 | Purulent nasal discharge |
| UpperRespiratoryInfection-WRpt | 1007 | 14465002 | Ulcerative tonsillitis |
| UpperRespiratoryInfection-WRpt | 1007 | 14948001 | Acute suppurative otitis media without spontaneous rupture of ear drum |
| UpperRespiratoryInfection-WRpt | 1007 | 14969004 | Catarrhal laryngitis |
| UpperRespiratoryInfection-WRpt | 1007 | 15033003 | Peritonsillar abscess |
| UpperRespiratoryInfection-WRpt | 1007 | 15682004 | Anterior nasal diphtheria |
| UpperRespiratoryInfection-WRpt | 1007 | 15805002 | Acute sinusitis |
| UpperRespiratoryInfection-WRpt | 1007 | 16036000 | Acute empyema of frontal sinus |
| UpperRespiratoryInfection-WRpt | 1007 | 16664009 | Malignant otitis media |
| UpperRespiratoryInfection-WRpt | 1007 | 17357005 | Acute suppuration of frontal sinus |
| UpperRespiratoryInfection-WRpt | 1007 | 17741008 | Acute tonsillitis |
| UpperRespiratoryInfection-WRpt | 1007 | 17866004 | Acute allergic sanguinous otitis media |
| UpperRespiratoryInfection-WRpt | 1007 | 18643000 | Ethmoidal sinusitis |
| UpperRespiratoryInfection-WRpt | 1007 | 19021002 | Haemophilus influenzae otitis media |
| UpperRespiratoryInfection-WRpt | 1007 | 19399000 | Acute exudative otitis media |
| UpperRespiratoryInfection-WRpt | 1007 | 20906004 | Mycosis leptothrica |
| UpperRespiratoryInfection-WRpt | 1007 | 21060003 | Acute bacterial epiglottitis |
| UpperRespiratoryInfection-WRpt | 1007 | 23166004 | Cellulitis of pharynx |
| UpperRespiratoryInfection-WRpt | 1007 | 23884004 | Acute suppuration of maxillary sinus |
| UpperRespiratoryInfection-WRpt | 1007 | 24078009 | Gangosa of yaws |
| UpperRespiratoryInfection-WRpt | 1007 | 24347001 | Cellulitis of vocal cords |
| UpperRespiratoryInfection-WRpt | 1007 | 24662006 | Influenza due to Influenza B virus |
| UpperRespiratoryInfection-WRpt | 1007 | 25764005 | Acute abscess of frontal sinus |

| Condition Name | Condition ID | Concept ID | Primary Term |
|---|---|---|---|
| UpperRespiratoryInfection-WRpt | 1007 | 25831001 | Tuberculosis of nasal septum |
| UpperRespiratoryInfection-WRpt | 1007 | 26650005 | Acute tracheitis |
| UpperRespiratoryInfection-WRpt | 1007 | 26808007 | Polypoid sinus degeneration |
| UpperRespiratoryInfection-WRpt | 1007 | 27278006 | Acute empyema of sphenoidal sinus |
| UpperRespiratoryInfection-WRpt | 1007 | 27836007 | Pertussis |
| UpperRespiratoryInfection-WRpt | 1007 | 27878001 | Follicular tonsillitis |
| UpperRespiratoryInfection-WRpt | 1007 | 28709001 | Cellulitis of larynx |
| UpperRespiratoryInfection-WRpt | 1007 | 29608009 | Acute epiglottitis |
| UpperRespiratoryInfection-WRpt | 1007 | 29951006 | Chronic laryngitis |
| UpperRespiratoryInfection-WRpt | 1007 | 30239003 | Acute abscess of sphenoidal sinus |
| UpperRespiratoryInfection-WRpt | 1007 | 31309002 | Respiratory syncytial virus pharyngitis |
| UpperRespiratoryInfection-WRpt | 1007 | 32179007 | Furuncle of nasal septum |
| UpperRespiratoryInfection-WRpt | 1007 | 32904004 | Pneumococcal laryngitis |
| UpperRespiratoryInfection-WRpt | 1007 | 33261009 | Abscess of tonsil |
| UpperRespiratoryInfection-WRpt | 1007 | 33924006 | Abscess of larynx |
| UpperRespiratoryInfection-WRpt | 1007 | 34790005 | Chronic tubotympanic disease with anterior perforation of ear drum |
| UpperRespiratoryInfection-WRpt | 1007 | 35168006 | Acute empyema of ethmoidal sinus |
| UpperRespiratoryInfection-WRpt | 1007 | 35183001 | Acute transudative otitis media |
| UpperRespiratoryInfection-WRpt | 1007 | 35301006 | Acute tracheobronchitis |
| UpperRespiratoryInfection-WRpt | 1007 | 35377009 | Parainfluenza virus laryngotracheobronchitis |
| UpperRespiratoryInfection-WRpt | 1007 | 35923002 | Chronic maxillary sinusitis |
| UpperRespiratoryInfection-WRpt | 1007 | 36971009 | Sinusitis |
| UpperRespiratoryInfection-WRpt | 1007 | 37426002 | Ulcerative laryngitis |

| Condition Name | Condition ID | Concept ID | Primary Term |
|---|---|---|---|
| UpperRespiratoryInfection-WRpt | 1007 | 37948003 | Acute laryngotracheitis without obstruction |
| UpperRespiratoryInfection-WRpt | 1007 | 38394007 | Chronic purulent otitis media |
| UpperRespiratoryInfection-WRpt | 1007 | 38961000 | Chronic sphenoidal sinusitis |
| UpperRespiratoryInfection-WRpt | 1007 | 39271004 | Ulcerative pharyngitis |
| UpperRespiratoryInfection-WRpt | 1007 | 39288006 | Purulent otitis media |
| UpperRespiratoryInfection-WRpt | 1007 | 40055000 | Chronic sinusitis |
| UpperRespiratoryInfection-WRpt | 1007 | 40766000 | Enteroviral lymphonodular pharyngitis |
| UpperRespiratoryInfection-WRpt | 1007 | 41048006 | Haemophilus influenzae laryngitis |
| UpperRespiratoryInfection-WRpt | 1007 | 41269000 | Influenzal bronchopneumonia |
| UpperRespiratoryInfection-WRpt | 1007 | 41582007 | Streptococcal tonsillitis |
| UpperRespiratoryInfection-WRpt | 1007 | 41954005 | Chronic atticoantral suppurative otitis media |
| UpperRespiratoryInfection-WRpt | 1007 | 42402006 | Kartagener syndrome |
| UpperRespiratoryInfection-WRpt | 1007 | 42964004 | Influenza with pneumonia |
| UpperRespiratoryInfection-WRpt | 1007 | 43692000 | Influenzal acute upper respiratory infection |
| UpperRespiratoryInfection-WRpt | 1007 | 43878008 | Streptococcal sore throat |
| UpperRespiratoryInfection-WRpt | 1007 | 45629003 | Abscess of nasopharynx |
| UpperRespiratoryInfection-WRpt | 1007 | 45913009 | Laryngitis |
| UpperRespiratoryInfection-WRpt | 1007 | 46171006 | Influenza due to Influenza virus, type A, porcine |
| UpperRespiratoryInfection-WRpt | 1007 | 47841006 | Chronic nasopharyngitis |
| UpperRespiratoryInfection-WRpt | 1007 | 49908003 | Acute epiglottitis without obstruction |
| UpperRespiratoryInfection-WRpt | 1007 | 50211006 | Catarrhal tracheitis |
| UpperRespiratoryInfection-WRpt | 1007 | 50215002 | Laryngeal diphtheria |
| UpperRespiratoryInfection-WRpt | 1007 | 51152000 | Abscess of vocal cords |

| Condition Name | Condition ID | Concept ID | Primary Term |
|---|---|---|---|
| UpperRespiratoryInfection-WRpt | 1007 | 51209006 | Viral tonsillitis |
| UpperRespiratoryInfection-WRpt | 1007 | 51476001 | Nasopharyngitis |
| UpperRespiratoryInfection-WRpt | 1007 | 51960003 | Secondary syphilis of pharynx |
| UpperRespiratoryInfection-WRpt | 1007 | 52353000 | Acute mucoid otitis media |
| UpperRespiratoryInfection-WRpt | 1007 | 52571006 | Chronic tracheobronchitis |
| UpperRespiratoryInfection-WRpt | 1007 | 52721006 | Tuberculosis of nasal sinus |
| UpperRespiratoryInfection-WRpt | 1007 | 54150009 | Upper respiratory infection |
| UpperRespiratoryInfection-WRpt | 1007 | 54383008 | Inclusion body rhinitis of swine |
| UpperRespiratoryInfection-WRpt | 1007 | 54398005 | Acute upper respiratory infection |
| UpperRespiratoryInfection-WRpt | 1007 | 55130001 | Laryngotracheitis |
| UpperRespiratoryInfection-WRpt | 1007 | 55355000 | Acute laryngopharyngitis |
| UpperRespiratoryInfection-WRpt | 1007 | 55419007 | Tuberculosis of glottis |
| UpperRespiratoryInfection-WRpt | 1007 | 55604004 | Avian influenza |
| UpperRespiratoryInfection-WRpt | 1007 | 56498009 | Tuberculosis of nasopharynx |
| UpperRespiratoryInfection-WRpt | 1007 | 57713008 | Chorditis |
| UpperRespiratoryInfection-WRpt | 1007 | 58031004 | Suppurative pharyngitis |
| UpperRespiratoryInfection-WRpt | 1007 | 58194007 | Chronic seromucinous otitis media |
| UpperRespiratoryInfection-WRpt | 1007 | 58576005 | Haemophilus influenzae epiglottitis |
| UpperRespiratoryInfection-WRpt | 1007 | 58763001 | Acute empyema of nasal sinus |
| UpperRespiratoryInfection-WRpt | 1007 | 59221008 | Parainfluenza virus rhinopharyngitis |
| UpperRespiratoryInfection-WRpt | 1007 | 59275002 | Acute allergic serous otitis media |
| UpperRespiratoryInfection-WRpt | 1007 | 59707005 | Abscess of pharynx |
| UpperRespiratoryInfection-WRpt | 1007 | 59967003 | Acute laryngotracheitis with obstruction |

| Condition Name | Condition ID | Concept ID | Primary Term |
|---|---|---|---|
| UpperRespiratoryInfection-WRpt | 1007 | 60130002 | Chronic frontal sinusitis |
| UpperRespiratoryInfection-WRpt | 1007 | 61437000 | Furuncle of nose |
| UpperRespiratoryInfection-WRpt | 1007 | 61700007 | Influenza with non-respiratory manifestation |
| UpperRespiratoryInfection-WRpt | 1007 | 61711004 | Acute abscess of ethmoidal sinus |
| UpperRespiratoryInfection-WRpt | 1007 | 62877002 | Infectious canine tracheobronchitis |
| UpperRespiratoryInfection-WRpt | 1007 | 62994001 | Tracheitis |
| UpperRespiratoryInfection-WRpt | 1007 | 63039003 | Influenza with respiratory manifestation other than pneumonia |
| UpperRespiratoryInfection-WRpt | 1007 | 63140003 | Acute suppuration of ethmoidal sinus |
| UpperRespiratoryInfection-WRpt | 1007 | 63866002 | Atrophic pharyngitis |
| UpperRespiratoryInfection-WRpt | 1007 | 64369009 | Acute tracheitis without obstruction |
| UpperRespiratoryInfection-WRpt | 1007 | 64375000 | Acute laryngotracheitis |
| UpperRespiratoryInfection-WRpt | 1007 | 64531003 | Nasal discharge |
| UpperRespiratoryInfection-WRpt | 1007 | 64611009 | Catarrhal nasal discharge |
| UpperRespiratoryInfection-WRpt | 1007 | 65363002 | Otitis media |
| UpperRespiratoryInfection-WRpt | 1007 | 66011008 | Viral tracheitis |
| UpperRespiratoryInfection-WRpt | 1007 | 67832005 | Acute ethmoidal sinusitis |
| UpperRespiratoryInfection-WRpt | 1007 | 68272006 | Acute maxillary sinusitis |
| UpperRespiratoryInfection-WRpt | 1007 | 68686002 | Infectious bovine rhinotracheitis |
| UpperRespiratoryInfection-WRpt | 1007 | 70020005 | Adenoiditis |
| UpperRespiratoryInfection-WRpt | 1007 | 70341005 | Tuberculous laryngitis |
| UpperRespiratoryInfection-WRpt | 1007 | 70385007 | Adenoviral pharyngoconjunctivitis |
| UpperRespiratoryInfection-WRpt | 1007 | 70976000 | Viral epiglottitis |
| UpperRespiratoryInfection-WRpt | 1007 | 71186008 | Croup |

| Condition Name | Condition ID | Concept ID | Primary Term |
|---|---|---|---|
| UpperRespiratoryInfection-WRpt | 1007 | 71255007 | Adenoviral laryngotracheobronchitis |
| UpperRespiratoryInfection-WRpt | 1007 | 72189003 | Haemorrhagic nasal discharge |
| UpperRespiratoryInfection-WRpt | 1007 | 72204002 | Respiratory syncytial virus laryngotracheobronchitis |
| UpperRespiratoryInfection-WRpt | 1007 | 72211003 | Laryngeal granuloma |
| UpperRespiratoryInfection-WRpt | 1007 | 72409005 | Rhinoscleroma |
| UpperRespiratoryInfection-WRpt | 1007 | 72430001 | Gangrenous pharyngitis |
| UpperRespiratoryInfection-WRpt | 1007 | 73237007 | Chronic ethmoidal sinusitis |
| UpperRespiratoryInfection-WRpt | 1007 | 73414003 | Haemophilus influenzae laryngotracheobronchitis |
| UpperRespiratoryInfection-WRpt | 1007 | 74372003 | Gonorrhoea of pharynx |
| UpperRespiratoryInfection-WRpt | 1007 | 74644004 | Influenza with encephalopathy |
| UpperRespiratoryInfection-WRpt | 1007 | 75498004 | Acute bacterial sinusitis |
| UpperRespiratoryInfection-WRpt | 1007 | 75589004 | Nasopharyngeal diphtheria |
| UpperRespiratoryInfection-WRpt | 1007 | 75803007 | Posterior rhinorrhoea |
| UpperRespiratoryInfection-WRpt | 1007 | 76651006 | Pneumococcal pharyngitis |
| UpperRespiratoryInfection-WRpt | 1007 | 76653009 | Acute empyema of maxillary sinus |
| UpperRespiratoryInfection-WRpt | 1007 | 77478005 | Acute sanguinous otitis media |
| UpperRespiratoryInfection-WRpt | 1007 | 77668003 | Isolated tracheal tuberculosis |
| UpperRespiratoryInfection-WRpt | 1007 | 77919000 | Acute sphenoidal sinusitis |
| UpperRespiratoryInfection-WRpt | 1007 | 78337007 | Acute upper respiratory infection of multiple sites |
| UpperRespiratoryInfection-WRpt | 1007 | 78430008 | Adenoviral pharyngitis |
| UpperRespiratoryInfection-WRpt | 1007 | 78431007 | Influenza due to Influenza virus, type A, human |
| UpperRespiratoryInfection-WRpt | 1007 | 78737005 | Frontal sinusitis |
| UpperRespiratoryInfection-WRpt | 1007 | 78911000 | Parainfluenza virus pharyngitis |

| Condition Name | Condition ID | Concept ID | Primary Term |
|---|---|---|---|
| UpperRespiratoryInfection-WRpt | 1007 | 80327007 | Serous otitis media |
| UpperRespiratoryInfection-WRpt | 1007 | 80384002 | Epiglottitis |
| UpperRespiratoryInfection-WRpt | 1007 | 80600003 | Acute suppuration of nasal sinus |
| UpperRespiratoryInfection-WRpt | 1007 | 81339006 | Secondary syphilis of tonsil |
| UpperRespiratoryInfection-WRpt | 1007 | 81524006 | Influenza due to Influenza C virus |
| UpperRespiratoryInfection-WRpt | 1007 | 81564005 | Chronic serous otitis media |
| UpperRespiratoryInfection-WRpt | 1007 | 82228008 | Staphylococcal pharyngitis |
| UpperRespiratoryInfection-WRpt | 1007 | 82272006 | Common cold |
| UpperRespiratoryInfection-WRpt | 1007 | 82454002 | Carbuncle of nasal septum |
| UpperRespiratoryInfection-WRpt | 1007 | 82670009 | Whooping cough due to organism other than Bordetella pertussis |
| UpperRespiratoryInfection-WRpt | 1007 | 82690000 | Suppurative laryngitis |
| UpperRespiratoryInfection-WRpt | 1007 | 83271005 | Chronic laryngotracheitis |
| UpperRespiratoryInfection-WRpt | 1007 | 83492008 | Congenital syphilitic coryza |
| UpperRespiratoryInfection-WRpt | 1007 | 84037004 | Swine influenza |
| UpperRespiratoryInfection-WRpt | 1007 | 85083002 | Streptococcal laryngitis |
| UpperRespiratoryInfection-WRpt | 1007 | 85638002 | Cerebrospinal fluid rhinorrhoea |
| UpperRespiratoryInfection-WRpt | 1007 | 85832003 | Parainfluenza virus laryngitis |
| UpperRespiratoryInfection-WRpt | 1007 | 85915003 | Laryngotracheobronchitis |
| UpperRespiratoryInfection-WRpt | 1007 | 85940005 | Tuberculous otitis media |
| UpperRespiratoryInfection-WRpt | 1007 | 86279000 | Acute suppurative otitis media with spontaneous rupture of ear drum |
| UpperRespiratoryInfection-WRpt | 1007 | 86359006 | Subacute transudative otitis media |
| UpperRespiratoryInfection-WRpt | 1007 | 86850004 | Serosanguineous chronic otitis media |
| UpperRespiratoryInfection-WRpt | 1007 | 87665008 | Chronic tubotympanic suppurative otitis media |

| Condition Name | Condition ID | Concept ID | Primary Term |
|---|---|---|---|
| UpperRespiratoryInfection-WRpt | 1007 | 88348008 | Maxillary sinusitis |
| UpperRespiratoryInfection-WRpt | 1007 | 88850006 | Chronic pansinusitis |
| UpperRespiratoryInfection-WRpt | 1007 | 89194009 | Acute abscess of maxillary sinus |
| UpperRespiratoryInfection-WRpt | 1007 | 90176007 | Tonsillitis |
| UpperRespiratoryInfection-WRpt | 1007 | 90347002 | Infectious avian laryngotracheitis |
| UpperRespiratoryInfection-WRpt | 1007 | 90979004 | Chronic tonsillitis |
| UpperRespiratoryInfection-WRpt | 1007 | 91038008 | Acute frontal sinusitis |
| UpperRespiratoryInfection-WRpt | 1007 | 95885008 | Mycoplasmal pharyngitis |
| UpperRespiratoryInfection-WRpt | 1007 | 95886009 | Mycoplasmal tracheobronchitis |
| UpperRespiratoryInfection-WRpt | 1007 | 102453009 | Peritonsillar cellulitis |
| UpperRespiratoryInfection-WRpt | 1007 | 111274000 | Acute abscess of nasal sinus |
| UpperRespiratoryInfection-WRpt | 1007 | 111275004 | Abscess of nasal septum |
| UpperRespiratoryInfection-WRpt | 1007 | 111816002 | Pneumococcal tonsillitis |
| UpperRespiratoryInfection-WRpt | 1007 | 126664009 | Exudative pharyngitis |
| UpperRespiratoryInfection-WRpt | 1007 | 126665005 | Oropharyngeal mucositis |
| UpperRespiratoryInfection-WRpt | 1007 | 139168000 | Influenza-like symptoms |
| UpperRespiratoryInfection-WRpt | 1007 | 154341002 | Postmeasles otitis media |
| UpperRespiratoryInfection-WRpt | 1007 | 155222006 | Otitis media NOS |
| UpperRespiratoryInfection-WRpt | 1007 | 155505008 | Acute laryngitis and tracheitis |
| UpperRespiratoryInfection-WRpt | 1007 | 161913008 | Influenza-like symptoms |
| UpperRespiratoryInfection-WRpt | 1007 | 164189000 | O/E - nasal discharge NOS |
| UpperRespiratoryInfection-WRpt | 1007 | 186353006 | Whooping cough - other specified organism |
| UpperRespiratoryInfection-WRpt | 1007 | 186355004 | Other whooping cough NOS |

| Condition Name | Condition ID | Concept ID | Primary Term |
|---|---|---|---|
| UpperRespiratoryInfection-WRpt | 1007 | 186356003 | Whooping cough NOS |
| UpperRespiratoryInfection-WRpt | 1007 | 186361001 | Streptococcal sore throat NOS |
| UpperRespiratoryInfection-WRpt | 1007 | 186560001 | Postmeasles otitis media |
| UpperRespiratoryInfection-WRpt | 1007 | 186659004 | Herpangina |
| UpperRespiratoryInfection-WRpt | 1007 | 186675001 | Viral pharyngoconjunctivitis |
| UpperRespiratoryInfection-WRpt | 1007 | 186963008 | Vincent's angina |
| UpperRespiratoryInfection-WRpt | 1007 | 187084006 | Tonsillar aspergillosis |
| UpperRespiratoryInfection-WRpt | 1007 | 187100003 | Rhinocerebral mucormycosis |
| UpperRespiratoryInfection-WRpt | 1007 | 187222000 | Nasopharyngeal myiasis |
| UpperRespiratoryInfection-WRpt | 1007 | 187324001 | [X]Whooping cough due to other Bordetella species |
| UpperRespiratoryInfection-WRpt | 1007 | 187325000 | [X]Whooping cough, unspecified |
| UpperRespiratoryInfection-WRpt | 1007 | 194202008 | Infective otitis externa due to erysipelas |
| UpperRespiratoryInfection-WRpt | 1007 | 194203003 | Infective otitis externa due to impetigo |
| UpperRespiratoryInfection-WRpt | 1007 | 194240006 | Acute non-suppurative serous otitis media |
| UpperRespiratoryInfection-WRpt | 1007 | 194244002 | Acute non-suppurative otitis media NOS |
| UpperRespiratoryInfection-WRpt | 1007 | 194248004 | Bilateral chronic serous otitis |
| UpperRespiratoryInfection-WRpt | 1007 | 194249007 | Unilateral chronic serous otitis |
| UpperRespiratoryInfection-WRpt | 1007 | 194250007 | Chronic serous otitis media NOS |
| UpperRespiratoryInfection-WRpt | 1007 | 194252004 | Glue ear, unspecified |
| UpperRespiratoryInfection-WRpt | 1007 | 194254003 | Chronic mucoid otitis media NOS |
| UpperRespiratoryInfection-WRpt | 1007 | 194256001 | Chronic otitis media with effusion, other |
| UpperRespiratoryInfection-WRpt | 1007 | 194260003 | Unspecified non-suppurative otitis media |
| UpperRespiratoryInfection-WRpt | 1007 | 194261004 | Allergic otitis media NOS |

| Condition Name | Condition ID | Concept ID | Primary Term |
|---|---|---|---|
| UpperRespiratoryInfection-WRpt | 1007 | 194262006 | Serous otitis media NOS |
| UpperRespiratoryInfection-WRpt | 1007 | 194263001 | Catarrhal otitis media NOS |
| UpperRespiratoryInfection-WRpt | 1007 | 194264007 | Mucoid otitis media NOS |
| UpperRespiratoryInfection-WRpt | 1007 | 194265008 | Non-suppurative otitis media NOS |
| UpperRespiratoryInfection-WRpt | 1007 | 194276007 | Chronic otitis media with effusion, unspecified |
| UpperRespiratoryInfection-WRpt | 1007 | 194281003 | Acute suppurative otitis media |
| UpperRespiratoryInfection-WRpt | 1007 | 194282005 | Acute suppurative otitis media due to another disease |
| UpperRespiratoryInfection-WRpt | 1007 | 194283000 | Acute suppurative otitis media NOS |
| UpperRespiratoryInfection-WRpt | 1007 | 194284006 | Chronic suppurative otitis media NOS |
| UpperRespiratoryInfection-WRpt | 1007 | 194286008 | Bilateral suppurative otitis media |
| UpperRespiratoryInfection-WRpt | 1007 | 194287004 | Recurrent acute otitis media |
| UpperRespiratoryInfection-WRpt | 1007 | 194288009 | Acute left otitis media |
| UpperRespiratoryInfection-WRpt | 1007 | 194289001 | Acute right otitis media |
| UpperRespiratoryInfection-WRpt | 1007 | 194290005 | Acute bilateral otitis media |
| UpperRespiratoryInfection-WRpt | 1007 | 194311006 | Acute myringitis without otitis media |
| UpperRespiratoryInfection-WRpt | 1007 | 194312004 | Unspecified acute tympanitis |
| UpperRespiratoryInfection-WRpt | 1007 | 194313009 | Acute myringitis NOS |
| UpperRespiratoryInfection-WRpt | 1007 | 194675002 | [X]Other acute non-suppurative otitis media |
| UpperRespiratoryInfection-WRpt | 1007 | 194677005 | [X]Other chronic suppurative otitis media |
| UpperRespiratoryInfection-WRpt | 1007 | 194680006 | [X]Otitis media in other diseases classified elsewhere |
| UpperRespiratoryInfection-WRpt | 1007 | 195651009 | Other acute sinusitis |
| UpperRespiratoryInfection-WRpt | 1007 | 195652002 | Other acute sinusitis NOS |
| UpperRespiratoryInfection-WRpt | 1007 | 195653007 | Acute sinusitis NOS |

| Condition Name | Condition ID | Concept ID | Primary Term |
|---|---|---|---|
| UpperRespiratoryInfection-WRpt | 1007 | 195655000 | Acute gangrenous pharyngitis |
| UpperRespiratoryInfection-WRpt | 1007 | 195656004 | Acute phlegmonous pharyngitis |
| UpperRespiratoryInfection-WRpt | 1007 | 195657008 | Acute ulcerative pharyngitis |
| UpperRespiratoryInfection-WRpt | 1007 | 195658003 | Acute bacterial pharyngitis |
| UpperRespiratoryInfection-WRpt | 1007 | 195659006 | Acute pneumococcal pharyngitis |
| UpperRespiratoryInfection-WRpt | 1007 | 195660001 | Acute staphylococcal pharyngitis |
| UpperRespiratoryInfection-WRpt | 1007 | 195661002 | Acute bacterial pharyngitis NOS |
| UpperRespiratoryInfection-WRpt | 1007 | 195662009 | Acute viral pharyngitis |
| UpperRespiratoryInfection-WRpt | 1007 | 195664005 | Acute pharyngitis NOS |
| UpperRespiratoryInfection-WRpt | 1007 | 195666007 | Acute erythematous tonsillitis |
| UpperRespiratoryInfection-WRpt | 1007 | 195667003 | Acute follicular tonsillitis |
| UpperRespiratoryInfection-WRpt | 1007 | 195668008 | Acute ulcerative tonsillitis |
| UpperRespiratoryInfection-WRpt | 1007 | 195669000 | Acute catarrhal tonsillitis |
| UpperRespiratoryInfection-WRpt | 1007 | 195670004 | Acute gangrenous tonsillitis |
| UpperRespiratoryInfection-WRpt | 1007 | 195671000 | Acute bacterial tonsillitis |
| UpperRespiratoryInfection-WRpt | 1007 | 195672007 | Acute pneumococcal tonsillitis |
| UpperRespiratoryInfection-WRpt | 1007 | 195673002 | Acute staphylococcal tonsillitis |
| UpperRespiratoryInfection-WRpt | 1007 | 195674008 | Acute bacterial tonsillitis NOS |
| UpperRespiratoryInfection-WRpt | 1007 | 195676005 | Acute viral tonsillitis |
| UpperRespiratoryInfection-WRpt | 1007 | 195677001 | Recurrent acute tonsillitis |
| UpperRespiratoryInfection-WRpt | 1007 | 195678006 | Acute tonsillitis NOS |
| UpperRespiratoryInfection-WRpt | 1007 | 195679003 | Acute laryngitis and tracheitis |
| UpperRespiratoryInfection-WRpt | 1007 | 195680000 | Acute oedematous laryngitis |

| Condition Name | Condition ID | Concept ID | Primary Term |
|---|---|---|---|
| UpperRespiratoryInfection-WRpt | 1007 | 195681001 | Acute ulcerative laryngitis |
| UpperRespiratoryInfection-WRpt | 1007 | 195682008 | Acute catarrhal laryngitis |
| UpperRespiratoryInfection-WRpt | 1007 | 195683003 | Acute phlegmonous laryngitis |
| UpperRespiratoryInfection-WRpt | 1007 | 195684009 | Acute laryngitis caused by Haemophilus influenzae |
| UpperRespiratoryInfection-WRpt | 1007 | 195685005 | Acute pneumococcal laryngitis |
| UpperRespiratoryInfection-WRpt | 1007 | 195686006 | Acute suppurative laryngitis |
| UpperRespiratoryInfection-WRpt | 1007 | 195688007 | Acute viral laryngitis unspecified |
| UpperRespiratoryInfection-WRpt | 1007 | 195689004 | Acute bacterial laryngitis unspecified |
| UpperRespiratoryInfection-WRpt | 1007 | 195690008 | Acute laryngitis NOS |
| UpperRespiratoryInfection-WRpt | 1007 | 195693005 | Acute tracheitis NOS |
| UpperRespiratoryInfection-WRpt | 1007 | 195697006 | Acute laryngotracheitis NOS |
| UpperRespiratoryInfection-WRpt | 1007 | 195701009 | Acute epiglottitis NOS |
| UpperRespiratoryInfection-WRpt | 1007 | 195703007 | Acute laryngitis and tracheitis NOS |
| UpperRespiratoryInfection-WRpt | 1007 | 195704001 | Other acute upper respiratory infections |
| UpperRespiratoryInfection-WRpt | 1007 | 195707008 | Tracheopharyngitis |
| UpperRespiratoryInfection-WRpt | 1007 | 195708003 | Recurrent upper respiratory tract infection |
| UpperRespiratoryInfection-WRpt | 1007 | 195709006 | Pharyngolaryngitis |
| UpperRespiratoryInfection-WRpt | 1007 | 195710001 | Other upper respiratory infections of multiple sites |
| UpperRespiratoryInfection-WRpt | 1007 | 195756009 | Woakes' ethmoiditis |
| UpperRespiratoryInfection-WRpt | 1007 | 195757000 | Polypoid sinus degeneration NOS |
| UpperRespiratoryInfection-WRpt | 1007 | 195770009 | Chronic infective rhinitis |
| UpperRespiratoryInfection-WRpt | 1007 | 195779005 | Pharyngitis keratosa |
| UpperRespiratoryInfection-WRpt | 1007 | 195780008 | Pharyngitis sicca |

| Condition Name | Condition ID | Concept ID | Primary Term |
|---|---|---|---|
| UpperRespiratoryInfection-WRpt | 1007 | 195782000 | Chronic follicular pharyngitis |
| UpperRespiratoryInfection-WRpt | 1007 | 195783005 | Chronic pharyngitis NOS |
| UpperRespiratoryInfection-WRpt | 1007 | 195784004 | Chronic pharyngitis and nasopharyngitis NOS |
| UpperRespiratoryInfection-WRpt | 1007 | 195788001 | Recurrent sinusitis |
| UpperRespiratoryInfection-WRpt | 1007 | 195789009 | Other chronic sinusitis |
| UpperRespiratoryInfection-WRpt | 1007 | 195790000 | Pansinusitis |
| UpperRespiratoryInfection-WRpt | 1007 | 195791001 | Other chronic sinusitis NOS |
| UpperRespiratoryInfection-WRpt | 1007 | 195792008 | Chronic sinusitis NOS |
| UpperRespiratoryInfection-WRpt | 1007 | 195798007 | Chronic adenotonsillitis |
| UpperRespiratoryInfection-WRpt | 1007 | 195803003 | Caseous tonsillitis |
| UpperRespiratoryInfection-WRpt | 1007 | 195804009 | Lingular tonsillitis |
| UpperRespiratoryInfection-WRpt | 1007 | 195810009 | Chronic laryngitis and laryngotracheitis |
| UpperRespiratoryInfection-WRpt | 1007 | 195811008 | Chronic simple laryngitis |
| UpperRespiratoryInfection-WRpt | 1007 | 195812001 | Chronic catarrhal laryngitis |
| UpperRespiratoryInfection-WRpt | 1007 | 195816003 | Chronic laryngitis NOS |
| UpperRespiratoryInfection-WRpt | 1007 | 195836002 | Pharynx or nasopharynx abscess |
| UpperRespiratoryInfection-WRpt | 1007 | 195853009 | Singers' chorditis |
| UpperRespiratoryInfection-WRpt | 1007 | 195854003 | Fibrinous chorditis |
| UpperRespiratoryInfection-WRpt | 1007 | 195855002 | Chorditis vocalis inferior |
| UpperRespiratoryInfection-WRpt | 1007 | 195878008 | Pneumonia and influenza |
| UpperRespiratoryInfection-WRpt | 1007 | 195920000 | Influenza with pneumonia, influenza virus identified |
| UpperRespiratoryInfection-WRpt | 1007 | 195921001 | Influenza with pneumonia NOS |
| UpperRespiratoryInfection-WRpt | 1007 | 195922008 | Influenza with other respiratory manifestation |

| Condition Name | Condition ID | Concept ID | Primary Term |
|---|---|---|---|
| UpperRespiratoryInfection-WRpt | 1007 | 195923003 | Influenza with laryngitis |
| UpperRespiratoryInfection-WRpt | 1007 | 195924009 | Influenza with pharyngitis |
| UpperRespiratoryInfection-WRpt | 1007 | 195925005 | Influenza with respiratory manifestations NOS |
| UpperRespiratoryInfection-WRpt | 1007 | 195927002 | Influenza with other manifestations |
| UpperRespiratoryInfection-WRpt | 1007 | 195930009 | Influenza with other manifestations NOS |
| UpperRespiratoryInfection-WRpt | 1007 | 195933006 | Other specified pneumonia or influenza |
| UpperRespiratoryInfection-WRpt | 1007 | 195934000 | Pneumonia or influenza NOS |
| UpperRespiratoryInfection-WRpt | 1007 | 195937007 | Tracheobronchitis NOS |
| UpperRespiratoryInfection-WRpt | 1007 | 196196007 | [X]Other acute sinusitis |
| UpperRespiratoryInfection-WRpt | 1007 | 196197003 | [X]Acute pharyngitis due to other specified organisms |
| UpperRespiratoryInfection-WRpt | 1007 | 196198008 | [X]Acute tonsillitis due to other specified organisms |
| UpperRespiratoryInfection-WRpt | 1007 | 196199000 | [X]Other acute upper respiratory infections of multiple sites |
| UpperRespiratoryInfection-WRpt | 1007 | 196200002 | [X]Influenza with other respiratory manifestations, influenza virus identified |
| UpperRespiratoryInfection-WRpt | 1007 | 196201003 | [X]Influenza with other manifestations, influenza virus identified |
| UpperRespiratoryInfection-WRpt | 1007 | 196202005 | [X]Influenza with other respiratory manifestations, virus not identified |
| UpperRespiratoryInfection-WRpt | 1007 | 196203000 | [X]Influenza with other manifestations, virus not identified |
| UpperRespiratoryInfection-WRpt | 1007 | 196220001 | [X]Other chronic sinusitis |
| UpperRespiratoryInfection-WRpt | 1007 | 196226007 | [X]Other abscess of pharynx |
| UpperRespiratoryInfection-WRpt | 1007 | 232251007 | Recurrent acute suppurative otitis media |
| UpperRespiratoryInfection-WRpt | 1007 | 232252000 | Recurrent acute non-suppurative otitis media |
| UpperRespiratoryInfection-WRpt | 1007 | 232311007 | Endocochlear cytomegalovirus infection |
| UpperRespiratoryInfection-WRpt | 1007 | 232363000 | Abscess of nasal cavity |
| UpperRespiratoryInfection-WRpt | 1007 | 232367004 | Nasal syphilis |

| Condition Name | Condition ID | Concept ID | Primary Term |
|---|---|---|---|
| UpperRespiratoryInfection-WRpt | 1007 | 232390009 | Suppurative sinusitis with complications |
| UpperRespiratoryInfection-WRpt | 1007 | 232391008 | Recurrent acute sinusitis |
| UpperRespiratoryInfection-WRpt | 1007 | 232393006 | Chronic anterior ethmoidal sinusitis |
| UpperRespiratoryInfection-WRpt | 1007 | 232394000 | Chronic posterior ethmoidal sinusitis |
| UpperRespiratoryInfection-WRpt | 1007 | 232395004 | Chronic panethmoidal sinusitis |
| UpperRespiratoryInfection-WRpt | 1007 | 232396003 | Chronic osteomeatal disease |
| UpperRespiratoryInfection-WRpt | 1007 | 232397007 | Chronic frontoethmoidal sinusitis |
| UpperRespiratoryInfection-WRpt | 1007 | 232399005 | Acute herpes simplex pharyngitis |
| UpperRespiratoryInfection-WRpt | 1007 | 232400003 | Acute herpes zoster pharyngitis |
| UpperRespiratoryInfection-WRpt | 1007 | 232401004 | Glandular fever pharyngitis |
| UpperRespiratoryInfection-WRpt | 1007 | 232402006 | Meningococcal pharyngitis |
| UpperRespiratoryInfection-WRpt | 1007 | 232403001 | Chlamydial pharyngitis |
| UpperRespiratoryInfection-WRpt | 1007 | 232404007 | Acute pharyngeal candidiasis |
| UpperRespiratoryInfection-WRpt | 1007 | 232405008 | Chronic ulcerative pharyngitis |
| UpperRespiratoryInfection-WRpt | 1007 | 232406009 | Chronic pharyngeal candidiasis |
| UpperRespiratoryInfection-WRpt | 1007 | 232417005 | Vincent's tonsillitis |
| UpperRespiratoryInfection-WRpt | 1007 | 232418000 | Acute infection of tonsillar remnant |
| UpperRespiratoryInfection-WRpt | 1007 | 232420002 | Chronic adenoiditis |
| UpperRespiratoryInfection-WRpt | 1007 | 232426008 | Acute simple laryngitis |
| UpperRespiratoryInfection-WRpt | 1007 | 232428009 | Acute membranous laryngitis |
| UpperRespiratoryInfection-WRpt | 1007 | 232429001 | Acute subglottic laryngitis |
| UpperRespiratoryInfection-WRpt | 1007 | 232430006 | Recurrent allergic croup |
| UpperRespiratoryInfection-WRpt | 1007 | 232432003 | Paediatric acute epiglottitis and supraglottitis |

| Condition Name | Condition ID | Concept ID | Primary Term |
|---|---|---|---|
| UpperRespiratoryInfection-WRpt | 1007 | 232433008 | Adult acute epiglottitis and supraglottitis |
| UpperRespiratoryInfection-WRpt | 1007 | 232434002 | Chronic fungal laryngitis |
| UpperRespiratoryInfection-WRpt | 1007 | 232439007 | Relapsing polychondritis of larynx |
| UpperRespiratoryInfection-WRpt | 1007 | 232460001 | Granulomatosis with polyangiitis of larynx |
| UpperRespiratoryInfection-WRpt | 1007 | 233596008 | Viral upper respiratory tract infection NOS |
| UpperRespiratoryInfection-WRpt | 1007 | 233799004 | Acute toxic tracheobronchitis |
| UpperRespiratoryInfection-WRpt | 1007 | 234528007 | Nasopharyngeal sarcoidosis |
| UpperRespiratoryInfection-WRpt | 1007 | 240444009 | Fusobacterial necrotising tonsillitis |
| UpperRespiratoryInfection-WRpt | 1007 | 240547000 | Lymphonodular coxsackie pharyngitis |
| UpperRespiratoryInfection-WRpt | 1007 | 240704003 | Pharyngeal candidiasis |
| UpperRespiratoryInfection-WRpt | 1007 | 240735009 | Classical histoplasmosis nasal ulceration |
| UpperRespiratoryInfection-WRpt | 1007 | 240913009 | Halzoun |
| UpperRespiratoryInfection-WRpt | 1007 | 249458000 | Supraglottic abscess |
| UpperRespiratoryInfection-WRpt | 1007 | 249463001 | Subglottic abscess |
| UpperRespiratoryInfection-WRpt | 1007 | 266146001 | Vincent's angina NOS |
| UpperRespiratoryInfection-WRpt | 1007 | 266337001 | Acute epiglottitis (non-streptococcal) |
| UpperRespiratoryInfection-WRpt | 1007 | 266338006 | Upper respiratory infection NOS |
| UpperRespiratoryInfection-WRpt | 1007 | 266353003 | Influenza NOS |
| UpperRespiratoryInfection-WRpt | 1007 | 267669008 | Otitis media NOS |
| UpperRespiratoryInfection-WRpt | 1007 | 267759006 | Chronic atticoantral disease |
| UpperRespiratoryInfection-WRpt | 1007 | 270490007 | Acute otitis media with effusion |
| UpperRespiratoryInfection-WRpt | 1007 | 271567008 | Whooping cough-like syndrome |
| UpperRespiratoryInfection-WRpt | 1007 | 275281000 | Catarrh |

| Condition Name | Condition ID | Concept ID | Primary Term |
|---|---|---|---|
| UpperRespiratoryInfection-WRpt | 1007 | 275376007 | Congenital syphilitic chronic coryza |
| UpperRespiratoryInfection-WRpt | 1007 | 275495004 | Acute fibrinous laryngotracheobronchitis |
| UpperRespiratoryInfection-WRpt | 1007 | 276443001 | Acute laryngitis and/or tracheitis |
| UpperRespiratoryInfection-WRpt | 1007 | 276700005 | Congenital syphilitic rhinitis |
| UpperRespiratoryInfection-WRpt | 1007 | 281794004 | Viral upper respiratory tract infection |
| UpperRespiratoryInfection-WRpt | 1007 | 301824001 | Acute viral laryngotracheitis |
| UpperRespiratoryInfection-WRpt | 1007 | 302911003 | Acute lingual tonsillitis |
| UpperRespiratoryInfection-WRpt | 1007 | 312118003 | Bacterial upper respiratory infection |
| UpperRespiratoryInfection-WRpt | 1007 | 312218008 | Infective otitis media |
| UpperRespiratoryInfection-WRpt | 1007 | 312400008 | Acute infective tracheobronchitis |
| UpperRespiratoryInfection-WRpt | 1007 | 312422001 | Infective pharyngitis |
| UpperRespiratoryInfection-WRpt | 1007 | 312423006 | Infective laryngitis |
| UpperRespiratoryInfection-WRpt | 1007 | 315642008 | Influenza-like symptoms |
| UpperRespiratoryInfection-WRpt | 1007 | 359609001 | Acute secretory otitis media |
| UpperRespiratoryInfection-WRpt | 1007 | 360595002 | Acute necrotising otitis media |
| UpperRespiratoryInfection-WRpt | 1007 | 363746003 | Acute pharyngitis |
| UpperRespiratoryInfection-WRpt | 1007 | 371103000 | Granulomatous epiglottitis |
| UpperRespiratoryInfection-WRpt | 1007 | 371127003 | Obstructive sinusitis |
| UpperRespiratoryInfection-WRpt | 1007 | 399095008 | Fusospirochaetal pharyngitis |
| UpperRespiratoryInfection-WRpt | 1007 | 405737000 | Pharyngitis |
| UpperRespiratoryInfection-WRpt | 1007 | 408669002 | Acute laryngitis with obstruction |
| UpperRespiratoryInfection-WRpt | 1007 | 408682005 | Healthcare associated pertussis |
| UpperRespiratoryInfection-WRpt | 1007 | 408687004 | Healthcare associated influenza disease |

| Condition Name | Condition ID | Concept ID | Primary Term |
|---|---|---|---|
| UpperRespiratoryInfection-WRpt | 1007 | 414304001 | Furuncle of nasal cavity |
| UpperRespiratoryInfection-WRpt | 1007 | 415724006 | Tonsillitis due to Gram negative bacteria |
| UpperRespiratoryInfection-WRpt | 1007 | 420706008 | Nasopharyngeal fascioliasis |
| UpperRespiratoryInfection-WRpt | 1007 | 424327005 | Tonsillar actinomycosis |
| UpperRespiratoryInfection-WRpt | 1007 | 427873006 | Influenza due to influenza virus type A, avian, H5N1 strain |
| UpperRespiratoryInfection-WRpt | 1007 | 427909005 | Chronic recurrent sinusitis |
| UpperRespiratoryInfection-WRpt | 1007 | 429427008 | Invasive fungal sinusitis |
| UpperRespiratoryInfection-WRpt | 1007 | 429759002 | Cerebrospinal fluid leak from nose and mouth |
| UpperRespiratoryInfection-WRpt | 1007 | 431231008 | Acute rhinosinusitis |
| UpperRespiratoryInfection-WRpt | 1007 | 432381000 | Human papilloma virus infection of vocal cord |
| UpperRespiratoryInfection-WRpt | 1007 | 441551009 | Viral laryngitis |
| UpperRespiratoryInfection-WRpt | 1007 | 442438000 | Influenza due to Influenza A virus |
| UpperRespiratoryInfection-WRpt | 1007 | 442696006 | Influenza due to Influenza A virus subtype H1N1 |
| UpperRespiratoryInfection-WRpt | 1007 | 444745000 | Infection of mucous cyst of nasal sinus |
| UpperRespiratoryInfection-WRpt | 1007 | 444814009 | Viral sinusitis |
| UpperRespiratoryInfection-WRpt | 1007 | 445130008 | Acute infective adenoiditis |
| UpperRespiratoryInfection-WRpt | 1007 | 446594000 | Infection of pharynx due to Chlamydia trachomatis |
| UpperRespiratoryInfection-WRpt | 1007 | 446753005 | Tuberculosis of oropharynx |
| UpperRespiratoryInfection-WRpt | 1007 | 450715004 | Influenza due to Influenza A virus subtype H7 |
| UpperRespiratoryInfection-WRpt | 1007 | 450716003 | Influenza due to Influenza A virus subtype H9 |
| UpperRespiratoryInfection-WRpt | 1007 | 703468005 | Bacterial tonsillitis |
| UpperRespiratoryInfection-WRpt | 1007 | 703469002 | Bacterial otitis media |
| UpperRespiratoryInfection-WRpt | 1007 | 703470001 | Bacterial sinusitis |

| Condition Name | Condition ID | Concept ID | Primary Term |
|---|---|---|---|
| UpperRespiratoryInfection-WRpt | 1007 | 707448003 | Influenza due to Influenza A virus subtype H7N9 |
| UpperRespiratoryInfection-WRpt | 1007 | 707509000 | Tracheobronchitis due to Aspergillus |
| UpperRespiratoryInfection-WRpt | 1007 | 709663002 | Supraglottitis |
| UpperRespiratoryInfection-WRpt | 1007 | 711128004 | Influenza due to influenza virus type A, avian, H3N2 strain |
| UpperRespiratoryInfection-WRpt | 1007 | 713083002 | Influenza caused by Influenza A virus subtype H5 |
| UpperRespiratoryInfection-WRpt | 1007 | 715593000 | Candidiasis of nose |
| UpperRespiratoryInfection-WRpt | 1007 | 715882005 | Severe acute respiratory syndrome of upper respiratory tract |
| UpperRespiratoryInfection-WRpt | 1007 | 716673006 | Infection of upper respiratory tract caused by fungus |
| UpperRespiratoryInfection-WRpt | 1007 | 717231003 | PFAPA syndrome |
| UpperRespiratoryInfection-WRpt | 1007 | 719522009 | Candidiasis of upper respiratory tract co-occurrent with human immunodeficiency virus infection |
| UpperRespiratoryInfection-WRpt | 1007 | 719590007 | Influenza caused by seasonal influenza virus |
| UpperRespiratoryInfection-WRpt | 1007 | 719865001 | Influenza caused by pandemic influenza virus |
| UpperRespiratoryInfection-WRpt | 1007 | 721586007 | Pharyngotonsillitis caused by Human herpes simplex virus |
| UpperRespiratoryInfection-WRpt | 1007 | 721741006 | Sinusitis caused by Streptococcus pneumoniae |
| UpperRespiratoryInfection-WRpt | 1007 | 721742004 | Otitis media caused by Streptococcus pneumoniae |
| UpperRespiratoryInfection-WRpt | 1007 | 721755003 | Sinusitis caused by Haemophilus influenzae |
| UpperRespiratoryInfection-WRpt | 1007 | 723880004 | Abscess of upper respiratory tract |
| UpperRespiratoryInfection-WRpt | 1007 | 725916003 | Acute sinusitis caused by fungus |
| UpperRespiratoryInfection-WRpt | 1007 | 725917007 | Acute sinusitis caused by virus |
| UpperRespiratoryInfection-WRpt | 1007 | 733170007 | Chronic aspergillosis of paranasal sinus |
| UpperRespiratoryInfection-WRpt | 1007 | 735463000 | Perichondritis of trachea |
| UpperRespiratoryInfection-WRpt | 1007 | 735740009 | Infection causing tracheitis in neonate |
| UpperRespiratoryInfection-WRpt | 1007 | 765152004 | Chronic serous otitis media of left ear |

| Condition Name | Condition ID | Concept ID | Primary Term |
|---|---|---|---|
| UpperRespiratoryInfection-WRpt | 1007 | 765153009 | Chronic serous otitis media of right ear |
| UpperRespiratoryInfection-WRpt | 1007 | 772810003 | Influenza caused by Influenza A virus subtype H3N2 |
| UpperRespiratoryInfection-WRpt | 1007 | 772828001 | Influenza caused by Influenza A virus subtype H5N1 |
| UpperRespiratoryInfection-WRpt | 1007 | 772839003 | Pneumonia caused by Influenza A virus |
| UpperRespiratoryInfection-WRpt | 1007 | 788964000 | Laryngeal myiasis |
| UpperRespiratoryInfection-WRpt | 1007 | 789057009 | Tuberculosis of middle ear |
| UpperRespiratoryInfection-WRpt | 1007 | 827090008 | Foul smelling discharge from nose |
| UpperRespiratoryInfection-WRpt | 1007 | 836475004 | Mucopurulent discharge from nose |
| UpperRespiratoryInfection-WRpt | 1007 | 838367000 | Allergic rhinosinusitis caused by Aspergillus |
| UpperRespiratoryInfection-WRpt | 1007 | 878818001 | Pharyngotonsillitis |
| UpperRespiratoryInfection-WRpt | 1007 | 897656009 | Rhinosinusitis |
| UpperRespiratoryInfection-WRpt | 1007 | 897657000 | Chronic rhinosinusitis |
| UpperRespiratoryInfection-WRpt | 1007 | 1010619008 | Inflammatory tracheobronchial papilloma |
| UpperRespiratoryInfection-WRpt | 1007 | 1010626008 | Recurrent peritonsillar abscess |
| UpperRespiratoryInfection-WRpt | 1007 | 1149091008 | Influenza caused by Influenza A virus subtype H2 |
| UpperRespiratoryInfection-WRpt | 1007 | 1163148001 | Chronic suppurative otitis media caused by Aspergillus |
| UpperRespiratoryInfection-WRpt | 1007 | 1163523005 | Fibrosis of upper respiratory tract due to paracoccidioidomycosis |
| UpperRespiratoryInfection-WRpt | 1007 | 1186932001 | Leakage of cerebrospinal fluid from nose following surgical procedure |
| UpperRespiratoryInfection-WRpt | 1007 | 7271000119107 | Acute bilateral otitis media with effusion |
| UpperRespiratoryInfection-WRpt | 1007 | 12181000119103 | Contact granuloma of larynx |
| UpperRespiratoryInfection-WRpt | 1007 | 16311000119108 | Pneumonia due to influenza |
| UpperRespiratoryInfection-WRpt | 1007 | 22951000119104 | Recurrent croup |
| UpperRespiratoryInfection-WRpt | 1007 | 41931000119102 | Sinusitis co-occurrent with nasal polyps |

| Condition Name | Condition ID | Concept ID | Primary Term |
|---|---|---|---|
| UpperRespiratoryInfection-WRpt | 1007 | 84261000119106 | Acute persistent otitis media |
| UpperRespiratoryInfection-WRpt | 1007 | 88171000119100 | Acute adenoiditis |
| UpperRespiratoryInfection-WRpt | 1007 | 104041000119108 | Fungal sinusitis |
| UpperRespiratoryInfection-WRpt | 1007 | 133171000119105 | Chronic pharyngolaryngitis |
| UpperRespiratoryInfection-WRpt | 1007 | 142921000119103 | Upper respiratory tract infection due to avian influenza |
| UpperRespiratoryInfection-WRpt | 1007 | 142931000119100 | Pneumonia due to H1N1 influenza |
| UpperRespiratoryInfection-WRpt | 1007 | 142941000119109 | Upper respiratory tract infection due to H1N1 influenza |
| UpperRespiratoryInfection-WRpt | 1007 | 143111000119103 | Pneumonia due to avian influenza |
| UpperRespiratoryInfection-WRpt | 1007 | 198691000000108 | Acute obstructive laryngitis |
| UpperRespiratoryInfection-WRpt | 1007 | 214081000000107 | Acute obstructive laryngitis |
| UpperRespiratoryInfection-WRpt | 1007 | 280331000000102 | Avian influenza |
| UpperRespiratoryInfection-WRpt | 1007 | 290051000119103 | Bilateral recurrent acute suppurative otitis media of middle ears |
| UpperRespiratoryInfection-WRpt | 1007 | 292631000000106 | Avian influenza |
| UpperRespiratoryInfection-WRpt | 1007 | 297831000000109 | Cerebrospinal fluid leak from nose and mouth |
| UpperRespiratoryInfection-WRpt | 1007 | 313281000000109 | Acute rhinosinusitis |
| UpperRespiratoryInfection-WRpt | 1007 | 328531000119104 | Upper respiratory tract infection due to Influenza A |
| UpperRespiratoryInfection-WRpt | 1007 | 338111000000109 | Acute rhinosinusitis |
| UpperRespiratoryInfection-WRpt | 1007 | 402101000000108 | [X]Other abscess of pharynx |
| UpperRespiratoryInfection-WRpt | 1007 | 402501000000101 | [X]Other chronic suppurative otitis media |
| UpperRespiratoryInfection-WRpt | 1007 | 411271000000104 | [X]Other acute upper respiratory infections of multiple sites |
| UpperRespiratoryInfection-WRpt | 1007 | 418181000000104 | [X]Influenza with other respiratory manifestations, influenza virus identified |
| UpperRespiratoryInfection-WRpt | 1007 | 418191000000102 | [X]Influenza with other manifestations, influenza virus identified |
| UpperRespiratoryInfection-WRpt | 1007 | 420141000000100 | [X]Otitis media in other diseases classified elsewhere |

| Condition Name | Condition ID | Concept ID | Primary Term |
|---|---|---|---|
| UpperRespiratoryInfection-WRpt | 1007 | 427821000000103 | [X]Acute pharyngitis due to other specified organisms |
| UpperRespiratoryInfection-WRpt | 1007 | 430891000000103 | [X]Influenza with other respiratory manifestations, virus not identified |
| UpperRespiratoryInfection-WRpt | 1007 | 441131000000104 | [X]Influenza with other manifestations, virus not identified |
| UpperRespiratoryInfection-WRpt | 1007 | 444061000000106 | [X]Whooping cough due to other Bordetella species |
| UpperRespiratoryInfection-WRpt | 1007 | 444121000000104 | [X]Other acute non-suppurative otitis media |
| UpperRespiratoryInfection-WRpt | 1007 | 452761000000100 | [X]Acute tonsillitis due to other specified organisms |
| UpperRespiratoryInfection-WRpt | 1007 | 454211000000100 | [X]Other acute sinusitis |
| UpperRespiratoryInfection-WRpt | 1007 | 455311000000109 | [X]Other chronic sinusitis |
| UpperRespiratoryInfection-WRpt | 1007 | 456041000000104 | [X]Whooping cough, unspecified |
| UpperRespiratoryInfection-WRpt | 1007 | 505131000000104 | Influenza due to Influenza A virus subtype H1N1 |
| UpperRespiratoryInfection-WRpt | 1007 | 510671000000104 | Influenza due to Influenza A virus subtype H1N1 |
| UpperRespiratoryInfection-WRpt | 1007 | 538321000000103 | Streptococcal sore throat NOS |
| UpperRespiratoryInfection-WRpt | 1007 | 539811000000107 | Chronic serous otitis media NOS |
| UpperRespiratoryInfection-WRpt | 1007 | 539871000000102 | Acute suppurative otitis media NOS |
| UpperRespiratoryInfection-WRpt | 1007 | 540121000000103 | Influenza with other manifestations |
| UpperRespiratoryInfection-WRpt | 1007 | 540131000000101 | Influenza with other manifestations NOS |
| UpperRespiratoryInfection-WRpt | 1007 | 540141000000105 | Other specified pneumonia or influenza |
| UpperRespiratoryInfection-WRpt | 1007 | 540151000000108 | Pneumonia or influenza NOS |
| UpperRespiratoryInfection-WRpt | 1007 | 546371000000104 | Whooping cough NOS |
| UpperRespiratoryInfection-WRpt | 1007 | 551571000000104 | Glue ear, unspecified |
| UpperRespiratoryInfection-WRpt | 1007 | 555361000000102 | Other whooping cough NOS |
| UpperRespiratoryInfection-WRpt | 1007 | 570791000000100 | Whooping cough - other specified organism |
| UpperRespiratoryInfection-WRpt | 1007 | 576581000000107 | Acute viral laryngitis unspecified |

| Condition Name | Condition ID | Concept ID | Primary Term |
|---|---|---|---|
| UpperRespiratoryInfection-WRpt | 1007 | 579671000000101 | Other chronic sinusitis |
| UpperRespiratoryInfection-WRpt | 1007 | 579681000000104 | Other chronic sinusitis NOS |
| UpperRespiratoryInfection-WRpt | 1007 | 598921000000106 | Unspecified acute tympanitis |
| UpperRespiratoryInfection-WRpt | 1007 | 598931000000108 | Acute myringitis NOS |
| UpperRespiratoryInfection-WRpt | 1007 | 600041000000109 | Catarrhal otitis media NOS |
| UpperRespiratoryInfection-WRpt | 1007 | 600051000000107 | Non-suppurative otitis media NOS |
| UpperRespiratoryInfection-WRpt | 1007 | 600981000000100 | Other acute sinusitis NOS |
| UpperRespiratoryInfection-WRpt | 1007 | 600991000000103 | Acute sinusitis NOS |
| UpperRespiratoryInfection-WRpt | 1007 | 603081000000108 | Acute bacterial laryngitis unspecified |
| UpperRespiratoryInfection-WRpt | 1007 | 603091000000105 | Acute laryngitis NOS |
| UpperRespiratoryInfection-WRpt | 1007 | 603101000000102 | Acute tracheitis NOS |
| UpperRespiratoryInfection-WRpt | 1007 | 603121000000106 | Acute epiglottitis NOS |
| UpperRespiratoryInfection-WRpt | 1007 | 603131000000108 | Acute laryngitis and tracheitis NOS |
| UpperRespiratoryInfection-WRpt | 1007 | 603141000000104 | Other acute upper respiratory infections |
| UpperRespiratoryInfection-WRpt | 1007 | 605921000000107 | Chronic laryngitis NOS |
| UpperRespiratoryInfection-WRpt | 1007 | 606871000000108 | Unspecified non-suppurative otitis media |
| UpperRespiratoryInfection-WRpt | 1007 | 606881000000105 | Allergic otitis media NOS |
| UpperRespiratoryInfection-WRpt | 1007 | 607911000000108 | Other acute sinusitis |
| UpperRespiratoryInfection-WRpt | 1007 | 607921000000102 | Acute pharyngitis NOS |
| UpperRespiratoryInfection-WRpt | 1007 | 607931000000100 | Acute laryngotracheitis NOS |
| UpperRespiratoryInfection-WRpt | 1007 | 611481000000104 | Chronic sinusitis NOS |
| UpperRespiratoryInfection-WRpt | 1007 | 616161000000107 | Influenza with pneumonia NOS |
| UpperRespiratoryInfection-WRpt | 1007 | 616171000000100 | Influenza with other respiratory manifestation |

| Condition Name | Condition ID | Concept ID | Primary Term |
|---|---|---|---|
| UpperRespiratoryInfection-WRpt | 1007 | 616181000000103 | Influenza with respiratory manifestations NOS |
| UpperRespiratoryInfection-WRpt | 1007 | 619661000000107 | Chronic otitis media with effusion, unspecified |
| UpperRespiratoryInfection-WRpt | 1007 | 621131000000104 | Acute non-suppurative otitis media NOS |
| UpperRespiratoryInfection-WRpt | 1007 | 621141000000108 | Chronic mucoid otitis media NOS |
| UpperRespiratoryInfection-WRpt | 1007 | 621151000000106 | Chronic otitis media with effusion, other |
| UpperRespiratoryInfection-WRpt | 1007 | 622321000000109 | O/E - nasal discharge NOS |
| UpperRespiratoryInfection-WRpt | 1007 | 623441000000100 | Acute bacterial pharyngitis NOS |
| UpperRespiratoryInfection-WRpt | 1007 | 623461000000104 | Acute bacterial tonsillitis NOS |
| UpperRespiratoryInfection-WRpt | 1007 | 623471000000106 | Acute tonsillitis NOS |
| UpperRespiratoryInfection-WRpt | 1007 | 623611000000103 | Polypoid sinus degeneration NOS |
| UpperRespiratoryInfection-WRpt | 1007 | 623621000000109 | Chronic pharyngitis NOS |
| UpperRespiratoryInfection-WRpt | 1007 | 623631000000106 | Chronic pharyngitis and nasopharyngitis NOS |
| UpperRespiratoryInfection-WRpt | 1007 | 643741000000108 | Serous otitis media NOS |
| UpperRespiratoryInfection-WRpt | 1007 | 643751000000106 | Mucoid otitis media NOS |
| UpperRespiratoryInfection-WRpt | 1007 | 644251000000101 | Viral upper respiratory tract infection NOS |
| UpperRespiratoryInfection-WRpt | 1007 | 645631000000105 | Other upper respiratory infections of multiple sites |
| UpperRespiratoryInfection-WRpt | 1007 | 647141000000100 | Otitis media NOS |
| UpperRespiratoryInfection-WRpt | 1007 | 649111000000109 | Tracheobronchitis NOS |
| UpperRespiratoryInfection-WRpt | 1007 | 670551000000108 | Influenza NOS |
| UpperRespiratoryInfection-WRpt | 1007 | 686051000000106 | Vincent's angina NOS |
| UpperRespiratoryInfection-WRpt | 1007 | 693581000000101 | Upper respiratory infection NOS |
| UpperRespiratoryInfection-WRpt | 1007 | 694131000000108 | Chronic suppurative otitis media NOS |
| UpperRespiratoryInfection-WRpt | 1007 | 849571000000102 | Acute bacterial laryngitis |

| Condition Name | Condition ID | Concept ID | Primary Term |
|---|---|---|---|
| UpperRespiratoryInfection-WRpt | 1007 | 856211000006111 | Post influenzal debility |
| UpperRespiratoryInfection-WRpt | 1007 | 877891000000108 | Pharyngeal lymphogranuloma venereum |
| UpperRespiratoryInfection-WRpt | 1007 | 877901000000109 | Pharyngeal lymphogranuloma venereum |
| UpperRespiratoryInfection-WRpt | 1007 | 1033051000000101 | Influenza due to zoonotic influenza virus |
| UpperRespiratoryInfection-WRpt | 1007 | 1033061000000103 | Influenza due to zoonotic influenza virus |
| UpperRespiratoryInfection-WRpt | 1007 | 1033071000000105 | Influenza due to pandemic influenza virus |
| UpperRespiratoryInfection-WRpt | 1007 | 1033081000000107 | Influenza due to pandemic influenza virus |
| UpperRespiratoryInfection-WRpt | 1007 | 1033091000000109 | Influenza due to seasonal influenza virus |
| UpperRespiratoryInfection-WRpt | 1007 | 1033101000000101 | Influenza due to seasonal influenza virus |
| UpperRespiratoryInfection-WRpt | 1007 | 1033111000000104 | Influenza with pneumonia due to seasonal influenza virus |
| UpperRespiratoryInfection-WRpt | 1007 | 1033121000000105 | Influenzal bronchopneumonia due to seasonal influenza virus |
| UpperRespiratoryInfection-WRpt | 1007 | 1050601000000101 | Influenza due to seasonal influenza virus |
| UpperRespiratoryInfection-WRpt | 1007 | 1050981000000100 | Influenza due to seasonal influenza virus |
| UpperRespiratoryInfection-WRpt | 1007 | 1082561000119104 | Recurrent acute suppurative otitis media with spontaneous rupture of ear drum |
| UpperRespiratoryInfection-WRpt | 1007 | 1082871000119108 | Bilateral acute allergic otitis media of middle ears |
| UpperRespiratoryInfection-WRpt | 1007 | 1082911000119106 | Bilateral acute eustachian salpingitis |
| UpperRespiratoryInfection-WRpt | 1007 | 1082951000119107 | Bilateral mucoid otitis media of middle ears |
| UpperRespiratoryInfection-WRpt | 1007 | 1083051000119102 | Bilateral recurrent acute serous otitis media of middle ears |
| UpperRespiratoryInfection-WRpt | 1007 | 1083061000119100 | Bilateral spontaneous rupture of tympanic membranes of ears co-occurrent and due to recurrent acute suppurative otitis media |
| UpperRespiratoryInfection-WRpt | 1007 | 1083421000119103 | Bilateral chronic serosanguineous otitis media of middle ears |
| UpperRespiratoryInfection-WRpt | 1007 | 1083431000119100 | Bilateral chronic suppurative otitis media of middle ears |
| UpperRespiratoryInfection-WRpt | 1007 | 1087981000119103 | Acute allergic otitis media of left middle ear |
| UpperRespiratoryInfection-WRpt | 1007 | 1088021000119100 | Left acute eustachian salpingitis |

| Condition Name | Condition ID | Concept ID | Primary Term |
|---|---|---|---|
| UpperRespiratoryInfection-WRpt | 1007 | 1088061000119105 | Acute mucoid otitis media of left middle ear |
| UpperRespiratoryInfection-WRpt | 1007 | 1088181000119100 | Recurrent acute suppurative otitis media of left ear with spontaneous rupture of tympanic membrane |
| UpperRespiratoryInfection-WRpt | 1007 | 1088401000119103 | Chronic atticoantral suppurative otitis media of left middle ear |
| UpperRespiratoryInfection-WRpt | 1007 | 1088551000119103 | Left chronic serosanguineous otitis media |
| UpperRespiratoryInfection-WRpt | 1007 | 1088571000119107 | Chronic suppurative otitis media of left middle ear |
| UpperRespiratoryInfection-WRpt | 1007 | 1088581000119105 | Chronic tubotympanic suppurative otitis media of left middle ear |
| UpperRespiratoryInfection-WRpt | 1007 | 1090001000119105 | Otitis media due to scarlet fever |
| UpperRespiratoryInfection-WRpt | 1007 | 1090211000119102 | Pharyngeal diphtheria |
| UpperRespiratoryInfection-WRpt | 1007 | 1090601000119102 | Acute allergic otitis media of right middle ear |
| UpperRespiratoryInfection-WRpt | 1007 | 1090641000119100 | Right acute eustachian salpingitis |
| UpperRespiratoryInfection-WRpt | 1007 | 1090681000119105 | Acute mucoid otitis media of right middle ear |
| UpperRespiratoryInfection-WRpt | 1007 | 1090791000119102 | Recurrent acute suppurative otitis media of right ear with spontaneous rupture of tympanic membrane |
| UpperRespiratoryInfection-WRpt | 1007 | 1091011000119101 | Chronic atticoantral suppurative otitis media of right middle ear |
| UpperRespiratoryInfection-WRpt | 1007 | 1091161000119104 | Right chronic serosanguineous otitis media |
| UpperRespiratoryInfection-WRpt | 1007 | 1091181000119108 | Chronic suppurative otitis media of right middle ear |
| UpperRespiratoryInfection-WRpt | 1007 | 1091191000119106 | Chronic tubotympanic suppurative otitis media of right middle ear |
| UpperRespiratoryInfection-WRpt | 1007 | 1240521000000100 | Otitis media due to disease caused by SARS-CoV-2 (severe acute respiratory syndrome coronavirus 2) |
| UpperRespiratoryInfection-WRpt | 1007 | 1240541000000107 | Upper respiratory tract infection caused by SARS-CoV-2 (severe acute respiratory syndrome coronavirus 2) |
| UpperRespiratoryInfection-WRpt | 1007 | 1787121000006116 | Community acquired pneumonia |
| UpperRespiratoryInfection-WRpt | 1007 | 1787131000006118 | Hospital acquired pneumonia |
| UpperRespiratoryInfection-WRpt | 1007 | 10624911000119107 | Otitis media due to H1N1 influenza |

| Condition Name | Condition ID | Concept ID | Primary Term |
|---|---|---|---|
| UpperRespiratoryInfection-WRpt | 1007 | 10624951000119108 | Otitis media due to influenza |
| UpperRespiratoryInfection-WRpt | 1007 | 10629191000119100 | Bronchiolitis caused by influenza virus |
| UpperRespiratoryInfection-WRpt | 1007 | 10629231000119109 | Recurrent acute streptococcal tonsillitis |
| UpperRespiratoryInfection-WRpt | 1007 | 10629551000119101 | Recurrent acute sphenoid sinusitis |
| UpperRespiratoryInfection-WRpt | 1007 | 10629591000119106 | Recurrent acute frontal sinusitis |
| UpperRespiratoryInfection-WRpt | 1007 | 10629631000119106 | Recurrent acute pansinusitis |
| UpperRespiratoryInfection-WRpt | 1007 | 10629671000119109 | Recurrent acute maxillary sinusitis |
| UpperRespiratoryInfection-WRpt | 1007 | 10629711000119108 | Recurrent acute ethmoid sinusitis |
| UpperRespiratoryInfection-WRpt | 1007 | 10674911000119108 | Otitis media due to Influenza A virus |
| UpperRespiratoryInfection-WRpt | 1007 | 10685111000119102 | Upper respiratory tract infection due to Influenza |
| UpperRespiratoryInfection-WRpt | 1007 | 12366661000119100 | Recurrent croup |

**Table S2: SNOMED CT Expression Constraint Language (ECL) codes for asthma**

| Condition ID | Condition Name | ECL Element | ConceptID |
|---|---|---|---|
| 4204 | AsthmaExacerbation | AddSupertype | 281239006 |
| 4210 | AsthmaManagementNoPlan | AddSubtype | 176711000000100 |
| 4210 | AsthmaManagementNoPlan | AddSubtype | 892301000000100 |
| 4209 | AsthmaManagementPlan | AddSubtype | 736056000 |
| 4209 | AsthmaManagementPlan | AddSubtype | 811921000000103 |
| 4209 | AsthmaManagementPlan | AddSupertype | 390872009 |
| 4208 | AsthmaReview | AddSubtype | 394700004 |
| 4208 | AsthmaReview | AddSubtype | 394720003 |
| 4208 | AsthmaReview | AddSubtype | 754061000000100 |
| 7163 | HospitalAdmission | AddSupertype | 32485007 |
| 1020 | Influenza-likeIllness | AddSubtype | 315642008 |
| 1020 | Influenza-likeIllness | AddSupertype | 6142004 |
| 1020 | Influenza-likeIllness | AddSupertype | 78046005 |
| 1020 | Influenza-likeIllness | AddSupertype | 95891005 |
| 1020 | Influenza-likeIllness | AddSupertype | 195929004 |
| 1020 | Influenza-likeIllness | AddSupertype | 309789002 |
| 1055 | LowerRespiratoryTractInfection | AddSubtype | 7063008 |
| 1055 | LowerRespiratoryTractInfection | AddSubtype | 18988001 |
| 1055 | LowerRespiratoryTractInfection | AddSubtype | 27757009 |

| Condition ID | Condition Name | ECL Element | ConceptID |
|---|---|---|---|
| 1055 | LowerRespiratoryTractInfection | AddSubtype | 29731002 |
| 1055 | LowerRespiratoryTractInfection | AddSubtype | 36426008 |
| 1055 | LowerRespiratoryTractInfection | AddSubtype | 71186008 |
| 1055 | LowerRespiratoryTractInfection | AddSubtype | 71255007 |
| 1055 | LowerRespiratoryTractInfection | AddSubtype | 73414003 |
| 1055 | LowerRespiratoryTractInfection | AddSubtype | 85469005 |
| 1055 | LowerRespiratoryTractInfection | AddSubtype | 85915003 |
| 1055 | LowerRespiratoryTractInfection | AddSubtype | 187196002 |
| 1055 | LowerRespiratoryTractInfection | AddSubtype | 195949008 |
| 1055 | LowerRespiratoryTractInfection | AddSubtype | 195951007 |
| 1055 | LowerRespiratoryTractInfection | AddSubtype | 233604007 |
| 1055 | LowerRespiratoryTractInfection | AddSubtype | 233617005 |
| 1055 | LowerRespiratoryTractInfection | AddSubtype | 233619008 |
| 1055 | LowerRespiratoryTractInfection | AddSubtype | 314978007 |
| 1055 | LowerRespiratoryTractInfection | AddSubtype | 385093006 |
| 1055 | LowerRespiratoryTractInfection | AddSubtype | 1092951000119106 |
| 1055 | LowerRespiratoryTractInfection | AddSupertype | 4120002 |
| 1055 | LowerRespiratoryTractInfection | AddSupertype | 10509002 |
| 1055 | LowerRespiratoryTractInfection | AddSupertype | 13617004 |
| 1055 | LowerRespiratoryTractInfection | AddSupertype | 50417007 |
| 1055 | LowerRespiratoryTractInfection | AddSupertype | 58554001 |
| 1055 | LowerRespiratoryTractInfection | AddSupertype | 64667001 |
| 1055 | LowerRespiratoryTractInfection | AddSupertype | 73452002 |
| 1055 | LowerRespiratoryTractInfection | AddSupertype | 78895009 |
| 1055 | LowerRespiratoryTractInfection | AddSupertype | 278516003 |
| 1055 | LowerRespiratoryTractInfection | AddSupertype | 312134000 |
| 1055 | LowerRespiratoryTractInfection | AddSupertype | 314042000 |
| 1055 | LowerRespiratoryTractInfection | AddSupertype | 396285007 |
| 1055 | LowerRespiratoryTractInfection | AddSupertype | 398447004 |
| 1055 | LowerRespiratoryTractInfection | AddSupertype | 407671000 |
| 1055 | LowerRespiratoryTractInfection | AddSupertype | 417018008 |
| 1055 | LowerRespiratoryTractInfection | AddSupertype | 446986002 |
| 1055 | LowerRespiratoryTractInfection | MinusSubtype | 196019004 |
| 1055 | LowerRespiratoryTractInfection | MinusSubtype | 846639005 |
| 1055 | LowerRespiratoryTractInfection | MinusSubtype | 866901000000103 |
| 1055 | LowerRespiratoryTractInfection | MinusSupertype | 40100001 |
| 1055 | LowerRespiratoryTractInfection | MinusSupertype | 62994001 |
| 1055 | LowerRespiratoryTractInfection | MinusSupertype | 66011008 |
| 1055 | LowerRespiratoryTractInfection | MinusSupertype | 405720007 |
| 1055 | LowerRespiratoryTractInfection | MinusSupertype | 405944004 |
| 1055 | LowerRespiratoryTractInfection | MinusSupertype | 700249006 |
| 1055 | LowerRespiratoryTractInfection | MinusSupertype | 10625791000119101 |
| 7183 | SeenInHospitalCasualty | AddSubtype | 4525004 |
| 7183 | SeenInHospitalCasualty | AddSubtype | 305226003 |

| Condition ID | Condition Name | ECL Element | ConceptID |
|---|---|---|---|
| 7183 | SeenInHospitalCasualty | AddSubtype | 305451000 |
| 7183 | SeenInHospitalCasualty | AddSubtype | 305633005 |
| 7183 | SeenInHospitalCasualty | AddSubtype | 305925007 |
| 7183 | SeenInHospitalCasualty | AddSubtype | 306390007 |
| 7183 | SeenInHospitalCasualty | AddSubtype | 397721007 |
| 7183 | SeenInHospitalCasualty | AddSubtype | 413845009 |
| 7183 | SeenInHospitalCasualty | AddSubtype | 417119002 |
| 7183 | SeenInHospitalCasualty | AddSubtype | 507291000000100 |
| 7183 | SeenInHospitalCasualty | AddSubtype | 812481000000104 |
| 7183 | SeenInHospitalCasualty | AddSubtype | 812491000000102 |
| 7183 | SeenInHospitalCasualty | AddSubtype | 826931000000104 |
| 7183 | SeenInHospitalCasualty | AddSubtype | 963261000000108 |
| 7183 | SeenInHospitalCasualty | AddSubtype | 980491000000106 |
| 7183 | SeenInHospitalCasualty | AddSubtype | 989531000000100 |
| 7183 | SeenInHospitalCasualty | AddSubtype | 1066331000000109 |
| 7183 | SeenInHospitalCasualty | AddSubtype | 1066341000000100 |
| 7183 | SeenInHospitalCasualty | AddSubtype | 1066361000000104 |
| 7183 | SeenInHospitalCasualty | AddSubtype | 1066371000000106 |
| 7183 | SeenInHospitalCasualty | AddSubtype | 1066381000000108 |
| 7183 | SeenInHospitalCasualty | AddSubtype | 1082421000000101 |
| 7183 | SeenInHospitalCasualty | AddSupertype | 50849002 |
| 7183 | SeenInHospitalCasualty | AddSupertype | 185210004 |
| 7183 | SeenInHospitalCasualty | AddSupertype | 306563004 |
| 7183 | SeenInHospitalCasualty | AddSupertype | 1066391000000105 |
| 7183 | SeenInHospitalCasualty | AddSupertype | 1077011000000106 |
| 1007 | UpperRespiratoryInfection | AddSubtype | 8304007 |
| 1007 | UpperRespiratoryInfection | AddSubtype | 13420004 |
| 1007 | UpperRespiratoryInfection | AddSubtype | 16664009 |
| 1007 | UpperRespiratoryInfection | AddSubtype | 65363002 |
| 1007 | UpperRespiratoryInfection | AddSubtype | 194202008 |
| 1007 | UpperRespiratoryInfection | AddSubtype | 194203003 |
| 1007 | UpperRespiratoryInfection | AddSubtype | 194286008 |
| 1007 | UpperRespiratoryInfection | AddSubtype | 195680000 |
| 1007 | UpperRespiratoryInfection | AddSubtype | 232311007 |
| 1007 | UpperRespiratoryInfection | AddSubtype | 271567008 |
| 1007 | UpperRespiratoryInfection | AddSubtype | 276443001 |
| 1007 | UpperRespiratoryInfection | AddSubtype | 315642008 |
| 1007 | UpperRespiratoryInfection | AddSubtype | 1090001000119105 |
| 1007 | UpperRespiratoryInfection | AddSubtype | 1240521000000100 |
| 1007 | UpperRespiratoryInfection | AddSupertype | 140004 |
| 1007 | UpperRespiratoryInfection | AddSupertype | 297009 |
| 1007 | UpperRespiratoryInfection | AddSupertype | 3110003 |
| 1007 | UpperRespiratoryInfection | AddSupertype | 6142004 |
| 1007 | UpperRespiratoryInfection | AddSupertype | 27836007 |

| Condition ID | Condition Name | ECL Element | ConceptID |
|---|---|---|---|
| 1007 | UpperRespiratoryInfection | AddSupertype | 33924006 |
| 1007 | UpperRespiratoryInfection | AddSupertype | 36971009 |
| 1007 | UpperRespiratoryInfection | AddSupertype | 45913009 |
| 1007 | UpperRespiratoryInfection | AddSupertype | 54150009 |
| 1007 | UpperRespiratoryInfection | AddSupertype | 54398005 |
| 1007 | UpperRespiratoryInfection | AddSupertype | 59707005 |
| 1007 | UpperRespiratoryInfection | AddSupertype | 62994001 |
| 1007 | UpperRespiratoryInfection | AddSupertype | 64531003 |
| 1007 | UpperRespiratoryInfection | AddSupertype | 80327007 |
| 1007 | UpperRespiratoryInfection | AddSupertype | 80384002 |
| 1007 | UpperRespiratoryInfection | AddSupertype | 90176007 |
| 1007 | UpperRespiratoryInfection | AddSupertype | 195709006 |
| 1007 | UpperRespiratoryInfection | AddSupertype | 312218008 |
| 1007 | UpperRespiratoryInfection | AddSupertype | 359609001 |
| 1007 | UpperRespiratoryInfection | AddSupertype | 405737000 |
| 1007 | UpperRespiratoryInfection | AddSupertype | 723880004 |
| 1007 | UpperRespiratoryInfection | MinusSubtype | 1282001 |
| 1007 | UpperRespiratoryInfection | MinusSubtype | 2091005 |
| 1007 | UpperRespiratoryInfection | MinusSubtype | 54287007 |
| 1007 | UpperRespiratoryInfection | MinusSubtype | 59454008 |
| 1007 | UpperRespiratoryInfection | MinusSubtype | 59471009 |
| 1007 | UpperRespiratoryInfection | MinusSubtype | 87326000 |
| 1007 | UpperRespiratoryInfection | MinusSubtype | 111277007 |
| 1007 | UpperRespiratoryInfection | MinusSubtype | 195663004 |
| 1007 | UpperRespiratoryInfection | MinusSubtype | 232389000 |
| 1007 | UpperRespiratoryInfection | MinusSubtype | 232458003 |
| 1007 | UpperRespiratoryInfection | MinusSubtype | 262678008 |
| 1007 | UpperRespiratoryInfection | MinusSubtype | 307500008 |
| 1007 | UpperRespiratoryInfection | MinusSubtype | 425011002 |
| 1007 | UpperRespiratoryInfection | MinusSubtype | 427780002 |
| 1007 | UpperRespiratoryInfection | MinusSubtype | 10692681000119108 |
| 1007 | UpperRespiratoryInfection | MinusSupertype | 17904003 |
| 1007 | UpperRespiratoryInfection | MinusSupertype | 23919004 |
| 1007 | UpperRespiratoryInfection | MinusSupertype | 86773000 |
| 1007 | UpperRespiratoryInfection | MinusSupertype | 232214001 |
| 1007 | UpperRespiratoryInfection | MinusSupertype | 402698005 |

**Table S3: DM+D codes for prescriptions for asthma**

| ConditionID | Condition name | DMD code | Description |
|---|---|---|---|
| 5153 | Antimuscarinics | 320339009 | Ipratropium bromide 40microgram inhalation powder capsules |
| 5153 | Antimuscarinics | 320340006 | Ipratropium bromide 40microgram inhalation powder capsules with device |

| Con ditio nID | Condition name | DMD code | Description |
|---|---|---|---|
| 5153 | Antimuscarinics | 320345001 | Ipratropium bromide 20micrograms/dose breath actuated inhaler |
| 5153 | Antimuscarinics | 320346000 | Ipratropium bromide 250micrograms/1ml nebuliser liquid unit dose vials |
| 5153 | Antimuscarinics | 320347009 | Ipratropium bromide 500micrograms/2ml nebuliser liquid unit dose vials |
| 5153 | Antimuscarinics | 320348004 | Ipratropium bromide 20micrograms/dose inhaler |
| 5153 | Antimuscarinics | 320350007 | Ipratropium bromide 40micrograms/dose inhaler |
| 5153 | Antimuscarinics | 320440005 | Fenoterol 100micrograms/dose / Ipratropium 40micrograms/dose inhaler |
| 5153 | Antimuscarinics | 320441009 | Fenoterol 100micrograms/dose / Ipratropium bromide 40micrograms/dose breath actuated inhaler |
| 5153 | Antimuscarinics | 320442002 | Salbutamol 100micrograms/dose / Ipratropium 20micrograms/dose inhaler |
| 5153 | Antimuscarinics | 320445000 | Salbutamol 2.5mg/2.5ml / Ipratropium bromide 500micrograms/2.5ml nebuliser liquid unit dose vials |
| 5153 | Antimuscarinics | 349394001 | Fenoterol 1.25mg/4ml / Ipratropium 500micrograms/4ml nebuliser liquid unit dose vials |
| 5153 | Antimuscarinics | 414538008 | Ipratropium bromide 20mcg CFC-free inhaler |
| 5153 | Antimuscarinics | 703924000 | Aclidinium bromide 375micrograms inhaler |
| 5153 | Antimuscarinics | 24011000001103 | Atrovent 20micrograms/dose inhaler (Boehringer Ingelheim Ltd) |
| 5153 | Antimuscarinics | 109751000001104 | Fenoterol 1.25mg/4ml / Ipratropium 500micrograms/4ml nebuliser liquid unit dose vials |
| 5153 | Antimuscarinics | 110221000001105 | Ipratropium bromide 500micrograms/2ml nebuliser liquid unit dose vials |
| 5153 | Antimuscarinics | 110481000001107 | Ipratropium bromide 250micrograms/1ml nebuliser liquid unit dose vials |
| 5153 | Antimuscarinics | 110651000001100 | Salbutamol 2.5mg/2.5ml / Ipratropium bromide 500micrograms/2.5ml nebuliser liquid unit dose vials |
| 5153 | Antimuscarinics | 111781000001105 | Ipratropium bromide 40microgram inhalation powder capsules with device |
| 5153 | Antimuscarinics | 112141000001107 | Ipratropium bromide 40microgram inhalation powder capsules |
| 5153 | Antimuscarinics | 347611000001103 | Atrovent Forte 40micrograms/dose inhaler (Boehringer Ingelheim Ltd) |
| 5153 | Antimuscarinics | 351411000001106 | Atrovent 20micrograms/dose Autohaler (Boehringer Ingelheim Ltd) |
| 5153 | Antimuscarinics | 2923111000001107 | Duovent inhaler (Boehringer Ingelheim Ltd) |
| 5153 | Antimuscarinics | 2964011000001104 | Duovent Autohaler (Boehringer Ingelheim Ltd) |
| 5153 | Antimuscarinics | 3221011000001103 | Ipratropium bromide 500micrograms/2ml nebuliser liquid unit dose vials (A A H Pharmaceuticals Ltd) |
| 5153 | Antimuscarinics | 3221911000001104 | Tropiovent 500micrograms/2ml nebuliser liquid unit dose Steripoule vials (Ashbourne Pharmaceuticals Ltd) |
| 5153 | Antimuscarinics | 3222411000001102 | Atrovent 500micrograms/2ml nebuliser liquid UDVs (Boehringer Ingelheim Ltd) |
| 5153 | Antimuscarinics | 3222711000001108 | Ipratropium bromide 500micrograms/2ml nebuliser liquid unit dose vials (The Boots Company Plc) |
| 5153 | Antimuscarinics | 3222911000001105 | Respontin 500micrograms/2ml Nebules (GlaxoSmithKline UK Ltd) |
| 5153 | Antimuscarinics | 3223111000001101 | Ipratropium 500micrograms/2ml nebuliser liquid Steri-Neb unit dose vials (Teva UK Ltd) |
| 5153 | Antimuscarinics | 3223511000001105 | Ipratropium bromide 500micrograms/2ml nebuliser liquid unit dose vials (Alliance Healthcare (Distribution) Ltd) |
| 5153 | Antimuscarinics | 3225311000001103 | Ipratropium bromide 250micrograms/1ml nebuliser liquid unit dose vials (A A H Pharmaceuticals Ltd) |
| 5153 | Antimuscarinics | 3225811000001107 | Tropiovent 250micrograms/1ml nebuliser liquid unit dose Steripoule vials (Ashbourne Pharmaceuticals Ltd) |
| 5153 | Antimuscarinics | 3226411000001101 | Atrovent 250micrograms/1ml nebuliser liquid UDVs (Boehringer Ingelheim Ltd) |
| 5153 | Antimuscarinics | 3226811000001104 | Ipratropium bromide 250micrograms/1ml nebuliser liquid unit dose vials (The Boots Company Plc) |
| 5153 | Antimuscarinics | 3227011000001108 | Respontin 250micrograms/1ml Nebules (GlaxoSmithKline UK Ltd) |
| 5153 | Antimuscarinics | 3227211000001103 | Ipratropium 250micrograms/1ml nebuliser liquid Steri-Neb unit dose vials (Teva UK Ltd) |
| 5153 | Antimuscarinics | 3227511000001100 | Ipratropium bromide 250micrograms/1ml nebuliser liquid unit dose vials (Alliance Healthcare (Distribution) Ltd) |
| 5153 | Antimuscarinics | 3235911000001100 | Atrovent 40microgram Aerocaps (Boehringer Ingelheim Ltd) |
| 5153 | Antimuscarinics | 3238911000001106 | Atrovent 40microgram Aerocaps with Aerohaler (Boehringer Ingelheim Ltd) |

| Con ditio nID | Condition name | DMD code | Description |
|---|---|---|---|
| 5153 | Antimuscarinics | 3348611000001107 | Combivent inhaler (Boehringer Ingelheim Ltd) |
| 5153 | Antimuscarinics | 3378211000001106 | Spiriva 18microgram inhalation powder capsules with HandiHaler (Boehringer Ingelheim Ltd) |
| 5153 | Antimuscarinics | 3380011000001106 | Spiriva 18microgram inhalation powder capsules (Boehringer Ingelheim Ltd) |
| 5153 | Antimuscarinics | 3406011000001104 | Combivent nebuliser liquid 2.5ml UDVs (Boehringer Ingelheim Ltd) |
| 5153 | Antimuscarinics | 4192411000001109 | Duovent UDVs nebuliser liquid 4ml (Boehringer Ingelheim Ltd) |
| 5153 | Antimuscarinics | 5255211000001100 | Atrovent 20micrograms/dose inhaler (Waymade Healthcare Plc) |
| 5153 | Antimuscarinics | 5259111000001108 | Combivent inhaler (Waymade Healthcare Plc) |
| 5153 | Antimuscarinics | 5281611000001108 | Atrovent 20micrograms/dose inhaler (Dowelhurst Ltd) |
| 5153 | Antimuscarinics | 5287111000001107 | Combivent inhaler (Dowelhurst Ltd) |
| 5153 | Antimuscarinics | 5287711000001108 | Combivent nebuliser liquid 2.5ml UDVs (Dowelhurst Ltd) |
| 5153 | Antimuscarinics | 5297911000001106 | Ipratropium 20micrograms/dose inhaler (Dowelhurst Ltd) |
| 5153 | Antimuscarinics | 5319611000001103 | Spiriva 18microgram inhalation powder capsules with HandiHaler (Dowelhurst Ltd) |
| 5153 | Antimuscarinics | 7389911000001105 | Atrovent 20micrograms/dose inhaler CFC free (Boehringer Ingelheim Ltd) |
| 5153 | Antimuscarinics | 8097311000001109 | Ipratropium bromide 250micrograms/1ml nebuliser liquid unit dose vials (Kent Pharma (UK) Ltd) |
| 5153 | Antimuscarinics | 8097811000001100 | Ipratropium bromide 500micrograms/2ml nebuliser liquid unit dose vials (Kent Pharma (UK) Ltd) |
| 5153 | Antimuscarinics | 9039611000001103 | Ipratropium 250micrograms/1ml nebuliser liquid unit dose Steripoule vials (Galen Ltd) |
| 5153 | Antimuscarinics | 9039811000001104 | Ipratropium 500micrograms/2ml nebuliser liquid unit dose Steripoule vials (Galen Ltd) |
| 5153 | Antimuscarinics | 9101911000001108 | Ipratropium bromide 250micrograms/1ml nebuliser liquid unit dose vials (Teva UK Ltd) |
| 5153 | Antimuscarinics | 9102111000001100 | Ipratropium bromide 500micrograms/2ml nebuliser liquid unit dose vials (Teva UK Ltd) |
| 5153 | Antimuscarinics | 9478911000001107 | Tiotropium bromide 18microgram inhalation powder capsules |
| 5153 | Antimuscarinics | 9479011000001103 | Tiotropium bromide 18microgram inhalation powder capsules with device |
| 5153 | Antimuscarinics | 10453411000001106 | Ipratropium bromide 250micrograms/1ml nebuliser liquid unit dose vials (Arrow Generics Ltd) |
| 5153 | Antimuscarinics | 10453611000001109 | Ipratropium bromide 500micrograms/2ml nebuliser liquid unit dose vials (Arrow Generics Ltd) |
| 5153 | Antimuscarinics | 10469711000001108 | Spiriva 18microgram inhalation powder capsules (Dowelhurst Ltd) |
| 5153 | Antimuscarinics | 10509311000001101 | Spiriva 18microgram inhalation powder capsules with HandiHaler (Waymade Healthcare Plc) |
| 5153 | Antimuscarinics | 10776911000001102 | Combivent nebuliser liquid 2.5ml UDVs (Waymade Healthcare Plc) |
| 5153 | Antimuscarinics | 10785611000001108 | Spiriva 18microgram inhalation powder capsules (Waymade Healthcare Plc) |
| 5153 | Antimuscarinics | 10927511000001104 | Ipramol nebuliser solution 2.5ml Steri-Neb unit dose vials (Teva UK Ltd) |
| 5153 | Antimuscarinics | 11006111000001102 | Ipratropium bromide 250micrograms/1ml nebuliser liquid unit dose vials (Accord Healthcare Ltd) |
| 5153 | Antimuscarinics | 11006311000001100 | Ipratropium bromide 500micrograms/2ml nebuliser liquid unit dose vials (Accord Healthcare Ltd) |
| 5153 | Antimuscarinics | 12146911000001103 | Spiriva Respimat 2.5micrograms/dose inhalation solution cartridge with device (Boehringer Ingelheim Ltd) |
| 5153 | Antimuscarinics | 12197411000001102 | Tiotropium bromide 2.5micrograms/dose solution for inhalation cartridge with device CFC free |
| 5153 | Antimuscarinics | 13163001000001106 | Ipratropium bromide 20micrograms/dose breath actuated inhaler |
| 5153 | Antimuscarinics | 13164501000001104 | Fenoterol 100micrograms/dose / Ipratropium 40micrograms/dose inhaler |
| 5153 | Antimuscarinics | 13164601000001100 | Fenoterol 100micrograms/dose / Ipratropium bromide 40micrograms/dose breath actuated inhaler |
| 5153 | Antimuscarinics | 13164701000001105 | Salbutamol 100micrograms/dose / Ipratropium 20micrograms/dose inhaler |
| 5153 | Antimuscarinics | 13266301000001102 | Ipratropium bromide 20micrograms/dose inhaler CFC free |
| 5153 | Antimuscarinics | 13952411000001104 | Spiriva 18microgram inhalation powder capsules with HandiHaler (DE Pharmaceuticals) |
| 5153 | Antimuscarinics | 13953011000001104 | Spiriva 18microgram inhalation powder capsules (DE Pharmaceuticals) |

| Con ditio nID | Condition name | DMD code | Description |
|---|---|---|---|
| 5153 | Antimuscarinics | 14206211000001105 | Atrovent 20micrograms/dose inhaler CFC free (Sigma Pharmaceuticals Plc) |
| 5153 | Antimuscarinics | 14214011000001107 | Duovent UDVs nebuliser liquid 4ml (Sigma Pharmaceuticals Plc) |
| 5153 | Antimuscarinics | 14382411000001103 | Spiriva 18microgram inhalation powder capsules (Sigma Pharmaceuticals Plc) |
| 5153 | Antimuscarinics | 14384011000001101 | Spiriva 18microgram inhalation powder capsules with HandiHaler (Sigma Pharmaceuticals Plc) |
| 5153 | Antimuscarinics | 15213511000001109 | Ipratropium bromide 250micrograms/1ml nebuliser liquid unit dose vials (Sigma Pharmaceuticals Plc) |
| 5153 | Antimuscarinics | 15214311000001101 | Ipratropium bromide 500micrograms/2ml nebuliser liquid unit dose vials (Sigma Pharmaceuticals Plc) |
| 5153 | Antimuscarinics | 15522411000001103 | Salipraneb 0.5mg/2.5mg nebuliser solution 2.5ml ampoules (Arrow Generics Ltd) |
| 5153 | Antimuscarinics | 15534911000001100 | Salbutamol 2.5mg/2.5ml / Ipratropium bromide 500micrograms/2.5ml nebuliser liquid ampoules |
| 5153 | Antimuscarinics | 16733611000001107 | Salbutamol 2.5mg/2.5ml / Ipratropium bromide 500micrograms/2.5ml nebuliser liquid ampoules (A A H Pharmaceuticals Ltd) |
| 5153 | Antimuscarinics | 17454411000001104 | Spiriva 18microgram inhalation powder capsules with HandiHaler (Mawdsley-Brooks & Company Ltd) |
| 5153 | Antimuscarinics | 17454611000001101 | Spiriva Respimat 2.5micrograms/dose solution for inhalation cartridge with device (Mawdsley-Brooks & Company Ltd) |
| 5153 | Antimuscarinics | 17933111000001105 | Ipratropium bromide 250micrograms/1ml nebuliser liquid unit dose vials (Phoenix Healthcare Distribution Ltd) |
| 5153 | Antimuscarinics | 17933311000001107 | Ipratropium bromide 500micrograms/2ml nebuliser liquid unit dose vials (Phoenix Healthcare Distribution Ltd) |
| 5153 | Antimuscarinics | 18071311000001107 | Atrovent 20micrograms/dose inhaler CFC free (Lexon (UK) Ltd) |
| 5153 | Antimuscarinics | 18155011000001100 | Spiriva 18microgram inhalation powder capsules (Mawdsley-Brooks & Company Ltd) |
| 5153 | Antimuscarinics | 18220811000001104 | Combivent nebuliser liquid 2.5ml UDVs (Mawdsley-Brooks & Company Ltd) |
| 5153 | Antimuscarinics | 19541511000001101 | Combivent nebuliser liquid 2.5ml UDVs (DE Pharmaceuticals) |
| 5153 | Antimuscarinics | 19869411000001109 | Combivent nebuliser liquid 2.5ml UDVs (Lexon (UK) Ltd) |
| 5153 | Antimuscarinics | 20164811000001104 | Atrovent 20micrograms/dose inhaler CFC free (DE Pharmaceuticals) |
| 5153 | Antimuscarinics | 20985511000001101 | Eklira 322micrograms/dose Genuair (AstraZeneca UK Ltd) |
| 5153 | Antimuscarinics | 21021211000001107 | Aclidinium bromide 375micrograms/dose dry powder inhaler |
| 5153 | Antimuscarinics | 21495411000001107 | Seebri Breezhaler 44microgram inhalation powder capsules with device (Novartis Pharmaceuticals UK Ltd) |
| 5153 | Antimuscarinics | 21496211000001102 | Glycopyrronium bromide 55microgram inhalation powder capsules with device |
| 5153 | Antimuscarinics | 21508411000001109 | Atrovent 500micrograms/2ml nebuliser liquid UDVs (Waymade Healthcare Plc) |
| 5153 | Antimuscarinics | 21719511000001100 | Ipratropium bromide 250micrograms/1ml nebuliser liquid unit dose vials (Advanz Pharma) |
| 5153 | Antimuscarinics | 21720011000001109 | Ipratropium bromide 500micrograms/2ml nebuliser liquid unit dose vials (Advanz Pharma) |
| 5153 | Antimuscarinics | 22062311000001100 | Ipratropium bromide 250micrograms/1ml nebuliser liquid unit dose vials (Waymade Healthcare Plc) |
| 5153 | Antimuscarinics | 22062511000001106 | Ipratropium bromide 500micrograms/2ml nebuliser liquid unit dose vials (Waymade Healthcare Plc) |
| 5153 | Antimuscarinics | 23433411000001108 | Salbutamol 2.5mg/2.5ml / Ipratropium bromide 500micrograms/2.5ml nebuliser liquid unit dose vials (J M McGill Ltd) |
| 5153 | Antimuscarinics | 23949111000001102 | Ipratropium bromide 250micrograms/1ml nebuliser liquid unit dose vials (DE Pharmaceuticals) |
| 5153 | Antimuscarinics | 23949311000001100 | Ipratropium bromide 500micrograms/2ml nebuliser liquid unit dose vials (DE Pharmaceuticals) |
| 5153 | Antimuscarinics | 23961011000001108 | Salbutamol 2.5mg/2.5ml / Ipratropium bromide 500micrograms/2.5ml nebuliser liquid unit dose vials (Niche Pharma Ltd) |
| 5153 | Antimuscarinics | 24498211000001109 | Spiriva Respimat 2.5micrograms/dose solution for inhalation cartridge with device (Waymade Healthcare Plc) |
| 5153 | Antimuscarinics | 24644611000001108 | Anoro Ellipta 55micrograms/dose / 22micrograms/dose dry powder inhaler (GlaxoSmithKline UK Ltd) |
| 5153 | Antimuscarinics | 24645511000001105 | Umeclidinium bromide 65micrograms/dose / Vilanterol 22micrograms/dose dry powder inhaler |
| 5153 | Antimuscarinics | 27567911000001101 | Incruse Ellipta 55micrograms/dose dry powder inhaler (GlaxoSmithKline UK Ltd) |

| Con ditio nID | Condition name | DMD code | Description |
|---|---|---|---|
| 5153 | Antimuscarinics | 27872911000001109 | Ipratropium bromide 20micrograms/dose inhaler CFC free (A A H Pharmaceuticals Ltd) |
| 5153 | Antimuscarinics | 27890611000001109 | Umeclidinium bromide 65micrograms/dose dry powder inhaler |
| 5153 | Antimuscarinics | 28007211000001102 | Ultibro Breezhaler 85microgram/43microgram inhalation powder capsules with device (Novartis Pharmaceuticals UK Ltd) |
| 5153 | Antimuscarinics | 28049611000001104 | Indacaterol 85micrograms/dose / Glycopyrronium bromide 54micrograms/dose inhalation powder capsules with device |
| 5153 | Antimuscarinics | 28357211000001106 | Duaklir 340micrograms/dose / 12micrograms/dose Genuair (AstraZeneca UK Ltd) |
| 5153 | Antimuscarinics | 28365011000001100 | Aclidinium bromide 396micrograms/dose / Formoterol 11.8micrograms/dose dry powder inhaler |
| 5153 | Antimuscarinics | 28409511000001104 | Tiotropium bromide 2.5micrograms/dose solution for inhalation cartridge with device CFC free (AM Distributions (Yorkshire) Ltd) |
| 5153 | Antimuscarinics | 28410811000001107 | Berodual N inhaler (Imported (Germany)) |
| 5153 | Antimuscarinics | 28422511000001101 | Fenoterol 50micrograms/dose / Ipratropium bromide 20micrograms/dose inhaler CFC free |
| 5153 | Antimuscarinics | 28424911000001104 | Eklira 322micrograms/dose Genuair (Waymade Healthcare Plc) |
| 5153 | Antimuscarinics | 29211411000001103 | Salbutamol 2.5mg/2.5ml / Ipratropium bromide 500micrograms/2.5ml nebuliser liquid unit dose vials (Ennogen Healthcare Ltd) |
| 5153 | Antimuscarinics | 29971311000001100 | Spiolto Respimat 2.5micrograms/dose / 2.5micrograms/dose inhalation solution cartridge with device (Boehringer Ingelheim Ltd) |
| 5153 | Antimuscarinics | 29978611000001102 | Atrovent 250micrograms/1ml nebuliser liquid UDVs (Lexon (UK) Ltd) |
| 5153 | Antimuscarinics | 29978911000001108 | Atrovent 500micrograms/2ml nebuliser liquid UDVs (Lexon (UK) Ltd) |
| 5153 | Antimuscarinics | 29987211000001108 | Tiotropium bromide 2.5micrograms/dose / Olodaterol 2.5micrograms/dose solution for inhalation cartridge with device CFC free |
| 5153 | Antimuscarinics | 30215011000001107 | Ipratropium bromide 500micrograms/2ml nebuliser liquid unit dose vials (Mawdsley-Brooks & Company Ltd) |
| 5153 | Antimuscarinics | 30273611000001108 | Seebri Breezhaler 44microgram inhalation powder capsules with device (Waymade Healthcare Plc) |
| 5153 | Antimuscarinics | 30933811000001108 | Glycopyrronium bromide 55microgram inhalation powder capsules with device (Ennogen Healthcare Ltd) |
| 5153 | Antimuscarinics | 31451611000001103 | Aclidinium bromide 375micrograms/dose dry powder inhaler (J M McGill Ltd) |
| 5153 | Antimuscarinics | 31500911000001102 | Glycopyrronium bromide 55microgram inhalation powder capsules with device (J M McGill Ltd) |
| 5153 | Antimuscarinics | 32180811000001101 | Aclidinium bromide 375micrograms/dose dry powder inhaler (Niche Pharma Ltd) |
| 5153 | Antimuscarinics | 32336011000001106 | Glycopyrronium bromide 55microgram inhalation powder capsules with device (Colorama Pharmaceuticals Ltd) |
| 5153 | Antimuscarinics | 32364511000001104 | Umeclidinium bromide 65micrograms/dose / Vilanterol 22micrograms/dose dry powder inhaler (Ennogen Healthcare Ltd) |
| 5153 | Antimuscarinics | 32408711000001106 | Ipratropium bromide 20micrograms/dose inhaler CFC free (Kent Pharmaceuticals Ltd) |
| 5153 | Antimuscarinics | 32411611000001108 | Aclidinium bromide 375micrograms/dose dry powder inhaler (Colorama Pharmaceuticals Ltd) |
| 5153 | Antimuscarinics | 32480911000001107 | Salbutamol 2.5mg/2.5ml / Ipratropium bromide 500micrograms/2.5ml nebuliser liquid unit dose vials (Colorama Pharmaceuticals Ltd) |
| 5153 | Antimuscarinics | 32629811000001101 | Umeclidinium bromide 65micrograms/dose / Vilanterol 22micrograms/dose dry powder inhaler (J M McGill Ltd) |
| 5153 | Antimuscarinics | 32633011000001106 | Aclidinium bromide 396micrograms/dose / Formoterol 11.8micrograms/dose dry powder inhaler (Colorama Pharmaceuticals Ltd) |
| 5153 | Antimuscarinics | 32898611000001109 | Salipraneb 0.5mg/2.5mg nebuliser solution 2.5ml ampoules (Actavis UK Ltd) |
| 5153 | Antimuscarinics | 33594911000001100 | Braltus 10microgram inhalation powder capsules with Zonda inhaler (Teva UK Ltd) |
| 5153 | Antimuscarinics | 33596311000001107 | Tiotropium bromide 10microgram inhalation powder capsules with device |
| 5153 | Antimuscarinics | 34681611000001100 | Trimbow 87micrograms/dose / 5micrograms/dose / 9micrograms/dose inhaler (Chiesi Ltd) |
| 5153 | Antimuscarinics | 34683311000001106 | Generic Trimbow 87micrograms/dose / 5micrograms/dose / 9micrograms/dose inhaler |
| 5153 | Antimuscarinics | 34952211000001104 | Trelegy Ellipta 92micrograms/dose / 55micrograms/dose / 22micrograms/dose dry powder inhaler (GlaxoSmithKline UK Ltd) |
| 5153 | Antimuscarinics | 34954811000001109 | Inhalvent 20micrograms/dose inhaler (Alissa Healthcare Research Ltd) |
| 5153 | Antimuscarinics | 34955111000001103 | Generic Trelegy Ellipta 92micrograms/dose / 55micrograms/dose / 22micrograms/dose dry powder inhaler |

| Con ditio nID | Condition name | DMD code | Description |
|---|---|---|---|
| 5153 | Antimuscarinics | 35894111000001105 | Aclidinium bromide 375micrograms/dose dry powder inhaler |
| 5153 | Antimuscarinics | 35936311000001102 | Salbutamol 100micrograms/dose / Ipratropium 20micrograms/dose inhaler |
| 5153 | Antimuscarinics | 36049211000001106 | Ipratropium bromide 20micrograms/dose breath actuated inhaler |
| 5153 | Antimuscarinics | 36049311000001103 | Ipratropium bromide 20micrograms/dose inhaler |
| 5153 | Antimuscarinics | 36049411000001105 | Ipratropium bromide 40micrograms/dose inhaler |
| 5153 | Antimuscarinics | 36066611000001106 | Fenoterol 100micrograms/dose / Ipratropium 40micrograms/dose inhaler |
| 5153 | Antimuscarinics | 36066711000001102 | Fenoterol 100micrograms/dose / Ipratropium bromide 40micrograms/dose breath actuated inhaler |
| 5153 | Antimuscarinics | 37344011000001102 | Atrovent 20micrograms/dose inhaler CFC free (Mawdsley-Brooks & Company Ltd) |
| 5153 | Antimuscarinics | 37344411000001106 | Atrovent 250micrograms/1ml nebuliser liquid UDVs (Mawdsley-Brooks & Company Ltd) |
| 5153 | Antimuscarinics | 37344911000001103 | Atrovent 500micrograms/2ml nebuliser liquid UDVs (Mawdsley-Brooks & Company Ltd) |
| 5153 | Antimuscarinics | 37364411000001100 | Combivent nebuliser liquid 2.5ml UDVs (CST Pharma Ltd) |
| 5153 | Antimuscarinics | 37394911000001105 | Spiriva 18microgram inhalation powder capsules (CST Pharma Ltd) |
| 5153 | Antimuscarinics | 37399011000001107 | Eklira 322micrograms/dose Genuair (Mawdsley-Brooks & Company Ltd) |
| 5153 | Antimuscarinics | 37439611000001101 | Incruse Ellipta 55micrograms/dose dry powder inhaler (CST Pharma Ltd) |
| 5153 | Antimuscarinics | 37511511000001102 | Incruse Ellipta 55micrograms/dose dry powder inhaler (Mawdsley-Brooks & Company Ltd) |
| 5153 | Antimuscarinics | 37540311000001105 | Yanimo Respimat 2.5micrograms/dose / 2.5micrograms/dose inhalation solution cartridge with device (Boehringer Ingelheim Ltd) |
| 5153 | Antimuscarinics | 37593111000001108 | Seebri Breezhaler 44microgram inhalation powder capsules with device (CST Pharma Ltd) |
| 5153 | Antimuscarinics | 37596311000001101 | Atrovent 20micrograms/dose inhaler CFC free (CST Pharma Ltd) |
| 5153 | Antimuscarinics | 37625611000001100 | Combivent nebuliser liquid 2.5ml UDVs (Pilsco Ltd) |
| 5153 | Antimuscarinics | 37633411000001104 | Eklira 322micrograms/dose Genuair (Pilsco Ltd) |
| 5153 | Antimuscarinics | 37666611000001102 | Incruse Ellipta 55micrograms/dose dry powder inhaler (Ethigen Ltd) |
| 5153 | Antimuscarinics | 37677711000001102 | Spiriva Respimat 2.5micrograms/dose inhalation solution refill cartridge (Boehringer Ingelheim Ltd) |
| 5153 | Antimuscarinics | 37678011000001103 | Spiolto Respimat 2.5micrograms/dose / 2.5micrograms/dose inhalation solution refill cartridge (Boehringer Ingelheim Ltd) |
| 5153 | Antimuscarinics | 37692511000001100 | Tiotropium bromide 2.5micrograms/dose / Olodaterol 2.5micrograms/dose inhalation solution cartridge CFC free |
| 5153 | Antimuscarinics | 37692711000001105 | Tiotropium bromide 2.5micrograms/dose inhalation solution cartridge CFC free |
| 5153 | Antimuscarinics | 37716011000001104 | Braltus 10microgram inhalation powder capsules with Zonda inhaler (Pilsco Ltd) |
| 5153 | Antimuscarinics | 37728211000001103 | Seebri Breezhaler 44microgram inhalation powder capsules with device (Pilsco Ltd) |
| 5153 | Antimuscarinics | 37730011000001109 | Spiriva 18microgram inhalation powder capsules with HandiHaler (Pilsco Ltd) |
| 5153 | Antimuscarinics | 37743311000001100 | Spiriva 18microgram inhalation powder capsules with HandiHaler (CST Pharma Ltd) |
| 5153 | Antimuscarinics | 37743711000001101 | Braltus 10microgram inhalation powder capsules with Zonda inhaler (CST Pharma Ltd) |
| 5153 | Antimuscarinics | 37842911000001109 | Ipravent 20micrograms/dose inhaler CFC free (Cipla EU Ltd) |
| 5153 | Antimuscarinics | 37979411000001103 | Anoro Ellipta 55micrograms/dose / 22micrograms/dose dry powder inhaler (Pharmaram Ltd) |
| 5153 | Antimuscarinics | 37989411000001108 | Incruse Ellipta 55micrograms/dose dry powder inhaler (Pharmaram Ltd) |
| 5153 | Antimuscarinics | 37996311000001109 | Seebri Breezhaler 44microgram inhalation powder capsules with device (Pharmaram Ltd) |
| 5153 | Antimuscarinics | 38120811000001104 | Braltus 10microgram inhalation powder capsules with Zonda inhaler (DE Pharmaceuticals) |
| 5153 | Antimuscarinics | 38131811000001105 | Eklira 322micrograms/dose Genuair (DE Pharmaceuticals) |
| 5153 | Antimuscarinics | 38140511000001104 | Incruse Ellipta 55micrograms/dose dry powder inhaler (DE Pharmaceuticals) |
| 5153 | Antimuscarinics | 38617811000001107 | Combiprasal 0.5mg/2.5mg nebuliser solution 2.5ml unit dose vials (TriOn Pharma Ltd) |

| Con ditio nID | Condition name | DMD code | Description |
|---|---|---|---|
| 5153 | Antimuscarinics | 38893611000001108 | Aclidinium bromide 375micrograms/dose dry powder inhaler |
| 5153 | Antimuscarinics | 38894511000001107 | Ipratropium bromide 20micrograms/dose inhaler CFC free |
| 5153 | Antimuscarinics | 39001311000001101 | Ipratropium bromide 250micrograms/1ml nebuliser liquid unit dose vials (Medihealth (Northern) Ltd) |
| 5153 | Antimuscarinics | 39001511000001107 | Ipratropium bromide 500micrograms/2ml nebuliser liquid unit dose vials (Medihealth (Northern) Ltd) |
| 5153 | Antimuscarinics | 39134511000001107 | Generic Enerzair Breezhaler 114micrograms/dose / 46micrograms/dose / 136micrograms/dose inhalation powder capsules with device |
| 5153 | Antimuscarinics | 39134711000001102 | Enerzair Breezhaler 114micrograms/dose / 46micrograms/dose / 136micrograms/dose inhalation powder capsules with device (Sandoz Ltd) |
| 5153 | Antimuscarinics | 39167711000001100 | Rolufta Ellipta 55micrograms/dose dry powder inhaler (Ethigen Ltd) |
| 5153 | Antimuscarinics | 39327311000001104 | Trixeo Aerosphere 5micrograms/dose / 7.2micrograms/dose / 160micrograms/dose pressurised inhaler (AstraZeneca UK Ltd) |
| 5153 | Antimuscarinics | 39329111000001107 | Bevespi Aerosphere 7.2micrograms/dose / 5micrograms/dose pressurised inhaler (AstraZeneca UK Ltd) |
| 5153 | Antimuscarinics | 39343511000001103 | Generic Trixeo Aerosphere 5micrograms/dose / 7.2micrograms/dose / 160micrograms/dose inhaler CFC free |
| 5153 | Antimuscarinics | 39343611000001104 | Glycopyrronium 7.2micrograms/dose / Formoterol 5micrograms/dose inhaler CFC free |
| 5153 | Antimuscarinics | 39408811000001105 | Spiriva 18microgram inhalation powder capsules (Pilsco Ltd) |
| 5153 | Antimuscarinics | 39605711000001106 | Tiogiva 18microgram inhalation powder capsules with device (Glenmark Pharmaceuticals Europe Ltd) |
| 5153 | Antimuscarinics | 39606211000001105 | Tiogiva 18microgram inhalation powder capsules (Glenmark Pharmaceuticals Europe Ltd) |
| 5153 | Antimuscarinics | 39666311000001103 | Acopair 18microgram inhalation powder capsules with NeumoHaler (Viatris UK Healthcare Ltd) |
| 5153 | Antimuscarinics | 39710511000001100 | Salbutamol 2.5mg/2.5ml / Ipratropium bromide 500micrograms/2.5ml nebuliser liquid unit dose vials |
| 5153 | Antimuscarinics | 39711511000001107 | Fenoterol 1.25mg/4ml / Ipratropium 500micrograms/4ml nebuliser liquid unit dose vials |
| 5153 | Antimuscarinics | 39717211000001104 | Ipratropium bromide 250micrograms/1ml nebuliser liquid unit dose vials |
| 5153 | Antimuscarinics | 39717311000001107 | Ipratropium bromide 40microgram inhalation powder capsules with device |
| 5153 | Antimuscarinics | 39717411000001100 | Ipratropium bromide 40microgram inhalation powder capsules |
| 5153 | Antimuscarinics | 39717511000001101 | Ipratropium bromide 500micrograms/2ml nebuliser liquid unit dose vials |
| 5153 | Antimuscarinics | 39993311000001105 | Trimbow NEXThaler 88micrograms/dose / 5micrograms/dose / 9micrograms/dose dry powder inhaler (Chiesi Ltd) |
| 5153 | Antimuscarinics | 40087411000001104 | Generic Trimbow NEXThaler 88micrograms/dose / 5micrograms/dose / 9micrograms/dose dry powder inhaler |
| 5153 | Antimuscarinics | 40478511000001100 | Seebri Breezhaler 44microgram inhalation powder capsules with device (DE Pharmaceuticals) |
| 5153 | Antimuscarinics | 40752211000001109 | Trimbow 172micrograms/dose / 5micrograms/dose / 9micrograms/dose inhaler (Chiesi Ltd) |
| 5153 | Antimuscarinics | 40766811000001104 | Generic Trimbow 172micrograms/dose / 5micrograms/dose / 9micrograms/dose inhaler |
| 5154 | Beta2AdrenoceptorAgonistsSelective | 3417111000001102 | Salbutamol 100micrograms/dose inhaler (Alliance Healthcare (Distribution) Ltd) |
| 5154 | Beta2AdrenoceptorAgonistsSelective | 3633911000001104 | Bricanyl 5mg/2ml Respules (AstraZeneca UK Ltd) |
| 5154 | Beta2AdrenoceptorAgonistsSelective | 3654411000001106 | Terbutaline 5mg/2ml nebuliser liquid unit dose vials |
| 5154 | Beta2AdrenoceptorAgonistsSelective | 3926911000001108 | Ventolin 500micrograms/1ml solution for injection ampoules (GlaxoSmithKline UK Ltd) |
| 5154 | Beta2AdrenoceptorAgonistsSelective | 4179611000001107 | Aerocrom Syncroner with spacer (Castlemead Healthcare Ltd) |
| 5154 | Beta2AdrenoceptorAgonistsSelective | 4192411000001109 | Duovent UDVs nebuliser liquid 4ml (Boehringer Ingelheim Ltd) |
| 5154 | Beta2AdrenoceptorAgonistsSelective | 4373811000001100 | Symbicort 400/12 Turbohaler (AstraZeneca UK Ltd) |
| 5154 | Beta2AdrenoceptorAgonistsSelective | 4378111000001103 | Budesonide 400micrograms/dose / Formoterol 12micrograms/dose dry powder inhaler |
| 5154 | Beta2AdrenoceptorAgonistsSelective | 4537611000001107 | Berotec 200micrograms/dose inhaler (Boehringer Ingelheim Ltd) |
| 5154 | Beta2AdrenoceptorAgonistsSelective | 4558411000001107 | Fenoterol 200micrograms/dose inhaler |
| 5154 | Beta2AdrenoceptorAgonistsSelective | 4843911000001107 | Ventolin 5mg/ml respirator solution (GlaxoSmithKline UK Ltd) |
| 5154 | Beta2AdrenoceptorAgonistsSelective | 4889111000001103 | Salbutamol 5mg/50ml solution for infusion vials (Martindale Pharmaceuticals Ltd) |

| Con ditio nID | Condition name | DMD code | Description |
|---|---|---|---|
| 5154 | Beta2AdrenoceptorAgonistsSelective | 5257611000001105 | Bricanyl 250micrograms/dose inhaler (Waymade Healthcare Plc) |
| 5154 | Beta2AdrenoceptorAgonistsSelective | 5257911000001104 | Bricanyl 500micrograms/dose Turbohaler (Waymade Healthcare Plc) |
| 5154 | Beta2AdrenoceptorAgonistsSelective | 5259111000001108 | Combivent inhaler (Waymade Healthcare Plc) |
| 5154 | Beta2AdrenoceptorAgonistsSelective | 5271811000001100 | Oxis 12 Turbohaler (Waymade Healthcare Plc) |
| 5154 | Beta2AdrenoceptorAgonistsSelective | 5272211000001108 | Oxis 6 Turbohaler (Waymade Healthcare Plc) |
| 5154 | Beta2AdrenoceptorAgonistsSelective | 5275011000001108 | Salmeterol 25micrograms/dose inhaler (Waymade Healthcare Plc) |
| 5154 | Beta2AdrenoceptorAgonistsSelective | 5275311000001106 | Serevent 25micrograms/dose inhaler (Waymade Healthcare Plc) |
| 5154 | Beta2AdrenoceptorAgonistsSelective | 5275611000001101 | Seretide 100 Accuhaler (Waymade Healthcare Plc) |
| 5154 | Beta2AdrenoceptorAgonistsSelective | 5276011000001104 | Seretide 250 Accuhaler (Waymade Healthcare Plc) |
| 5154 | Beta2AdrenoceptorAgonistsSelective | 5276211000001109 | Seretide 500 Accuhaler (Waymade Healthcare Plc) |
| 5154 | Beta2AdrenoceptorAgonistsSelective | 5276511000001107 | Serevent 50micrograms/dose Accuhaler (Waymade Healthcare Plc) |
| 5154 | Beta2AdrenoceptorAgonistsSelective | 5276811000001105 | Seretide 250 Evohaler (Waymade Healthcare Plc) |
| 5154 | Beta2AdrenoceptorAgonistsSelective | 5277211000001106 | Symbicort 200/6 Turbohaler (Waymade Healthcare Plc) |
| 5154 | Beta2AdrenoceptorAgonistsSelective | 5277411000001105 | Terbutaline 250micrograms/dose inhaler (Waymade Healthcare Plc) |
| 5154 | Beta2AdrenoceptorAgonistsSelective | 5277911000001102 | Ventolin 100micrograms/dose Evohaler (Waymade Healthcare Plc) |
| 5154 | Beta2AdrenoceptorAgonistsSelective | 5278711000001103 | Ventide inhaler (Waymade Healthcare Plc) |
| 5154 | Beta2AdrenoceptorAgonistsSelective | 5284911000001104 | Bricanyl 250micrograms/dose inhaler (Dowelhurst Ltd) |
| 5154 | Beta2AdrenoceptorAgonistsSelective | 5285911000001100 | Bricanyl 500micrograms/dose Turbohaler (Dowelhurst Ltd) |
| 5154 | Beta2AdrenoceptorAgonistsSelective | 5287111000001107 | Combivent inhaler (Dowelhurst Ltd) |
| 5154 | Beta2AdrenoceptorAgonistsSelective | 5287711000001108 | Combivent nebuliser liquid 2.5ml UDVs (Dowelhurst Ltd) |
| 5154 | Beta2AdrenoceptorAgonistsSelective | 5307111000001103 | Oxis 12 Turbohaler (Dowelhurst Ltd) |
| 5154 | Beta2AdrenoceptorAgonistsSelective | 5307711000001102 | Oxis 6 Turbohaler (Dowelhurst Ltd) |
| 5154 | Beta2AdrenoceptorAgonistsSelective | 5315611000001101 | Salmeterol 25micrograms/dose inhaler (Dowelhurst Ltd) |
| 5154 | Beta2AdrenoceptorAgonistsSelective | 5317111000001100 | Seretide 100 Accuhaler (Dowelhurst Ltd) |
| 5154 | Beta2AdrenoceptorAgonistsSelective | 5317311000001103 | Seretide 250 Accuhaler (Dowelhurst Ltd) |
| 5154 | Beta2AdrenoceptorAgonistsSelective | 5317511000001109 | Seretide 500 Accuhaler (Dowelhurst Ltd) |
| 5154 | Beta2AdrenoceptorAgonistsSelective | 5320411000001102 | Serevent 25micrograms/dose inhaler (Dowelhurst Ltd) |
| 5154 | Beta2AdrenoceptorAgonistsSelective | 5320611000001104 | Serevent 50micrograms/dose Accuhaler (Dowelhurst Ltd) |
| 5154 | Beta2AdrenoceptorAgonistsSelective | 5321011000001102 | Symbicort 200/6 Turbohaler (Dowelhurst Ltd) |
| 5154 | Beta2AdrenoceptorAgonistsSelective | 5321111000001101 | Serevent 50microgram disks with Diskhaler (Dowelhurst Ltd) |
| 5154 | Beta2AdrenoceptorAgonistsSelective | 5321611000001109 | Serevent 50microgram disks (Dowelhurst Ltd) |
| 5154 | Beta2AdrenoceptorAgonistsSelective | 5322811000001104 | Terbutaline 250micrograms/dose inhaler (Dowelhurst Ltd) |
| 5154 | Beta2AdrenoceptorAgonistsSelective | 5323111000001100 | Terbutaline 500micrograms/dose Turbohaler (Dowelhurst Ltd) |
| 5154 | Beta2AdrenoceptorAgonistsSelective | 5323811000001107 | Salmeterol 50microgram Diskhaler (Dowelhurst Ltd) |
| 5154 | Beta2AdrenoceptorAgonistsSelective | 5324211000001109 | Ventide inhaler (Dowelhurst Ltd) |
| 5154 | Beta2AdrenoceptorAgonistsSelective | 5324411000001108 | Ventolin 200micrograms/dose Accuhaler (Dowelhurst Ltd) |
| 5154 | Beta2AdrenoceptorAgonistsSelective | 5324811000001105 | Ventolin 200microgram Rotacaps (Dowelhurst Ltd) |
| 5154 | Beta2AdrenoceptorAgonistsSelective | 5411211000001107 | Salbutamol 8mg modified-release tablets (Waymade Healthcare Plc) |
| 5154 | Beta2AdrenoceptorAgonistsSelective | 5420011000001102 | Salbutamol 4mg modified-release tablets (Waymade Healthcare Plc) |
| 5154 | Beta2AdrenoceptorAgonistsSelective | 5443411000001101 | Volmax 4mg modified-release tablets (Dowelhurst Ltd) |
| 5154 | Beta2AdrenoceptorAgonistsSelective | 5443711000001107 | Volmax 8mg modified-release tablets (Dowelhurst Ltd) |
| 5154 | Beta2AdrenoceptorAgonistsSelective | 5583011000001107 | Ventolin 400microgram Rotacaps (Dowelhurst Ltd) |
| 5154 | Beta2AdrenoceptorAgonistsSelective | 5594511000001100 | Ventolin 100micrograms/dose Evohaler (Dowelhurst Ltd) |

| Con ditio nID | Condition name | DMD code | Description |
|---|---|---|---|
| 5154 | Beta2AdrenoceptorAgonistsSelective | 9040011000001103 | Salbutamol 2.5mg/2.5ml nebuliser liquid unit dose Steripoule vials (Galen Ltd) |
| 5154 | Beta2AdrenoceptorAgonistsSelective | 9040211000001108 | Salbutamol 5mg/2.5ml nebuliser liquid unit dose Steripoule vials (Galen Ltd) |
| 5154 | Beta2AdrenoceptorAgonistsSelective | 9102311000001103 | Salbutamol 2.5mg/2.5ml nebuliser liquid unit dose vials (Teva UK Ltd) |
| 5154 | Beta2AdrenoceptorAgonistsSelective | 9102511000001109 | Salbutamol 5mg/2.5ml nebuliser liquid unit dose vials (Teva UK Ltd) |
| 5154 | Beta2AdrenoceptorAgonistsSelective | 9111011000001106 | Terbutaline 5mg/2ml nebuliser liquid unit dose vials (Galen Ltd) |
| 5154 | Beta2AdrenoceptorAgonistsSelective | 9204911000001109 | Easyhaler Salbutamol sulfate 200micrograms/dose dry powder inhaler (Orion Pharma (UK) Ltd) |
| 5154 | Beta2AdrenoceptorAgonistsSelective | 9205211000001104 | Easyhaler Salbutamol sulfate 100micrograms/dose dry powder inhaler (Orion Pharma (UK) Ltd) |
| 5154 | Beta2AdrenoceptorAgonistsSelective | 9207411000001106 | Salbutamol 100micrograms/dose dry powder inhaler |
| 5154 | Beta2AdrenoceptorAgonistsSelective | 9239411000001108 | Terbutaline 1.5mg/5ml oral solution sugar free (A A H Pharmaceuticals Ltd) |
| 5154 | Beta2AdrenoceptorAgonistsSelective | 9628711000001106 | Atimos Modulite 12micrograms/dose inhaler (Chiesi Ltd) |
| 5154 | Beta2AdrenoceptorAgonistsSelective | 9652711000001107 | Formoterol 12micrograms/dose inhaler CFC free |
| 5154 | Beta2AdrenoceptorAgonistsSelective | 10073011000001107 | Serevent 25micrograms/dose Evohaler (GlaxoSmithKline UK Ltd) |
| 5154 | Beta2AdrenoceptorAgonistsSelective | 10075611000001101 | Salmeterol 25micrograms/dose inhaler CFC free |
| 5154 | Beta2AdrenoceptorAgonistsSelective | 10432311000001108 | Salbutamol 100micrograms/dose inhaler (Arrow Generics Ltd) |
| 5154 | Beta2AdrenoceptorAgonistsSelective | 10453811000001108 | Salbutamol 2.5mg/2.5ml nebuliser liquid unit dose vials (Arrow Generics Ltd) |
| 5154 | Beta2AdrenoceptorAgonistsSelective | 10454011000001100 | Salbutamol 5mg/2.5ml nebuliser liquid unit dose vials (Arrow Generics Ltd) |
| 5154 | Beta2AdrenoceptorAgonistsSelective | 10472811000001102 | Seretide 250 Evohaler (Dowelhurst Ltd) |
| 5154 | Beta2AdrenoceptorAgonistsSelective | 10473211000001109 | Seretide 125 Evohaler (Dowelhurst Ltd) |
| 5154 | Beta2AdrenoceptorAgonistsSelective | 10474211000001107 | Salbutamol 4mg modified-release tablets (Dowelhurst Ltd) |
| 5154 | Beta2AdrenoceptorAgonistsSelective | 10474711000001100 | Salbutamol 8mg modified-release tablets (Dowelhurst Ltd) |
| 5154 | Beta2AdrenoceptorAgonistsSelective | 10493811000001104 | Ventolin 200micrograms/dose Accuhaler (Waymade Healthcare Plc) |
| 5154 | Beta2AdrenoceptorAgonistsSelective | 10506611000001103 | Symbicort 400/12 Turbohaler (Waymade Healthcare Plc) |
| 5154 | Beta2AdrenoceptorAgonistsSelective | 10512011000001109 | Seretide 125 Evohaler (Waymade Healthcare Plc) |
| 5154 | Beta2AdrenoceptorAgonistsSelective | 10776911000001102 | Combivent nebuliser liquid 2.5ml UDVs (Waymade Healthcare Plc) |
| 5154 | Beta2AdrenoceptorAgonistsSelective | 10847711000001102 | Serevent 25micrograms/dose Evohaler (Waymade Healthcare Plc) |
| 5154 | Beta2AdrenoceptorAgonistsSelective | 10927511000001104 | Ipramol nebuliser solution 2.5ml Steri-Neb unit dose vials (Teva UK Ltd) |
| 5154 | Beta2AdrenoceptorAgonistsSelective | 10983311000001107 | Symbicort 100/6 Turbohaler (Waymade Healthcare Plc) |
| 5154 | Beta2AdrenoceptorAgonistsSelective | 11006511000001106 | Salbutamol 2.5mg/2.5ml nebuliser liquid unit dose vials (Accord Healthcare Ltd) |
| 5154 | Beta2AdrenoceptorAgonistsSelective | 11006711000001101 | Salbutamol 5mg/2.5ml nebuliser liquid unit dose vials (Accord Healthcare Ltd) |
| 5154 | Beta2AdrenoceptorAgonistsSelective | 11150411000001106 | Terbutaline 5mg/2ml nebuliser liquid unit dose vials (Accord Healthcare Ltd) |
| 5154 | Beta2AdrenoceptorAgonistsSelective | 11176411000001106 | Formoterol Easyhaler 12micrograms/dose dry powder inhaler (Orion Pharma (UK) Ltd) |
| 5154 | Beta2AdrenoceptorAgonistsSelective | 11256611000001108 | Terbutaline 5mg/2ml nebuliser liquid unit dose vials (A A H Pharmaceuticals Ltd) |
| 5154 | Beta2AdrenoceptorAgonistsSelective | 12062211000001109 | Terbutaline 5mg/2ml nebuliser liquid unit dose vials (Alliance Healthcare (Distribution) Ltd) |
| 5154 | Beta2AdrenoceptorAgonistsSelective | 12626211000001108 | Formoterol 12microgram inhalation powder capsules with device |
| 5154 | Beta2AdrenoceptorAgonistsSelective | 12880611000001109 | Salbutamol 2.5mg/2.5ml nebuliser liquid unit dose vials (Kent Pharma (UK) Ltd) |
| 5154 | Beta2AdrenoceptorAgonistsSelective | 12880811000001108 | Salbutamol 5mg/2.5ml nebuliser liquid unit dose vials (Kent Pharma (UK) Ltd) |
| 5154 | Beta2AdrenoceptorAgonistsSelective | 12906411000001100 | Fostair 100micrograms/dose / 6micrograms/dose inhaler (Chiesi Ltd) |
| 5154 | Beta2AdrenoceptorAgonistsSelective | 12911011000001100 | Beclometasone 100micrograms/dose / Formoterol 6micrograms/dose inhaler CFC free |
| 5154 | Beta2AdrenoceptorAgonistsSelective | 13132801000001101 | Budesonide 200micrograms/dose / Formoterol 6micrograms/dose dry powder inhaler |

| ConditionID | Condition name | DMD code | Description |
|---|---|---|---|
| 5154 | Beta2AdrenoceptorAgonistsSelective | 13159001000001101 | Salbutamol 4mg modified-release tablets |
| 5154 | Beta2AdrenoceptorAgonistsSelective | 13159101000001100 | Salbutamol 4mg modified-release capsules |
| 5154 | Beta2AdrenoceptorAgonistsSelective | 13159201000001107 | Salbutamol 8mg modified-release capsules |
| 5154 | Beta2AdrenoceptorAgonistsSelective | 13159401000001106 | Salbutamol 2mg/5ml oral solution sugar free |
| 5154 | Beta2AdrenoceptorAgonistsSelective | 13159601000001109 | Salbutamol 500micrograms/1ml solution for injection ampoules |
| 5154 | Beta2AdrenoceptorAgonistsSelective | 13159701000001104 | Salbutamol 5mg/5ml solution for infusion ampoules |
| 5154 | Beta2AdrenoceptorAgonistsSelective | 13159801000001108 | Salbutamol 200microgram inhalation powder blisters with device |
| 5154 | Beta2AdrenoceptorAgonistsSelective | 13159901000001102 | Salbutamol 400microgram inhalation powder blisters with device |
| 5154 | Beta2AdrenoceptorAgonistsSelective | 13160001000001100 | Salbutamol 200microgram inhalation powder blisters |
| 5154 | Beta2AdrenoceptorAgonistsSelective | 13160101000001104 | Salbutamol 400microgram inhalation powder blisters |
| 5154 | Beta2AdrenoceptorAgonistsSelective | 13160301000001102 | Salbutamol 100micrograms/dose inhaler CFC free |
| 5154 | Beta2AdrenoceptorAgonistsSelective | 13160401000001105 | Salbutamol 200micrograms/dose dry powder inhaler |
| 5154 | Beta2AdrenoceptorAgonistsSelective | 13160601000001108 | Salbutamol 100micrograms/dose breath actuated inhaler CFC free |
| 5154 | Beta2AdrenoceptorAgonistsSelective | 13160701000001103 | Salbutamol 100micrograms/dose inhaler |
| 5154 | Beta2AdrenoceptorAgonistsSelective | 13161101000001106 | Terbutaline 250micrograms/dose inhaler |
| 5154 | Beta2AdrenoceptorAgonistsSelective | 13161201000001104 | Terbutaline 250micrograms/dose inhaler with spacer |
| 5154 | Beta2AdrenoceptorAgonistsSelective | 13161501000001101 | Salmeterol 25micrograms/dose inhaler |
| 5154 | Beta2AdrenoceptorAgonistsSelective | 13161601000001102 | Salmeterol 50microgram inhalation powder blisters with device |
| 5154 | Beta2AdrenoceptorAgonistsSelective | 13161701000001107 | Salmeterol 50microgram inhalation powder blisters |
| 5154 | Beta2AdrenoceptorAgonistsSelective | 13161801000001103 | Salmeterol 50micrograms/dose dry powder inhaler |
| 5154 | Beta2AdrenoceptorAgonistsSelective | 13161901000001109 | Formoterol 6micrograms/dose dry powder inhaler |
| 5154 | Beta2AdrenoceptorAgonistsSelective | 13162001000001101 | Formoterol 12micrograms/dose dry powder inhaler |
| 5154 | Beta2AdrenoceptorAgonistsSelective | 13162101000001100 | Fluticasone 50micrograms/dose / Salmeterol 25micrograms/dose inhaler CFC free |
| 5154 | Beta2AdrenoceptorAgonistsSelective | 13162201000001107 | Fluticasone 125micrograms/dose / Salmeterol 25micrograms/dose inhaler CFC free |
| 5154 | Beta2AdrenoceptorAgonistsSelective | 13162301000001103 | Fluticasone 250micrograms/dose / Salmeterol 25micrograms/dose inhaler CFC free |
| 5154 | Beta2AdrenoceptorAgonistsSelective | 13162401000001106 | Fluticasone propionate 100micrograms/dose / Salmeterol 50micrograms/dose dry powder inhaler |
| 5154 | Beta2AdrenoceptorAgonistsSelective | 13162501000001105 | Fluticasone propionate 250micrograms/dose / Salmeterol 50micrograms/dose dry powder inhaler |
| 5154 | Beta2AdrenoceptorAgonistsSelective | 13162601000001109 | Fluticasone propionate 500micrograms/dose / Salmeterol 50micrograms/dose dry powder inhaler |
| 5154 | Beta2AdrenoceptorAgonistsSelective | 13164501000001104 | Fenoterol 100micrograms/dose / Ipratropium 40micrograms/dose inhaler |
| 5154 | Beta2AdrenoceptorAgonistsSelective | 13164601000001100 | Fenoterol 100micrograms/dose / Ipratropium bromide 40micrograms/dose breath actuated inhaler |
| 5154 | Beta2AdrenoceptorAgonistsSelective | 13164701000001105 | Salbutamol 100micrograms/dose / Ipratropium 20micrograms/dose inhaler |
| 5154 | Beta2AdrenoceptorAgonistsSelective | 13166001000001102 | Salbutamol 100micrograms/dose / Beclometasone 50micrograms/dose inhaler |
| 5154 | Beta2AdrenoceptorAgonistsSelective | 13168701000001101 | Sodium cromoglicate 1mg/dose / Salbutamol 100micrograms/dose inhaler with spacer |
| 5154 | Beta2AdrenoceptorAgonistsSelective | 13168801000001105 | Sodium cromoglicate 1mg/dose / Salbutamol 100micrograms/dose inhaler |
| 5154 | Beta2AdrenoceptorAgonistsSelective | 13203711000001107 | Serevent 25micrograms/dose Evohaler (Dowelhurst Ltd) |
| 5154 | Beta2AdrenoceptorAgonistsSelective | 13206411000001106 | Symbicort 100/6 Turbohaler (Dowelhurst Ltd) |
| 5154 | Beta2AdrenoceptorAgonistsSelective | 13206611000001109 | Symbicort 400/12 Turbohaler (Dowelhurst Ltd) |
| 5154 | Beta2AdrenoceptorAgonistsSelective | 13533511000001104 | Salbulin Novolizer 100micrograms/dose inhalation powder (Viatris UK Healthcare Ltd) |
| 5154 | Beta2AdrenoceptorAgonistsSelective | 13533711000001109 | Salbulin Novolizer 100micrograms/dose inhalation powder refill (Viatris UK Healthcare Ltd) |
| 5154 | Beta2AdrenoceptorAgonistsSelective | 13566111000001109 | Salbutamol 100micrograms/dose dry powder inhalation cartridge |

| Con ditio nID | Condition name | DMD code | Description |
|---|---|---|---|
| 5154 | Beta2AdrenoceptorAgonistsSelective | 13566211000001103 | Salbutamol 100micrograms/dose dry powder inhalation cartridge with device |
| 5154 | Beta2AdrenoceptorAgonistsSelective | 13626311000001105 | Salbutamol 2mg/5ml oral solution sugar free (Pinewood Healthcare) |
| 5154 | Beta2AdrenoceptorAgonistsSelective | 13837711000001100 | Bricanyl 500micrograms/dose Turbohaler (DE Pharmaceuticals) |
| 5154 | Beta2AdrenoceptorAgonistsSelective | 13943811000001108 | Oxis 6 Turbohaler (DE Pharmaceuticals) |
| 5154 | Beta2AdrenoceptorAgonistsSelective | 13944011000001100 | Oxis 12 Turbohaler (DE Pharmaceuticals) |
| 5154 | Beta2AdrenoceptorAgonistsSelective | 13958011000001106 | Symbicort 100/6 Turbohaler (DE Pharmaceuticals) |
| 5154 | Beta2AdrenoceptorAgonistsSelective | 13958611000001104 | Symbicort 200/6 Turbohaler (DE Pharmaceuticals) |
| 5154 | Beta2AdrenoceptorAgonistsSelective | 13959211000001106 | Symbicort 400/12 Turbohaler (DE Pharmaceuticals) |
| 5154 | Beta2AdrenoceptorAgonistsSelective | 13967411000001108 | Ventolin 200micrograms/dose Accuhaler (DE Pharmaceuticals) |
| 5154 | Beta2AdrenoceptorAgonistsSelective | 13968911000001104 | Ventolin 100micrograms/dose Evohaler (DE Pharmaceuticals) |
| 5154 | Beta2AdrenoceptorAgonistsSelective | 13991111000001107 | Terbutaline 500micrograms/dose Turbohaler (DE Pharmaceuticals) |
| 5154 | Beta2AdrenoceptorAgonistsSelective | 13997111000001100 | Seretide 100 Accuhaler (DE Pharmaceuticals) |
| 5154 | Beta2AdrenoceptorAgonistsSelective | 13997511000001109 | Seretide 250 Accuhaler (DE Pharmaceuticals) |
| 5154 | Beta2AdrenoceptorAgonistsSelective | 13997811000001107 | Serevent 50micrograms/dose Accuhaler (DE Pharmaceuticals) |
| 5154 | Beta2AdrenoceptorAgonistsSelective | 13998111000001104 | Seretide 125 Evohaler (DE Pharmaceuticals) |
| 5154 | Beta2AdrenoceptorAgonistsSelective | 13998411000001109 | Seretide 250 Evohaler (DE Pharmaceuticals) |
| 5154 | Beta2AdrenoceptorAgonistsSelective | 14207711000001100 | Bambec 10mg tablets (Sigma Pharmaceuticals Plc) |
| 5154 | Beta2AdrenoceptorAgonistsSelective | 14208011000001101 | Bambec 20mg tablets (Sigma Pharmaceuticals Plc) |
| 5154 | Beta2AdrenoceptorAgonistsSelective | 14214011000001107 | Duovent UDVs nebuliser liquid 4ml (Sigma Pharmaceuticals Plc) |
| 5154 | Beta2AdrenoceptorAgonistsSelective | 14239811000001105 | Foradil 12microgram inhalation powder capsules with device (Sigma Pharmaceuticals Plc) |
| 5154 | Beta2AdrenoceptorAgonistsSelective | 14620511000001104 | Symbicort 100/6 Turbohaler (Sigma Pharmaceuticals Plc) |
| 5154 | Beta2AdrenoceptorAgonistsSelective | 14620711000001109 | Symbicort 200/6 Turbohaler (Sigma Pharmaceuticals Plc) |
| 5154 | Beta2AdrenoceptorAgonistsSelective | 14621111000001102 | Symbicort 400/12 Turbohaler (Sigma Pharmaceuticals Plc) |
| 5154 | Beta2AdrenoceptorAgonistsSelective | 14662411000001107 | Bricanyl 500micrograms/dose Turbohaler (Sigma Pharmaceuticals Plc) |
| 5154 | Beta2AdrenoceptorAgonistsSelective | 14674111000001100 | Seretide 100 Accuhaler (Sigma Pharmaceuticals Plc) |
| 5154 | Beta2AdrenoceptorAgonistsSelective | 14674711000001104 | Seretide 250 Accuhaler (Sigma Pharmaceuticals Plc) |
| 5154 | Beta2AdrenoceptorAgonistsSelective | 14705211000001107 | Seretide 500 Accuhaler (Sigma Pharmaceuticals Plc) |
| 5154 | Beta2AdrenoceptorAgonistsSelective | 14705411000001106 | Seretide 125 Evohaler (Sigma Pharmaceuticals Plc) |
| 5154 | Beta2AdrenoceptorAgonistsSelective | 14705611000001109 | Oxis 12 Turbohaler (Sigma Pharmaceuticals Plc) |
| 5154 | Beta2AdrenoceptorAgonistsSelective | 14705811000001108 | Seretide 250 Evohaler (Sigma Pharmaceuticals Plc) |
| 5154 | Beta2AdrenoceptorAgonistsSelective | 14706011000001106 | Serevent 50micrograms/dose Accuhaler (Sigma Pharmaceuticals Plc) |
| 5154 | Beta2AdrenoceptorAgonistsSelective | 14706211000001101 | Serevent 25micrograms/dose Evohaler (Sigma Pharmaceuticals Plc) |
| 5154 | Beta2AdrenoceptorAgonistsSelective | 14706311000001109 | Oxis 6 Turbohaler (Sigma Pharmaceuticals Plc) |
| 5154 | Beta2AdrenoceptorAgonistsSelective | 14750911000001104 | Ventolin 200micrograms/dose Accuhaler (Sigma Pharmaceuticals Plc) |
| 5154 | Beta2AdrenoceptorAgonistsSelective | 14759211000001101 | Volmax 8mg modified-release tablets (Sigma Pharmaceuticals Plc) |
| 5154 | Beta2AdrenoceptorAgonistsSelective | 15522411000001103 | Salipraneb 0.5mg/2.5mg nebuliser solution 2.5ml ampoules (Arrow Generics Ltd) |
| 5154 | Beta2AdrenoceptorAgonistsSelective | 15534911000001100 | Salbutamol 2.5mg/2.5ml / Ipratropium bromide 500micrograms/2.5ml nebuliser liquid ampoules |
| 5154 | Beta2AdrenoceptorAgonistsSelective | 16219211000001106 | Oxis 6 Turbohaler (Lexon (UK) Ltd) |
| 5154 | Beta2AdrenoceptorAgonistsSelective | 16219411000001105 | Oxis 12 Turbohaler (Lexon (UK) Ltd) |
| 5154 | Beta2AdrenoceptorAgonistsSelective | 16240811000001109 | Seretide 100 Accuhaler (Lexon (UK) Ltd) |
| 5154 | Beta2AdrenoceptorAgonistsSelective | 16241011000001107 | Seretide 250 Accuhaler (Lexon (UK) Ltd) |
| 5154 | Beta2AdrenoceptorAgonistsSelective | 16241111000001108 | Seretide 500 Accuhaler (Lexon (UK) Ltd) |
| 5154 | Beta2AdrenoceptorAgonistsSelective | 16241311000001105 | Seretide 125 Evohaler (Lexon (UK) Ltd) |

| ConditionID | Condition name | DMD code | Description |
|---|---|---|---|
| 5154 | Beta2AdrenoceptorAgonistsSelective | 16241511000001104 | Seretide 250 Evohaler (Lexon (UK) Ltd) |
| 5154 | Beta2AdrenoceptorAgonistsSelective | 16241711000001109 | Serevent 25micrograms/dose Evohaler (Lexon (UK) Ltd) |
| 5154 | Beta2AdrenoceptorAgonistsSelective | 16241911000001106 | Serevent 50micrograms/dose Accuhaler (Lexon (UK) Ltd) |
| 5154 | Beta2AdrenoceptorAgonistsSelective | 16545911000001103 | Foradil 12microgram inhalation powder capsules with device (Stephar (U.K.) Ltd) |
| 5154 | Beta2AdrenoceptorAgonistsSelective | 16581611000001101 | Foradil 12microgram inhalation powder capsules with device (Mawdsley-Brooks & Company Ltd) |
| 5154 | Beta2AdrenoceptorAgonistsSelective | 16585911000001109 | Oxis 12 Turbohaler (Stephar (U.K.) Ltd) |
| 5154 | Beta2AdrenoceptorAgonistsSelective | 16587911000001104 | Seretide 100 Accuhaler (Stephar (U.K.) Ltd) |
| 5154 | Beta2AdrenoceptorAgonistsSelective | 16588111000001101 | Seretide 250 Evohaler (Stephar (U.K.) Ltd) |
| 5154 | Beta2AdrenoceptorAgonistsSelective | 16728511000001101 | Seretide 50 Evohaler (Waymade Healthcare Plc) |
| 5154 | Beta2AdrenoceptorAgonistsSelective | 16733611000001107 | Salbutamol 2.5mg/2.5ml / Ipratropium bromide 500micrograms/2.5ml nebuliser liquid ampoules (A A H Pharmaceuticals Ltd) |
| 5154 | Beta2AdrenoceptorAgonistsSelective | 17026511000001100 | Salbutamol 2.5mg/2.5ml nebuliser liquid unit dose vials (Alliance Healthcare (Distribution) Ltd) |
| 5154 | Beta2AdrenoceptorAgonistsSelective | 17026711000001105 | Salbutamol 5mg/2.5ml nebuliser liquid unit dose vials (Alliance Healthcare (Distribution) Ltd) |
| 5154 | Beta2AdrenoceptorAgonistsSelective | 17299911000001102 | Onbrez Breezhaler 300microgram inhalation powder capsules with device (Novartis Pharmaceuticals UK Ltd) |
| 5154 | Beta2AdrenoceptorAgonistsSelective | 17300811000001109 | Onbrez Breezhaler 150microgram inhalation powder capsules with device (Novartis Pharmaceuticals UK Ltd) |
| 5154 | Beta2AdrenoceptorAgonistsSelective | 17313711000001107 | Indacaterol 150microgram inhalation powder capsules with device |
| 5154 | Beta2AdrenoceptorAgonistsSelective | 17313811000001104 | Indacaterol 300microgram inhalation powder capsules with device |
| 5154 | Beta2AdrenoceptorAgonistsSelective | 17344411000001108 | Oxis 6 Turbohaler (Mawdsley-Brooks & Company Ltd) |
| 5154 | Beta2AdrenoceptorAgonistsSelective | 17344611000001106 | Oxis 12 Turbohaler (Mawdsley-Brooks & Company Ltd) |
| 5154 | Beta2AdrenoceptorAgonistsSelective | 17438511000001108 | Salamol 100micrograms/dose Easi-Breathe inhaler (Mawdsley-Brooks & Company Ltd) |
| 5154 | Beta2AdrenoceptorAgonistsSelective | 17440111000001102 | Seretide 100 Accuhaler (Mawdsley-Brooks & Company Ltd) |
| 5154 | Beta2AdrenoceptorAgonistsSelective | 17440311000001100 | Seretide 250 Accuhaler (Mawdsley-Brooks & Company Ltd) |
| 5154 | Beta2AdrenoceptorAgonistsSelective | 17440511000001106 | Seretide 500 Accuhaler (Mawdsley-Brooks & Company Ltd) |
| 5154 | Beta2AdrenoceptorAgonistsSelective | 17440711000001101 | Seretide 125 Evohaler (Mawdsley-Brooks & Company Ltd) |
| 5154 | Beta2AdrenoceptorAgonistsSelective | 17440911000001104 | Seretide 250 Evohaler (Mawdsley-Brooks & Company Ltd) |
| 5154 | Beta2AdrenoceptorAgonistsSelective | 17441111000001108 | Serevent 50micrograms/dose Accuhaler (Mawdsley-Brooks & Company Ltd) |
| 5154 | Beta2AdrenoceptorAgonistsSelective | 17441311000001105 | Serevent 25micrograms/dose Evohaler (Mawdsley-Brooks & Company Ltd) |
| 5154 | Beta2AdrenoceptorAgonistsSelective | 17457311000001102 | Symbicort 100/6 Turbohaler (Mawdsley-Brooks & Company Ltd) |
| 5154 | Beta2AdrenoceptorAgonistsSelective | 17457611000001107 | Symbicort 200/6 Turbohaler (Mawdsley-Brooks & Company Ltd) |
| 5154 | Beta2AdrenoceptorAgonistsSelective | 17457811000001106 | Symbicort 400/12 Turbohaler (Mawdsley-Brooks & Company Ltd) |
| 5154 | Beta2AdrenoceptorAgonistsSelective | 17470911000001100 | Ventolin 200micrograms/dose Accuhaler (Mawdsley-Brooks & Company Ltd) |
| 5154 | Beta2AdrenoceptorAgonistsSelective | 17471111000001109 | Ventolin 100micrograms/dose Evohaler (Mawdsley-Brooks & Company Ltd) |
| 5154 | Beta2AdrenoceptorAgonistsSelective | 17471311000001106 | Ventolin 5mg Nebules (Mawdsley-Brooks & Company Ltd) |
| 5154 | Beta2AdrenoceptorAgonistsSelective | 17471511000001100 | Ventolin 2.5mg Nebules (Mawdsley-Brooks & Company Ltd) |
| 5154 | Beta2AdrenoceptorAgonistsSelective | 17495111000001101 | Bambec 10mg tablets (Mawdsley-Brooks & Company Ltd) |
| 5154 | Beta2AdrenoceptorAgonistsSelective | 17602911000001107 | Bricanyl 500micrograms/dose Turbohaler (Necessity Supplies Ltd) |
| 5154 | Beta2AdrenoceptorAgonistsSelective | 17616311000001108 | Oxis 6 Turbohaler (Necessity Supplies Ltd) |
| 5154 | Beta2AdrenoceptorAgonistsSelective | 17616911000001109 | Oxis 12 Turbohaler (Necessity Supplies Ltd) |
| 5154 | Beta2AdrenoceptorAgonistsSelective | 17661711000001102 | Terbutaline 5mg/2ml nebuliser liquid unit dose vials (Arrow Generics Ltd) |
| 5154 | Beta2AdrenoceptorAgonistsSelective | 17927011000001101 | Salbutamol 100micrograms/dose inhaler CFC free (Phoenix Healthcare Distribution Ltd) |
| 5154 | Beta2AdrenoceptorAgonistsSelective | 17927211000001106 | Salbutamol 2.5mg/2.5ml nebuliser liquid unit dose vials (Phoenix Healthcare Distribution Ltd) |

| Con ditio nID | Condition name | DMD code | Description |
|---|---|---|---|
| 5154 | Beta2AdrenoceptorAgonistsSelective | 17927411000001105 | Salbutamol 5mg/2.5ml nebuliser liquid unit dose vials (Phoenix Healthcare Distribution Ltd) |
| 5154 | Beta2AdrenoceptorAgonistsSelective | 18041311000001106 | Salbutamol 100micrograms/dose inhaler CFC free (Sandoz Ltd) |
| 5154 | Beta2AdrenoceptorAgonistsSelective | 18058411000001109 | Foradil 12microgram inhalation powder capsules with device (Lexon (UK) Ltd) |
| 5154 | Beta2AdrenoceptorAgonistsSelective | 18080711000001105 | Salbutamol 2.5mg/2.5ml nebuliser liquid unit dose vials (Fannin (UK) Ltd) |
| 5154 | Beta2AdrenoceptorAgonistsSelective | 18080911000001107 | Salbutamol 5mg/2.5ml nebuliser liquid unit dose vials (Fannin (UK) Ltd) |
| 5154 | Beta2AdrenoceptorAgonistsSelective | 18148111000001107 | Asmavent 100micrograms/dose inhaler CFC free (Kent Pharma (UK) Ltd) |
| 5154 | Beta2AdrenoceptorAgonistsSelective | 18185011000001106 | Seretide 100 Accuhaler (Necessity Supplies Ltd) |
| 5154 | Beta2AdrenoceptorAgonistsSelective | 18185211000001101 | Seretide 250 Accuhaler (Necessity Supplies Ltd) |
| 5154 | Beta2AdrenoceptorAgonistsSelective | 18185411000001102 | Seretide 500 Accuhaler (Necessity Supplies Ltd) |
| 5154 | Beta2AdrenoceptorAgonistsSelective | 18185611000001104 | Seretide 125 Evohaler (Necessity Supplies Ltd) |
| 5154 | Beta2AdrenoceptorAgonistsSelective | 18185811000001100 | Seretide 250 Evohaler (Necessity Supplies Ltd) |
| 5154 | Beta2AdrenoceptorAgonistsSelective | 18186011000001102 | Serevent 50micrograms/dose Accuhaler (Necessity Supplies Ltd) |
| 5154 | Beta2AdrenoceptorAgonistsSelective | 18186311000001104 | Serevent 25micrograms/dose Evohaler (Necessity Supplies Ltd) |
| 5154 | Beta2AdrenoceptorAgonistsSelective | 18195111000001106 | Symbicort 100/6 Turbohaler (Necessity Supplies Ltd) |
| 5154 | Beta2AdrenoceptorAgonistsSelective | 18195311000001108 | Symbicort 200/6 Turbohaler (Necessity Supplies Ltd) |
| 5154 | Beta2AdrenoceptorAgonistsSelective | 18195511000001102 | Symbicort 400/12 Turbohaler (Necessity Supplies Ltd) |
| 5154 | Beta2AdrenoceptorAgonistsSelective | 18220811000001104 | Combivent nebuliser liquid 2.5ml UDVs (Mawdsley-Brooks & Company Ltd) |
| 5154 | Beta2AdrenoceptorAgonistsSelective | 18488911000001103 | Onbrez Breezhaler 150microgram inhalation powder capsules with device (Waymade Healthcare Plc) |
| 5154 | Beta2AdrenoceptorAgonistsSelective | 18489111000001108 | Onbrez Breezhaler 300microgram inhalation powder capsules with device (Waymade Healthcare Plc) |
| 5154 | Beta2AdrenoceptorAgonistsSelective | 18610611000001106 | Salamol 100micrograms/dose Easi-Breathe inhaler (DE Pharmaceuticals) |
| 5154 | Beta2AdrenoceptorAgonistsSelective | 18611411000001104 | Seretide 500 Accuhaler (DE Pharmaceuticals) |
| 5154 | Beta2AdrenoceptorAgonistsSelective | 18611611000001101 | Serevent 25micrograms/dose Evohaler (DE Pharmaceuticals) |
| 5154 | Beta2AdrenoceptorAgonistsSelective | 19541511000001101 | Combivent nebuliser liquid 2.5ml UDVs (DE Pharmaceuticals) |
| 5154 | Beta2AdrenoceptorAgonistsSelective | 19562111000001107 | Neovent 25micrograms/dose inhaler CFC free (Teva UK Ltd) |
| 5154 | Beta2AdrenoceptorAgonistsSelective | 19568411000001100 | Neovent 25micrograms/dose inhaler CFC free (Kent Pharma (UK) Ltd) |
| 5154 | Beta2AdrenoceptorAgonistsSelective | 19869411000001109 | Combivent nebuliser liquid 2.5ml UDVs (Lexon (UK) Ltd) |
| 5154 | Beta2AdrenoceptorAgonistsSelective | 20031011000001108 | Ventolin 200micrograms/dose Accuhaler (Lexon (UK) Ltd) |
| 5154 | Beta2AdrenoceptorAgonistsSelective | 20434411000001107 | Salamol 100micrograms/dose inhaler CFC free (Arrow Generics Ltd) |
| 5154 | Beta2AdrenoceptorAgonistsSelective | 20638911000001108 | Salmeterol 25micrograms/dose inhaler CFC free (A A H Pharmaceuticals Ltd) |
| 5154 | Beta2AdrenoceptorAgonistsSelective | 21019411000001101 | Flutiform 125micrograms/dose / 5micrograms/dose inhaler (Napp Pharmaceuticals Ltd) |
| 5154 | Beta2AdrenoceptorAgonistsSelective | 21019711000001107 | Flutiform 250micrograms/dose / 10micrograms/dose inhaler (Napp Pharmaceuticals Ltd) |
| 5154 | Beta2AdrenoceptorAgonistsSelective | 21020611000001104 | Flutiform 50micrograms/dose / 5micrograms/dose inhaler (Napp Pharmaceuticals Ltd) |
| 5154 | Beta2AdrenoceptorAgonistsSelective | 21113711000001102 | Fluticasone 125micrograms/dose / Formoterol 5micrograms/dose inhaler CFC free |
| 5154 | Beta2AdrenoceptorAgonistsSelective | 21113811000001105 | Fluticasone 250micrograms/dose / Formoterol 10micrograms/dose inhaler CFC free |
| 5154 | Beta2AdrenoceptorAgonistsSelective | 21113911000001100 | Fluticasone 50micrograms/dose / Formoterol 5micrograms/dose inhaler CFC free |
| 5154 | Beta2AdrenoceptorAgonistsSelective | 21787311000001103 | Terbutaline 5mg/2ml nebuliser liquid unit dose vials (Waymade Healthcare Plc) |
| 5154 | Beta2AdrenoceptorAgonistsSelective | 21866011000001104 | Foradil 12microgram inhalation powder capsules with device (Waymade Healthcare Plc) |
| 5154 | Beta2AdrenoceptorAgonistsSelective | 21895011000001105 | Salbutamol 100micrograms/dose inhaler CFC free (Waymade Healthcare Plc) |
| 5154 | Beta2AdrenoceptorAgonistsSelective | 21895211000001100 | Salbutamol 2mg tablets (Waymade Healthcare Plc) |
| 5154 | Beta2AdrenoceptorAgonistsSelective | 21895411000001101 | Salbutamol 4mg tablets (Waymade Healthcare Plc) |

| Con ditio nID | Condition name | DMD code | Description |
|---|---|---|---|
| 5154 | Beta2AdrenoceptorAgonistsSelective | 22354211000001108 | Salbutamol 100micrograms/dose inhaler CFC free (AM Distributions (Yorkshire) Ltd) |
| 5154 | Beta2AdrenoceptorAgonistsSelective | 22355111000001103 | Terbutaline 5mg/2ml nebuliser liquid unit dose vials (AM Distributions (Yorkshire) Ltd) |
| 5154 | Beta2AdrenoceptorAgonistsSelective | 22503111000001109 | AirSalb 100micrograms/dose inhaler CFC free (Sandoz Ltd) |
| 5154 | Beta2AdrenoceptorAgonistsSelective | 22650111000001100 | Vertine 25micrograms/dose inhaler CFC free (Teva UK Ltd) |
| 5154 | Beta2AdrenoceptorAgonistsSelective | 23433411000001108 | Salbutamol 2.5mg/2.5ml / Ipratropium bromide 500micrograms/2.5ml nebuliser liquid unit dose vials (J M McGill Ltd) |
| 5154 | Beta2AdrenoceptorAgonistsSelective | 23621711000001102 | Relvar Ellipta 184micrograms/dose / 22micrograms/dose dry powder inhaler (GlaxoSmithKline UK Ltd) |
| 5154 | Beta2AdrenoceptorAgonistsSelective | 23622011000001107 | Relvar Ellipta 92micrograms/dose / 22micrograms/dose dry powder inhaler (GlaxoSmithKline UK Ltd) |
| 5154 | Beta2AdrenoceptorAgonistsSelective | 23661311000001105 | Fluticasone furoate 184micrograms/dose / Vilanterol 22micrograms/dose dry powder inhaler |
| 5154 | Beta2AdrenoceptorAgonistsSelective | 23661411000001103 | Fluticasone furoate 92micrograms/dose / Vilanterol 22micrograms/dose dry powder inhaler |
| 5154 | Beta2AdrenoceptorAgonistsSelective | 23961011000001108 | Salbutamol 2.5mg/2.5ml / Ipratropium bromide 500micrograms/2.5ml nebuliser liquid unit dose vials (Niche Pharma Ltd) |
| 5154 | Beta2AdrenoceptorAgonistsSelective | 24608011000001103 | Striverdi Respimat 2.5micrograms/dose inhalation solution cartridge with device (Boehringer Ingelheim Ltd) |
| 5154 | Beta2AdrenoceptorAgonistsSelective | 24644611000001108 | Anoro Ellipta 55micrograms/dose / 22micrograms/dose dry powder inhaler (GlaxoSmithKline UK Ltd) |
| 5154 | Beta2AdrenoceptorAgonistsSelective | 24645511000001105 | Umeclidinium bromide 65micrograms/dose / Vilanterol 22micrograms/dose dry powder inhaler |
| 5154 | Beta2AdrenoceptorAgonistsSelective | 24670111000001108 | Olodaterol 2.5micrograms/dose solution for inhalation cartridge with device CFC free |
| 5154 | Beta2AdrenoceptorAgonistsSelective | 25254111000001105 | DuoResp Spiromax 160micrograms/dose / 4.5micrograms/dose dry powder inhaler (Teva UK Ltd) |
| 5154 | Beta2AdrenoceptorAgonistsSelective | 25254711000001106 | DuoResp Spiromax 320micrograms/dose / 9micrograms/dose dry powder inhaler (Teva UK Ltd) |
| 5154 | Beta2AdrenoceptorAgonistsSelective | 26112111000001106 | Fostair NEXThaler 100micrograms/dose / 6micrograms/dose dry powder inhaler (Chiesi Ltd) |
| 5154 | Beta2AdrenoceptorAgonistsSelective | 26148711000001101 | Beclometasone 100micrograms/dose / Formoterol 6micrograms/dose dry powder inhaler |
| 5154 | Beta2AdrenoceptorAgonistsSelective | 28007211000001102 | Ultibro Breezhaler 85microgram/43microgram inhalation powder capsules with device (Novartis Pharmaceuticals UK Ltd) |
| 5154 | Beta2AdrenoceptorAgonistsSelective | 28049611000001104 | Indacaterol 85micrograms/dose / Glycopyrronium bromide 54micrograms/dose inhalation powder capsules with device |
| 5154 | Beta2AdrenoceptorAgonistsSelective | 28194211000001100 | Terbutaline 7.5mg/15ml solution for infusion pre-filled syringes (Special Order) |
| 5154 | Beta2AdrenoceptorAgonistsSelective | 28279711000001100 | Terbutaline 7.5mg/15ml solution for infusion pre-filled syringes |
| 5154 | Beta2AdrenoceptorAgonistsSelective | 28357211000001106 | Duaklir 340micrograms/dose / 12micrograms/dose Genuair (AstraZeneca UK Ltd) |
| 5154 | Beta2AdrenoceptorAgonistsSelective | 28365011000001100 | Aclidinium bromide 396micrograms/dose / Formoterol 11.8micrograms/dose dry powder inhaler |
| 5154 | Beta2AdrenoceptorAgonistsSelective | 28410811000001107 | Berodual N inhaler (Imported (Germany)) |
| 5154 | Beta2AdrenoceptorAgonistsSelective | 28422511000001101 | Fenoterol 50micrograms/dose / Ipratropium bromide 20micrograms/dose inhaler CFC free |
| 5154 | Beta2AdrenoceptorAgonistsSelective | 29211411000001103 | Salbutamol 2.5mg/2.5ml / Ipratropium bromide 500micrograms/2.5ml nebuliser liquid unit dose vials (Ennogen Healthcare Ltd) |
| 5154 | Beta2AdrenoceptorAgonistsSelective | 29749211000001101 | Flutiform 125micrograms/dose / 5micrograms/dose inhaler (Waymade Healthcare Plc) |
| 5154 | Beta2AdrenoceptorAgonistsSelective | 29749411000001102 | Flutiform 50micrograms/dose / 5micrograms/dose inhaler (Waymade Healthcare Plc) |
| 5154 | Beta2AdrenoceptorAgonistsSelective | 29749611000001104 | Flutiform 250micrograms/dose / 10micrograms/dose inhaler (Waymade Healthcare Plc) |
| 5154 | Beta2AdrenoceptorAgonistsSelective | 29782111000001107 | Sirdupla 25micrograms/dose / 125micrograms/dose inhaler (Viatris UK Healthcare Ltd) |
| 5154 | Beta2AdrenoceptorAgonistsSelective | 29782511000001103 | Sirdupla 25micrograms/dose / 250micrograms/dose inhaler (Viatris UK Healthcare Ltd) |
| 5154 | Beta2AdrenoceptorAgonistsSelective | 29971311000001100 | Spiolto Respimat 2.5micrograms/dose / 2.5micrograms/dose inhalation solution cartridge with device (Boehringer Ingelheim Ltd) |
| 5154 | Beta2AdrenoceptorAgonistsSelective | 29980011000001104 | Bricanyl 500micrograms/dose Turbohaler (Lexon (UK) Ltd) |
| 5154 | Beta2AdrenoceptorAgonistsSelective | 29987211000001108 | Tiotropium bromide 2.5micrograms/dose / Olodaterol 2.5micrograms/dose solution for inhalation cartridge with device CFC free |
| 5154 | Beta2AdrenoceptorAgonistsSelective | 30041511000001100 | Atimos Modulite 12micrograms/dose inhaler (Waymade Healthcare Plc) |

| Con ditio nID | Condition name | DMD code | Description |
|---|---|---|---|
| 5154 | Beta2AdrenoceptorAgonistsSelective | 30094711000001100 | Salbutamol 100micrograms/dose inhaler CFC free (DE Pharmaceuticals) |
| 5154 | Beta2AdrenoceptorAgonistsSelective | 30094911000001103 | Salbutamol 2mg tablets (DE Pharmaceuticals) |
| 5154 | Beta2AdrenoceptorAgonistsSelective | 30095111000001102 | Salbutamol 4mg tablets (DE Pharmaceuticals) |
| 5154 | Beta2AdrenoceptorAgonistsSelective | 30095311000001100 | Salmeterol 25micrograms/dose inhaler CFC free (DE Pharmaceuticals) |
| 5154 | Beta2AdrenoceptorAgonistsSelective | 30253911000001100 | Sirdupla 25micrograms/dose / 250micrograms/dose inhaler (Waymade Healthcare Plc) |
| 5154 | Beta2AdrenoceptorAgonistsSelective | 30273011000001101 | Relvar Ellipta 184micrograms/dose / 22micrograms/dose dry powder inhaler (Waymade Healthcare Plc) |
| 5154 | Beta2AdrenoceptorAgonistsSelective | 30273211000001106 | Relvar Ellipta 92micrograms/dose / 22micrograms/dose dry powder inhaler (Waymade Healthcare Plc) |
| 5154 | Beta2AdrenoceptorAgonistsSelective | 30891711000001102 | Salbutamol 100micrograms/dose inhaler CFC free (Mawdsley-Brooks & Company Ltd) |
| 5154 | Beta2AdrenoceptorAgonistsSelective | 30891911000001100 | Salbutamol 2.5mg/2.5ml nebuliser liquid unit dose vials (Mawdsley-Brooks & Company Ltd) |
| 5154 | Beta2AdrenoceptorAgonistsSelective | 30892111000001108 | Salbutamol 5mg/2.5ml nebuliser liquid unit dose vials (Mawdsley-Brooks & Company Ltd) |
| 5154 | Beta2AdrenoceptorAgonistsSelective | 30924211000001107 | Terbutaline 5mg/2ml nebuliser liquid unit dose vials (Mawdsley-Brooks & Company Ltd) |
| 5154 | Beta2AdrenoceptorAgonistsSelective | 30950311000001106 | AirFluSal Forspiro 50micrograms/dose / 500micrograms/dose dry powder inhaler (Sandoz Ltd) |
| 5154 | Beta2AdrenoceptorAgonistsSelective | 31063111000001106 | Fostair NEXThaler 200micrograms/dose / 6micrograms/dose dry powder inhaler (Chiesi Ltd) |
| 5154 | Beta2AdrenoceptorAgonistsSelective | 31063411000001101 | Fostair 200micrograms/dose / 6micrograms/dose inhaler (Chiesi Ltd) |
| 5154 | Beta2AdrenoceptorAgonistsSelective | 31064411000001103 | Fluticasone 125micrograms/dose / Salmeterol 25micrograms/dose inhaler CFC free (A A H Pharmaceuticals Ltd) |
| 5154 | Beta2AdrenoceptorAgonistsSelective | 31064611000001100 | Fluticasone 250micrograms/dose / Salmeterol 25micrograms/dose inhaler CFC free (A A H Pharmaceuticals Ltd) |
| 5154 | Beta2AdrenoceptorAgonistsSelective | 31064811000001101 | Fluticasone propionate 500micrograms/dose / Salmeterol 50micrograms/dose dry powder inhaler (A A H Pharmaceuticals Ltd) |
| 5154 | Beta2AdrenoceptorAgonistsSelective | 31087411000001106 | Beclometasone 200micrograms/dose / Formoterol 6micrograms/dose inhaler CFC free |
| 5154 | Beta2AdrenoceptorAgonistsSelective | 31087511000001105 | Beclometasone 200micrograms/dose / Formoterol 6micrograms/dose dry powder inhaler |
| 5154 | Beta2AdrenoceptorAgonistsSelective | 31457011000001100 | Fluticasone furoate 184micrograms/dose / Vilanterol 22micrograms/dose dry powder inhaler (J M McGill Ltd) |
| 5154 | Beta2AdrenoceptorAgonistsSelective | 31457411000001109 | Fluticasone furoate 92micrograms/dose / Vilanterol 22micrograms/dose dry powder inhaler (J M McGill Ltd) |
| 5154 | Beta2AdrenoceptorAgonistsSelective | 32333511000001106 | Fluticasone furoate 184micrograms/dose / Vilanterol 22micrograms/dose dry powder inhaler (Colorama Pharmaceuticals Ltd) |
| 5154 | Beta2AdrenoceptorAgonistsSelective | 32333911000001104 | Fluticasone furoate 92micrograms/dose / Vilanterol 22micrograms/dose dry powder inhaler (Colorama Pharmaceuticals Ltd) |
| 5154 | Beta2AdrenoceptorAgonistsSelective | 32364511000001104 | Umeclidinium bromide 65micrograms/dose / Vilanterol 22micrograms/dose dry powder inhaler (Ennogen Healthcare Ltd) |
| 5154 | Beta2AdrenoceptorAgonistsSelective | 32480911000001107 | Salbutamol 2.5mg/2.5ml / Ipratropium bromide 500micrograms/2.5ml nebuliser liquid unit dose vials (Colorama Pharmaceuticals Ltd) |
| 5154 | Beta2AdrenoceptorAgonistsSelective | 32629811000001101 | Umeclidinium bromide 65micrograms/dose / Vilanterol 22micrograms/dose dry powder inhaler (J M McGill Ltd) |
| 5154 | Beta2AdrenoceptorAgonistsSelective | 32633011000001106 | Aclidinium bromide 396micrograms/dose / Formoterol 11.8micrograms/dose dry powder inhaler (Colorama Pharmaceuticals Ltd) |
| 5154 | Beta2AdrenoceptorAgonistsSelective | 32684611000001106 | Fluticasone furoate 184micrograms/dose / Vilanterol 22micrograms/dose dry powder inhaler (DE Pharmaceuticals) |
| 5154 | Beta2AdrenoceptorAgonistsSelective | 32684911000001100 | Fluticasone furoate 92micrograms/dose / Vilanterol 22micrograms/dose dry powder inhaler (DE Pharmaceuticals) |
| 5154 | Beta2AdrenoceptorAgonistsSelective | 32898611000001109 | Salipraneb 0.5mg/2.5mg nebuliser solution 2.5ml ampoules (Actavis UK Ltd) |
| 5154 | Beta2AdrenoceptorAgonistsSelective | 32926011000001100 | Symbicort 200micrograms/dose / 6micrograms/dose pressurised inhaler (AstraZeneca UK Ltd) |
| 5154 | Beta2AdrenoceptorAgonistsSelective | 32960711000001105 | Budesonide 200micrograms/dose / Formoterol 6micrograms/dose inhaler CFC free |
| 5154 | Beta2AdrenoceptorAgonistsSelective | 33561211000001109 | Soltel 25micrograms/dose inhaler CFC free (Cipla EU Ltd) |
| 5154 | Beta2AdrenoceptorAgonistsSelective | 33679711000001103 | Aerivio Spiromax 50micrograms/dose / 500micrograms/dose dry powder inhaler (Teva UK Ltd) |
| 5154 | Beta2AdrenoceptorAgonistsSelective | 34023611000001101 | Sereflo 25micrograms/dose / 125micrograms/dose inhaler (Cipla EU Ltd) |
| 5154 | Beta2AdrenoceptorAgonistsSelective | 34023811000001102 | Sereflo 25micrograms/dose / 250micrograms/dose inhaler (Cipla EU Ltd) |

| Con ditio nID | Condition name | DMD code | Description |
|---|---|---|---|
| 5154 | Beta2AdrenoceptorAgonistsSelective | 34215311000001107 | AirFluSal 25micrograms/dose / 125micrograms/dose inhaler (Sandoz Ltd) |
| 5154 | Beta2AdrenoceptorAgonistsSelective | 34215511000001101 | AirFluSal 25micrograms/dose / 250micrograms/dose inhaler (Sandoz Ltd) |
| 5154 | Beta2AdrenoceptorAgonistsSelective | 34675711000001103 | Aloflute 25micrograms/dose / 250micrograms/dose inhaler (Viatris UK Healthcare Ltd) |
| 5154 | Beta2AdrenoceptorAgonistsSelective | 34677011000001107 | Aloflute 25micrograms/dose / 125micrograms/dose inhaler (Viatris UK Healthcare Ltd) |
| 5154 | Beta2AdrenoceptorAgonistsSelective | 34681611000001100 | Trimbow 87micrograms/dose / 5micrograms/dose / 9micrograms/dose inhaler (Chiesi Ltd) |
| 5154 | Beta2AdrenoceptorAgonistsSelective | 34683311000001106 | Generic Trimbow 87micrograms/dose / 5micrograms/dose / 9micrograms/dose inhaler |
| 5154 | Beta2AdrenoceptorAgonistsSelective | 34812111000001106 | Fobumix Easyhaler 320micrograms/dose / 9micrograms/dose dry powder inhaler (Orion Pharma (UK) Ltd) |
| 5154 | Beta2AdrenoceptorAgonistsSelective | 34950311000001108 | Fobumix Easyhaler 160micrograms/dose / 4.5micrograms/dose dry powder inhaler (Orion Pharma (UK) Ltd) |
| 5154 | Beta2AdrenoceptorAgonistsSelective | 34950611000001103 | Fobumix Easyhaler 80micrograms/dose / 4.5micrograms/dose dry powder inhaler (Orion Pharma (UK) Ltd) |
| 5154 | Beta2AdrenoceptorAgonistsSelective | 34952211000001104 | Trelegy Ellipta 92micrograms/dose / 55micrograms/dose / 22micrograms/dose dry powder inhaler (GlaxoSmithKline UK Ltd) |
| 5154 | Beta2AdrenoceptorAgonistsSelective | 34955111000001103 | Generic Trelegy Ellipta 92micrograms/dose / 55micrograms/dose / 22micrograms/dose dry powder inhaler |
| 5154 | Beta2AdrenoceptorAgonistsSelective | 35369511000001103 | Salbutamol 200microgram / Beclometasone 100microgram inhalation powder capsules |
| 5154 | Beta2AdrenoceptorAgonistsSelective | 35515311000001106 | Fusacomb Easyhaler 50micrograms/dose / 500micrograms/dose dry powder inhaler (Orion Pharma (UK) Ltd) |
| 5154 | Beta2AdrenoceptorAgonistsSelective | 35515511000001100 | Fusacomb Easyhaler 50micrograms/dose / 250micrograms/dose dry powder inhaler (Orion Pharma (UK) Ltd) |
| 5154 | Beta2AdrenoceptorAgonistsSelective | 35594011000001105 | Combisal 25micrograms/dose / 50micrograms/dose inhaler (Aspire Pharma Ltd) |
| 5154 | Beta2AdrenoceptorAgonistsSelective | 35594211000001100 | Combisal 25micrograms/dose / 125micrograms/dose inhaler (Aspire Pharma Ltd) |
| 5154 | Beta2AdrenoceptorAgonistsSelective | 35594411000001101 | Combisal 25micrograms/dose / 250micrograms/dose inhaler (Aspire Pharma Ltd) |
| 5154 | Beta2AdrenoceptorAgonistsSelective | 35647311000001101 | Flutiform K-haler 125micrograms/dose / 5micrograms/dose breath actuated inhaler (Napp Pharmaceuticals Ltd) |
| 5154 | Beta2AdrenoceptorAgonistsSelective | 35647511000001107 | Fluticasone 125micrograms/dose / Formoterol 5micrograms/dose breath actuated inhaler CFC free |
| 5154 | Beta2AdrenoceptorAgonistsSelective | 35647611000001106 | Fluticasone 50micrograms/dose / Formoterol 5micrograms/dose breath actuated inhaler CFC free |
| 5154 | Beta2AdrenoceptorAgonistsSelective | 35650811000001109 | Flutiform K-haler 50micrograms/dose / 5micrograms/dose breath actuated inhaler (Napp Pharmaceuticals Ltd) |
| 5154 | Beta2AdrenoceptorAgonistsSelective | 35912011000001109 | Budesonide 100micrograms/dose / Formoterol 6micrograms/dose dry powder inhaler |
| 5154 | Beta2AdrenoceptorAgonistsSelective | 35915911000001100 | Terbutaline 1.5mg/5ml oral solution sugar free |
| 5154 | Beta2AdrenoceptorAgonistsSelective | 35916011000001108 | Terbutaline 2.5mg/5ml solution for injection ampoules |
| 5154 | Beta2AdrenoceptorAgonistsSelective | 35916111000001109 | Terbutaline 250micrograms/dose inhaler with spacer |
| 5154 | Beta2AdrenoceptorAgonistsSelective | 35916211000001103 | Terbutaline 500micrograms/1ml solution for injection ampoules |
| 5154 | Beta2AdrenoceptorAgonistsSelective | 35916311000001106 | Terbutaline 500micrograms/dose dry powder inhaler |
| 5154 | Beta2AdrenoceptorAgonistsSelective | 35916411000001104 | Terbutaline 7.5mg modified-release tablets |
| 5154 | Beta2AdrenoceptorAgonistsSelective | 35926311000001100 | Sodium cromoglicate 1mg/dose / Salbutamol 100micrograms/dose inhaler |
| 5154 | Beta2AdrenoceptorAgonistsSelective | 35926411000001107 | Sodium cromoglicate 1mg/dose / Salbutamol 100micrograms/dose inhaler with spacer |
| 5154 | Beta2AdrenoceptorAgonistsSelective | 35936211000001105 | Salbutamol 100micrograms/dose / Beclometasone 50micrograms/dose inhaler |
| 5154 | Beta2AdrenoceptorAgonistsSelective | 35936311000001102 | Salbutamol 100micrograms/dose / Ipratropium 20micrograms/dose inhaler |
| 5154 | Beta2AdrenoceptorAgonistsSelective | 35936411000001109 | Salbutamol 100micrograms/dose breath actuated inhaler |
| 5154 | Beta2AdrenoceptorAgonistsSelective | 35936511000001108 | Salbutamol 100micrograms/dose inhaler |
| 5154 | Beta2AdrenoceptorAgonistsSelective | 35936611000001107 | Salbutamol 200microgram inhalation powder blisters |
| 5154 | Beta2AdrenoceptorAgonistsSelective | 35936711000001103 | Salbutamol 200microgram inhalation powder blisters with device |
| 5154 | Beta2AdrenoceptorAgonistsSelective | 35936811000001106 | Salbutamol 400microgram inhalation powder blisters |
| 5154 | Beta2AdrenoceptorAgonistsSelective | 35936911000001101 | Salbutamol 400microgram inhalation powder blisters with device |

| Con ditio nID | Condition name | DMD code | Description |
|---|---|---|---|
| 5154 | Beta2AdrenoceptorAgonistsSelective | 35937011000001102 | Salbutamol 5mg/50ml solution for infusion vials |
| 5154 | Beta2AdrenoceptorAgonistsSelective | 35937111000001101 | Salbutamol 8mg modified-release tablets |
| 5154 | Beta2AdrenoceptorAgonistsSelective | 35937211000001107 | Salbutamol 95micrograms/dose dry powder inhaler |
| 5154 | Beta2AdrenoceptorAgonistsSelective | 35937811000001108 | Salmeterol 50microgram inhalation powder blisters with device |
| 5154 | Beta2AdrenoceptorAgonistsSelective | 35938011000001101 | Salmeterol 50microgram inhalation powder blisters |
| 5154 | Beta2AdrenoceptorAgonistsSelective | 36066611000001106 | Fenoterol 100micrograms/dose / Ipratropium 40micrograms/dose inhaler |
| 5154 | Beta2AdrenoceptorAgonistsSelective | 36066711000001102 | Fenoterol 100micrograms/dose / Ipratropium bromide 40micrograms/dose breath actuated inhaler |
| 5154 | Beta2AdrenoceptorAgonistsSelective | 36604711000001102 | Stalpex 50micrograms/dose / 500micrograms/dose dry powder inhaler (Glenmark Pharmaceuticals Europe Ltd) |
| 5154 | Beta2AdrenoceptorAgonistsSelective | 36889311000001107 | DuoResp Spiromax 160micrograms/dose / 4.5micrograms/dose dry powder inhaler (Pharmaram Ltd) |
| 5154 | Beta2AdrenoceptorAgonistsSelective | 36889511000001101 | DuoResp Spiromax 320micrograms/dose / 9micrograms/dose dry powder inhaler (Pharmaram Ltd) |
| 5154 | Beta2AdrenoceptorAgonistsSelective | 36895211000001101 | Serevent 50micrograms/dose Accuhaler (Originalis B.V.) |
| 5154 | Beta2AdrenoceptorAgonistsSelective | 37350511000001103 | Bricanyl 500micrograms/dose Turbohaler (Mawdsley-Brooks & Company Ltd) |
| 5154 | Beta2AdrenoceptorAgonistsSelective | 37364411000001100 | Combivent nebuliser liquid 2.5ml UDVs (CST Pharma Ltd) |
| 5154 | Beta2AdrenoceptorAgonistsSelective | 37391711000001105 | Flutiform 125micrograms/dose / 5micrograms/dose inhaler (CST Pharma Ltd) |
| 5154 | Beta2AdrenoceptorAgonistsSelective | 37395311000001108 | Salamol 100micrograms/dose Easi-Breathe inhaler (CST Pharma Ltd) |
| 5154 | Beta2AdrenoceptorAgonistsSelective | 37397211000001108 | DuoResp Spiromax 320micrograms/dose / 9micrograms/dose dry powder inhaler (Mawdsley-Brooks & Company Ltd) |
| 5154 | Beta2AdrenoceptorAgonistsSelective | 37397411000001107 | DuoResp Spiromax 160micrograms/dose / 4.5micrograms/dose dry powder inhaler (Mawdsley-Brooks & Company Ltd) |
| 5154 | Beta2AdrenoceptorAgonistsSelective | 37415511000001109 | Serevent 50micrograms/dose Accuhaler (CST Pharma Ltd) |
| 5154 | Beta2AdrenoceptorAgonistsSelective | 37434411000001103 | DuoResp Spiromax 160micrograms/dose / 4.5micrograms/dose dry powder inhaler (CST Pharma Ltd) |
| 5154 | Beta2AdrenoceptorAgonistsSelective | 37434611000001100 | DuoResp Spiromax 320micrograms/dose / 9micrograms/dose dry powder inhaler (CST Pharma Ltd) |
| 5154 | Beta2AdrenoceptorAgonistsSelective | 37443211000001101 | Seretide 250 Accuhaler (CST Pharma Ltd) |
| 5154 | Beta2AdrenoceptorAgonistsSelective | 37443411000001102 | Seretide 500 Accuhaler (CST Pharma Ltd) |
| 5154 | Beta2AdrenoceptorAgonistsSelective | 37443611000001104 | Seretide 250 Evohaler (CST Pharma Ltd) |
| 5154 | Beta2AdrenoceptorAgonistsSelective | 37443811000001100 | Oxis 6 Turbohaler (CST Pharma Ltd) |
| 5154 | Beta2AdrenoceptorAgonistsSelective | 37444011000001108 | Seretide 125 Evohaler (CST Pharma Ltd) |
| 5154 | Beta2AdrenoceptorAgonistsSelective | 37444211000001103 | Symbicort 400/12 Turbohaler (CST Pharma Ltd) |
| 5154 | Beta2AdrenoceptorAgonistsSelective | 37448711000001102 | Flutiform 250micrograms/dose / 10micrograms/dose inhaler (CST Pharma Ltd) |
| 5154 | Beta2AdrenoceptorAgonistsSelective | 37484711000001101 | Flutiform 250micrograms/dose / 10micrograms/dose inhaler (Mawdsley-Brooks & Company Ltd) |
| 5154 | Beta2AdrenoceptorAgonistsSelective | 37484911000001104 | Flutiform 125micrograms/dose / 5micrograms/dose inhaler (Mawdsley-Brooks & Company Ltd) |
| 5154 | Beta2AdrenoceptorAgonistsSelective | 37526111000001109 | Onbrez Breezhaler 150microgram inhalation powder capsules with device (CST Pharma Ltd) |
| 5154 | Beta2AdrenoceptorAgonistsSelective | 37526611000001101 | Onbrez Breezhaler 300microgram inhalation powder capsules with device (CST Pharma Ltd) |
| 5154 | Beta2AdrenoceptorAgonistsSelective | 37532911000001106 | Relvar Ellipta 184micrograms/dose / 22micrograms/dose dry powder inhaler (CST Pharma Ltd) |
| 5154 | Beta2AdrenoceptorAgonistsSelective | 37533111000001102 | Relvar Ellipta 92micrograms/dose / 22micrograms/dose dry powder inhaler (CST Pharma Ltd) |
| 5154 | Beta2AdrenoceptorAgonistsSelective | 37540311000001105 | Yanimo Respimat 2.5micrograms/dose / 2.5micrograms/dose inhalation solution cartridge with device (Boehringer Ingelheim Ltd) |
| 5154 | Beta2AdrenoceptorAgonistsSelective | 37549211000001109 | Onbrez Breezhaler 150microgram inhalation powder capsules with device (Mawdsley-Brooks & Company Ltd) |
| 5154 | Beta2AdrenoceptorAgonistsSelective | 37574711000001102 | Foradil 12microgram inhalation powder capsules with device (CST Pharma Ltd) |
| 5154 | Beta2AdrenoceptorAgonistsSelective | 37575111000001104 | Oxis 12 Turbohaler (CST Pharma Ltd) |
| 5154 | Beta2AdrenoceptorAgonistsSelective | 37576711000001106 | Bricanyl 500micrograms/dose Turbohaler (CST Pharma Ltd) |

| Con ditio nID | Condition name | DMD code | Description |
|---|---|---|---|
| 5154 | Beta2AdrenoceptorAgonistsSelective | 37625611000001100 | Combivent nebuliser liquid 2.5ml UDVs (Pilsco Ltd) |
| 5154 | Beta2AdrenoceptorAgonistsSelective | 37631511000001104 | DuoResp Spiromax 160micrograms/dose / 4.5micrograms/dose dry powder inhaler (Ethigen Ltd) |
| 5154 | Beta2AdrenoceptorAgonistsSelective | 37631711000001109 | DuoResp Spiromax 320micrograms/dose / 9micrograms/dose dry powder inhaler (Ethigen Ltd) |
| 5154 | Beta2AdrenoceptorAgonistsSelective | 37665111000001104 | Flutiform 125micrograms/dose / 5micrograms/dose inhaler (Pilsco Ltd) |
| 5154 | Beta2AdrenoceptorAgonistsSelective | 37665311000001102 | Flutiform 250micrograms/dose / 10micrograms/dose inhaler (Pilsco Ltd) |
| 5154 | Beta2AdrenoceptorAgonistsSelective | 37678011000001103 | Spiolto Respimat 2.5micrograms/dose / 2.5micrograms/dose inhalation solution refill cartridge (Boehringer Ingelheim Ltd) |
| 5154 | Beta2AdrenoceptorAgonistsSelective | 37678311000001100 | Striverdi Respimat 2.5micrograms/dose inhalation solution refill cartridge (Boehringer Ingelheim Ltd) |
| 5154 | Beta2AdrenoceptorAgonistsSelective | 37692311000001106 | Olodaterol 2.5micrograms/dose inhalation solution cartridge CFC free |
| 5154 | Beta2AdrenoceptorAgonistsSelective | 37692511000001100 | Tiotropium bromide 2.5micrograms/dose / Olodaterol 2.5micrograms/dose inhalation solution cartridge CFC free |
| 5154 | Beta2AdrenoceptorAgonistsSelective | 37700511000001108 | Relvar Ellipta 184micrograms/dose / 22micrograms/dose dry powder inhaler (Pilsco Ltd) |
| 5154 | Beta2AdrenoceptorAgonistsSelective | 37700711000001103 | Relvar Ellipta 92micrograms/dose / 22micrograms/dose dry powder inhaler (Pilsco Ltd) |
| 5154 | Beta2AdrenoceptorAgonistsSelective | 37701911000001108 | Salamol 100micrograms/dose Easi-Breathe inhaler (Pilsco Ltd) |
| 5154 | Beta2AdrenoceptorAgonistsSelective | 37702211000001106 | Seretide 250 Accuhaler (Pilsco Ltd) |
| 5154 | Beta2AdrenoceptorAgonistsSelective | 37702411000001105 | Seretide 250 Evohaler (Pilsco Ltd) |
| 5154 | Beta2AdrenoceptorAgonistsSelective | 37702611000001108 | Seretide 500 Accuhaler (Pilsco Ltd) |
| 5154 | Beta2AdrenoceptorAgonistsSelective | 37702811000001107 | Serevent 50micrograms/dose Accuhaler (Pilsco Ltd) |
| 5154 | Beta2AdrenoceptorAgonistsSelective | 37714411000001100 | AirFluSal Forspiro 50micrograms/dose / 500micrograms/dose dry powder inhaler (Pilsco Ltd) |
| 5154 | Beta2AdrenoceptorAgonistsSelective | 37714611000001102 | AirFluSal 25micrograms/dose / 125micrograms/dose inhaler (Pilsco Ltd) |
| 5154 | Beta2AdrenoceptorAgonistsSelective | 37714811000001103 | AirFluSal 25micrograms/dose / 250micrograms/dose inhaler (Pilsco Ltd) |
| 5154 | Beta2AdrenoceptorAgonistsSelective | 37715611000001101 | Atimos Modulite 12micrograms/dose inhaler (Pilsco Ltd) |
| 5154 | Beta2AdrenoceptorAgonistsSelective | 37729411000001107 | Sereflo 25micrograms/dose / 250micrograms/dose inhaler (Pilsco Ltd) |
| 5154 | Beta2AdrenoceptorAgonistsSelective | 37739011000001101 | Symbicort 200/6 Turbohaler (CST Pharma Ltd) |
| 5154 | Beta2AdrenoceptorAgonistsSelective | 37979411000001103 | Anoro Ellipta 55micrograms/dose / 22micrograms/dose dry powder inhaler (Pharmaram Ltd) |
| 5154 | Beta2AdrenoceptorAgonistsSelective | 37994011000001101 | Relvar Ellipta 184micrograms/dose / 22micrograms/dose dry powder inhaler (Pharmaram Ltd) |
| 5154 | Beta2AdrenoceptorAgonistsSelective | 37994211000001106 | Relvar Ellipta 92micrograms/dose / 22micrograms/dose dry powder inhaler (Pharmaram Ltd) |
| 5154 | Beta2AdrenoceptorAgonistsSelective | 37996511000001103 | Seretide 125 Evohaler (Pharmaram Ltd) |
| 5154 | Beta2AdrenoceptorAgonistsSelective | 37996711000001108 | Seretide 250 Evohaler (Pharmaram Ltd) |
| 5154 | Beta2AdrenoceptorAgonistsSelective | 37997311000001107 | Symbicort 100/6 Turbohaler (Pharmaram Ltd) |
| 5154 | Beta2AdrenoceptorAgonistsSelective | 37997511000001101 | Symbicort 200/6 Turbohaler (Pharmaram Ltd) |
| 5154 | Beta2AdrenoceptorAgonistsSelective | 37997711000001106 | Symbicort 400/12 Turbohaler (Pharmaram Ltd) |
| 5154 | Beta2AdrenoceptorAgonistsSelective | 38130011000001108 | DuoResp Spiromax 160micrograms/dose / 4.5micrograms/dose dry powder inhaler (DE Pharmaceuticals) |
| 5154 | Beta2AdrenoceptorAgonistsSelective | 38130211000001103 | DuoResp Spiromax 320micrograms/dose / 9micrograms/dose dry powder inhaler (DE Pharmaceuticals) |
| 5154 | Beta2AdrenoceptorAgonistsSelective | 38131211000001109 | Easyhaler Salbutamol sulfate 100micrograms/dose dry powder inhaler (DE Pharmaceuticals) |
| 5154 | Beta2AdrenoceptorAgonistsSelective | 38131411000001108 | Easyhaler Salbutamol sulfate 200micrograms/dose dry powder inhaler (DE Pharmaceuticals) |
| 5154 | Beta2AdrenoceptorAgonistsSelective | 38134511000001109 | Flutiform 125micrograms/dose / 5micrograms/dose inhaler (DE Pharmaceuticals) |
| 5154 | Beta2AdrenoceptorAgonistsSelective | 38134711000001104 | Flutiform 250micrograms/dose / 10micrograms/dose inhaler (DE Pharmaceuticals) |
| 5154 | Beta2AdrenoceptorAgonistsSelective | 38134911000001102 | Flutiform 50micrograms/dose / 5micrograms/dose inhaler (DE Pharmaceuticals) |
| 5154 | Beta2AdrenoceptorAgonistsSelective | 38135111000001101 | Flutiform K-haler 125micrograms/dose / 5micrograms/dose breath actuated inhaler (DE Pharmaceuticals) |

| ConditionID | Condition name | DMD code | Description |
|---|---|---|---|
| 5154 | Beta2AdrenoceptorAgonistsSelective | 38135311000001104 | Flutiform K-haler 50micrograms/dose / 5micrograms/dose breath actuated inhaler (DE Pharmaceuticals) |
| 5154 | Beta2AdrenoceptorAgonistsSelective | 38136011000001106 | Fostair NEXThaler 100micrograms/dose / 6micrograms/dose dry powder inhaler (DE Pharmaceuticals) |
| 5154 | Beta2AdrenoceptorAgonistsSelective | 38157111000001106 | Onbrez Breezhaler 150microgram inhalation powder capsules with device (DE Pharmaceuticals) |
| 5154 | Beta2AdrenoceptorAgonistsSelective | 38157311000001108 | Onbrez Breezhaler 300microgram inhalation powder capsules with device (DE Pharmaceuticals) |
| 5154 | Beta2AdrenoceptorAgonistsSelective | 38165311000001109 | Relvar Ellipta 92micrograms/dose / 22micrograms/dose dry powder inhaler (DE Pharmaceuticals) |
| 5154 | Beta2AdrenoceptorAgonistsSelective | 38165511000001103 | Relvar Ellipta 184micrograms/dose / 22micrograms/dose dry powder inhaler (DE Pharmaceuticals) |
| 5154 | Beta2AdrenoceptorAgonistsSelective | 38166911000001103 | Sirdupla 25micrograms/dose / 125micrograms/dose inhaler (DE Pharmaceuticals) |
| 5154 | Beta2AdrenoceptorAgonistsSelective | 38167111000001103 | Sirdupla 25micrograms/dose / 250micrograms/dose inhaler (DE Pharmaceuticals) |
| 5154 | Beta2AdrenoceptorAgonistsSelective | 38172811000001104 | Ventolin 5mg Nebules (DE Pharmaceuticals) |
| 5154 | Beta2AdrenoceptorAgonistsSelective | 38188711000001104 | Terbutaline 10mg/23ml solution for infusion pre-filled syringes (Special Order) |
| 5154 | Beta2AdrenoceptorAgonistsSelective | 38189211000001101 | Terbutaline 12mg/24ml solution for infusion pre-filled syringes (Special Order) |
| 5154 | Beta2AdrenoceptorAgonistsSelective | 38189511000001103 | Terbutaline 3mg/12ml solution for infusion pre-filled syringes (Special Order) |
| 5154 | Beta2AdrenoceptorAgonistsSelective | 38189911000001105 | Terbutaline 6mg/23ml solution for infusion pre-filled syringes (Special Order) |
| 5154 | Beta2AdrenoceptorAgonistsSelective | 38191511000001103 | Terbutaline 8mg/23ml solution for infusion pre-filled syringes (Special Order) |
| 5154 | Beta2AdrenoceptorAgonistsSelective | 38191811000001100 | Terbutaline 9mg/23ml solution for infusion pre-filled syringes (Special Order) |
| 5154 | Beta2AdrenoceptorAgonistsSelective | 38196411000001108 | Terbutaline 10mg/23ml solution for infusion pre-filled syringes |
| 5154 | Beta2AdrenoceptorAgonistsSelective | 38196511000001107 | Terbutaline 12mg/24ml solution for infusion pre-filled syringes |
| 5154 | Beta2AdrenoceptorAgonistsSelective | 38196611000001106 | Terbutaline 3mg/12ml solution for infusion pre-filled syringes |
| 5154 | Beta2AdrenoceptorAgonistsSelective | 38196711000001102 | Terbutaline 6mg/23ml solution for infusion pre-filled syringes |
| 5154 | Beta2AdrenoceptorAgonistsSelective | 38196811000001105 | Terbutaline 8mg/23ml solution for infusion pre-filled syringes |
| 5154 | Beta2AdrenoceptorAgonistsSelective | 38196911000001100 | Terbutaline 9mg/23ml solution for infusion pre-filled syringes |
| 5154 | Beta2AdrenoceptorAgonistsSelective | 38617811000001107 | Combiprasal 0.5mg/2.5mg nebuliser solution 2.5ml unit dose vials (TriOn Pharma Ltd) |
| 5154 | Beta2AdrenoceptorAgonistsSelective | 38640111000001109 | Sirdupla 25micrograms/dose / 125micrograms/dose inhaler (Pilsco Ltd) |
| 5154 | Beta2AdrenoceptorAgonistsSelective | 38640311000001106 | Sirdupla 25micrograms/dose / 250micrograms/dose inhaler (Pilsco Ltd) |
| 5154 | Beta2AdrenoceptorAgonistsSelective | 38895911000001101 | Salbutamol 500micrograms/1ml solution for injection ampoules |
| 5154 | Beta2AdrenoceptorAgonistsSelective | 38896811000001103 | Budesonide 200micrograms/dose / Formoterol 6micrograms/dose dry powder inhaler |
| 5154 | Beta2AdrenoceptorAgonistsSelective | 38897411000001103 | Fluticasone propionate 500micrograms/dose / Salmeterol 50micrograms/dose dry powder inhaler |
| 5154 | Beta2AdrenoceptorAgonistsSelective | 38897511000001104 | Fluticasone propionate 250micrograms/dose / Salmeterol 50micrograms/dose dry powder inhaler |
| 5154 | Beta2AdrenoceptorAgonistsSelective | 38897611000001100 | Fluticasone propionate 100micrograms/dose / Salmeterol 50micrograms/dose dry powder inhaler |
| 5154 | Beta2AdrenoceptorAgonistsSelective | 39025011000001108 | Salbutamol 4mg modified-release tablets |
| 5154 | Beta2AdrenoceptorAgonistsSelective | 39025111000001109 | Salbutamol 8mg modified-release capsules |
| 5154 | Beta2AdrenoceptorAgonistsSelective | 39025211000001103 | Salbutamol 4mg modified-release capsules |
| 5154 | Beta2AdrenoceptorAgonistsSelective | 39025311000001106 | Salbutamol 5mg/5ml solution for infusion ampoules |
| 5154 | Beta2AdrenoceptorAgonistsSelective | 39089311000001103 | Flutiform K-haler 125micrograms/dose / 5micrograms/dose breath actuated inhaler (Pilsco Ltd) |
| 5154 | Beta2AdrenoceptorAgonistsSelective | 39105811000001102 | Symbicort 100micrograms/dose / 3micrograms/dose pressurised inhaler (AstraZeneca UK Ltd) |
| 5154 | Beta2AdrenoceptorAgonistsSelective | 39109411000001101 | Salbutamol 2mg/5ml oral solution sugar free |
| 5154 | Beta2AdrenoceptorAgonistsSelective | 39110811000001105 | Salmeterol 25micrograms/dose inhaler |
| 5154 | Beta2AdrenoceptorAgonistsSelective | 39111011000001108 | Fluticasone 50micrograms/dose / Salmeterol 25micrograms/dose inhaler CFC free |

| Con ditio nID | Condition name | DMD code | Description |
|---|---|---|---|
| 5154 | Beta2AdrenoceptorAgonistsSelective | 39111111000001109 | Fluticasone 125micrograms/dose / Salmeterol 25micrograms/dose inhaler CFC free |
| 5154 | Beta2AdrenoceptorAgonistsSelective | 39111311000001106 | Fluticasone 250micrograms/dose / Salmeterol 25micrograms/dose inhaler CFC free |
| 5154 | Beta2AdrenoceptorAgonistsSelective | 39112711000001103 | Salbutamol 100micrograms/dose breath actuated inhaler CFC free |
| 5154 | Beta2AdrenoceptorAgonistsSelective | 39112911000001101 | Formoterol 6micrograms/dose dry powder inhaler |
| 5154 | Beta2AdrenoceptorAgonistsSelective | 39113011000001109 | Formoterol 12micrograms/dose dry powder inhaler |
| 5154 | Beta2AdrenoceptorAgonistsSelective | 39113111000001105 | Terbutaline 250micrograms/dose inhaler |
| 5154 | Beta2AdrenoceptorAgonistsSelective | 39113211000001104 | Salmeterol 50micrograms/dose dry powder inhaler |
| 5154 | Beta2AdrenoceptorAgonistsSelective | 39113311000001107 | Salbutamol 200micrograms/dose dry powder inhaler |
| 5154 | Beta2AdrenoceptorAgonistsSelective | 39113611000001102 | Salbutamol 100micrograms/dose inhaler CFC free |
| 5154 | Beta2AdrenoceptorAgonistsSelective | 39114511000001103 | Atectura Breezhaler 125micrograms/62.5micrograms inhalation powder capsules with device (Sandoz Ltd) |
| 5154 | Beta2AdrenoceptorAgonistsSelective | 39115411000001101 | Atectura Breezhaler 125micrograms/127.5micrograms inhalation powder capsules with device (Sandoz Ltd) |
| 5154 | Beta2AdrenoceptorAgonistsSelective | 39115911000001109 | Atectura Breezhaler 125micrograms/260micrograms inhalation powder capsules with device (Sandoz Ltd) |
| 5154 | Beta2AdrenoceptorAgonistsSelective | 39116311000001103 | Indacaterol 125micrograms/dose / Mometasone 127.5micrograms/dose inhalation powder capsules with device |
| 5154 | Beta2AdrenoceptorAgonistsSelective | 39116411000001105 | Indacaterol 125micrograms/dose / Mometasone 260micrograms/dose inhalation powder capsules with device |
| 5154 | Beta2AdrenoceptorAgonistsSelective | 39116511000001109 | Indacaterol 125micrograms/dose / Mometasone 62.5micrograms/dose inhalation powder capsules with device |
| 5154 | Beta2AdrenoceptorAgonistsSelective | 39133611000001108 | Budesonide 100micrograms/dose / Formoterol 3micrograms/dose inhaler CFC free |
| 5154 | Beta2AdrenoceptorAgonistsSelective | 39134511000001107 | Generic Enerzair Breezhaler 114micrograms/dose / 46micrograms/dose / 136micrograms/dose inhalation powder capsules with device |
| 5154 | Beta2AdrenoceptorAgonistsSelective | 39134711000001102 | Enerzair Breezhaler 114micrograms/dose / 46micrograms/dose / 136micrograms/dose inhalation powder capsules with device (Sandoz Ltd) |
| 5154 | Beta2AdrenoceptorAgonistsSelective | 39241911000001100 | Salbutamol 2mg tablets (Medihealth (Northern) Ltd) |
| 5154 | Beta2AdrenoceptorAgonistsSelective | 39242111000001108 | Salbutamol 4mg tablets (Medihealth (Northern) Ltd) |
| 5154 | Beta2AdrenoceptorAgonistsSelective | 39327311000001104 | Trixeo Aerosphere 5micrograms/dose / 7.2micrograms/dose / 160micrograms/dose pressurised inhaler (AstraZeneca UK Ltd) |
| 5154 | Beta2AdrenoceptorAgonistsSelective | 39329111000001107 | Bevespi Aerosphere 7.2micrograms/dose / 5micrograms/dose pressurised inhaler (AstraZeneca UK Ltd) |
| 5154 | Beta2AdrenoceptorAgonistsSelective | 39343511000001103 | Generic Trixeo Aerosphere 5micrograms/dose / 7.2micrograms/dose / 160micrograms/dose inhaler CFC free |
| 5154 | Beta2AdrenoceptorAgonistsSelective | 39343611000001104 | Glycopyrronium 7.2micrograms/dose / Formoterol 5micrograms/dose inhaler CFC free |
| 5154 | Beta2AdrenoceptorAgonistsSelective | 39359011000001107 | Bricanyl 500micrograms/dose Turbohaler (Pilsco Ltd) |
| 5154 | Beta2AdrenoceptorAgonistsSelective | 39360211000001103 | Fostair NEXThaler 100micrograms/dose / 6micrograms/dose dry powder inhaler (Pilsco Ltd) |
| 5154 | Beta2AdrenoceptorAgonistsSelective | 39360411000001104 | Sereflo 25micrograms/dose / 125micrograms/dose inhaler (Pilsco Ltd) |
| 5154 | Beta2AdrenoceptorAgonistsSelective | 39567411000001102 | Fixkoh Airmaster 50micrograms/dose / 500micrograms/dose dry powder inhaler (Thornton & Ross Ltd) |
| 5154 | Beta2AdrenoceptorAgonistsSelective | 39567611000001104 | Fixkoh Airmaster 50micrograms/dose / 250micrograms/dose dry powder inhaler (Thornton & Ross Ltd) |
| 5154 | Beta2AdrenoceptorAgonistsSelective | 39567811000001100 | Fixkoh Airmaster 50micrograms/dose / 100micrograms/dose dry powder inhaler (Thornton & Ross Ltd) |
| 5154 | Beta2AdrenoceptorAgonistsSelective | 39696311000001100 | Salbutamol 400microgram / Beclometasone 200microgram inhalation powder capsules |
| 5154 | Beta2AdrenoceptorAgonistsSelective | 39703611000001107 | Salbutamol 4mg tablets |
| 5154 | Beta2AdrenoceptorAgonistsSelective | 39709511000001105 | Salbutamol 5mg/ml nebuliser liquid |
| 5154 | Beta2AdrenoceptorAgonistsSelective | 39709611000001109 | Salbutamol 2.5mg/2.5ml nebuliser liquid unit dose vials |
| 5154 | Beta2AdrenoceptorAgonistsSelective | 39709711000001100 | Salbutamol 200microgram inhalation powder capsules |
| 5154 | Beta2AdrenoceptorAgonistsSelective | 39709811000001108 | Salbutamol 400microgram inhalation powder capsules |
| 5154 | Beta2AdrenoceptorAgonistsSelective | 39710011000001108 | Salbutamol 5mg/2.5ml nebuliser liquid unit dose vials |
| 5154 | Beta2AdrenoceptorAgonistsSelective | 39710511000001100 | Salbutamol 2.5mg/2.5ml / Ipratropium bromide 500micrograms/2.5ml nebuliser liquid unit dose vials |

| Con ditio nID | Condition name | DMD code | Description |
|---|---|---|---|
| 5154 | Beta2AdrenoceptorAgonistsSelective | 39711511000001107 | Fenoterol 1.25mg/4ml / Ipratropium 500micrograms/4ml nebuliser liquid unit dose vials |
| 5154 | Beta2AdrenoceptorAgonistsSelective | 39714711000001104 | Terbutaline 10mg/ml nebuliser liquid |
| 5154 | Beta2AdrenoceptorAgonistsSelective | 39817511000001103 | Luforbec 100micrograms/dose / 6micrograms/dose inhaler (Lupin Healthcare (UK) Ltd) |
| 5154 | Beta2AdrenoceptorAgonistsSelective | 39939611000001107 | Fostair 100micrograms/dose / 6micrograms/dose inhaler (CST Pharma Ltd) |
| 5154 | Beta2AdrenoceptorAgonistsSelective | 39993311000001105 | Trimbow NEXThaler 88micrograms/dose / 5micrograms/dose / 9micrograms/dose dry powder inhaler (Chiesi Ltd) |
| 5154 | Beta2AdrenoceptorAgonistsSelective | 40034211000001104 | Avenor 25micrograms/dose / 50micrograms/dose inhaler (Zentiva Pharma UK Ltd) |
| 5154 | Beta2AdrenoceptorAgonistsSelective | 40040711000001101 | Avenor 25micrograms/dose / 125micrograms/dose inhaler (Zentiva Pharma UK Ltd) |
| 5154 | Beta2AdrenoceptorAgonistsSelective | 40040911000001104 | Avenor 25micrograms/dose / 250micrograms/dose inhaler (Zentiva Pharma UK Ltd) |
| 5154 | Beta2AdrenoceptorAgonistsSelective | 40087411000001104 | Generic Trimbow NEXThaler 88micrograms/dose / 5micrograms/dose / 9micrograms/dose dry powder inhaler |
| 5154 | Beta2AdrenoceptorAgonistsSelective | 40106011000001102 | WockAIR 160micrograms/dose / 4.5micrograms/dose dry powder inhaler (Wockhardt UK Ltd) |
| 5154 | Beta2AdrenoceptorAgonistsSelective | 40106211000001107 | WockAIR 320micrograms/dose / 9micrograms/dose dry powder inhaler (Wockhardt UK Ltd) |
| 5154 | Beta2AdrenoceptorAgonistsSelective | 40220711000001106 | Seretide 50 Evohaler (CST Pharma Ltd) |
| 5154 | Beta2AdrenoceptorAgonistsSelective | 40220911000001108 | Fostair NEXThaler 100micrograms/dose / 6micrograms/dose dry powder inhaler (CST Pharma Ltd) |
| 5154 | Beta2AdrenoceptorAgonistsSelective | 40444911000001101 | Seffalair Spiromax 12.75micrograms/dose / 202micrograms/dose dry powder inhaler (Teva UK Ltd) |
| 5154 | Beta2AdrenoceptorAgonistsSelective | 40445111000001100 | Seffalair Spiromax 12.75micrograms/dose / 100micrograms/dose dry powder inhaler (Teva UK Ltd) |
| 5154 | Beta2AdrenoceptorAgonistsSelective | 40455711000001101 | Fluticasone propionate 100micrograms/dose / Salmeterol 12.75micrograms/dose dry powder inhaler |
| 5154 | Beta2AdrenoceptorAgonistsSelective | 40455811000001109 | Fluticasone propionate 202micrograms/dose / Salmeterol 12.75micrograms/dose dry powder inhaler |
| 5154 | Beta2AdrenoceptorAgonistsSelective | 40504911000001103 | Sereflo Ciphaler 50micrograms/dose / 250micrograms/dose dry powder inhaler (Cipla EU Ltd) |
| 5154 | Beta2AdrenoceptorAgonistsSelective | 40752211000001109 | Trimbow 172micrograms/dose / 5micrograms/dose / 9micrograms/dose inhaler (Chiesi Ltd) |
| 5154 | Beta2AdrenoceptorAgonistsSelective | 40766811000001104 | Generic Trimbow 172micrograms/dose / 5micrograms/dose / 9micrograms/dose inhaler |
| 5154 | Beta2AdrenoceptorAgonistsSelective | 40852311000001103 | Luforbec 200micrograms/dose / 6micrograms/dose inhaler (Lupin Healthcare (UK) Ltd) |
| 5154 | Beta2AdrenoceptorAgonistsSelective | 134498003 | Budesonide+eformoterol fumarate 200/6mcg breath-actuated dry powder inhaler |
| 5154 | Beta2AdrenoceptorAgonistsSelective | 134499006 | Budesonide 100micrograms/dose / Formoterol 6micrograms/dose dry powder inhaler |
| 5154 | Beta2AdrenoceptorAgonistsSelective | 320096005 | Salbutamol 4mg m/r tablet |
| 5154 | Beta2AdrenoceptorAgonistsSelective | 320099003 | Salbutamol 4mg m/r capsule |
| 5154 | Beta2AdrenoceptorAgonistsSelective | 320100006 | Salbutamol 8mg m/r capsule |
| 5154 | Beta2AdrenoceptorAgonistsSelective | 320104002 | Salbutamol 4mg tablets |
| 5154 | Beta2AdrenoceptorAgonistsSelective | 320106000 | Salbutamol 2mg tablets |
| 5154 | Beta2AdrenoceptorAgonistsSelective | 320107009 | Salbutamol 8mg modified-release tablets |
| 5154 | Beta2AdrenoceptorAgonistsSelective | 320108004 | Salbutamol 2mg/5mL sugar free syrup |
| 5154 | Beta2AdrenoceptorAgonistsSelective | 320116008 | Salbutamol 5mg/50ml solution for infusion vials |
| 5154 | Beta2AdrenoceptorAgonistsSelective | 320118009 | Salbutamol 500micrograms/1mL injection |
| 5154 | Beta2AdrenoceptorAgonistsSelective | 320119001 | Salbutamol 5mg/5mL intravenous infusion |
| 5154 | Beta2AdrenoceptorAgonistsSelective | 320132006 | Salbutamol 200microgram inhalation powder blisters with device |
| 5154 | Beta2AdrenoceptorAgonistsSelective | 320133001 | Salbutamol 400microgram inhalation powder blisters with device |
| 5154 | Beta2AdrenoceptorAgonistsSelective | 320134007 | Salbutamol 200microgram inhalation powder blisters |
| 5154 | Beta2AdrenoceptorAgonistsSelective | 320135008 | Salbutamol 400microgram inhalation powder blisters |
| 5154 | Beta2AdrenoceptorAgonistsSelective | 320136009 | Salbutamol 100micrograms/dose breath actuated inhaler |

| Con ditio nID | Condition name | DMD code | Description |
|---|---|---|---|
| 5154 | Beta2AdrenoceptorAgonistsSelective | 320139002 | Salbutamol 100micrograms CFC-free inhaler |
| 5154 | Beta2AdrenoceptorAgonistsSelective | 320141001 | Salbutamol 200micrograms breath-actuated dry powder inhaler |
| 5154 | Beta2AdrenoceptorAgonistsSelective | 320148007 | Salbutamol 95micrograms/dose dry powder inhaler |
| 5154 | Beta2AdrenoceptorAgonistsSelective | 320151000 | Salbutamol 100micrograms CFC-free breath-actuated aerosol inhaler |
| 5154 | Beta2AdrenoceptorAgonistsSelective | 320171009 | Salbutamol 5mg/2.5ml nebuliser liquid unit dose vials |
| 5154 | Beta2AdrenoceptorAgonistsSelective | 320176004 | Salbutamol 100micrograms/dose inhaler |
| 5154 | Beta2AdrenoceptorAgonistsSelective | 320177008 | Salbutamol 2.5mg/2.5ml nebuliser liquid unit dose vials |
| 5154 | Beta2AdrenoceptorAgonistsSelective | 320178003 | Salbutamol 200microgram inhalation powder capsules |
| 5154 | Beta2AdrenoceptorAgonistsSelective | 320179006 | Salbutamol 400microgram inhalation powder capsules |
| 5154 | Beta2AdrenoceptorAgonistsSelective | 320180009 | Salbutamol 5mg/ml nebuliser liquid |
| 5154 | Beta2AdrenoceptorAgonistsSelective | 320199000 | Terbutaline 2.5mg/5ml solution for injection ampoules |
| 5154 | Beta2AdrenoceptorAgonistsSelective | 320200002 | Terbutaline 10mg/ml nebuliser liquid |
| 5154 | Beta2AdrenoceptorAgonistsSelective | 320201003 | Terbutaline 500micrograms/dose dry powder inhaler |
| 5154 | Beta2AdrenoceptorAgonistsSelective | 320203000 | Terbutaline 5mg tablets |
| 5154 | Beta2AdrenoceptorAgonistsSelective | 320205007 | Terbutaline 500micrograms/1ml solution for injection ampoules |
| 5154 | Beta2AdrenoceptorAgonistsSelective | 320206008 | Terbutaline sulfate 250micrograms inhaler |
| 5154 | Beta2AdrenoceptorAgonistsSelective | 320208009 | Terbutaline 250micrograms/dose inhaler with spacer |
| 5154 | Beta2AdrenoceptorAgonistsSelective | 320211005 | Terbutaline 7.5mg modified-release tablets |
| 5154 | Beta2AdrenoceptorAgonistsSelective | 320212003 | Terbutaline 1.5mg/5ml oral solution sugar free |
| 5154 | Beta2AdrenoceptorAgonistsSelective | 320244004 | Salmeterol 25mcg inhaler |
| 5154 | Beta2AdrenoceptorAgonistsSelective | 320248001 | Salmeterol 50microgram inhalation powder blisters with device |
| 5154 | Beta2AdrenoceptorAgonistsSelective | 320249009 | Salmeterol 50microgram inhalation powder blisters |
| 5154 | Beta2AdrenoceptorAgonistsSelective | 320250009 | Salmeterol 50micrograms breath-actuated dry powder inhaler |
| 5154 | Beta2AdrenoceptorAgonistsSelective | 320258002 | Bambuterol 10mg tablets |
| 5154 | Beta2AdrenoceptorAgonistsSelective | 320259005 | Bambuterol 20mg tablets |
| 5154 | Beta2AdrenoceptorAgonistsSelective | 320263003 | Eformoterol fumarate dihydrate 6micrograms breath-actuated dry powder inhaler |
| 5154 | Beta2AdrenoceptorAgonistsSelective | 320264009 | Eformoterol fumarate dihydrate 12micrograms breath-actuated dry powder inhaler |
| 5154 | Beta2AdrenoceptorAgonistsSelective | 320274007 | Salmeterol+fluticasone propionate 25micrograms/50micrograms CFC-free inhaler |
| 5154 | Beta2AdrenoceptorAgonistsSelective | 320275008 | Salmeterol+fluticasone propionate 25micrograms/125micrograms CFC-free inhaler |
| 5154 | Beta2AdrenoceptorAgonistsSelective | 320276009 | Salmeterol+fluticasone propionate 25micrograms/250micrograms CFC-free inhaler |
| 5154 | Beta2AdrenoceptorAgonistsSelective | 320277000 | Salmeterol+fluticasone propionate 50micrograms/100micrograms breath-actuated dry powder inhaler |
| 5154 | Beta2AdrenoceptorAgonistsSelective | 320279002 | Salmeterol+fluticasone propionate 50micrograms/250micrograms breath-actuated dry powder inhaler |
| 5154 | Beta2AdrenoceptorAgonistsSelective | 320280004 | Salmeterol+fluticasone propionate 50micrograms/500micrograms breath-actuated dry powder inhaler |
| 5154 | Beta2AdrenoceptorAgonistsSelective | 320440005 | Fenoterol 100micrograms/dose / Ipratropium 40micrograms/dose inhaler |
| 5154 | Beta2AdrenoceptorAgonistsSelective | 320441009 | Fenoterol 100micrograms/dose / Ipratropium bromide 40micrograms/dose breath actuated inhaler |
| 5154 | Beta2AdrenoceptorAgonistsSelective | 320442002 | Salbutamol 100micrograms/dose / Ipratropium 20micrograms/dose inhaler |
| 5154 | Beta2AdrenoceptorAgonistsSelective | 320445000 | Salbutamol 2.5mg/2.5ml / Ipratropium bromide 500micrograms/2.5ml nebuliser liquid unit dose vials |
| 5154 | Beta2AdrenoceptorAgonistsSelective | 320543009 | Salbutamol 400microgram / Beclometasone 200microgram inhalation powder capsules |
| 5154 | Beta2AdrenoceptorAgonistsSelective | 320544003 | Salbutamol 200microgram / Beclometasone 100microgram inhalation powder capsules |
| 5154 | Beta2AdrenoceptorAgonistsSelective | 320545002 | Salbutamol 100micrograms/dose / Beclometasone 50micrograms/dose inhaler |

| Con ditio nID | Condition name | DMD code | Description |
|---|---|---|---|
| 5154 | Beta2AdrenoceptorAgonistsSelective | 320660007 | Sodium cromoglicate 1mg/dose / Salbutamol 100micrograms/dose inhaler with spacer |
| 5154 | Beta2AdrenoceptorAgonistsSelective | 320661006 | Sodium cromoglicate 1mg/dose / Salbutamol 100micrograms/dose inhaler |
| 5154 | Beta2AdrenoceptorAgonistsSelective | 349394001 | Fenoterol 1.25mg/4ml / Ipratropium 500micrograms/4ml nebuliser liquid unit dose vials |
| 5154 | Beta2AdrenoceptorAgonistsSelective | 7511000001105 | Salbutamol 100micrograms/dose inhaler CFC free (Actavis UK Ltd) |
| 5154 | Beta2AdrenoceptorAgonistsSelective | 28011000001107 | Monovent 1.5mg/5ml syrup (Sandoz Ltd) |
| 5154 | Beta2AdrenoceptorAgonistsSelective | 45111000001100 | Salbutamol 100micrograms/dose inhaler CFC free (Viatris UK Healthcare Ltd) |
| 5154 | Beta2AdrenoceptorAgonistsSelective | 52811000001102 | Salbutamol 4mg tablets (Alliance Healthcare (Distribution) Ltd) |
| 5154 | Beta2AdrenoceptorAgonistsSelective | 101011000001104 | Bricanyl 2.5mg/5ml solution for injection ampoules (AstraZeneca UK Ltd) |
| 5154 | Beta2AdrenoceptorAgonistsSelective | 106511000001103 | Salamol 100micrograms/dose inhaler CFC free (Teva UK Ltd) |
| 5154 | Beta2AdrenoceptorAgonistsSelective | 109751000001104 | Fenoterol 1.25mg/4ml / Ipratropium 500micrograms/4ml nebuliser liquid unit dose vials |
| 5154 | Beta2AdrenoceptorAgonistsSelective | 110651000001100 | Salbutamol 2.5mg/2.5ml / Ipratropium bromide 500micrograms/2.5ml nebuliser liquid unit dose vials |
| 5154 | Beta2AdrenoceptorAgonistsSelective | 111271000001104 | Salbutamol 5mg/ml nebuliser liquid |
| 5154 | Beta2AdrenoceptorAgonistsSelective | 111351000001100 | Salbutamol 5mg/2.5ml nebuliser liquid unit dose vials |
| 5154 | Beta2AdrenoceptorAgonistsSelective | 111431000001100 | Salbutamol 4mg tablets |
| 5154 | Beta2AdrenoceptorAgonistsSelective | 111961000001101 | Salbutamol 2.5mg/2.5ml nebuliser liquid unit dose vials |
| 5154 | Beta2AdrenoceptorAgonistsSelective | 112121000001102 | Salbutamol 400microgram / Beclometasone 200microgram inhalation powder capsules |
| 5154 | Beta2AdrenoceptorAgonistsSelective | 112651000001104 | Salbutamol 200microgram inhalation powder capsules |
| 5154 | Beta2AdrenoceptorAgonistsSelective | 113271000001103 | Salbutamol 400microgram inhalation powder capsules |
| 5154 | Beta2AdrenoceptorAgonistsSelective | 113751000001100 | Terbutaline 10mg/ml nebuliser liquid |
| 5154 | Beta2AdrenoceptorAgonistsSelective | 161111000001102 | Salbutamol 2mg/5ml oral solution sugar free (A A H Pharmaceuticals Ltd) |
| 5154 | Beta2AdrenoceptorAgonistsSelective | 161811000001109 | Salbutamol 2mg tablets (Kent Pharma (UK) Ltd) |
| 5154 | Beta2AdrenoceptorAgonistsSelective | 189411000001105 | Salbutamol 100micrograms/dose inhaler CFC free (Alliance Healthcare (Distribution) Ltd) |
| 5154 | Beta2AdrenoceptorAgonistsSelective | 194411000001107 | Salbutamol 4mg tablets (Accord Healthcare Ltd) |
| 5154 | Beta2AdrenoceptorAgonistsSelective | 196411000001104 | Volmax 4mg modified-release tablets (GlaxoSmithKline UK Ltd) |
| 5154 | Beta2AdrenoceptorAgonistsSelective | 221511000001100 | Salbutamol 2mg/5ml oral solution sugar free (Sandoz Ltd) |
| 5154 | Beta2AdrenoceptorAgonistsSelective | 222311000001102 | Ventolin 100micrograms/dose Evohaler (GlaxoSmithKline UK Ltd) |
| 5154 | Beta2AdrenoceptorAgonistsSelective | 225511000001102 | Terbutaline 1.5mg/5ml oral solution sugar free (Kent Pharma (UK) Ltd) |
| 5154 | Beta2AdrenoceptorAgonistsSelective | 248411000001109 | Terbutaline 1.5mg/5ml oral solution sugar free (Alliance Healthcare (Distribution) Ltd) |
| 5154 | Beta2AdrenoceptorAgonistsSelective | 258311000001100 | Salbutamol 4mg tablets (Kent Pharma (UK) Ltd) |
| 5154 | Beta2AdrenoceptorAgonistsSelective | 262711000001105 | Salbutamol 4mg tablets (A A H Pharmaceuticals Ltd) |
| 5154 | Beta2AdrenoceptorAgonistsSelective | 273911000001107 | Salbutamol 4mg tablets (Approved Prescription Services Ltd) |
| 5154 | Beta2AdrenoceptorAgonistsSelective | 281511000001105 | Ventmax SR 8mg capsules (Chiesi Ltd) |
| 5154 | Beta2AdrenoceptorAgonistsSelective | 287611000001100 | Salbutamol 2mg tablets (Approved Prescription Services Ltd) |
| 5154 | Beta2AdrenoceptorAgonistsSelective | 364811000001103 | Bricanyl SA 7.5mg tablets (AstraZeneca UK Ltd) |
| 5154 | Beta2AdrenoceptorAgonistsSelective | 375711000001100 | Bambec 10mg tablets (AstraZeneca UK Ltd) |
| 5154 | Beta2AdrenoceptorAgonistsSelective | 408911000001105 | Serevent 25micrograms/dose inhaler (GlaxoSmithKline UK Ltd) |
| 5154 | Beta2AdrenoceptorAgonistsSelective | 453611000001102 | Seretide 50 Evohaler (GlaxoSmithKline UK Ltd) |
| 5154 | Beta2AdrenoceptorAgonistsSelective | 506611000001108 | Salbutamol 2mg tablets (Accord Healthcare Ltd) |
| 5154 | Beta2AdrenoceptorAgonistsSelective | 539811000001106 | Seretide 250 Evohaler (GlaxoSmithKline UK Ltd) |
| 5154 | Beta2AdrenoceptorAgonistsSelective | 571311000001104 | Salbutamol 100micrograms/dose inhaler CFC free (A A H Pharmaceuticals Ltd) |
| 5154 | Beta2AdrenoceptorAgonistsSelective | 597011000001101 | Airomir 100micrograms/dose inhaler (Teva UK Ltd) |

| Con ditio nID | Condition name | DMD code | Description |
|---|---|---|---|
| 5154 | Beta2AdrenoceptorAgonistsSelective | 626411000001107 | Ventmax SR 4mg capsules (Chiesi Ltd) |
| 5154 | Beta2AdrenoceptorAgonistsSelective | 650211000001107 | Salapin 2mg/5ml syrup (Pinewood Healthcare) |
| 5154 | Beta2AdrenoceptorAgonistsSelective | 659111000001106 | Salbutamol 2mg tablets (A A H Pharmaceuticals Ltd) |
| 5154 | Beta2AdrenoceptorAgonistsSelective | 691211000001105 | Bricanyl 500micrograms/1ml solution for injection ampoules (AstraZeneca UK Ltd) |
| 5154 | Beta2AdrenoceptorAgonistsSelective | 726111000001105 | Bricanyl 1.5mg/5ml syrup (AstraZeneca UK Ltd) |
| 5154 | Beta2AdrenoceptorAgonistsSelective | 738811000001100 | Bricanyl 5mg tablets (AstraZeneca UK Ltd) |
| 5154 | Beta2AdrenoceptorAgonistsSelective | 757611000001104 | Salbutamol 100micrograms/dose inhaler CFC free (Teva UK Ltd) |
| 5154 | Beta2AdrenoceptorAgonistsSelective | 803911000001107 | Salbutamol 2mg/5ml oral solution sugar free (Alliance Healthcare (Distribution) Ltd) |
| 5154 | Beta2AdrenoceptorAgonistsSelective | 809411000001102 | Bambec 20mg tablets (AstraZeneca UK Ltd) |
| 5154 | Beta2AdrenoceptorAgonistsSelective | 810211000001105 | Seretide 125 Evohaler (GlaxoSmithKline UK Ltd) |
| 5154 | Beta2AdrenoceptorAgonistsSelective | 831811000001109 | Volmax 8mg modified-release tablets (GlaxoSmithKline UK Ltd) |
| 5154 | Beta2AdrenoceptorAgonistsSelective | 840111000001107 | Salbulin 100micrograms/dose inhaler (3M Health Care Ltd) |
| 5154 | Beta2AdrenoceptorAgonistsSelective | 886111000001103 | Ventolin 5mg/5ml solution for infusion ampoules (GlaxoSmithKline UK Ltd) |
| 5154 | Beta2AdrenoceptorAgonistsSelective | 892011000001102 | Ventolin 2mg/5ml syrup (GlaxoSmithKline UK Ltd) |
| 5154 | Beta2AdrenoceptorAgonistsSelective | 896511000001106 | Salbutamol 2mg/5ml oral solution sugar free (Kent Pharma (UK) Ltd) |
| 5154 | Beta2AdrenoceptorAgonistsSelective | 921311000001107 | Salbutamol 2mg tablets (Alliance Healthcare (Distribution) Ltd) |
| 5154 | Beta2AdrenoceptorAgonistsSelective | 2831711000001102 | Bricanyl 10mg/ml respirator solution (AstraZeneca UK Ltd) |
| 5154 | Beta2AdrenoceptorAgonistsSelective | 2923111000001107 | Duovent inhaler (Boehringer Ingelheim Ltd) |
| 5154 | Beta2AdrenoceptorAgonistsSelective | 2964011000001104 | Duovent Autohaler (Boehringer Ingelheim Ltd) |
| 5154 | Beta2AdrenoceptorAgonistsSelective | 3080411000001101 | Ventodisks 200microgram with Diskhaler (GlaxoSmithKline UK Ltd) |
| 5154 | Beta2AdrenoceptorAgonistsSelective | 3082411000001100 | Serevent 50microgram disks (GlaxoSmithKline UK Ltd) |
| 5154 | Beta2AdrenoceptorAgonistsSelective | 3083011000001100 | Ventodisks 400microgram with Diskhaler (GlaxoSmithKline UK Ltd) |
| 5154 | Beta2AdrenoceptorAgonistsSelective | 3084011000001103 | Serevent 50microgram disks with Diskhaler (GlaxoSmithKline UK Ltd) |
| 5154 | Beta2AdrenoceptorAgonistsSelective | 3086111000001109 | Ventodisks 200microgram (GlaxoSmithKline UK Ltd) |
| 5154 | Beta2AdrenoceptorAgonistsSelective | 3089011000001102 | Ventodisks 400microgram (GlaxoSmithKline UK Ltd) |
| 5154 | Beta2AdrenoceptorAgonistsSelective | 3186011000001104 | Asmasal 95micrograms/dose Clickhaler (Focus Pharmaceuticals Ltd) |
| 5154 | Beta2AdrenoceptorAgonistsSelective | 3186911000001100 | Seretide 100 Accuhaler (GlaxoSmithKline UK Ltd) |
| 5154 | Beta2AdrenoceptorAgonistsSelective | 3187211000001106 | Seretide 250 Accuhaler (GlaxoSmithKline UK Ltd) |
| 5154 | Beta2AdrenoceptorAgonistsSelective | 3188311000001102 | Seretide 500 Accuhaler (GlaxoSmithKline UK Ltd) |
| 5154 | Beta2AdrenoceptorAgonistsSelective | 3200511000001109 | Ventide Paediatric Rotacaps (GlaxoSmithKline UK Ltd) |
| 5154 | Beta2AdrenoceptorAgonistsSelective | 3202211000001107 | Ventide Rotacaps (GlaxoSmithKline UK Ltd) |
| 5154 | Beta2AdrenoceptorAgonistsSelective | 3206811000001109 | Foradil 12microgram inhalation powder capsules with device (Novartis Pharmaceuticals UK Ltd) |
| 5154 | Beta2AdrenoceptorAgonistsSelective | 3214211000001100 | Ventolin 200microgram Rotacaps (GlaxoSmithKline UK Ltd) |
| 5154 | Beta2AdrenoceptorAgonistsSelective | 3214311000001108 | Airomir 100micrograms/dose Autohaler (Teva UK Ltd) |
| 5154 | Beta2AdrenoceptorAgonistsSelective | 3214611000001103 | Salbutamol 200 Cyclocaps (Teva UK Ltd) |
| 5154 | Beta2AdrenoceptorAgonistsSelective | 3215311000001107 | Salamol 100micrograms/dose Easi-Breathe inhaler (Teva UK Ltd) |
| 5154 | Beta2AdrenoceptorAgonistsSelective | 3217611000001109 | Salbutamol 400 Cyclocaps (Teva UK Ltd) |
| 5154 | Beta2AdrenoceptorAgonistsSelective | 3218011000001101 | Ventolin 400microgram Rotacaps (GlaxoSmithKline UK Ltd) |
| 5154 | Beta2AdrenoceptorAgonistsSelective | 3218311000001103 | Bricanyl 500micrograms/dose Turbohaler (AstraZeneca UK Ltd) |
| 5154 | Beta2AdrenoceptorAgonistsSelective | 3235011000001104 | Aerocrom inhaler (Castlemead Healthcare Ltd) |
| 5154 | Beta2AdrenoceptorAgonistsSelective | 3243511000001107 | Oxis 6 Turbohaler (AstraZeneca UK Ltd) |
| 5154 | Beta2AdrenoceptorAgonistsSelective | 3245011000001103 | Oxis 12 Turbohaler (AstraZeneca UK Ltd) |
| 5154 | Beta2AdrenoceptorAgonistsSelective | 3248011000001106 | Bricanyl 250micrograms/dose inhaler (AstraZeneca UK Ltd) |

| Con ditio nID | Condition name | DMD code | Description |
|---|---|---|---|
| 5154 | Beta2AdrenoceptorAgonistsSelective | 3248711000001108 | Bricanyl 250micrograms/dose spacer inhaler (AstraZeneca UK Ltd) |
| 5154 | Beta2AdrenoceptorAgonistsSelective | 3292811000001106 | Ventide inhaler (GlaxoSmithKline UK Ltd) |
| 5154 | Beta2AdrenoceptorAgonistsSelective | 3293111000001105 | Aerolin 100micrograms/dose Autohaler (3M Health Care Ltd) |
| 5154 | Beta2AdrenoceptorAgonistsSelective | 3294211000001101 | Symbicort 100/6 Turbohaler (AstraZeneca UK Ltd) |
| 5154 | Beta2AdrenoceptorAgonistsSelective | 3294611000001104 | Symbicort 200/6 Turbohaler (AstraZeneca UK Ltd) |
| 5154 | Beta2AdrenoceptorAgonistsSelective | 3348611000001107 | Combivent inhaler (Boehringer Ingelheim Ltd) |
| 5154 | Beta2AdrenoceptorAgonistsSelective | 3379611000001104 | Salbutamol 5mg/2.5ml nebuliser liquid unit dose vials (A A H Pharmaceuticals Ltd) |
| 5154 | Beta2AdrenoceptorAgonistsSelective | 3380111000001107 | Serevent 50micrograms/dose Accuhaler (GlaxoSmithKline UK Ltd) |
| 5154 | Beta2AdrenoceptorAgonistsSelective | 3380811000001100 | Maxivent 5mg/2.5ml nebuliser liquid unit dose Steripoule vials (Ashbourne Pharmaceuticals Ltd) |
| 5154 | Beta2AdrenoceptorAgonistsSelective | 3381111000001101 | Salbutamol 5mg/2.5ml nebuliser liquid unit dose vials (Viatris UK Healthcare Ltd) |
| 5154 | Beta2AdrenoceptorAgonistsSelective | 3381811000001108 | Ventolin 5mg Nebules (GlaxoSmithKline UK Ltd) |
| 5154 | Beta2AdrenoceptorAgonistsSelective | 3382711000001107 | Ventolin 200micrograms/dose Accuhaler (GlaxoSmithKline UK Ltd) |
| 5154 | Beta2AdrenoceptorAgonistsSelective | 3383511000001109 | Salamol 5mg/2.5ml nebuliser liquid Steri-Neb unit dose vials (Teva UK Ltd) |
| 5154 | Beta2AdrenoceptorAgonistsSelective | 3384111000001103 | Pulvinal Salbutamol 200micrograms/dose dry powder inhaler (Chiesi Ltd) |
| 5154 | Beta2AdrenoceptorAgonistsSelective | 3385711000001103 | Salbutamol 2.5mg/2.5ml nebuliser liquid unit dose vials (A A H Pharmaceuticals Ltd) |
| 5154 | Beta2AdrenoceptorAgonistsSelective | 3386411000001100 | Maxivent 2.5mg/2.5ml nebuliser liquid unit dose Steripoule vials (Ashbourne Pharmaceuticals Ltd) |
| 5154 | Beta2AdrenoceptorAgonistsSelective | 3386811000001103 | Salbutamol 2.5mg/2.5ml nebuliser liquid unit dose vials (Viatris UK Healthcare Ltd) |
| 5154 | Beta2AdrenoceptorAgonistsSelective | 3387011000001107 | Ventolin 2.5mg Nebules (GlaxoSmithKline UK Ltd) |
| 5154 | Beta2AdrenoceptorAgonistsSelective | 3387511000001104 | Salamol 2.5mg/2.5ml nebuliser liquid Steri-Neb unit dose vials (Teva UK Ltd) |
| 5154 | Beta2AdrenoceptorAgonistsSelective | 3406011000001104 | Combivent nebuliser liquid 2.5ml UDVs (Boehringer Ingelheim Ltd) |
| 5154 | Beta2AdrenoceptorAgonistsSelective | 3408611000001107 | Salbutamol 100micrograms/dose inhaler (A A H Pharmaceuticals Ltd) |
| 5154 | Beta2AdrenoceptorAgonistsSelective | 3410611000001106 | Salbutamol 100micrograms/dose inhaler (Viatris UK Healthcare Ltd) |
| 5154 | Beta2AdrenoceptorAgonistsSelective | 3412611000001107 | Salbutamol 100micrograms/dose inhaler (Kent Pharma (UK) Ltd) |
| 5154 | Beta2AdrenoceptorAgonistsSelective | 3415711000001107 | Salbutamol 100micrograms/dose inhaler (Sandoz Ltd) |
| 5119 | CorticosteroidsInhaled | 134498003 | Budesonide+eformoterol fumarate 200/6mcg breath-actuated dry powder inhaler |
| 5119 | CorticosteroidsInhaled | 134499006 | Budesonide 100micrograms/dose / Formoterol 6micrograms/dose dry powder inhaler |
| 5119 | CorticosteroidsInhaled | 320274007 | Salmeterol+fluticasone propionate 25micrograms/50micrograms CFC-free inhaler |
| 5119 | CorticosteroidsInhaled | 320275008 | Salmeterol+fluticasone propionate 25micrograms/125micrograms CFC-free inhaler |
| 5119 | CorticosteroidsInhaled | 320276009 | Salmeterol+fluticasone propionate 25micrograms/250micrograms CFC-free inhaler |
| 5119 | CorticosteroidsInhaled | 320277000 | Salmeterol+fluticasone propionate 50micrograms/100micrograms breath-actuated dry powder inhaler |
| 5119 | CorticosteroidsInhaled | 320279002 | Salmeterol+fluticasone propionate 50micrograms/250micrograms breath-actuated dry powder inhaler |
| 5119 | CorticosteroidsInhaled | 320280004 | Salmeterol+fluticasone propionate 50micrograms/500micrograms breath-actuated dry powder inhaler |
| 5119 | CorticosteroidsInhaled | 320487003 | Beclometasone 400microgram inhalation powder blisters with device |
| 5119 | CorticosteroidsInhaled | 320488008 | Beclometasone 400microgram inhalation powder blisters |
| 5119 | CorticosteroidsInhaled | 320490009 | Beclometasone 50micrograms/dose breath actuated inhaler |
| 5119 | CorticosteroidsInhaled | 320491008 | Beclometasone 250micrograms/dose breath actuated inhaler |
| 5119 | CorticosteroidsInhaled | 320492001 | Beclometasone 100micrograms/dose breath actuated inhaler |
| 5119 | CorticosteroidsInhaled | 320520000 | Beclometasone 400microgram inhalation powder capsules |
| 5119 | CorticosteroidsInhaled | 320527002 | Beclometasone 100microgram inhalation powder blisters with device |

| Con ditio nID | Condition name | DMD code | Description |
|---|---|---|---|
| 5119 | CorticosteroidsInhaled | 320528007 | Beclometasone 200microgram inhalation powder blisters with device |
| 5119 | CorticosteroidsInhaled | 320529004 | Beclometasone 100microgram inhalation powder blisters |
| 5119 | CorticosteroidsInhaled | 320530009 | Beclometasone 200microgram inhalation powder blisters |
| 5119 | CorticosteroidsInhaled | 320531008 | Beclometasone 250micrograms/dose inhaler |
| 5119 | CorticosteroidsInhaled | 320532001 | Beclometasone 200micrograms/dose inhaler |
| 5119 | CorticosteroidsInhaled | 320533006 | Beclometasone 50micrograms/dose inhaler |
| 5119 | CorticosteroidsInhaled | 320534000 | Beclometasone 100microgram inhalation powder capsules |
| 5119 | CorticosteroidsInhaled | 320535004 | Beclometasone 200microgram inhalation powder capsules |
| 5119 | CorticosteroidsInhaled | 320537007 | Beclometasone dipropionate 100 microgram/actuation pressurised solution for inhalation |
| 5119 | CorticosteroidsInhaled | 320543009 | Salbutamol 400microgram / Beclometasone 200microgram inhalation powder capsules |
| 5119 | CorticosteroidsInhaled | 320544003 | Salbutamol 200microgram / Beclometasone 100microgram inhalation powder capsules |
| 5119 | CorticosteroidsInhaled | 320545002 | Salbutamol 100micrograms/dose / Beclometasone 50micrograms/dose inhaler |
| 5119 | CorticosteroidsInhaled | 320565009 | Budesonide 100micrograms/dose dry powder inhaler |
| 5119 | CorticosteroidsInhaled | 320567001 | Budesonide 200micrograms/dose dry powder inhaler |
| 5119 | CorticosteroidsInhaled | 320568006 | Budesonide 400micrograms/dose dry powder inhaler |
| 5119 | CorticosteroidsInhaled | 320571003 | Budesonide 200micrograms/dose inhaler |
| 5119 | CorticosteroidsInhaled | 320574006 | Budesonide 50micrograms/dose inhaler |
| 5119 | CorticosteroidsInhaled | 320580003 | Fluticasone propionate 50microgram inhalation powder blisters with device |
| 5119 | CorticosteroidsInhaled | 320581004 | Fluticasone propionate 100microgram inhalation powder blisters with device |
| 5119 | CorticosteroidsInhaled | 320582006 | Fluticasone propionate 250microgram inhalation powder blisters with device |
| 5119 | CorticosteroidsInhaled | 320586009 | Fluticasone propionate 50microgram inhalation powder blisters |
| 5119 | CorticosteroidsInhaled | 320587000 | Fluticasone propionate 100microgram inhalation powder blisters |
| 5119 | CorticosteroidsInhaled | 320588005 | Fluticasone propionate 250microgram inhalation powder blisters |
| 5119 | CorticosteroidsInhaled | 320592003 | Fluticasone 25micrograms/dose inhaler |
| 5119 | CorticosteroidsInhaled | 320599007 | Fluticasone propionate 500microgram inhalation powder blisters with device |
| 5119 | CorticosteroidsInhaled | 320600005 | Fluticasone propionate 500microgram inhalation powder blisters |
| 5119 | CorticosteroidsInhaled | 320602002 | Fluticasone propionate 50micrograms breath-actuated dry powder inhaler |
| 5119 | CorticosteroidsInhaled | 320603007 | Fluticasone propionate 100micrograms breath-actuated dry powder inhaler |
| 5119 | CorticosteroidsInhaled | 320604001 | Fluticasone propionate 250micrograms/dose dry powder inhaler |
| 5119 | CorticosteroidsInhaled | 320605000 | Fluticasone propionate 500micrograms breath-actuated dry powder inhaler |
| 5119 | CorticosteroidsInhaled | 320610001 | Fluticasone 500micrograms/2ml nebuliser liquid unit dose vials |
| 5119 | CorticosteroidsInhaled | 320611002 | Fluticasone 2mg/2ml nebuliser liquid unit dose vials |
| 5119 | CorticosteroidsInhaled | 320614005 | Fluticasone propionate 125micrograms CFC-free inhaler |
| 5119 | CorticosteroidsInhaled | 320615006 | Fluticasone propionate 250micrograms CFC-free inhaler |
| 5119 | CorticosteroidsInhaled | 320618008 | Fluticasone propionate 50micrograms CFC-free inhaler |
| 5119 | CorticosteroidsInhaled | 320630002 | Beclometasone 50micrograms/dose dry powder inhaler |
| 5119 | CorticosteroidsInhaled | 320631003 | Beclometasone 100micrograms/dose dry powder inhaler |
| 5119 | CorticosteroidsInhaled | 320632005 | Beclometasone 250micrograms/dose dry powder inhaler |
| 5119 | CorticosteroidsInhaled | 407769008 | Beclometasone 400micrograms/dose dry powder inhaler |
| 5119 | CorticosteroidsInhaled | 407770009 | Beclometasone 200micrograms/dose dry powder inhaler |
| 5119 | CorticosteroidsInhaled | 408013007 | Mometasone furoate 400micrograms breath-actuated dry powder inhaler |
| 5119 | CorticosteroidsInhaled | 408026006 | Mometasone furoate 200micrograms breath-actuated dry powder inhaler |

| Con ditio nID | Condition name | DMD code | Description |
|---|---|---|---|
| 5119 | CorticosteroidsInhaled | 408061000 | Beclometasone 50micrograms/dose inhaler CFC free |
| 5119 | CorticosteroidsInhaled | 408062007 | Beclometasone 50micrograms/dose breath actuated inhaler CFC free |
| 5119 | CorticosteroidsInhaled | 408063002 | Beclometasone 100micrograms/dose inhaler CFC free |
| 5119 | CorticosteroidsInhaled | 408064008 | Beclometasone 100micrograms/dose breath actuated inhaler CFC free |
| 5119 | CorticosteroidsInhaled | 109781000001108 | Beclometasone 400microgram inhalation powder capsules |
| 5119 | CorticosteroidsInhaled | 109941000001100 | Beclometasone 200microgram inhalation powder capsules |
| 5119 | CorticosteroidsInhaled | 111721000001109 | Fluticasone 2mg/2ml nebuliser liquid unit dose vials |
| 5119 | CorticosteroidsInhaled | 112121000001102 | Salbutamol 400microgram / Beclometasone 200microgram inhalation powder capsules |
| 5119 | CorticosteroidsInhaled | 113791000001106 | Fluticasone 500micrograms/2ml nebuliser liquid unit dose vials |
| 5119 | CorticosteroidsInhaled | 113851000001106 | Beclometasone 100microgram inhalation powder capsules |
| 5119 | CorticosteroidsInhaled | 398511000001105 | Flixotide 125micrograms/dose Evohaler (GlaxoSmithKline UK Ltd) |
| 5119 | CorticosteroidsInhaled | 453611000001102 | Seretide 50 Evohaler (GlaxoSmithKline UK Ltd) |
| 5119 | CorticosteroidsInhaled | 539811000001106 | Seretide 250 Evohaler (GlaxoSmithKline UK Ltd) |
| 5119 | CorticosteroidsInhaled | 726611000001102 | Flixotide 50micrograms/dose Evohaler (GlaxoSmithKline UK Ltd) |
| 5119 | CorticosteroidsInhaled | 810211000001105 | Seretide 125 Evohaler (GlaxoSmithKline UK Ltd) |
| 5119 | CorticosteroidsInhaled | 2831211000001109 | Flixotide 250micrograms/dose Evohaler (GlaxoSmithKline UK Ltd) |
| 5119 | CorticosteroidsInhaled | 2924111000001109 | Pulmicort 200micrograms/dose inhaler (AstraZeneca UK Ltd) |
| 5119 | CorticosteroidsInhaled | 3086011000001108 | Becodisks 100microgram (GlaxoSmithKline UK Ltd) |
| 5119 | CorticosteroidsInhaled | 3088611000001100 | Becodisks 100microgram with Diskhaler (GlaxoSmithKline UK Ltd) |
| 5119 | CorticosteroidsInhaled | 3096011000001109 | Becodisks 200microgram with Diskhaler (GlaxoSmithKline UK Ltd) |
| 5119 | CorticosteroidsInhaled | 3097711000001109 | Flixotide 50microgram disks with Diskhaler (GlaxoSmithKline UK Ltd) |
| 5119 | CorticosteroidsInhaled | 3098611000001101 | Flixotide 100microgram disks with Diskhaler (GlaxoSmithKline UK Ltd) |
| 5119 | CorticosteroidsInhaled | 3099611000001105 | Flixotide 100microgram disks (GlaxoSmithKline UK Ltd) |
| 5119 | CorticosteroidsInhaled | 3099811000001109 | Becodisks 200microgram (GlaxoSmithKline UK Ltd) |
| 5119 | CorticosteroidsInhaled | 3100811000001109 | Flixotide 50microgram disks (GlaxoSmithKline UK Ltd) |
| 5119 | CorticosteroidsInhaled | 3102211000001109 | Becodisks 400microgram with Diskhaler (GlaxoSmithKline UK Ltd) |
| 5119 | CorticosteroidsInhaled | 3103211000001103 | Becloforte 400microgram disks with Diskhaler (GlaxoSmithKline UK Ltd) |
| 5119 | CorticosteroidsInhaled | 3103511000001100 | Flixotide 250microgram disks (GlaxoSmithKline UK Ltd) |
| 5119 | CorticosteroidsInhaled | 3104911000001109 | Becodisks 400microgram (GlaxoSmithKline UK Ltd) |
| 5119 | CorticosteroidsInhaled | 3105311000001107 | Becloforte 400microgram disks (GlaxoSmithKline UK Ltd) |
| 5119 | CorticosteroidsInhaled | 3106311000001102 | Flixotide 250microgram disks with Diskhaler (GlaxoSmithKline UK Ltd) |
| 5119 | CorticosteroidsInhaled | 3108411000001105 | Flixotide 500microgram disks with Diskhaler (GlaxoSmithKline UK Ltd) |
| 5119 | CorticosteroidsInhaled | 3110511000001108 | Flixotide 500microgram disks (GlaxoSmithKline UK Ltd) |
| 5119 | CorticosteroidsInhaled | 3111911000001108 | Asmabec 50 Clickhaler (Focus Pharmaceuticals Ltd) |
| 5119 | CorticosteroidsInhaled | 3112411000001105 | Pulmicort 200 Turbohaler (AstraZeneca UK Ltd) |
| 5119 | CorticosteroidsInhaled | 3112511000001109 | Asmabec 100 Clickhaler (Focus Pharmaceuticals Ltd) |
| 5119 | CorticosteroidsInhaled | 3112911000001102 | Pulvinal Beclometasone Dipropionate 100micrograms/dose dry powder inhaler (Chiesi Ltd) |
| 5119 | CorticosteroidsInhaled | 3113111000001106 | Pulmicort 100 Turbohaler (AstraZeneca UK Ltd) |
| 5119 | CorticosteroidsInhaled | 3113411000001101 | Pulvinal Beclometasone Dipropionate 200micrograms/dose dry powder inhaler (Chiesi Ltd) |
| 5119 | CorticosteroidsInhaled | 3174111000001102 | Asmabec 250 Clickhaler (Focus Pharmaceuticals Ltd) |
| 5119 | CorticosteroidsInhaled | 3175111000001103 | Pulvinal Beclometasone Dipropionate 400micrograms/dose dry powder inhaler (Chiesi Ltd) |
| 5119 | CorticosteroidsInhaled | 3175211000001109 | Filair 50 inhaler (Meda Pharmaceuticals Ltd) |
| 5119 | CorticosteroidsInhaled | 3175611000001106 | Qvar 100 inhaler (Teva UK Ltd) |

| Con ditio nID | Condition name | DMD code | Description |
|---|---|---|---|
| 5119 | CorticosteroidsInhaled | 3175711000001102 | Beclometasone 50micrograms/dose inhaler (A A H Pharmaceuticals Ltd) |
| 5119 | CorticosteroidsInhaled | 3175911000001100 | Beclometasone 50micrograms/dose inhaler (Viatris UK Healthcare Ltd) |
| 5119 | CorticosteroidsInhaled | 3176211000001103 | Becotide 50 inhaler (GlaxoSmithKline UK Ltd) |
| 5119 | CorticosteroidsInhaled | 3176411000001104 | Beclazone 50 inhaler (Teva UK Ltd) |
| 5119 | CorticosteroidsInhaled | 3176711000001105 | Beclometasone 50micrograms/dose inhaler (Kent Pharma (UK) Ltd) |
| 5119 | CorticosteroidsInhaled | 3176811000001102 | Qvar 50 inhaler (Teva UK Ltd) |
| 5119 | CorticosteroidsInhaled | 3177111000001107 | Beclometasone 50micrograms/dose inhaler (Alliance Healthcare (Distribution) Ltd) |
| 5119 | CorticosteroidsInhaled | 3177411000001102 | Qvar 50 Autohaler (Teva UK Ltd) |
| 5119 | CorticosteroidsInhaled | 3177711000001108 | Qvar 100 Autohaler (Teva UK Ltd) |
| 5119 | CorticosteroidsInhaled | 3177911000001105 | Filair 100 inhaler (Meda Pharmaceuticals Ltd) |
| 5119 | CorticosteroidsInhaled | 3178211000001102 | Beclometasone 100micrograms/dose inhaler (A A H Pharmaceuticals Ltd) |
| 5119 | CorticosteroidsInhaled | 3178411000001103 | Beclometasone 100micrograms/dose inhaler (Viatris UK Healthcare Ltd) |
| 5119 | CorticosteroidsInhaled | 3178611000001100 | Becotide 100 inhaler (GlaxoSmithKline UK Ltd) |
| 5119 | CorticosteroidsInhaled | 3178811000001101 | Beclazone 100 inhaler (Teva UK Ltd) |
| 5119 | CorticosteroidsInhaled | 3179011000001102 | Beclometasone 100micrograms/dose inhaler (Alliance Healthcare (Distribution) Ltd) |
| 5119 | CorticosteroidsInhaled | 3179311000001104 | Becotide 200 inhaler (GlaxoSmithKline UK Ltd) |
| 5119 | CorticosteroidsInhaled | 3179511000001105 | Beclazone 200 inhaler (Teva UK Ltd) |
| 5119 | CorticosteroidsInhaled | 3179811000001108 | Beclometasone 250micrograms/dose inhaler (A A H Pharmaceuticals Ltd) |
| 5119 | CorticosteroidsInhaled | 3180011000001102 | Beclometasone 250micrograms/dose inhaler (Viatris UK Healthcare Ltd) |
| 5119 | CorticosteroidsInhaled | 3180211000001107 | Becloforte 250micrograms/dose inhaler (GlaxoSmithKline UK Ltd) |
| 5119 | CorticosteroidsInhaled | 3180611000001109 | Beclazone 250 inhaler (Teva UK Ltd) |
| 5119 | CorticosteroidsInhaled | 3180911000001103 | Beclometasone 250micrograms/dose inhaler (Alliance Healthcare (Distribution) Ltd) |
| 5119 | CorticosteroidsInhaled | 3181411000001102 | AeroBec 100 Autohaler (Meda Pharmaceuticals Ltd) |
| 5119 | CorticosteroidsInhaled | 3181711000001108 | Beclazone 100 Easi-Breathe inhaler (Teva UK Ltd) |
| 5119 | CorticosteroidsInhaled | 3182411000001107 | AeroBec Forte 250 Autohaler (Meda Pharmaceuticals Ltd) |
| 5119 | CorticosteroidsInhaled | 3182611000001105 | Beclazone 250 Easi-Breathe inhaler (Teva UK Ltd) |
| 5119 | CorticosteroidsInhaled | 3183811000001101 | Flixotide 50micrograms/dose Accuhaler (GlaxoSmithKline UK Ltd) |
| 5119 | CorticosteroidsInhaled | 3184311000001107 | Flixotide 100micrograms/dose Accuhaler (GlaxoSmithKline UK Ltd) |
| 5119 | CorticosteroidsInhaled | 3184911000001108 | Flixotide 250micrograms/dose Accuhaler (GlaxoSmithKline UK Ltd) |
| 5119 | CorticosteroidsInhaled | 3185211000001103 | Flixotide 500micrograms/dose Accuhaler (GlaxoSmithKline UK Ltd) |
| 5119 | CorticosteroidsInhaled | 3186911000001100 | Seretide 100 Accuhaler (GlaxoSmithKline UK Ltd) |
| 5119 | CorticosteroidsInhaled | 3187211000001106 | Seretide 250 Accuhaler (GlaxoSmithKline UK Ltd) |
| 5119 | CorticosteroidsInhaled | 3188311000001102 | Seretide 500 Accuhaler (GlaxoSmithKline UK Ltd) |
| 5119 | CorticosteroidsInhaled | 3189711000001107 | Beclometasone 100 Cyclocaps (Teva UK Ltd) |
| 5119 | CorticosteroidsInhaled | 3190311000001103 | Becotide 100microgram Rotacaps (GlaxoSmithKline UK Ltd) |
| 5119 | CorticosteroidsInhaled | 3192111000001101 | Becotide 200microgram Rotacaps (GlaxoSmithKline UK Ltd) |
| 5119 | CorticosteroidsInhaled | 3192611000001109 | Beclometasone 200 Cyclocaps (Teva UK Ltd) |
| 5119 | CorticosteroidsInhaled | 3194011000001108 | Becotide 400microgram Rotacaps (GlaxoSmithKline UK Ltd) |
| 5119 | CorticosteroidsInhaled | 3194511000001100 | Beclometasone 400 Cyclocaps (Teva UK Ltd) |
| 5119 | CorticosteroidsInhaled | 3197211000001105 | Budesonide 200 Cyclocaps (Teva UK Ltd) |
| 5119 | CorticosteroidsInhaled | 3198411000001108 | Budesonide 400 Cyclocaps (Teva UK Ltd) |
| 5119 | CorticosteroidsInhaled | 3200511000001109 | Ventide Paediatric Rotacaps (GlaxoSmithKline UK Ltd) |
| 5119 | CorticosteroidsInhaled | 3202211000001107 | Ventide Rotacaps (GlaxoSmithKline UK Ltd) |

| Con ditio nID | Condition name | DMD code | Description |
|---|---|---|---|
| 5119 | CorticosteroidsInhaled | 3228711000001106 | Pulmicort 400 Turbohaler (AstraZeneca UK Ltd) |
| 5119 | CorticosteroidsInhaled | 3240911000001108 | Pulmicort LS 50micrograms/dose inhaler (AstraZeneca UK Ltd) |
| 5119 | CorticosteroidsInhaled | 3292811000001106 | Ventide inhaler (GlaxoSmithKline UK Ltd) |
| 5119 | CorticosteroidsInhaled | 3294211000001101 | Symbicort 100/6 Turbohaler (AstraZeneca UK Ltd) |
| 5119 | CorticosteroidsInhaled | 3294611000001104 | Symbicort 200/6 Turbohaler (AstraZeneca UK Ltd) |
| 5119 | CorticosteroidsInhaled | 3389111000001102 | Flixotide 0.5mg/2ml Nebules (GlaxoSmithKline UK Ltd) |
| 5119 | CorticosteroidsInhaled | 3397211000001103 | Flixotide 2mg/2ml Nebules (GlaxoSmithKline UK Ltd) |
| 5119 | CorticosteroidsInhaled | 3432911000001109 | Budesonide 200microgram inhalation powder capsules |
| 5119 | CorticosteroidsInhaled | 3433011000001101 | Budesonide 400microgram inhalation powder capsules |
| 5119 | CorticosteroidsInhaled | 3604611000001104 | Beclazone 50 Easi-Breathe inhaler (Teva UK Ltd) |
| 5119 | CorticosteroidsInhaled | 3604911000001105 | AeroBec 50 Autohaler (Meda Pharmaceuticals Ltd) |
| 5119 | CorticosteroidsInhaled | 3635411000001106 | Pulmicort 0.5mg Respules (AstraZeneca UK Ltd) |
| 5119 | CorticosteroidsInhaled | 3636511000001103 | Pulmicort 1mg Respules (AstraZeneca UK Ltd) |
| 5119 | CorticosteroidsInhaled | 3654511000001105 | Budesonide 500micrograms/2ml nebuliser liquid unit dose vials |
| 5119 | CorticosteroidsInhaled | 3654611000001109 | Budesonide 1mg/2ml nebuliser liquid unit dose vials |
| 5119 | CorticosteroidsInhaled | 4043811000001103 | Asmanex 400micrograms/dose Twisthaler (Organon Pharma (UK) Ltd) |
| 5119 | CorticosteroidsInhaled | 4045711000001107 | Asmanex 200micrograms/dose Twisthaler (Organon Pharma (UK) Ltd) |
| 5119 | CorticosteroidsInhaled | 4332611000001107 | Beclometasone 200micrograms/dose inhaler (A A H Pharmaceuticals Ltd) |
| 5119 | CorticosteroidsInhaled | 4373811000001100 | Symbicort 400/12 Turbohaler (AstraZeneca UK Ltd) |
| 5119 | CorticosteroidsInhaled | 4378111000001103 | Budesonide 400micrograms/dose / Formoterol 12micrograms/dose dry powder inhaler |
| 5119 | CorticosteroidsInhaled | 4753511000001101 | Filair Forte 250micrograms/dose inhaler (Meda Pharmaceuticals Ltd) |
| 5119 | CorticosteroidsInhaled | 4773611000001100 | Beclometasone 50micrograms/dose inhaler (Teva UK Ltd) |
| 5119 | CorticosteroidsInhaled | 4773811000001101 | Beclometasone 100micrograms/dose inhaler (Teva UK Ltd) |
| 5119 | CorticosteroidsInhaled | 4774011000001109 | Beclometasone 250micrograms/dose inhaler (Teva UK Ltd) |
| 5119 | CorticosteroidsInhaled | 4856011000001103 | Flixotide 25micrograms/dose inhaler (GlaxoSmithKline UK Ltd) |
| 5119 | CorticosteroidsInhaled | 4860811000001104 | Pulmicort 200micrograms/dose inhaler with Nebuchamber (AstraZeneca UK Ltd) |
| 5119 | CorticosteroidsInhaled | 4864911000001104 | Budesonide 200micrograms/dose inhaler with spacer |
| 5119 | CorticosteroidsInhaled | 5256011000001101 | Becloforte 250micrograms/dose inhaler (Waymade Healthcare Plc) |
| 5119 | CorticosteroidsInhaled | 5256411000001105 | Becotide 200microgram Rotacaps (Waymade Healthcare Plc) |
| 5119 | CorticosteroidsInhaled | 5256611000001108 | Becotide 100 inhaler (Waymade Healthcare Plc) |
| 5119 | CorticosteroidsInhaled | 5256811000001107 | Becotide 50 inhaler (Waymade Healthcare Plc) |
| 5119 | CorticosteroidsInhaled | 5258111000001101 | Budesonide 400micrograms/dose Turbohaler (Waymade Healthcare Plc) |
| 5119 | CorticosteroidsInhaled | 5261111000001103 | Flixotide 100micrograms/dose Accuhaler (Waymade Healthcare Plc) |
| 5119 | CorticosteroidsInhaled | 5261411000001108 | Flixotide 250micrograms/dose Accuhaler (Waymade Healthcare Plc) |
| 5119 | CorticosteroidsInhaled | 5262211000001102 | Flixotide 500micrograms/dose Accuhaler (Waymade Healthcare Plc) |
| 5119 | CorticosteroidsInhaled | 5265811000001108 | Flixotide 100microgram disks (Waymade Healthcare Plc) |
| 5119 | CorticosteroidsInhaled | 5266111000001107 | Flixotide 250microgram disks (Waymade Healthcare Plc) |
| 5119 | CorticosteroidsInhaled | 5266711000001108 | Flixotide 500microgram disks (Waymade Healthcare Plc) |
| 5119 | CorticosteroidsInhaled | 5267111000001105 | Flixotide 125micrograms/dose Evohaler (Waymade Healthcare Plc) |
| 5119 | CorticosteroidsInhaled | 5268111000001106 | Flixotide 250micrograms/dose Evohaler (Waymade Healthcare Plc) |
| 5119 | CorticosteroidsInhaled | 5273211000001102 | Pulmicort 100 Turbohaler (Waymade Healthcare Plc) |
| 5119 | CorticosteroidsInhaled | 5273611000001100 | Pulmicort 200 Turbohaler (Waymade Healthcare Plc) |
| 5119 | CorticosteroidsInhaled | 5273911000001106 | Pulmicort 400 Turbohaler (Waymade Healthcare Plc) |

| Con ditio nID | Condition name | DMD code | Description |
|---|---|---|---|
| 5119 | CorticosteroidsInhaled | 5275611000001101 | Seretide 100 Accuhaler (Waymade Healthcare Plc) |
| 5119 | CorticosteroidsInhaled | 5276011000001104 | Seretide 250 Accuhaler (Waymade Healthcare Plc) |
| 5119 | CorticosteroidsInhaled | 5276211000001109 | Seretide 500 Accuhaler (Waymade Healthcare Plc) |
| 5119 | CorticosteroidsInhaled | 5276811000001105 | Seretide 250 Evohaler (Waymade Healthcare Plc) |
| 5119 | CorticosteroidsInhaled | 5277211000001106 | Symbicort 200/6 Turbohaler (Waymade Healthcare Plc) |
| 5119 | CorticosteroidsInhaled | 5278711000001103 | Ventide inhaler (Waymade Healthcare Plc) |
| 5119 | CorticosteroidsInhaled | 5282711000001106 | Becloforte 250micrograms/dose inhaler (Dowelhurst Ltd) |
| 5119 | CorticosteroidsInhaled | 5284411000001107 | Becotide 200microgram Rotacaps (Dowelhurst Ltd) |
| 5119 | CorticosteroidsInhaled | 5284711000001101 | Becotide 50 inhaler (Dowelhurst Ltd) |
| 5119 | CorticosteroidsInhaled | 5290911000001105 | Flixotide 100micrograms/dose Accuhaler (Dowelhurst Ltd) |
| 5119 | CorticosteroidsInhaled | 5292311000001108 | Flixotide 100microgram disks (Dowelhurst Ltd) |
| 5119 | CorticosteroidsInhaled | 5293111000001100 | Flixotide 125micrograms/dose Evohaler (Dowelhurst Ltd) |
| 5119 | CorticosteroidsInhaled | 5293811000001107 | Flixotide 250micrograms/dose Accuhaler (Dowelhurst Ltd) |
| 5119 | CorticosteroidsInhaled | 5294311000001101 | Flixotide 250microgram disks (Dowelhurst Ltd) |
| 5119 | CorticosteroidsInhaled | 5294611000001106 | Flixotide 250micrograms/dose Evohaler (Dowelhurst Ltd) |
| 5119 | CorticosteroidsInhaled | 5294811000001105 | Flixotide 500micrograms/dose Accuhaler (Dowelhurst Ltd) |
| 5119 | CorticosteroidsInhaled | 5295011000001100 | Flixotide 500microgram disks (Dowelhurst Ltd) |
| 5119 | CorticosteroidsInhaled | 5295211000001105 | Flixotide 50micrograms/dose Accuhaler (Dowelhurst Ltd) |
| 5119 | CorticosteroidsInhaled | 5310511000001105 | Pulmicort 0.5mg Respules (Dowelhurst Ltd) |
| 5119 | CorticosteroidsInhaled | 5311011000001106 | Pulmicort 100 Turbohaler (Dowelhurst Ltd) |
| 5119 | CorticosteroidsInhaled | 5312311000001100 | Pulmicort 1mg Respules (Dowelhurst Ltd) |
| 5119 | CorticosteroidsInhaled | 5313011000001107 | Pulmicort 200 Turbohaler (Dowelhurst Ltd) |
| 5119 | CorticosteroidsInhaled | 5313811000001101 | Pulmicort 400 Turbohaler (Dowelhurst Ltd) |
| 5119 | CorticosteroidsInhaled | 5317111000001100 | Seretide 100 Accuhaler (Dowelhurst Ltd) |
| 5119 | CorticosteroidsInhaled | 5317311000001103 | Seretide 250 Accuhaler (Dowelhurst Ltd) |
| 5119 | CorticosteroidsInhaled | 5317511000001109 | Seretide 500 Accuhaler (Dowelhurst Ltd) |
| 5119 | CorticosteroidsInhaled | 5321011000001102 | Symbicort 200/6 Turbohaler (Dowelhurst Ltd) |
| 5119 | CorticosteroidsInhaled | 5324211000001109 | Ventide inhaler (Dowelhurst Ltd) |
| 5119 | CorticosteroidsInhaled | 5350011000001105 | Budesonide 100micrograms/dose Turbohaler (Dowelhurst Ltd) |
| 5119 | CorticosteroidsInhaled | 5350211000001100 | Budesonide 200micrograms/dose Turbohaler (Dowelhurst Ltd) |
| 5119 | CorticosteroidsInhaled | 5350411000001101 | Budesonide 400micrograms/dose Turbohaler (Dowelhurst Ltd) |
| 5119 | CorticosteroidsInhaled | 5350911000001109 | Fluticasone 125micrograms/dose Evohaler (Dowelhurst Ltd) |
| 5119 | CorticosteroidsInhaled | 5351111000001100 | Fluticasone 250micrograms/dose Evohaler (Dowelhurst Ltd) |
| 5119 | CorticosteroidsInhaled | 5404711000001104 | Pulmicort 0.5mg Respules (Waymade Healthcare Plc) |
| 5119 | CorticosteroidsInhaled | 5525411000001107 | Becotide 100microgram Rotacaps (Dowelhurst Ltd) |
| 5119 | CorticosteroidsInhaled | 7379111000001102 | Beclometasone 100micrograms/dose inhaler (Kent Pharma (UK) Ltd) |
| 5119 | CorticosteroidsInhaled | 7379311000001100 | Beclometasone 250micrograms/dose inhaler (Kent Pharma (UK) Ltd) |
| 5119 | CorticosteroidsInhaled | 8024611000001102 | Budesonide 200micrograms/dose dry powder inhalation cartridge with device |
| 5119 | CorticosteroidsInhaled | 8031811000001102 | Budelin Novolizer 200micrograms/dose inhalation powder (Viatris UK Healthcare Ltd) |
| 5119 | CorticosteroidsInhaled | 8159511000001107 | Qvar 50micrograms/dose Easi-Breathe inhaler (Teva UK Ltd) |
| 5119 | CorticosteroidsInhaled | 8159711000001102 | Qvar 100micrograms/dose Easi-Breathe inhaler (Teva UK Ltd) |
| 5119 | CorticosteroidsInhaled | 9003911000001102 | Alvesco 80 inhaler (Covis Pharma Europe B.V.) |
| 5119 | CorticosteroidsInhaled | 9004211000001109 | Alvesco 160 inhaler (Covis Pharma Europe B.V.) |

| Con ditio nID | Condition name | DMD code | Description |
|---|---|---|---|
| 5119 | CorticosteroidsInhaled | 9004411000001108 | Ciclesonide 160micrograms/dose inhaler CFC free |
| 5119 | CorticosteroidsInhaled | 9004511000001107 | Ciclesonide 80micrograms/dose inhaler CFC free |
| 5119 | CorticosteroidsInhaled | 9111811000001100 | Budelin Novolizer 200micrograms/dose inhalation powder refill (Viatris UK Healthcare Ltd) |
| 5119 | CorticosteroidsInhaled | 9117811000001107 | Budesonide 200micrograms/dose dry powder inhalation cartridge |
| 5119 | CorticosteroidsInhaled | 9525111000001105 | Easyhaler Beclometasone 200micrograms/dose dry powder inhaler (Orion Pharma (UK) Ltd) |
| 5119 | CorticosteroidsInhaled | 10073911000001106 | Easyhaler Budesonide 100micrograms/dose dry powder inhaler (Orion Pharma (UK) Ltd) |
| 5119 | CorticosteroidsInhaled | 10074411000001100 | Easyhaler Budesonide 400micrograms/dose dry powder inhaler (Orion Pharma (UK) Ltd) |
| 5119 | CorticosteroidsInhaled | 10074611000001102 | Easyhaler Budesonide 200micrograms/dose dry powder inhaler (Orion Pharma (UK) Ltd) |
| 5119 | CorticosteroidsInhaled | 10272811000001101 | Budesonide 500micrograms/2ml nebuliser liquid unit dose vials (A A H Pharmaceuticals Ltd) |
| 5119 | CorticosteroidsInhaled | 10273211000001108 | Budesonide 1mg/2ml nebuliser liquid unit dose vials (A A H Pharmaceuticals Ltd) |
| 5119 | CorticosteroidsInhaled | 10273611000001105 | Budesonide 500micrograms/2ml nebuliser liquid unit dose vials (Teva UK Ltd) |
| 5119 | CorticosteroidsInhaled | 10274211000001106 | Budesonide 1mg/2ml nebuliser liquid unit dose vials (Teva UK Ltd) |
| 5119 | CorticosteroidsInhaled | 10326511000001109 | Budesonide 500micrograms/2ml nebuliser liquid unit dose vials (Kent Pharma (UK) Ltd) |
| 5119 | CorticosteroidsInhaled | 10326711000001104 | Budesonide 1mg/2ml nebuliser liquid unit dose vials (Kent Pharma (UK) Ltd) |
| 5119 | CorticosteroidsInhaled | 10347711000001104 | Budesonide 500micrograms/2ml nebuliser liquid unit dose vials (Alliance Healthcare (Distribution) Ltd) |
| 5119 | CorticosteroidsInhaled | 10347911000001102 | Budesonide 1mg/2ml nebuliser liquid unit dose vials (Alliance Healthcare (Distribution) Ltd) |
| 5119 | CorticosteroidsInhaled | 10453011000001102 | Budesonide 500micrograms/2ml nebuliser liquid unit dose vials (Arrow Generics Ltd) |
| 5119 | CorticosteroidsInhaled | 10453211000001107 | Budesonide 1mg/2ml nebuliser liquid unit dose vials (Arrow Generics Ltd) |
| 5119 | CorticosteroidsInhaled | 10472811000001102 | Seretide 250 Evohaler (Dowelhurst Ltd) |
| 5119 | CorticosteroidsInhaled | 10473211000001109 | Seretide 125 Evohaler (Dowelhurst Ltd) |
| 5119 | CorticosteroidsInhaled | 10506611000001103 | Symbicort 400/12 Turbohaler (Waymade Healthcare Plc) |
| 5119 | CorticosteroidsInhaled | 10512011000001109 | Seretide 125 Evohaler (Waymade Healthcare Plc) |
| 5119 | CorticosteroidsInhaled | 10527411000001109 | Pulmicort LS 50micrograms/dose inhaler (Waymade Healthcare Plc) |
| 5119 | CorticosteroidsInhaled | 10617711000001103 | Clenil Modulite 50micrograms/dose inhaler (Chiesi Ltd) |
| 5119 | CorticosteroidsInhaled | 10618211000001109 | Clenil Modulite 100micrograms/dose inhaler (Chiesi Ltd) |
| 5119 | CorticosteroidsInhaled | 10619311000001107 | Clenil Modulite 200micrograms/dose inhaler (Chiesi Ltd) |
| 5119 | CorticosteroidsInhaled | 10619611000001102 | Clenil Modulite 250micrograms/dose inhaler (Chiesi Ltd) |
| 5119 | CorticosteroidsInhaled | 10621011000001101 | Beclometasone 200micrograms/dose inhaler CFC free |
| 5119 | CorticosteroidsInhaled | 10621111000001100 | Beclometasone 250micrograms/dose inhaler CFC free |
| 5119 | CorticosteroidsInhaled | 10785011000001101 | Pulmicort 1mg Respules (Waymade Healthcare Plc) |
| 5119 | CorticosteroidsInhaled | 10834011000001106 | Flixotide 2mg/2ml Nebules (Waymade Healthcare Plc) |
| 5119 | CorticosteroidsInhaled | 10835911000001109 | Flixotide 25micrograms/dose inhaler (Waymade Healthcare Plc) |
| 5119 | CorticosteroidsInhaled | 10838111000001101 | Becodisks 200microgram (Waymade Healthcare Plc) |
| 5119 | CorticosteroidsInhaled | 10838311000001104 | Becodisks 400microgram (Waymade Healthcare Plc) |
| 5119 | CorticosteroidsInhaled | 10844511000001104 | Becotide 200 inhaler (Waymade Healthcare Plc) |
| 5119 | CorticosteroidsInhaled | 10855011000001109 | Flixotide 50micrograms/dose Evohaler (Waymade Healthcare Plc) |
| 5119 | CorticosteroidsInhaled | 10983311000001107 | Symbicort 100/6 Turbohaler (Waymade Healthcare Plc) |
| 5119 | CorticosteroidsInhaled | 11005511000001104 | Budesonide 500micrograms/2ml nebuliser liquid unit dose vials (Accord Healthcare Ltd) |
| 5119 | CorticosteroidsInhaled | 11005711000001109 | Budesonide 1mg/2ml nebuliser liquid unit dose vials (Accord Healthcare Ltd) |
| 5119 | CorticosteroidsInhaled | 11400011000001108 | Beclometasone 50micrograms/dose inhaler (Almus Pharmaceuticals Ltd) |

| Con ditio nID | Condition name | DMD code | Description |
|---|---|---|---|
| 5119 | CorticosteroidsInhaled | 11400511000001100 | Beclometasone 100micrograms/dose inhaler (Almus Pharmaceuticals Ltd) |
| 5119 | CorticosteroidsInhaled | 11400811000001102 | Beclometasone 250micrograms/dose inhaler (Almus Pharmaceuticals Ltd) |
| 5119 | CorticosteroidsInhaled | 12888311000001102 | Flixotide 0.5mg/2ml Nebules (Waymade Healthcare Plc) |
| 5119 | CorticosteroidsInhaled | 12906411000001100 | Fostair 100micrograms/dose / 6micrograms/dose inhaler (Chiesi Ltd) |
| 5119 | CorticosteroidsInhaled | 12911011000001100 | Beclometasone 100micrograms/dose / Formoterol 6micrograms/dose inhaler CFC free |
| 5119 | CorticosteroidsInhaled | 13132801000001101 | Budesonide 200micrograms/dose / Formoterol 6micrograms/dose dry powder inhaler |
| 5119 | CorticosteroidsInhaled | 13162101000001100 | Fluticasone 50micrograms/dose / Salmeterol 25micrograms/dose inhaler CFC free |
| 5119 | CorticosteroidsInhaled | 13162201000001107 | Fluticasone 125micrograms/dose / Salmeterol 25micrograms/dose inhaler CFC free |
| 5119 | CorticosteroidsInhaled | 13162301000001103 | Fluticasone 250micrograms/dose / Salmeterol 25micrograms/dose inhaler CFC free |
| 5119 | CorticosteroidsInhaled | 13162401000001106 | Fluticasone propionate 100micrograms/dose / Salmeterol 50micrograms/dose dry powder inhaler |
| 5119 | CorticosteroidsInhaled | 13162501000001105 | Fluticasone propionate 250micrograms/dose / Salmeterol 50micrograms/dose dry powder inhaler |
| 5119 | CorticosteroidsInhaled | 13162601000001109 | Fluticasone propionate 500micrograms/dose / Salmeterol 50micrograms/dose dry powder inhaler |
| 5119 | CorticosteroidsInhaled | 13164801000001101 | Beclometasone 400microgram inhalation powder blisters with device |
| 5119 | CorticosteroidsInhaled | 13164901000001107 | Beclometasone 400microgram inhalation powder blisters |
| 5119 | CorticosteroidsInhaled | 13165001000001107 | Beclometasone 50micrograms/dose breath actuated inhaler |
| 5119 | CorticosteroidsInhaled | 13165101000001108 | Beclometasone 250micrograms/dose breath actuated inhaler |
| 5119 | CorticosteroidsInhaled | 13165201000001101 | Beclometasone 100micrograms/dose breath actuated inhaler |
| 5119 | CorticosteroidsInhaled | 13165301000001105 | Beclometasone 100microgram inhalation powder blisters with device |
| 5119 | CorticosteroidsInhaled | 13165401000001102 | Beclometasone 200microgram inhalation powder blisters with device |
| 5119 | CorticosteroidsInhaled | 13165501000001103 | Beclometasone 100microgram inhalation powder blisters |
| 5119 | CorticosteroidsInhaled | 13165601000001104 | Beclometasone 200microgram inhalation powder blisters |
| 5119 | CorticosteroidsInhaled | 13166001000001102 | Salbutamol 100micrograms/dose / Beclometasone 50micrograms/dose inhaler |
| 5119 | CorticosteroidsInhaled | 13166401000001107 | Budesonide 200micrograms/dose inhaler |
| 5119 | CorticosteroidsInhaled | 13166501000001106 | Budesonide 50micrograms/dose inhaler |
| 5119 | CorticosteroidsInhaled | 13166601000001105 | Fluticasone propionate 50microgram inhalation powder blisters with device |
| 5119 | CorticosteroidsInhaled | 13166701000001100 | Fluticasone propionate 100microgram inhalation powder blisters with device |
| 5119 | CorticosteroidsInhaled | 13166801000001109 | Fluticasone propionate 250microgram inhalation powder blisters with device |
| 5119 | CorticosteroidsInhaled | 13166901000001103 | Fluticasone propionate 50microgram inhalation powder blisters |
| 5119 | CorticosteroidsInhaled | 13167001000001104 | Fluticasone propionate 100microgram inhalation powder blisters |
| 5119 | CorticosteroidsInhaled | 13167101000001103 | Fluticasone propionate 250microgram inhalation powder blisters |
| 5119 | CorticosteroidsInhaled | 13167301000001101 | Fluticasone propionate 500microgram inhalation powder blisters with device |
| 5119 | CorticosteroidsInhaled | 13167401000001109 | Fluticasone propionate 500microgram inhalation powder blisters |
| 5119 | CorticosteroidsInhaled | 13167501000001108 | Fluticasone propionate 50micrograms/dose dry powder inhaler |
| 5119 | CorticosteroidsInhaled | 13167601000001107 | Fluticasone propionate 100micrograms/dose dry powder inhaler |
| 5119 | CorticosteroidsInhaled | 13167801000001106 | Fluticasone propionate 500micrograms/dose dry powder inhaler |
| 5119 | CorticosteroidsInhaled | 13167901000001100 | Fluticasone 125micrograms/dose inhaler CFC free |
| 5119 | CorticosteroidsInhaled | 13168001000001103 | Fluticasone 250micrograms/dose inhaler CFC free |
| 5119 | CorticosteroidsInhaled | 13168101000001102 | Fluticasone 50micrograms/dose inhaler CFC free |
| 5119 | CorticosteroidsInhaled | 13180411000001105 | Beclazone 100 Easi-Breathe inhaler (Dowelhurst Ltd) |
| 5119 | CorticosteroidsInhaled | 13180811000001107 | Beclazone 250 Easi-Breathe inhaler (Dowelhurst Ltd) |

| Con ditio nID | Condition name | DMD code | Description |
|---|---|---|---|
| 5119 | CorticosteroidsInhaled | 13181011000001105 | Beclazone 50 Easi-Breathe inhaler (Dowelhurst Ltd) |
| 5119 | CorticosteroidsInhaled | 13186411000001107 | Budesonide 1mg/2ml nebuliser liquid unit dose vials (Dowelhurst Ltd) |
| 5119 | CorticosteroidsInhaled | 13186811000001109 | Budesonide 500micrograms/2ml nebuliser liquid unit dose vials (Dowelhurst Ltd) |
| 5119 | CorticosteroidsInhaled | 13206411000001106 | Symbicort 100/6 Turbohaler (Dowelhurst Ltd) |
| 5119 | CorticosteroidsInhaled | 13206611000001109 | Symbicort 400/12 Turbohaler (Dowelhurst Ltd) |
| 5119 | CorticosteroidsInhaled | 13261601000001100 | Mometasone 400micrograms/dose dry powder inhaler |
| 5119 | CorticosteroidsInhaled | 13261701000001105 | Mometasone 200micrograms/dose dry powder inhaler |
| 5119 | CorticosteroidsInhaled | 13262201000001105 | Beclometasone 50micrograms/dose breath actuated inhaler CFC free |
| 5119 | CorticosteroidsInhaled | 13262401000001109 | Beclometasone 100micrograms/dose breath actuated inhaler CFC free |
| 5119 | CorticosteroidsInhaled | 13841811000001102 | Flixotide 50micrograms/dose Accuhaler (Waymade Healthcare Plc) |
| 5119 | CorticosteroidsInhaled | 13876511000001105 | Flixotide 100micrograms/dose Accuhaler (DE Pharmaceuticals) |
| 5119 | CorticosteroidsInhaled | 13876711000001100 | Flixotide 250micrograms/dose Accuhaler (DE Pharmaceuticals) |
| 5119 | CorticosteroidsInhaled | 13876911000001103 | Flixotide 500micrograms/dose Accuhaler (DE Pharmaceuticals) |
| 5119 | CorticosteroidsInhaled | 13877111000001103 | Flixotide 250microgram disks (DE Pharmaceuticals) |
| 5119 | CorticosteroidsInhaled | 13877311000001101 | Flixotide 500microgram disks (DE Pharmaceuticals) |
| 5119 | CorticosteroidsInhaled | 13877511000001107 | Flixotide 125micrograms/dose Evohaler (DE Pharmaceuticals) |
| 5119 | CorticosteroidsInhaled | 13877711000001102 | Flixotide 250micrograms/dose Evohaler (DE Pharmaceuticals) |
| 5119 | CorticosteroidsInhaled | 13878311000001100 | Flixotide 0.5mg/2ml Nebules (DE Pharmaceuticals) |
| 5119 | CorticosteroidsInhaled | 13878511000001106 | Flixotide 2mg/2ml Nebules (DE Pharmaceuticals) |
| 5119 | CorticosteroidsInhaled | 13952211000001103 | Pulmicort 0.5mg Respules (DE Pharmaceuticals) |
| 5119 | CorticosteroidsInhaled | 13952511000001100 | Pulmicort 1mg Respules (DE Pharmaceuticals) |
| 5119 | CorticosteroidsInhaled | 13952811000001102 | Pulmicort 100 Turbohaler (DE Pharmaceuticals) |
| 5119 | CorticosteroidsInhaled | 13953111000001103 | Pulmicort 200 Turbohaler (DE Pharmaceuticals) |
| 5119 | CorticosteroidsInhaled | 13953611000001106 | Pulmicort 400 Turbohaler (DE Pharmaceuticals) |
| 5119 | CorticosteroidsInhaled | 13954711000001100 | Qvar 100 inhaler (DE Pharmaceuticals) |
| 5119 | CorticosteroidsInhaled | 13958011000001106 | Symbicort 100/6 Turbohaler (DE Pharmaceuticals) |
| 5119 | CorticosteroidsInhaled | 13958611000001104 | Symbicort 200/6 Turbohaler (DE Pharmaceuticals) |
| 5119 | CorticosteroidsInhaled | 13959211000001106 | Symbicort 400/12 Turbohaler (DE Pharmaceuticals) |
| 5119 | CorticosteroidsInhaled | 13961211000001104 | Pulmicort LS 50micrograms/dose inhaler (DE Pharmaceuticals) |
| 5119 | CorticosteroidsInhaled | 13997111000001100 | Seretide 100 Accuhaler (DE Pharmaceuticals) |
| 5119 | CorticosteroidsInhaled | 13997511000001109 | Seretide 250 Accuhaler (DE Pharmaceuticals) |
| 5119 | CorticosteroidsInhaled | 13998111000001104 | Seretide 125 Evohaler (DE Pharmaceuticals) |
| 5119 | CorticosteroidsInhaled | 13998411000001109 | Seretide 250 Evohaler (DE Pharmaceuticals) |
| 5119 | CorticosteroidsInhaled | 14233711000001100 | Flixotide 100micrograms/dose Accuhaler (Sigma Pharmaceuticals Plc) |
| 5119 | CorticosteroidsInhaled | 14233911000001103 | Flixotide 500micrograms/dose Accuhaler (Sigma Pharmaceuticals Plc) |
| 5119 | CorticosteroidsInhaled | 14234111000001104 | Flixotide 250micrograms/dose Accuhaler (Sigma Pharmaceuticals Plc) |
| 5119 | CorticosteroidsInhaled | 14234311000001102 | Flixotide 50micrograms/dose Accuhaler (Sigma Pharmaceuticals Plc) |
| 5119 | CorticosteroidsInhaled | 14234511000001108 | Flixotide 125micrograms/dose Evohaler (Sigma Pharmaceuticals Plc) |
| 5119 | CorticosteroidsInhaled | 14234711000001103 | Flixotide 250micrograms/dose Evohaler (Sigma Pharmaceuticals Plc) |
| 5119 | CorticosteroidsInhaled | 14234911000001101 | Flixotide 0.5mg/2ml Nebules (Sigma Pharmaceuticals Plc) |
| 5119 | CorticosteroidsInhaled | 14235111000001100 | Flixotide 2mg/2ml Nebules (Sigma Pharmaceuticals Plc) |
| 5119 | CorticosteroidsInhaled | 14235311000001103 | Flixotide 100microgram disks (Sigma Pharmaceuticals Plc) |
| 5119 | CorticosteroidsInhaled | 14235511000001109 | Flixotide 250microgram disks (Sigma Pharmaceuticals Plc) |

| Con ditio nID | Condition name | DMD code | Description |
|---|---|---|---|
| 5119 | CorticosteroidsInhaled | 14235711000001104 | Flixotide 500microgram disks (Sigma Pharmaceuticals Plc) |
| 5119 | CorticosteroidsInhaled | 14238811000001103 | Fluticasone 250micrograms/dose Evohaler (Sigma Pharmaceuticals Plc) |
| 5119 | CorticosteroidsInhaled | 14382711000001109 | Pulmicort 1mg Respules (Sigma Pharmaceuticals Plc) |
| 5119 | CorticosteroidsInhaled | 14386211000001101 | Pulmicort 0.5mg Respules (Sigma Pharmaceuticals Plc) |
| 5119 | CorticosteroidsInhaled | 14387611000001102 | Pulmicort 400 Turbohaler (Sigma Pharmaceuticals Plc) |
| 5119 | CorticosteroidsInhaled | 14390211000001104 | Pulmicort 100 Turbohaler (Sigma Pharmaceuticals Plc) |
| 5119 | CorticosteroidsInhaled | 14390411000001100 | Pulmicort 200 Turbohaler (Sigma Pharmaceuticals Plc) |
| 5119 | CorticosteroidsInhaled | 14616311000001102 | Qvar 100 Autohaler (Sigma Pharmaceuticals Plc) |
| 5119 | CorticosteroidsInhaled | 14616611000001107 | Qvar 100 inhaler (Sigma Pharmaceuticals Plc) |
| 5119 | CorticosteroidsInhaled | 14620511000001104 | Symbicort 100/6 Turbohaler (Sigma Pharmaceuticals Plc) |
| 5119 | CorticosteroidsInhaled | 14620711000001109 | Symbicort 200/6 Turbohaler (Sigma Pharmaceuticals Plc) |
| 5119 | CorticosteroidsInhaled | 14621111000001102 | Symbicort 400/12 Turbohaler (Sigma Pharmaceuticals Plc) |
| 5119 | CorticosteroidsInhaled | 14674111000001100 | Seretide 100 Accuhaler (Sigma Pharmaceuticals Plc) |
| 5119 | CorticosteroidsInhaled | 14674711000001104 | Seretide 250 Accuhaler (Sigma Pharmaceuticals Plc) |
| 5119 | CorticosteroidsInhaled | 14705211000001107 | Seretide 500 Accuhaler (Sigma Pharmaceuticals Plc) |
| 5119 | CorticosteroidsInhaled | 14705411000001106 | Seretide 125 Evohaler (Sigma Pharmaceuticals Plc) |
| 5119 | CorticosteroidsInhaled | 14705811000001108 | Seretide 250 Evohaler (Sigma Pharmaceuticals Plc) |
| 5119 | CorticosteroidsInhaled | 14755711000001106 | Qvar 100 Autohaler (Waymade Healthcare Plc) |
| 5119 | CorticosteroidsInhaled | 14951111000001102 | Pulmicort 100micrograms/dose inhaler CFC free (AstraZeneca UK Ltd) |
| 5119 | CorticosteroidsInhaled | 14959511000001107 | Budesonide 100micrograms/dose inhaler CFC free |
| 5119 | CorticosteroidsInhaled | 15052211000001104 | Beclometasone 200micrograms/dose inhaler (Sigma Pharmaceuticals Plc) |
| 5119 | CorticosteroidsInhaled | 15060511000001109 | Budesonide 1mg/2ml nebuliser liquid unit dose vials (Sigma Pharmaceuticals Plc) |
| 5119 | CorticosteroidsInhaled | 15358411000001102 | Pulmicort 200micrograms/dose inhaler CFC free (AstraZeneca UK Ltd) |
| 5119 | CorticosteroidsInhaled | 15374611000001106 | Budesonide 200micrograms/dose inhaler CFC free |
| 5119 | CorticosteroidsInhaled | 15418911000001106 | Qvar 100micrograms/dose Easi-Breathe inhaler (Waymade Healthcare Plc) |
| 5119 | CorticosteroidsInhaled | 15884311000001105 | Qvar 100 inhaler (Waymade Healthcare Plc) |
| 5119 | CorticosteroidsInhaled | 16140411000001105 | Becodisks 200microgram (Lexon (UK) Ltd) |
| 5119 | CorticosteroidsInhaled | 16140911000001102 | Becodisks 400microgram (Lexon (UK) Ltd) |
| 5119 | CorticosteroidsInhaled | 16179611000001103 | Flixotide 250micrograms/dose Accuhaler (Lexon (UK) Ltd) |
| 5119 | CorticosteroidsInhaled | 16179811000001104 | Flixotide 500micrograms/dose Accuhaler (Lexon (UK) Ltd) |
| 5119 | CorticosteroidsInhaled | 16180011000001105 | Flixotide 125micrograms/dose Evohaler (Lexon (UK) Ltd) |
| 5119 | CorticosteroidsInhaled | 16180211000001100 | Flixotide 250micrograms/dose Evohaler (Lexon (UK) Ltd) |
| 5119 | CorticosteroidsInhaled | 16180411000001101 | Flixotide 0.5mg/2ml Nebules (Lexon (UK) Ltd) |
| 5119 | CorticosteroidsInhaled | 16180611000001103 | Flixotide 2mg/2ml Nebules (Lexon (UK) Ltd) |
| 5119 | CorticosteroidsInhaled | 16230011000001102 | Pulmicort 100 Turbohaler (Lexon (UK) Ltd) |
| 5119 | CorticosteroidsInhaled | 16230211000001107 | Pulmicort 200 Turbohaler (Lexon (UK) Ltd) |
| 5119 | CorticosteroidsInhaled | 16230511000001105 | Pulmicort 400 Turbohaler (Lexon (UK) Ltd) |
| 5119 | CorticosteroidsInhaled | 16230911000001103 | Pulmicort 0.5mg Respules (Lexon (UK) Ltd) |
| 5119 | CorticosteroidsInhaled | 16231411000001102 | Pulmicort 1mg Respules (Lexon (UK) Ltd) |
| 5119 | CorticosteroidsInhaled | 16232011000001103 | Qvar 100 Autohaler (Lexon (UK) Ltd) |
| 5119 | CorticosteroidsInhaled | 16240811000001109 | Seretide 100 Accuhaler (Lexon (UK) Ltd) |
| 5119 | CorticosteroidsInhaled | 16241011000001107 | Seretide 250 Accuhaler (Lexon (UK) Ltd) |
| 5119 | CorticosteroidsInhaled | 16241111000001108 | Seretide 500 Accuhaler (Lexon (UK) Ltd) |

| Con ditio nID | Condition name | DMD code | Description |
|---|---|---|---|
| 5119 | CorticosteroidsInhaled | 16241311000001105 | Seretide 125 Evohaler (Lexon (UK) Ltd) |
| 5119 | CorticosteroidsInhaled | 16241511000001104 | Seretide 250 Evohaler (Lexon (UK) Ltd) |
| 5119 | CorticosteroidsInhaled | 16277711000001101 | Becodisks 200microgram (Mawdsley-Brooks & Company Ltd) |
| 5119 | CorticosteroidsInhaled | 16278211000001107 | Becodisks 400microgram (Mawdsley-Brooks & Company Ltd) |
| 5119 | CorticosteroidsInhaled | 16545111000001101 | Flixotide 250micrograms/dose Accuhaler (Stephar (U.K.) Ltd) |
| 5119 | CorticosteroidsInhaled | 16545311000001104 | Flixotide 125micrograms/dose Evohaler (Stephar (U.K.) Ltd) |
| 5119 | CorticosteroidsInhaled | 16545511000001105 | Flixotide 250micrograms/dose Evohaler (Stephar (U.K.) Ltd) |
| 5119 | CorticosteroidsInhaled | 16579011000001104 | Flixotide 50micrograms/dose Accuhaler (Mawdsley-Brooks & Company Ltd) |
| 5119 | CorticosteroidsInhaled | 16579211000001109 | Flixotide 100micrograms/dose Accuhaler (Mawdsley-Brooks & Company Ltd) |
| 5119 | CorticosteroidsInhaled | 16579411000001108 | Flixotide 250micrograms/dose Accuhaler (Mawdsley-Brooks & Company Ltd) |
| 5119 | CorticosteroidsInhaled | 16579611000001106 | Flixotide 500micrograms/dose Accuhaler (Mawdsley-Brooks & Company Ltd) |
| 5119 | CorticosteroidsInhaled | 16579811000001105 | Flixotide 125micrograms/dose Evohaler (Mawdsley-Brooks & Company Ltd) |
| 5119 | CorticosteroidsInhaled | 16580011000001104 | Flixotide 250micrograms/dose Evohaler (Mawdsley-Brooks & Company Ltd) |
| 5119 | CorticosteroidsInhaled | 16580211000001109 | Flixotide 0.5mg/2ml Nebules (Mawdsley-Brooks & Company Ltd) |
| 5119 | CorticosteroidsInhaled | 16580411000001108 | Flixotide 2mg/2ml Nebules (Mawdsley-Brooks & Company Ltd) |
| 5119 | CorticosteroidsInhaled | 16581211000001103 | Fluticasone 250micrograms/dose Evohaler (Mawdsley-Brooks & Company Ltd) |
| 5119 | CorticosteroidsInhaled | 16586711000001104 | Pulmicort 400 Turbohaler (Stephar (U.K.) Ltd) |
| 5119 | CorticosteroidsInhaled | 16587011000001103 | Pulmicort 0.5mg Respules (Stephar (U.K.) Ltd) |
| 5119 | CorticosteroidsInhaled | 16587211000001108 | Pulmicort 1mg Respules (Stephar (U.K.) Ltd) |
| 5119 | CorticosteroidsInhaled | 16587911000001104 | Seretide 100 Accuhaler (Stephar (U.K.) Ltd) |
| 5119 | CorticosteroidsInhaled | 16588111000001101 | Seretide 250 Evohaler (Stephar (U.K.) Ltd) |
| 5119 | CorticosteroidsInhaled | 16728511000001101 | Seretide 50 Evohaler (Waymade Healthcare Plc) |
| 5119 | CorticosteroidsInhaled | 17425011000001106 | Pulmicort 0.5mg Respules (Mawdsley-Brooks & Company Ltd) |
| 5119 | CorticosteroidsInhaled | 17425311000001109 | Pulmicort 1mg Respules (Mawdsley-Brooks & Company Ltd) |
| 5119 | CorticosteroidsInhaled | 17425611000001104 | Pulmicort 100 Turbohaler (Mawdsley-Brooks & Company Ltd) |
| 5119 | CorticosteroidsInhaled | 17425811000001100 | Pulmicort 200 Turbohaler (Mawdsley-Brooks & Company Ltd) |
| 5119 | CorticosteroidsInhaled | 17426011000001102 | Pulmicort 400 Turbohaler (Mawdsley-Brooks & Company Ltd) |
| 5119 | CorticosteroidsInhaled | 17426811000001108 | Qvar 50 inhaler (Mawdsley-Brooks & Company Ltd) |
| 5119 | CorticosteroidsInhaled | 17427011000001104 | Qvar 100 inhaler (Mawdsley-Brooks & Company Ltd) |
| 5119 | CorticosteroidsInhaled | 17427211000001109 | Qvar 100 Autohaler (Mawdsley-Brooks & Company Ltd) |
| 5119 | CorticosteroidsInhaled | 17440111000001102 | Seretide 100 Accuhaler (Mawdsley-Brooks & Company Ltd) |
| 5119 | CorticosteroidsInhaled | 17440311000001100 | Seretide 250 Accuhaler (Mawdsley-Brooks & Company Ltd) |
| 5119 | CorticosteroidsInhaled | 17440511000001106 | Seretide 500 Accuhaler (Mawdsley-Brooks & Company Ltd) |
| 5119 | CorticosteroidsInhaled | 17440711000001101 | Seretide 125 Evohaler (Mawdsley-Brooks & Company Ltd) |
| 5119 | CorticosteroidsInhaled | 17440911000001104 | Seretide 250 Evohaler (Mawdsley-Brooks & Company Ltd) |
| 5119 | CorticosteroidsInhaled | 17457311000001102 | Symbicort 100/6 Turbohaler (Mawdsley-Brooks & Company Ltd) |
| 5119 | CorticosteroidsInhaled | 17457611000001107 | Symbicort 200/6 Turbohaler (Mawdsley-Brooks & Company Ltd) |
| 5119 | CorticosteroidsInhaled | 17457811000001106 | Symbicort 400/12 Turbohaler (Mawdsley-Brooks & Company Ltd) |
| 5119 | CorticosteroidsInhaled | 17490511000001100 | Alvesco 160 inhaler (Mawdsley-Brooks & Company Ltd) |
| 5119 | CorticosteroidsInhaled | 17505711000001109 | Flixotide 50micrograms/dose Evohaler (Mawdsley-Brooks & Company Ltd) |
| 5119 | CorticosteroidsInhaled | 17600111000001105 | Becodisks 200microgram (Necessity Supplies Ltd) |

| ConditionID | Condition name | DMD code | Description |
|---|---|---|---|
| 5119 | CorticosteroidsInhaled | 17600311000001107 | Becodisks 400microgram (Necessity Supplies Ltd) |
| 5119 | CorticosteroidsInhaled | 17793411000001101 | Beclometasone 50micrograms/dose inhaler (Phoenix Healthcare Distribution Ltd) |
| 5119 | CorticosteroidsInhaled | 17794111000001108 | Beclometasone 100micrograms/dose inhaler (Phoenix Healthcare Distribution Ltd) |
| 5119 | CorticosteroidsInhaled | 17809311000001106 | Budesonide 500micrograms/2ml nebuliser liquid unit dose vials (Phoenix Healthcare Distribution Ltd) |
| 5119 | CorticosteroidsInhaled | 17810011000001102 | Budesonide 1mg/2ml nebuliser liquid unit dose vials (Phoenix Healthcare Distribution Ltd) |
| 5119 | CorticosteroidsInhaled | 17833911000001100 | Beclometasone 250micrograms/dose inhaler (Phoenix Healthcare Distribution Ltd) |
| 5119 | CorticosteroidsInhaled | 18050311000001104 | Alvesco 160 inhaler (Lexon (UK) Ltd) |
| 5119 | CorticosteroidsInhaled | 18058211000001105 | Flixotide 50micrograms/dose Evohaler (Lexon (UK) Ltd) |
| 5119 | CorticosteroidsInhaled | 18058811000001106 | Qvar 100micrograms/dose Easi-Breathe inhaler (Sigma Pharmaceuticals Plc) |
| 5119 | CorticosteroidsInhaled | 18059011000001105 | Qvar 50micrograms/dose Easi-Breathe inhaler (Sigma Pharmaceuticals Plc) |
| 5119 | CorticosteroidsInhaled | 18174511000001109 | Pulmicort 1mg Respules (Necessity Supplies Ltd) |
| 5119 | CorticosteroidsInhaled | 18174711000001104 | Pulmicort 0.5mg Respules (Necessity Supplies Ltd) |
| 5119 | CorticosteroidsInhaled | 18174911000001102 | Pulmicort 100 Turbohaler (Necessity Supplies Ltd) |
| 5119 | CorticosteroidsInhaled | 18175111000001101 | Pulmicort 200 Turbohaler (Necessity Supplies Ltd) |
| 5119 | CorticosteroidsInhaled | 18175311000001104 | Pulmicort 400 Turbohaler (Necessity Supplies Ltd) |
| 5119 | CorticosteroidsInhaled | 18185011000001106 | Seretide 100 Accuhaler (Necessity Supplies Ltd) |
| 5119 | CorticosteroidsInhaled | 18185211000001101 | Seretide 250 Accuhaler (Necessity Supplies Ltd) |
| 5119 | CorticosteroidsInhaled | 18185411000001102 | Seretide 500 Accuhaler (Necessity Supplies Ltd) |
| 5119 | CorticosteroidsInhaled | 18185611000001104 | Seretide 125 Evohaler (Necessity Supplies Ltd) |
| 5119 | CorticosteroidsInhaled | 18185811000001100 | Seretide 250 Evohaler (Necessity Supplies Ltd) |
| 5119 | CorticosteroidsInhaled | 18195111000001106 | Symbicort 100/6 Turbohaler (Necessity Supplies Ltd) |
| 5119 | CorticosteroidsInhaled | 18195311000001108 | Symbicort 200/6 Turbohaler (Necessity Supplies Ltd) |
| 5119 | CorticosteroidsInhaled | 18195511000001102 | Symbicort 400/12 Turbohaler (Necessity Supplies Ltd) |
| 5119 | CorticosteroidsInhaled | 18261611000001102 | Flixotide 100micrograms/dose Accuhaler (Necessity Supplies Ltd) |
| 5119 | CorticosteroidsInhaled | 18261811000001103 | Flixotide 250micrograms/dose Accuhaler (Necessity Supplies Ltd) |
| 5119 | CorticosteroidsInhaled | 18262011000001101 | Flixotide 500micrograms/dose Accuhaler (Necessity Supplies Ltd) |
| 5119 | CorticosteroidsInhaled | 18262211000001106 | Flixotide 125micrograms/dose Evohaler (Necessity Supplies Ltd) |
| 5119 | CorticosteroidsInhaled | 18262411000001105 | Flixotide 250micrograms/dose Evohaler (Necessity Supplies Ltd) |
| 5119 | CorticosteroidsInhaled | 18262611000001108 | Flixotide 250microgram disks (Necessity Supplies Ltd) |
| 5119 | CorticosteroidsInhaled | 18263011000001105 | Flixotide 500microgram disks (Necessity Supplies Ltd) |
| 5119 | CorticosteroidsInhaled | 18295511000001100 | Qvar 100 Autohaler (Stephar (U.K.) Ltd) |
| 5119 | CorticosteroidsInhaled | 18507011000001106 | Budesonide 500micrograms/2ml nebuliser liquid unit dose vials (Almus Pharmaceuticals Ltd) |
| 5119 | CorticosteroidsInhaled | 18507211000001101 | Budesonide 1mg/2ml nebuliser liquid unit dose vials (Almus Pharmaceuticals Ltd) |
| 5119 | CorticosteroidsInhaled | 18579211000001101 | Flixotide 50micrograms/dose Evohaler (DE Pharmaceuticals) |
| 5119 | CorticosteroidsInhaled | 18608111000001104 | Qvar 100 Autohaler (DE Pharmaceuticals) |
| 5119 | CorticosteroidsInhaled | 18608311000001102 | Qvar 50 Autohaler (DE Pharmaceuticals) |
| 5119 | CorticosteroidsInhaled | 18608511000001108 | Qvar 100micrograms/dose Easi-Breathe inhaler (DE Pharmaceuticals) |
| 5119 | CorticosteroidsInhaled | 18608711000001103 | Qvar 50micrograms/dose Easi-Breathe inhaler (DE Pharmaceuticals) |
| 5119 | CorticosteroidsInhaled | 18608911000001101 | Qvar 50 inhaler (DE Pharmaceuticals) |
| 5119 | CorticosteroidsInhaled | 18611411000001104 | Seretide 500 Accuhaler (DE Pharmaceuticals) |
| 5119 | CorticosteroidsInhaled | 19612611000001105 | Flixotide 50micrograms/dose Accuhaler (DE Pharmaceuticals) |

| Con ditio nID | Condition name | DMD code | Description |
|---|---|---|---|
| 5119 | CorticosteroidsInhaled | 19726711000001101 | Alvesco 80 inhaler (Mawdsley-Brooks & Company Ltd) |
| 5119 | CorticosteroidsInhaled | 19727911000001101 | Asmanex 400micrograms/dose Twisthaler (Mawdsley-Brooks & Company Ltd) |
| 5119 | CorticosteroidsInhaled | 19737111000001101 | Clenil Modulite 50micrograms/dose inhaler (Mawdsley-Brooks & Company Ltd) |
| 5119 | CorticosteroidsInhaled | 19737311000001104 | Clenil Modulite 100micrograms/dose inhaler (Mawdsley-Brooks & Company Ltd) |
| 5119 | CorticosteroidsInhaled | 19737511000001105 | Clenil Modulite 200micrograms/dose inhaler (Mawdsley-Brooks & Company Ltd) |
| 5119 | CorticosteroidsInhaled | 19737811000001108 | Clenil Modulite 250micrograms/dose inhaler (Mawdsley-Brooks & Company Ltd) |
| 5119 | CorticosteroidsInhaled | 19965011000001108 | Flixotide 100micrograms/dose Accuhaler (Lexon (UK) Ltd) |
| 5119 | CorticosteroidsInhaled | 21019411000001101 | Flutiform 125micrograms/dose / 5micrograms/dose inhaler (Napp Pharmaceuticals Ltd) |
| 5119 | CorticosteroidsInhaled | 21019711000001107 | Flutiform 250micrograms/dose / 10micrograms/dose inhaler (Napp Pharmaceuticals Ltd) |
| 5119 | CorticosteroidsInhaled | 21020611000001104 | Flutiform 50micrograms/dose / 5micrograms/dose inhaler (Napp Pharmaceuticals Ltd) |
| 5119 | CorticosteroidsInhaled | 21113711000001102 | Fluticasone 125micrograms/dose / Formoterol 5micrograms/dose inhaler CFC free |
| 5119 | CorticosteroidsInhaled | 21113811000001105 | Fluticasone 250micrograms/dose / Formoterol 10micrograms/dose inhaler CFC free |
| 5119 | CorticosteroidsInhaled | 21113911000001100 | Fluticasone 50micrograms/dose / Formoterol 5micrograms/dose inhaler CFC free |
| 5119 | CorticosteroidsInhaled | 21690711000001109 | Alvesco 160 inhaler (Waymade Healthcare Plc) |
| 5119 | CorticosteroidsInhaled | 21804411000001104 | Budesonide 500micrograms/2ml nebuliser liquid unit dose vials (Waymade Healthcare Plc) |
| 5119 | CorticosteroidsInhaled | 21804611000001101 | Budesonide 1mg/2ml nebuliser liquid unit dose vials (Waymade Healthcare Plc) |
| 5119 | CorticosteroidsInhaled | 22622411000001105 | Budesonide 500micrograms/2ml nebuliser liquid unit dose vials (DE Pharmaceuticals) |
| 5119 | CorticosteroidsInhaled | 22622611000001108 | Budesonide 1mg/2ml nebuliser liquid unit dose vials (DE Pharmaceuticals) |
| 5119 | CorticosteroidsInhaled | 23438611000001104 | Alvesco 80 inhaler (Waymade Healthcare Plc) |
| 5119 | CorticosteroidsInhaled | 24591711000001104 | Clenil Modulite 200micrograms/dose inhaler (Waymade Healthcare Plc) |
| 5119 | CorticosteroidsInhaled | 24591911000001102 | Clenil Modulite 250micrograms/dose inhaler (Waymade Healthcare Plc) |
| 5119 | CorticosteroidsInhaled | 24660811000001107 | Beclometasone 100micrograms/dose inhaler CFC free (Ennogen Healthcare Ltd) |
| 5119 | CorticosteroidsInhaled | 24667411000001109 | Asmanex 200micrograms/dose Twisthaler (Waymade Healthcare Plc) |
| 5119 | CorticosteroidsInhaled | 25254111000001105 | DuoResp Spiromax 160micrograms/dose / 4.5micrograms/dose dry powder inhaler (Teva UK Ltd) |
| 5119 | CorticosteroidsInhaled | 25254711000001106 | DuoResp Spiromax 320micrograms/dose / 9micrograms/dose dry powder inhaler (Teva UK Ltd) |
| 5119 | CorticosteroidsInhaled | 26112111000001106 | Fostair NEXThaler 100micrograms/dose / 6micrograms/dose dry powder inhaler (Chiesi Ltd) |
| 5119 | CorticosteroidsInhaled | 26148711000001101 | Beclometasone 100micrograms/dose / Formoterol 6micrograms/dose dry powder inhaler |
| 5119 | CorticosteroidsInhaled | 27991211000001105 | Qvar 50micrograms/dose Easi-Breathe inhaler (Waymade Healthcare Plc) |
| 5119 | CorticosteroidsInhaled | 29525711000001109 | Beclometasone 50micrograms/dose inhaler CFC free (Colorama Pharmaceuticals Ltd) |
| 5119 | CorticosteroidsInhaled | 29526111000001102 | Beclometasone 100micrograms/dose inhaler CFC free (Colorama Pharmaceuticals Ltd) |
| 5119 | CorticosteroidsInhaled | 29526411000001107 | Beclometasone 200micrograms/dose inhaler CFC free (Colorama Pharmaceuticals Ltd) |
| 5119 | CorticosteroidsInhaled | 29749211000001101 | Flutiform 125micrograms/dose / 5micrograms/dose inhaler (Waymade Healthcare Plc) |
| 5119 | CorticosteroidsInhaled | 29749411000001102 | Flutiform 50micrograms/dose / 5micrograms/dose inhaler (Waymade Healthcare Plc) |
| 5119 | CorticosteroidsInhaled | 29749611000001104 | Flutiform 250micrograms/dose / 10micrograms/dose inhaler (Waymade Healthcare Plc) |
| 5119 | CorticosteroidsInhaled | 29775411000001101 | Budesonide 500micrograms/2ml nebuliser liquid unit dose vials (Sigma Pharmaceuticals Plc) |
| 5119 | CorticosteroidsInhaled | 29782111000001107 | Sirdupla 25micrograms/dose / 125micrograms/dose inhaler (Viatris UK Healthcare Ltd) |

| Con ditio nID | Condition name | DMD code | Description |
|---|---|---|---|
| 5119 | CorticosteroidsInhaled | 29782511000001103 | Sirdupla 25micrograms/dose / 250micrograms/dose inhaler (Viatris UK Healthcare Ltd) |
| 5119 | CorticosteroidsInhaled | 29959011000001102 | Alvesco 80 inhaler (Lexon (UK) Ltd) |
| 5119 | CorticosteroidsInhaled | 30004311000001102 | Budesonide 500micrograms/2ml nebuliser liquid unit dose vials (Mawdsley-Brooks & Company Ltd) |
| 5119 | CorticosteroidsInhaled | 30004511000001108 | Budesonide 1mg/2ml nebuliser liquid unit dose vials (Mawdsley-Brooks & Company Ltd) |
| 5119 | CorticosteroidsInhaled | 30253911000001100 | Sirdupla 25micrograms/dose / 250micrograms/dose inhaler (Waymade Healthcare Plc) |
| 5119 | CorticosteroidsInhaled | 30950311000001106 | AirFluSal Forspiro 50micrograms/dose / 500micrograms/dose dry powder inhaler (Sandoz Ltd) |
| 5119 | CorticosteroidsInhaled | 30950511000001100 | Beclometasone 100micrograms/dose inhaler CFC free (DE Pharmaceuticals) |
| 5119 | CorticosteroidsInhaled | 30950711000001105 | Beclometasone 200micrograms/dose inhaler CFC free (DE Pharmaceuticals) |
| 5119 | CorticosteroidsInhaled | 30950911000001107 | Beclometasone 50micrograms/dose inhaler CFC free (DE Pharmaceuticals) |
| 5119 | CorticosteroidsInhaled | 31063111000001106 | Fostair NEXThaler 200micrograms/dose / 6micrograms/dose dry powder inhaler (Chiesi Ltd) |
| 5119 | CorticosteroidsInhaled | 31063411000001101 | Fostair 200micrograms/dose / 6micrograms/dose inhaler (Chiesi Ltd) |
| 5119 | CorticosteroidsInhaled | 31064411000001103 | Fluticasone 125micrograms/dose / Salmeterol 25micrograms/dose inhaler CFC free (A A H Pharmaceuticals Ltd) |
| 5119 | CorticosteroidsInhaled | 31064611000001100 | Fluticasone 250micrograms/dose / Salmeterol 25micrograms/dose inhaler CFC free (A A H Pharmaceuticals Ltd) |
| 5119 | CorticosteroidsInhaled | 31064811000001101 | Fluticasone propionate 500micrograms/dose / Salmeterol 50micrograms/dose dry powder inhaler (A A H Pharmaceuticals Ltd) |
| 5119 | CorticosteroidsInhaled | 31087411000001106 | Beclometasone 200micrograms/dose / Formoterol 6micrograms/dose inhaler CFC free |
| 5119 | CorticosteroidsInhaled | 31087511000001105 | Beclometasone 200micrograms/dose / Formoterol 6micrograms/dose dry powder inhaler |
| 5119 | CorticosteroidsInhaled | 31352911000001106 | Beclometasone 100micrograms/dose inhaler CFC free (J M McGill Ltd) |
| 5119 | CorticosteroidsInhaled | 31353111000001102 | Beclometasone 200micrograms/dose inhaler CFC free (J M McGill Ltd) |
| 5119 | CorticosteroidsInhaled | 31353311000001100 | Beclometasone 50micrograms/dose inhaler CFC free (J M McGill Ltd) |
| 5119 | CorticosteroidsInhaled | 32199311000001102 | Beclometasone 100micrograms/dose inhaler CFC free (Niche Pharma Ltd) |
| 5119 | CorticosteroidsInhaled | 32199911000001101 | Beclometasone 200micrograms/dose inhaler CFC free (Niche Pharma Ltd) |
| 5119 | CorticosteroidsInhaled | 32200311000001106 | Beclometasone 50micrograms/dose inhaler CFC free (Niche Pharma Ltd) |
| 5119 | CorticosteroidsInhaled | 32926011000001100 | Symbicort 200micrograms/dose / 6micrograms/dose pressurised inhaler (AstraZeneca UK Ltd) |
| 5119 | CorticosteroidsInhaled | 32960711000001105 | Budesonide 200micrograms/dose / Formoterol 6micrograms/dose inhaler CFC free |
| 5119 | CorticosteroidsInhaled | 33679711000001103 | Aerivio Spiromax 50micrograms/dose / 500micrograms/dose dry powder inhaler (Teva UK Ltd) |
| 5119 | CorticosteroidsInhaled | 34023611000001101 | Sereflo 25micrograms/dose / 125micrograms/dose inhaler (Cipla EU Ltd) |
| 5119 | CorticosteroidsInhaled | 34023811000001102 | Sereflo 25micrograms/dose / 250micrograms/dose inhaler (Cipla EU Ltd) |
| 5119 | CorticosteroidsInhaled | 34215311000001107 | AirFluSal 25micrograms/dose / 125micrograms/dose inhaler (Sandoz Ltd) |
| 5119 | CorticosteroidsInhaled | 34215511000001101 | AirFluSal 25micrograms/dose / 250micrograms/dose inhaler (Sandoz Ltd) |
| 5119 | CorticosteroidsInhaled | 34675711000001103 | Aloflute 25micrograms/dose / 250micrograms/dose inhaler (Viatris UK Healthcare Ltd) |
| 5119 | CorticosteroidsInhaled | 34677011000001107 | Aloflute 25micrograms/dose / 125micrograms/dose inhaler (Viatris UK Healthcare Ltd) |
| 5119 | CorticosteroidsInhaled | 34681611000001100 | Trimbow 87micrograms/dose / 5micrograms/dose / 9micrograms/dose inhaler (Chiesi Ltd) |
| 5119 | CorticosteroidsInhaled | 34683311000001106 | Generic Trimbow 87micrograms/dose / 5micrograms/dose / 9micrograms/dose inhaler |
| 5119 | CorticosteroidsInhaled | 34812111000001106 | Fobumix Easyhaler 320micrograms/dose / 9micrograms/dose dry powder inhaler (Orion Pharma (UK) Ltd) |
| 5119 | CorticosteroidsInhaled | 34950311000001108 | Fobumix Easyhaler 160micrograms/dose / 4.5micrograms/dose dry powder inhaler (Orion Pharma (UK) Ltd) |
| 5119 | CorticosteroidsInhaled | 34950611000001103 | Fobumix Easyhaler 80micrograms/dose / 4.5micrograms/dose dry powder inhaler (Orion Pharma (UK) Ltd) |
| 5119 | CorticosteroidsInhaled | 35369511000001103 | Salbutamol 200microgram / Beclometasone 100microgram inhalation powder capsules |

| Con ditio nID | Condition name | DMD code | Description |
|---|---|---|---|
| 5119 | CorticosteroidsInhaled | 35430111000001100 | Kelhale 50micrograms/dose inhaler (Cipla EU Ltd) |
| 5119 | CorticosteroidsInhaled | 35430311000001103 | Kelhale 100micrograms/dose inhaler (Cipla EU Ltd) |
| 5119 | CorticosteroidsInhaled | 35515311000001106 | Fusacomb Easyhaler 50micrograms/dose / 500micrograms/dose dry powder inhaler (Orion Pharma (UK) Ltd) |
| 5119 | CorticosteroidsInhaled | 35515511000001100 | Fusacomb Easyhaler 50micrograms/dose / 250micrograms/dose dry powder inhaler (Orion Pharma (UK) Ltd) |
| 5119 | CorticosteroidsInhaled | 35594011000001105 | Combisal 25micrograms/dose / 50micrograms/dose inhaler (Aspire Pharma Ltd) |
| 5119 | CorticosteroidsInhaled | 35594211000001100 | Combisal 25micrograms/dose / 125micrograms/dose inhaler (Aspire Pharma Ltd) |
| 5119 | CorticosteroidsInhaled | 35594411000001101 | Combisal 25micrograms/dose / 250micrograms/dose inhaler (Aspire Pharma Ltd) |
| 5119 | CorticosteroidsInhaled | 35647311000001101 | Flutiform K-haler 125micrograms/dose / 5micrograms/dose breath actuated inhaler (Napp Pharmaceuticals Ltd) |
| 5119 | CorticosteroidsInhaled | 35647511000001107 | Fluticasone 125micrograms/dose / Formoterol 5micrograms/dose breath actuated inhaler CFC free |
| 5119 | CorticosteroidsInhaled | 35647611000001106 | Fluticasone 50micrograms/dose / Formoterol 5micrograms/dose breath actuated inhaler CFC free |
| 5119 | CorticosteroidsInhaled | 35650811000001109 | Flutiform K-haler 50micrograms/dose / 5micrograms/dose breath actuated inhaler (Napp Pharmaceuticals Ltd) |
| 5119 | CorticosteroidsInhaled | 35907611000001101 | Beclometasone 100microgram inhalation powder blisters |
| 5119 | CorticosteroidsInhaled | 35907711000001105 | Beclometasone 100microgram inhalation powder blisters with device |
| 5119 | CorticosteroidsInhaled | 35907811000001102 | Beclometasone 100micrograms/dose breath actuated inhaler |
| 5119 | CorticosteroidsInhaled | 35907911000001107 | Beclometasone 100micrograms/dose breath actuated inhaler CFC free |
| 5119 | CorticosteroidsInhaled | 35908011000001109 | Beclometasone 100micrograms/dose dry powder inhaler |
| 5119 | CorticosteroidsInhaled | 35908111000001105 | Beclometasone 100micrograms/dose inhaler CFC free |
| 5119 | CorticosteroidsInhaled | 35908211000001104 | Beclometasone 200microgram inhalation powder blisters |
| 5119 | CorticosteroidsInhaled | 35908311000001107 | Beclometasone 200microgram inhalation powder blisters with device |
| 5119 | CorticosteroidsInhaled | 35908411000001100 | Beclometasone 200micrograms/dose dry powder inhaler |
| 5119 | CorticosteroidsInhaled | 35908511000001101 | Beclometasone 200micrograms/dose inhaler |
| 5119 | CorticosteroidsInhaled | 35908611000001102 | Beclometasone 250micrograms/dose breath actuated inhaler |
| 5119 | CorticosteroidsInhaled | 35908711000001106 | Beclometasone 250micrograms/dose dry powder inhaler |
| 5119 | CorticosteroidsInhaled | 35908811000001103 | Beclometasone 250micrograms/dose inhaler |
| 5119 | CorticosteroidsInhaled | 35908911000001108 | Beclometasone 400microgram inhalation powder blisters |
| 5119 | CorticosteroidsInhaled | 35909011000001104 | Beclometasone 400microgram inhalation powder blisters with device |
| 5119 | CorticosteroidsInhaled | 35909111000001103 | Beclometasone 400micrograms/dose dry powder inhaler |
| 5119 | CorticosteroidsInhaled | 35909211000001109 | Beclometasone 50micrograms/dose breath actuated inhaler |
| 5119 | CorticosteroidsInhaled | 35909311000001101 | Beclometasone 50micrograms/dose breath actuated inhaler CFC free |
| 5119 | CorticosteroidsInhaled | 35909411000001108 | Beclometasone 50micrograms/dose dry powder inhaler |
| 5119 | CorticosteroidsInhaled | 35909511000001107 | Beclometasone 50micrograms/dose inhaler |
| 5119 | CorticosteroidsInhaled | 35909611000001106 | Beclometasone 50micrograms/dose inhaler CFC free |
| 5119 | CorticosteroidsInhaled | 35912011000001109 | Budesonide 100micrograms/dose / Formoterol 6micrograms/dose dry powder inhaler |
| 5119 | CorticosteroidsInhaled | 35912111000001105 | Budesonide 100micrograms/dose dry powder inhaler |
| 5119 | CorticosteroidsInhaled | 35912411000001100 | Budesonide 200micrograms/dose dry powder inhaler |
| 5119 | CorticosteroidsInhaled | 35912511000001101 | Budesonide 200micrograms/dose inhaler |
| 5119 | CorticosteroidsInhaled | 35912711000001106 | Budesonide 400micrograms/dose dry powder inhaler |
| 5119 | CorticosteroidsInhaled | 35912811000001103 | Budesonide 50micrograms/dose inhaler |
| 5119 | CorticosteroidsInhaled | 35936211000001105 | Salbutamol 100micrograms/dose / Beclometasone 50micrograms/dose inhaler |
| 5119 | CorticosteroidsInhaled | 36059111000001102 | Fluticasone 25micrograms/dose inhaler |
| 5119 | CorticosteroidsInhaled | 36059211000001108 | Fluticasone propionate 100microgram inhalation powder blisters |

| Con ditio nID | Condition name | DMD code | Description |
|---|---|---|---|
| 5119 | CorticosteroidsInhaled | 36059311000001100 | Fluticasone propionate 100microgram inhalation powder blisters with device |
| 5119 | CorticosteroidsInhaled | 36059411000001107 | Fluticasone propionate 250microgram inhalation powder blisters with device |
| 5119 | CorticosteroidsInhaled | 36059511000001106 | Fluticasone propionate 250microgram inhalation powder blisters |
| 5119 | CorticosteroidsInhaled | 36059611000001105 | Fluticasone propionate 500microgram inhalation powder blisters |
| 5119 | CorticosteroidsInhaled | 36059711000001101 | Fluticasone propionate 500microgram inhalation powder blisters with device |
| 5119 | CorticosteroidsInhaled | 36059811000001109 | Fluticasone propionate 50microgram inhalation powder blisters |
| 5119 | CorticosteroidsInhaled | 36059911000001104 | Fluticasone propionate 50microgram inhalation powder blisters with device |
| 5119 | CorticosteroidsInhaled | 36565411000001101 | Fluticasone propionate 250micrograms/dose dry powder inhaler |
| 5119 | CorticosteroidsInhaled | 36603211000001106 | Soprobec 50micrograms/dose inhaler (Glenmark Pharmaceuticals Europe Ltd) |
| 5119 | CorticosteroidsInhaled | 36603411000001105 | Soprobec 200micrograms/dose inhaler (Glenmark Pharmaceuticals Europe Ltd) |
| 5119 | CorticosteroidsInhaled | 36603611000001108 | Soprobec 100micrograms/dose inhaler (Glenmark Pharmaceuticals Europe Ltd) |
| 5119 | CorticosteroidsInhaled | 36603811000001107 | Soprobec 250micrograms/dose inhaler (Glenmark Pharmaceuticals Europe Ltd) |
| 5119 | CorticosteroidsInhaled | 36604711000001102 | Stalpex 50micrograms/dose / 500micrograms/dose dry powder inhaler (Glenmark Pharmaceuticals Europe Ltd) |
| 5119 | CorticosteroidsInhaled | 36889311000001107 | DuoResp Spiromax 160micrograms/dose / 4.5micrograms/dose dry powder inhaler (Pharmaram Ltd) |
| 5119 | CorticosteroidsInhaled | 36889511000001101 | DuoResp Spiromax 320micrograms/dose / 9micrograms/dose dry powder inhaler (Pharmaram Ltd) |
| 5119 | CorticosteroidsInhaled | 37391711000001105 | Flutiform 125micrograms/dose / 5micrograms/dose inhaler (CST Pharma Ltd) |
| 5119 | CorticosteroidsInhaled | 37397211000001108 | DuoResp Spiromax 320micrograms/dose / 9micrograms/dose dry powder inhaler (Mawdsley-Brooks & Company Ltd) |
| 5119 | CorticosteroidsInhaled | 37397411000001107 | DuoResp Spiromax 160micrograms/dose / 4.5micrograms/dose dry powder inhaler (Mawdsley-Brooks & Company Ltd) |
| 5119 | CorticosteroidsInhaled | 37400911000001108 | Qvar 100 Autohaler (CST Pharma Ltd) |
| 5119 | CorticosteroidsInhaled | 37434411000001103 | DuoResp Spiromax 160micrograms/dose / 4.5micrograms/dose dry powder inhaler (CST Pharma Ltd) |
| 5119 | CorticosteroidsInhaled | 37434611000001100 | DuoResp Spiromax 320micrograms/dose / 9micrograms/dose dry powder inhaler (CST Pharma Ltd) |
| 5119 | CorticosteroidsInhaled | 37443211000001101 | Seretide 250 Accuhaler (CST Pharma Ltd) |
| 5119 | CorticosteroidsInhaled | 37443411000001102 | Seretide 500 Accuhaler (CST Pharma Ltd) |
| 5119 | CorticosteroidsInhaled | 37443611000001104 | Seretide 250 Evohaler (CST Pharma Ltd) |
| 5119 | CorticosteroidsInhaled | 37444011000001108 | Seretide 125 Evohaler (CST Pharma Ltd) |
| 5119 | CorticosteroidsInhaled | 37444211000001103 | Symbicort 400/12 Turbohaler (CST Pharma Ltd) |
| 5119 | CorticosteroidsInhaled | 37444611000001101 | Qvar 100 inhaler (CST Pharma Ltd) |
| 5119 | CorticosteroidsInhaled | 37446211000001108 | Flixotide 100micrograms/dose Accuhaler (CST Pharma Ltd) |
| 5119 | CorticosteroidsInhaled | 37446411000001107 | Flixotide 250micrograms/dose Accuhaler (CST Pharma Ltd) |
| 5119 | CorticosteroidsInhaled | 37446611000001105 | Flixotide 500micrograms/dose Accuhaler (CST Pharma Ltd) |
| 5119 | CorticosteroidsInhaled | 37448711000001102 | Flutiform 250micrograms/dose / 10micrograms/dose inhaler (CST Pharma Ltd) |
| 5119 | CorticosteroidsInhaled | 37484711000001101 | Flutiform 250micrograms/dose / 10micrograms/dose inhaler (Mawdsley-Brooks & Company Ltd) |
| 5119 | CorticosteroidsInhaled | 37484911000001104 | Flutiform 125micrograms/dose / 5micrograms/dose inhaler (Mawdsley-Brooks & Company Ltd) |
| 5119 | CorticosteroidsInhaled | 37577111000001108 | Alvesco 160 inhaler (CST Pharma Ltd) |
| 5119 | CorticosteroidsInhaled | 37631511000001104 | DuoResp Spiromax 160micrograms/dose / 4.5micrograms/dose dry powder inhaler (Ethigen Ltd) |
| 5119 | CorticosteroidsInhaled | 37631711000001109 | DuoResp Spiromax 320micrograms/dose / 9micrograms/dose dry powder inhaler (Ethigen Ltd) |
| 5119 | CorticosteroidsInhaled | 37665111000001104 | Flutiform 125micrograms/dose / 5micrograms/dose inhaler (Pilsco Ltd) |
| 5119 | CorticosteroidsInhaled | 37665311000001102 | Flutiform 250micrograms/dose / 10micrograms/dose inhaler (Pilsco Ltd) |

| ConditionID | Condition name | DMD code | Description |
|---|---|---|---|
| 5119 | CorticosteroidsInhaled | 3769911000001100 | Pulmicort 0.5mg Respules (Pilsco Ltd) |
| 5119 | CorticosteroidsInhaled | 3769931000001103 | Pulmicort 1mg Respules (Pilsco Ltd) |
| 5119 | CorticosteroidsInhaled | 3769971000001104 | Qvar 100 Autohaler (Pilsco Ltd) |
| 5119 | CorticosteroidsInhaled | 3770221000001106 | Seretide 250 Accuhaler (Pilsco Ltd) |
| 5119 | CorticosteroidsInhaled | 3770241000001105 | Seretide 250 Evohaler (Pilsco Ltd) |
| 5119 | CorticosteroidsInhaled | 3770261000001108 | Seretide 500 Accuhaler (Pilsco Ltd) |
| 5119 | CorticosteroidsInhaled | 3771441000001100 | AirFluSal Forspiro 50micrograms/dose / 500micrograms/dose dry powder inhaler (Pilsco Ltd) |
| 5119 | CorticosteroidsInhaled | 3771461000001102 | AirFluSal 25micrograms/dose / 125micrograms/dose inhaler (Pilsco Ltd) |
| 5119 | CorticosteroidsInhaled | 3771481000001103 | AirFluSal 25micrograms/dose / 250micrograms/dose inhaler (Pilsco Ltd) |
| 5119 | CorticosteroidsInhaled | 3772781000001101 | Qvar 100 inhaler (Pilsco Ltd) |
| 5119 | CorticosteroidsInhaled | 3772941000001107 | Sereflo 25micrograms/dose / 250micrograms/dose inhaler (Pilsco Ltd) |
| 5119 | CorticosteroidsInhaled | 3773901000001101 | Symbicort 200/6 Turbohaler (CST Pharma Ltd) |
| 5119 | CorticosteroidsInhaled | 3799651000001103 | Seretide 125 Evohaler (Pharmaram Ltd) |
| 5119 | CorticosteroidsInhaled | 3799671000001108 | Seretide 250 Evohaler (Pharmaram Ltd) |
| 5119 | CorticosteroidsInhaled | 3799731000001107 | Symbicort 100/6 Turbohaler (Pharmaram Ltd) |
| 5119 | CorticosteroidsInhaled | 3799751000001101 | Symbicort 200/6 Turbohaler (Pharmaram Ltd) |
| 5119 | CorticosteroidsInhaled | 3799771000001106 | Symbicort 400/12 Turbohaler (Pharmaram Ltd) |
| 5119 | CorticosteroidsInhaled | 3813001000001108 | DuoResp Spiromax 160micrograms/dose / 4.5micrograms/dose dry powder inhaler (DE Pharmaceuticals) |
| 5119 | CorticosteroidsInhaled | 3813021000001103 | DuoResp Spiromax 320micrograms/dose / 9micrograms/dose dry powder inhaler (DE Pharmaceuticals) |
| 5119 | CorticosteroidsInhaled | 3813041000001104 | Easyhaler Beclometasone 200micrograms/dose dry powder inhaler (DE Pharmaceuticals) |
| 5119 | CorticosteroidsInhaled | 3813061000001101 | Easyhaler Budesonide 100micrograms/dose dry powder inhaler (DE Pharmaceuticals) |
| 5119 | CorticosteroidsInhaled | 3813081000001102 | Easyhaler Budesonide 200micrograms/dose dry powder inhaler (DE Pharmaceuticals) |
| 5119 | CorticosteroidsInhaled | 3813101000001104 | Easyhaler Budesonide 400micrograms/dose dry powder inhaler (DE Pharmaceuticals) |
| 5119 | CorticosteroidsInhaled | 3813451000001109 | Flutiform 125micrograms/dose / 5micrograms/dose inhaler (DE Pharmaceuticals) |
| 5119 | CorticosteroidsInhaled | 3813471000001104 | Flutiform 250micrograms/dose / 10micrograms/dose inhaler (DE Pharmaceuticals) |
| 5119 | CorticosteroidsInhaled | 3813491000001102 | Flutiform 50micrograms/dose / 5micrograms/dose inhaler (DE Pharmaceuticals) |
| 5119 | CorticosteroidsInhaled | 3813511000001101 | Flutiform K-haler 125micrograms/dose / 5micrograms/dose breath actuated inhaler (DE Pharmaceuticals) |
| 5119 | CorticosteroidsInhaled | 3813531000001104 | Flutiform K-haler 50micrograms/dose / 5micrograms/dose breath actuated inhaler (DE Pharmaceuticals) |
| 5119 | CorticosteroidsInhaled | 3813601000001106 | Fostair NEXThaler 100micrograms/dose / 6micrograms/dose dry powder inhaler (DE Pharmaceuticals) |
| 5119 | CorticosteroidsInhaled | 3816691000001103 | Sirdupla 25micrograms/dose / 125micrograms/dose inhaler (DE Pharmaceuticals) |
| 5119 | CorticosteroidsInhaled | 3816711000001103 | Sirdupla 25micrograms/dose / 250micrograms/dose inhaler (DE Pharmaceuticals) |
| 5119 | CorticosteroidsInhaled | 3864011000001109 | Sirdupla 25micrograms/dose / 125micrograms/dose inhaler (Pilsco Ltd) |
| 5119 | CorticosteroidsInhaled | 3864031000001106 | Sirdupla 25micrograms/dose / 250micrograms/dose inhaler (Pilsco Ltd) |
| 5119 | CorticosteroidsInhaled | 3880721000001100 | Budesonide 500micrograms/2ml nebuliser liquid unit dose vials (Medihealth (Northern) Ltd) |
| 5119 | CorticosteroidsInhaled | 3880741000001101 | Budesonide 1mg/2ml nebuliser liquid unit dose vials (Medihealth (Northern) Ltd) |
| 5119 | CorticosteroidsInhaled | 3889541000001109 | Mometasone 200micrograms/dose dry powder inhaler |
| 5119 | CorticosteroidsInhaled | 3889561000001107 | Mometasone 400micrograms/dose dry powder inhaler |
| 5119 | CorticosteroidsInhaled | 3889681000001103 | Budesonide 200micrograms/dose / Formoterol 6micrograms/dose dry powder inhaler |
| 5119 | CorticosteroidsInhaled | 3889741000001103 | Fluticasone propionate 500micrograms/dose / Salmeterol 50micrograms/dose dry powder inhaler |

| Con ditio nID | Condition name | DMD code | Description |
|---|---|---|---|
| 5119 | CorticosteroidsInhaled | 38897511000001104 | Fluticasone propionate 250micrograms/dose / Salmeterol 50micrograms/dose dry powder inhaler |
| 5119 | CorticosteroidsInhaled | 38897611000001100 | Fluticasone propionate 100micrograms/dose / Salmeterol 50micrograms/dose dry powder inhaler |
| 5119 | CorticosteroidsInhaled | 38897711000001109 | Fluticasone propionate 500micrograms/dose dry powder inhaler |
| 5119 | CorticosteroidsInhaled | 38897811000001101 | Fluticasone propionate 100micrograms/dose dry powder inhaler |
| 5119 | CorticosteroidsInhaled | 38897911000001106 | Fluticasone propionate 50micrograms/dose dry powder inhaler |
| 5119 | CorticosteroidsInhaled | 38960111000001101 | Fluticasone 500micrograms/2ml nebuliser liquid unit dose vials (Imported) |
| 5119 | CorticosteroidsInhaled | 38960511000001105 | Fluticasone 2mg/2ml nebuliser liquid unit dose vials (Imported) |
| 5119 | CorticosteroidsInhaled | 39089311000001103 | Flutiform K-haler 125micrograms/dose / 5micrograms/dose breath actuated inhaler (Pilsco Ltd) |
| 5119 | CorticosteroidsInhaled | 39105811000001102 | Symbicort 100micrograms/dose / 3micrograms/dose pressurised inhaler (AstraZeneca UK Ltd) |
| 5119 | CorticosteroidsInhaled | 39110911000001100 | Fluticasone 50micrograms/dose inhaler CFC free |
| 5119 | CorticosteroidsInhaled | 39111011000001108 | Fluticasone 50micrograms/dose / Salmeterol 25micrograms/dose inhaler CFC free |
| 5119 | CorticosteroidsInhaled | 39111111000001109 | Fluticasone 125micrograms/dose / Salmeterol 25micrograms/dose inhaler CFC free |
| 5119 | CorticosteroidsInhaled | 39111211000001103 | Fluticasone 125micrograms/dose inhaler CFC free |
| 5119 | CorticosteroidsInhaled | 39111311000001106 | Fluticasone 250micrograms/dose / Salmeterol 25micrograms/dose inhaler CFC free |
| 5119 | CorticosteroidsInhaled | 39111411000001104 | Fluticasone 250micrograms/dose inhaler CFC free |
| 5119 | CorticosteroidsInhaled | 39114511000001103 | Atectura Breezhaler 125micrograms/62.5micrograms inhalation powder capsules with device (Sandoz Ltd) |
| 5119 | CorticosteroidsInhaled | 39115411000001101 | Atectura Breezhaler 125micrograms/127.5micrograms inhalation powder capsules with device (Sandoz Ltd) |
| 5119 | CorticosteroidsInhaled | 39115911000001109 | Atectura Breezhaler 125micrograms/260micrograms inhalation powder capsules with device (Sandoz Ltd) |
| 5119 | CorticosteroidsInhaled | 39116311000001103 | Indacaterol 125micrograms/dose / Mometasone 127.5micrograms/dose inhalation powder capsules with device |
| 5119 | CorticosteroidsInhaled | 39116411000001105 | Indacaterol 125micrograms/dose / Mometasone 260micrograms/dose inhalation powder capsules with device |
| 5119 | CorticosteroidsInhaled | 39116511000001109 | Indacaterol 125micrograms/dose / Mometasone 62.5micrograms/dose inhalation powder capsules with device |
| 5119 | CorticosteroidsInhaled | 39133611000001108 | Budesonide 100micrograms/dose / Formoterol 3micrograms/dose inhaler CFC free |
| 5119 | CorticosteroidsInhaled | 39134511000001107 | Generic Enerzair Breezhaler 114micrograms/dose / 46micrograms/dose / 136micrograms/dose inhalation powder capsules with device |
| 5119 | CorticosteroidsInhaled | 39134711000001102 | Enerzair Breezhaler 114micrograms/dose / 46micrograms/dose / 136micrograms/dose inhalation powder capsules with device (Sandoz Ltd) |
| 5119 | CorticosteroidsInhaled | 39327311000001104 | Trixeo Aerosphere 5micrograms/dose / 7.2micrograms/dose / 160micrograms/dose pressurised inhaler (AstraZeneca UK Ltd) |
| 5119 | CorticosteroidsInhaled | 39343511000001103 | Generic Trixeo Aerosphere 5micrograms/dose / 7.2micrograms/dose / 160micrograms/dose inhaler CFC free |
| 5119 | CorticosteroidsInhaled | 39360211000001103 | Fostair NEXThaler 100micrograms/dose / 6micrograms/dose dry powder inhaler (Pilsco Ltd) |
| 5119 | CorticosteroidsInhaled | 39360411000001104 | Sereflo 25micrograms/dose / 125micrograms/dose inhaler (Pilsco Ltd) |
| 5119 | CorticosteroidsInhaled | 39567411000001102 | Fixkoh Airmaster 50micrograms/dose / 500micrograms/dose dry powder inhaler (Thornton & Ross Ltd) |
| 5119 | CorticosteroidsInhaled | 39567611000001104 | Fixkoh Airmaster 50micrograms/dose / 250micrograms/dose dry powder inhaler (Thornton & Ross Ltd) |
| 5119 | CorticosteroidsInhaled | 39567811000001100 | Fixkoh Airmaster 50micrograms/dose / 100micrograms/dose dry powder inhaler (Thornton & Ross Ltd) |
| 5119 | CorticosteroidsInhaled | 39691211000001100 | Fluticasone propionate 100micrograms/dose dry powder inhaler (Imported) |
| 5119 | CorticosteroidsInhaled | 39695511000001104 | Beclometasone 100microgram inhalation powder capsules |
| 5119 | CorticosteroidsInhaled | 39695611000001100 | Beclometasone 200microgram inhalation powder capsules |
| 5119 | CorticosteroidsInhaled | 39695711000001109 | Beclometasone 400microgram inhalation powder capsules |
| 5119 | CorticosteroidsInhaled | 39696311000001100 | Salbutamol 400microgram / Beclometasone 200microgram inhalation powder capsules |
| 5119 | CorticosteroidsInhaled | 39712311000001105 | Fluticasone 500micrograms/2ml nebuliser liquid unit dose vials |
| 5119 | CorticosteroidsInhaled | 39712511000001104 | Fluticasone 2mg/2ml nebuliser liquid unit dose vials |

| Con ditio nID | Condition name | DMD code | Description |
|---|---|---|---|
| 5119 | CorticosteroidsInhaled | 39817511000001103 | Luforbec 100micrograms/dose / 6micrograms/dose inhaler (Lupin Healthcare (UK) Ltd) |
| 5119 | CorticosteroidsInhaled | 39939611000001107 | Fostair 100micrograms/dose / 6micrograms/dose inhaler (CST Pharma Ltd) |
| 5119 | CorticosteroidsInhaled | 39993311000001105 | Trimbow NEXThaler 88micrograms/dose / 5micrograms/dose / 9micrograms/dose dry powder inhaler (Chiesi Ltd) |
| 5119 | CorticosteroidsInhaled | 40034211000001104 | Avenor 25micrograms/dose / 50micrograms/dose inhaler (Zentiva Pharma UK Ltd) |
| 5119 | CorticosteroidsInhaled | 40040711000001101 | Avenor 25micrograms/dose / 125micrograms/dose inhaler (Zentiva Pharma UK Ltd) |
| 5119 | CorticosteroidsInhaled | 40040911000001104 | Avenor 25micrograms/dose / 250micrograms/dose inhaler (Zentiva Pharma UK Ltd) |
| 5119 | CorticosteroidsInhaled | 40087411000001104 | Generic Trimbow NEXThaler 88micrograms/dose / 5micrograms/dose / 9micrograms/dose dry powder inhaler |
| 5119 | CorticosteroidsInhaled | 40106011000001102 | WockAIR 160micrograms/dose / 4.5micrograms/dose dry powder inhaler (Wockhardt UK Ltd) |
| 5119 | CorticosteroidsInhaled | 40106211000001107 | WockAIR 320micrograms/dose / 9micrograms/dose dry powder inhaler (Wockhardt UK Ltd) |
| 5119 | CorticosteroidsInhaled | 40220711000001106 | Seretide 50 Evohaler (CST Pharma Ltd) |
| 5119 | CorticosteroidsInhaled | 40220911000001108 | Fostair NEXThaler 100micrograms/dose / 6micrograms/dose dry powder inhaler (CST Pharma Ltd) |
| 5119 | CorticosteroidsInhaled | 40377811000001105 | Alvesco 160 inhaler (DE Pharmaceuticals) |
| 5119 | CorticosteroidsInhaled | 40444911000001101 | Seffalair Spiromax 12.75micrograms/dose / 202micrograms/dose dry powder inhaler (Teva UK Ltd) |
| 5119 | CorticosteroidsInhaled | 40445111000001100 | Seffalair Spiromax 12.75micrograms/dose / 100micrograms/dose dry powder inhaler (Teva UK Ltd) |
| 5119 | CorticosteroidsInhaled | 40455711000001101 | Fluticasone propionate 100micrograms/dose / Salmeterol 12.75micrograms/dose dry powder inhaler |
| 5119 | CorticosteroidsInhaled | 40455811000001109 | Fluticasone propionate 202micrograms/dose / Salmeterol 12.75micrograms/dose dry powder inhaler |
| 5119 | CorticosteroidsInhaled | 40504911000001103 | Sereflo Ciphaler 50micrograms/dose / 250micrograms/dose dry powder inhaler (Cipla EU Ltd) |
| 5119 | CorticosteroidsInhaled | 40752211000001109 | Trimbow 172micrograms/dose / 5micrograms/dose / 9micrograms/dose inhaler (Chiesi Ltd) |
| 5119 | CorticosteroidsInhaled | 40766811000001104 | Generic Trimbow 172micrograms/dose / 5micrograms/dose / 9micrograms/dose inhaler |
| 5119 | CorticosteroidsInhaled | 40852311000001103 | Luforbec 200micrograms/dose / 6micrograms/dose inhaler (Lupin Healthcare (UK) Ltd) |
| 5267 | Prednisolone | 325426006 | Prednisolone 1mg tablets |
| 5267 | Prednisolone | 325427002 | Prednisolone 5mg tablets |
| 5267 | Prednisolone | 325442004 | Prednisolone 2.5mg gastro-resistant tablets |
| 5267 | Prednisolone | 325443009 | Prednisolone 5mg gastro-resistant tablets |
| 5267 | Prednisolone | 325444003 | Prednisolone 5mg soluble tablets |
| 5267 | Prednisolone | 325445002 | Prednisolone 50mg tablets |
| 5267 | Prednisolone | 325450008 | Prednisolone 25mg tablets |
| 5267 | Prednisolone | 416533002 | Prednisolone 15mg/5ml oral solution |
| 5267 | Prednisolone | 429995001 | Prednisolone 10mg/5ml oral solution |
| 5267 | Prednisolone | 432224007 | Prednisolone 5mg/5ml oral suspension |
| 5267 | Prednisolone | 432225008 | Prednisolone 15mg/5ml oral suspension |
| 5267 | Prednisolone | 52911000001107 | Prednisolone 1mg tablets (Alliance Healthcare (Distribution) Ltd) |
| 5267 | Prednisolone | 85511000001101 | Prednisolone 5mg tablets (Accord Healthcare Ltd) |
| 5267 | Prednisolone | 86711000001103 | Prednisolone 5mg tablets (The Boots Company Plc) |
| 5267 | Prednisolone | 110811000001100 | Prednisolone 5mg tablets (Teva UK Ltd) |
| 5267 | Prednisolone | 113391000001105 | Prednisolone 5mg soluble tablets |
| 5267 | Prednisolone | 157711000001100 | Prednisolone 5mg soluble tablets (Alliance Healthcare (Distribution) Ltd) |
| 5267 | Prednisolone | 238511000001107 | Prednisolone 1mg tablets (Teva UK Ltd) |
| 5267 | Prednisolone | 245011000001108 | Prednisolone 5mg soluble tablets (A A H Pharmaceuticals Ltd) |

| Con ditio nID | Condition name | DMD code | Description |
|---|---|---|---|
| 5267 | Prednisolone | 255211000001101 | Prednisolone 5mg gastro-resistant tablets (Alliance Healthcare (Distribution) Ltd) |
| 5267 | Prednisolone | 313911000001107 | Prednisolone 25mg tablets (Alliance Healthcare (Distribution) Ltd) |
| 5267 | Prednisolone | 331111000001100 | Deltacortril 5mg gastro-resistant tablets (Phoenix Labs Ltd) |
| 5267 | Prednisolone | 350611000001104 | Prednisolone 5mg tablets (Kent Pharma (UK) Ltd) |
| 5267 | Prednisolone | 380011000001100 | Prednisolone 2.5mg gastro-resistant tablets (Accord Healthcare Ltd) |
| 5267 | Prednisolone | 392511000001108 | Prednisolone 5mg soluble tablets (Advanz Pharma) |
| 5267 | Prednisolone | 447211000001105 | Prednisolone 5mg gastro-resistant tablets (Kent Pharma (UK) Ltd) |
| 5267 | Prednisolone | 459611000001100 | Deltacortril 2.5mg gastro-resistant tablets (Phoenix Labs Ltd) |
| 5267 | Prednisolone | 482891000001105 | Prednisolone 10mg/5ml oral solution |
| 5267 | Prednisolone | 512811000001108 | Prednisolone 5mg gastro-resistant tablets (Accord Healthcare Ltd) |
| 5267 | Prednisolone | 646211000001106 | Prednisolone 1mg tablets (Accord Healthcare Ltd) |
| 5267 | Prednisolone | 649711000001109 | Prednisolone 1mg tablets (A A H Pharmaceuticals Ltd) |
| 5267 | Prednisolone | 662011000001105 | Prednisolone 5mg gastro-resistant tablets (A A H Pharmaceuticals Ltd) |
| 5267 | Prednisolone | 685311000001104 | Prednisolone 2.5mg gastro-resistant tablets (Kent Pharma (UK) Ltd) |
| 5267 | Prednisolone | 707911000001100 | Prednisolone 1mg tablets (Kent Pharma (UK) Ltd) |
| 5267 | Prednisolone | 716111000001103 | Prednisolone 25mg tablets (A A H Pharmaceuticals Ltd) |
| 5267 | Prednisolone | 772111000001107 | Prednisolone 5mg tablets (Alliance Healthcare (Distribution) Ltd) |
| 5267 | Prednisolone | 779211000001109 | Prednisolone 2.5mg gastro-resistant tablets (Alliance Healthcare (Distribution) Ltd) |
| 5267 | Prednisolone | 798611000001108 | Prednisolone 1mg tablets (The Boots Company Plc) |
| 5267 | Prednisolone | 844111000001103 | Prednisolone 1mg tablets (Wockhardt UK Ltd) |
| 5267 | Prednisolone | 858811000001104 | Prednisolone 2.5mg gastro-resistant tablets (A A H Pharmaceuticals Ltd) |
| 5267 | Prednisolone | 876911000001107 | Prednisolone 5mg tablets (A A H Pharmaceuticals Ltd) |
| 5267 | Prednisolone | 940311000001100 | Prednisolone 5mg tablets (Wockhardt UK Ltd) |
| 5267 | Prednisolone | 4208511000001105 | Prednisolone 2.5mg gastro-resistant tablets (Approved Prescription Services Ltd) |
| 5267 | Prednisolone | 4208911000001103 | Prednisolone 5mg gastro-resistant tablets (Approved Prescription Services Ltd) |
| 5267 | Prednisolone | 4752611000001107 | Prednisolone 50mg tablets (A A H Pharmaceuticals Ltd) |
| 5267 | Prednisolone | 4752811000001106 | Prednisolone 50mg tablets (Approved Prescription Services Ltd) |
| 5267 | Prednisolone | 4852811000001108 | Prednisolone 25mg tablets (Zentiva Pharma UK Ltd) |
| 5267 | Prednisolone | 8651111000001103 | Prednisolone 2mg/5ml oral suspension (Special Order) |
| 5267 | Prednisolone | 8672011000001109 | Prednisolone 2mg/5ml oral suspension |
| 5267 | Prednisolone | 9807711000001108 | Prednisolone 1mg tablets (Almus Pharmaceuticals Ltd) |
| 5267 | Prednisolone | 9807911000001105 | Prednisolone 5mg tablets (Almus Pharmaceuticals Ltd) |
| 5267 | Prednisolone | 10409611000001103 | Prednisolone 1mg tablets (Arrow Generics Ltd) |
| 5267 | Prednisolone | 10410111000001103 | Prednisolone 5mg tablets (Arrow Generics Ltd) |
| 5267 | Prednisolone | 13053211000001103 | Prednisolone 1.5mg/5ml oral suspension (Special Order) |
| 5267 | Prednisolone | 13053711000001105 | Prednisolone 1.5mg/5ml oral solution (Special Order) |
| 5267 | Prednisolone | 13054411000001101 | Prednisolone 10mg/5ml oral suspension (Special Order) |
| 5267 | Prednisolone | 13054711000001107 | Prednisolone 10mg/5ml oral solution (Special Order) |
| 5267 | Prednisolone | 13055311000001107 | Prednisolone 15mg/5ml oral suspension (Special Order) |
| 5267 | Prednisolone | 13055911000001108 | Prednisolone 15mg/5ml oral solution (Special Order) |
| 5267 | Prednisolone | 13056211000001105 | Prednisolone 1mg/5ml oral suspension (Special Order) |
| 5267 | Prednisolone | 13056511000001108 | Prednisolone 1mg/5ml oral solution (Special Order) |
| 5267 | Prednisolone | 13056811000001106 | Prednisolone 2.5mg/5ml oral suspension (Special Order) |

| ConditionID | Condition name | DMD code | Description |
|---|---|---|---|
| 5267 | Prednisolone | 13057411000001106 | Prednisolone 2.5mg/5ml oral solution (Special Order) |
| 5267 | Prednisolone | 13058011000001101 | Prednisolone 20mg/5ml oral suspension (Special Order) |
| 5267 | Prednisolone | 13058311000001103 | Prednisolone 20mg/5ml oral solution (Special Order) |
| 5267 | Prednisolone | 13058611000001108 | Prednisolone 25mg/5ml oral suspension (Special Order) |
| 5267 | Prednisolone | 13058911000001102 | Prednisolone 25mg/5ml oral solution (Special Order) |
| 5267 | Prednisolone | 13078311000001101 | Prednisolone 1.5mg/5ml oral solution |
| 5267 | Prednisolone | 13078411000001108 | Prednisolone 1.5mg/5ml oral suspension |
| 5267 | Prednisolone | 13078611000001106 | Prednisolone 10mg/5ml oral suspension |
| 5267 | Prednisolone | 13078911000001100 | Prednisolone 1mg/5ml oral solution |
| 5267 | Prednisolone | 13079011000001109 | Prednisolone 1mg/5ml oral suspension |
| 5267 | Prednisolone | 13079111000001105 | Prednisolone 2.5mg/5ml oral solution |
| 5267 | Prednisolone | 13079211000001104 | Prednisolone 2.5mg/5ml oral suspension |
| 5267 | Prednisolone | 13079311000001107 | Prednisolone 20mg/5ml oral solution |
| 5267 | Prednisolone | 13079411000001100 | Prednisolone 20mg/5ml oral suspension |
| 5267 | Prednisolone | 13079511000001101 | Prednisolone 25mg/5ml oral solution |
| 5267 | Prednisolone | 13079611000001102 | Prednisolone 25mg/5ml oral suspension |
| 5267 | Prednisolone | 13120111000001109 | Prednisolone 5mg/5ml oral suspension (Special Order) |
| 5267 | Prednisolone | 13120411000001104 | Prednisolone 5mg/5ml oral solution (Special Order) |
| 5267 | Prednisolone | 13133111000001100 | Prednisolone 5mg/5ml oral solution |
| 5267 | Prednisolone | 13245311000001104 | Prednisolone 1mg tablets (Dowelhurst Ltd) |
| 5267 | Prednisolone | 13245511000001105 | Prednisolone 5mg tablets (Dowelhurst Ltd) |
| 5267 | Prednisolone | 13245711000001100 | Prednisolone 2.5mg gastro-resistant tablets (Dowelhurst Ltd) |
| 5267 | Prednisolone | 13245911000001103 | Prednisolone 5mg gastro-resistant tablets (Dowelhurst Ltd) |
| 5267 | Prednisolone | 14173111000001101 | Prednisolone 1.67mg/5ml oral solution (Special Order) |
| 5267 | Prednisolone | 14173511000001105 | Prednisolone 1.67mg/5ml oral suspension (Special Order) |
| 5267 | Prednisolone | 14204411000001101 | Prednisolone 1.67mg/5ml oral solution |
| 5267 | Prednisolone | 14204511000001102 | Prednisolone 1.67mg/5ml oral suspension |
| 5267 | Prednisolone | 14786411000001103 | Prednisolone 1mg tablets (Boston Healthcare Ltd) |
| 5267 | Prednisolone | 14786811000001101 | Prednisolone 5mg tablets (Boston Healthcare Ltd) |
| 5267 | Prednisolone | 15172311000001102 | Prednisolone 5mg soluble tablets (Sigma Pharmaceuticals Plc) |
| 5267 | Prednisolone | 15175511000001102 | Prednisolone 1mg tablets (Sigma Pharmaceuticals Plc) |
| 5267 | Prednisolone | 15175711000001107 | Prednisolone 2.5mg gastro-resistant tablets (Sigma Pharmaceuticals Plc) |
| 5267 | Prednisolone | 15176111000001100 | Prednisolone 5mg gastro-resistant tablets (Sigma Pharmaceuticals Plc) |
| 5267 | Prednisolone | 17916411000001100 | Prednisolone 2.5mg gastro-resistant tablets (Phoenix Healthcare Distribution Ltd) |
| 5267 | Prednisolone | 17916811000001103 | Prednisolone 5mg gastro-resistant tablets (Phoenix Healthcare Distribution Ltd) |
| 5267 | Prednisolone | 17917211000001102 | Prednisolone 25mg tablets (Phoenix Healthcare Distribution Ltd) |
| 5267 | Prednisolone | 17999311000001104 | Prednisolone 2.5mg gastro-resistant tablets (Phoenix Labs Ltd) |
| 5267 | Prednisolone | 17999611000001109 | Prednisolone 5mg gastro-resistant tablets (Phoenix Labs Ltd) |
| 5267 | Prednisolone | 18285011000001109 | Prednisolone 1mg tablets (Strides Pharma UK Ltd) |
| 5267 | Prednisolone | 18285211000001104 | Prednisolone 5mg tablets (Strides Pharma UK Ltd) |
| 5267 | Prednisolone | 18307011000001109 | Prednisolone 2.5mg gastro-resistant tablets (Teva UK Ltd) |
| 5267 | Prednisolone | 18307211000001104 | Prednisolone 5mg gastro-resistant tablets (Teva UK Ltd) |
| 5267 | Prednisolone | 18625211000001107 | Prednisolone 5mg tablets (Phoenix Healthcare Distribution Ltd) |

| Con ditio nID | Condition name | DMD code | Description |
|---|---|---|---|
| 5267 | Prednisolone | 19743011000001109 | Prednisolone 2.5mg gastro-resistant tablets (Almus Pharmaceuticals Ltd) |
| 5267 | Prednisolone | 19743211000001104 | Prednisolone 5mg gastro-resistant tablets (Almus Pharmaceuticals Ltd) |
| 5267 | Prednisolone | 21851211000001107 | Prednisolone 1mg tablets (Waymade Healthcare Plc) |
| 5267 | Prednisolone | 21851411000001106 | Prednisolone 5mg tablets (Waymade Healthcare Plc) |
| 5267 | Prednisolone | 22452711000001102 | Prednisolone 2.5mg gastro-resistant tablets (Waymade Healthcare Plc) |
| 5267 | Prednisolone | 22453011000001108 | Prednisolone 5mg gastro-resistant tablets (Waymade Healthcare Plc) |
| 5267 | Prednisolone | 22519711000001105 | Dilacort 2.5mg gastro-resistant tablets (Auden McKenzie (Pharma Division) Ltd) |
| 5267 | Prednisolone | 22520311000001107 | Dilacort 5mg gastro-resistant tablets (Auden McKenzie (Pharma Division) Ltd) |
| 5267 | Prednisolone | 28798211000001102 | Pevanti 2.5mg tablets (Advanz Pharma) |
| 5267 | Prednisolone | 28799011000001102 | Pevanti 5mg tablets (Advanz Pharma) |
| 5267 | Prednisolone | 28799311000001104 | Pevanti 10mg tablets (Advanz Pharma) |
| 5267 | Prednisolone | 28799611000001109 | Pevanti 20mg tablets (Advanz Pharma) |
| 5267 | Prednisolone | 28799811000001108 | Pevanti 25mg tablets (Advanz Pharma) |
| 5267 | Prednisolone | 28808411000001103 | Prednisolone 10mg tablets |
| 5267 | Prednisolone | 28808611000001100 | Prednisolone 2.5mg tablets |
| 5267 | Prednisolone | 28808711000001109 | Prednisolone 20mg tablets |
| 5267 | Prednisolone | 28937511000001104 | Prednisolone 5mg soluble tablets (Focus Pharmaceuticals Ltd) |
| 5267 | Prednisolone | 28995411000001102 | Prednisolone 1mg/ml oral solution (Logixx Pharma Solutions Ltd) |
| 5267 | Prednisolone | 29361211000001105 | Prednisolone 5mg/5ml oral solution unit dose (Logixx Pharma Solutions Ltd) |
| 5267 | Prednisolone | 29424511000001109 | Prednisolone 5mg/5ml oral solution unit dose |
| 5267 | Prednisolone | 29559211000001100 | Prednisolone 5mg/5ml oral solution unit dose (A A H Pharmaceuticals Ltd) |
| 5267 | Prednisolone | 29879111000001105 | Prednisolone 10mg/ml oral solution sugar free (Advanz Pharma) |
| 5267 | Prednisolone | 29904211000001102 | Prednisolone 10mg/ml oral solution sugar free |
| 5267 | Prednisolone | 30114011000001105 | Prednisolone 2.5mg gastro-resistant tablets (DE Pharmaceuticals) |
| 5267 | Prednisolone | 30114311000001108 | Prednisolone 5mg gastro-resistant tablets (DE Pharmaceuticals) |
| 5267 | Prednisolone | 30114611000001103 | Prednisolone 5mg soluble tablets (DE Pharmaceuticals) |
| 5267 | Prednisolone | 30114811000001104 | Prednisolone 1mg tablets (DE Pharmaceuticals) |
| 5267 | Prednisolone | 30115011000001109 | Prednisolone 5mg tablets (DE Pharmaceuticals) |
| 5267 | Prednisolone | 30129111000001103 | Prednisolone 10mg/ml oral solution sugar free (A A H Pharmaceuticals Ltd) |
| 5267 | Prednisolone | 30858911000001100 | Prednisolone 1mg tablets (Mawdsley-Brooks & Company Ltd) |
| 5267 | Prednisolone | 30859111000001105 | Prednisolone 5mg tablets (Mawdsley-Brooks & Company Ltd) |
| 5267 | Prednisolone | 30991311000001108 | Prednisolone 25mg tablets (Accord Healthcare Ltd) |
| 5267 | Prednisolone | 31386211000001109 | Prednisolone Dompe 5mg/5ml oral solution unit dose (Logixx Pharma Solutions Ltd) |
| 5267 | Prednisolone | 32584711000001105 | Prednisolone 1mg gastro-resistant tablets (Phoenix Labs Ltd) |
| 5267 | Prednisolone | 32611811000001105 | Prednisolone 1mg gastro-resistant tablets |
| 5267 | Prednisolone | 32652311000001103 | Dilacort 2.5mg gastro-resistant tablets (Crescent Pharma Ltd) |
| 5267 | Prednisolone | 32652511000001109 | Dilacort 5mg gastro-resistant tablets (Crescent Pharma Ltd) |
| 5267 | Prednisolone | 32688311000001106 | Prednisolone 5mg/5ml oral solution unit dose (Alliance Healthcare (Distribution) Ltd) |
| 5267 | Prednisolone | 32771211000001106 | Prednisolone 10mg tablets (Accord Healthcare Ltd) |
| 5267 | Prednisolone | 32772911000001108 | Prednisolone 2.5mg tablets (Accord Healthcare Ltd) |
| 5267 | Prednisolone | 32773211000001105 | Prednisolone 20mg tablets (Accord Healthcare Ltd) |
| 5267 | Prednisolone | 32774411000001107 | Prednisolone 30mg tablets (Accord Healthcare Ltd) |

| Con ditio nID | Condition name | DMD code | Description |
|---|---|---|---|
| 5267 | Prednisolone | 32776111000001109 | Prednisolone 2.5mg tablets (A A H Pharmaceuticals Ltd) |
| 5267 | Prednisolone | 32776311000001106 | Prednisolone 10mg tablets (A A H Pharmaceuticals Ltd) |
| 5267 | Prednisolone | 32776511000001100 | Prednisolone 20mg tablets (A A H Pharmaceuticals Ltd) |
| 5267 | Prednisolone | 32776711000001105 | Prednisolone 30mg tablets (A A H Pharmaceuticals Ltd) |
| 5267 | Prednisolone | 32781411000001102 | Prednisolone 30mg tablets |
| 5267 | Prednisolone | 32807911000001101 | Prednisolone 1mg tablets (Genesis Pharmaceuticals Ltd) |
| 5267 | Prednisolone | 32808311000001101 | Prednisolone 5mg tablets (Genesis Pharmaceuticals Ltd) |
| 5267 | Prednisolone | 33425811000001105 | Prednisolone 1mg gastro-resistant tablets (A A H Pharmaceuticals Ltd) |
| 5267 | Prednisolone | 33428411000001103 | Prednisolone 1mg gastro-resistant tablets (Alliance Healthcare (Distribution) Ltd) |
| 5267 | Prednisolone | 33577711000001104 | Dilacort 2.5mg gastro-resistant tablets (Teva UK Ltd) |
| 5267 | Prednisolone | 33577911000001102 | Dilacort 5mg gastro-resistant tablets (Teva UK Ltd) |
| 5267 | Prednisolone | 34035411000001102 | Prednisolone 5mg soluble tablets (Accord Healthcare Ltd) |
| 5267 | Prednisolone | 34172711000001106 | Prednisolone 5mg soluble tablets (Phoenix Labs Ltd) |
| 5267 | Prednisolone | 34444511000001106 | Prednisolone 5mg gastro-resistant tablets (Bristol Laboratories Ltd) |
| 5267 | Prednisolone | 36568211000001107 | Prednisolone 2.5mg gastro-resistant tablets (Bristol Laboratories Ltd) |
| 5267 | Prednisolone | 37130011000001104 | Prednisolone 1mg gastro-resistant tablets (Mawdsley-Brooks & Company Ltd) |
| 5267 | Prednisolone | 37130211000001109 | Prednisolone 2.5mg gastro-resistant tablets (Mawdsley-Brooks & Company Ltd) |
| 5267 | Prednisolone | 37130611000001106 | Prednisolone 5mg gastro-resistant tablets (Mawdsley-Brooks & Company Ltd) |
| 5267 | Prednisolone | 37131011000001108 | Prednisolone 25mg tablets (Mawdsley-Brooks & Company Ltd) |
| 5267 | Prednisolone | 37727411000001103 | Prednisolone 5mg soluble tablets (Pilsco Ltd) |
| 5267 | Prednisolone | 37778311000001107 | Prednisolone 1mg gastro-resistant tablets (DE Pharmaceuticals) |
| 5267 | Prednisolone | 37778511000001101 | Prednisolone 10mg/ml oral solution sugar free (DE Pharmaceuticals) |
| 5267 | Prednisolone | 37778711000001106 | Prednisolone 25mg tablets (DE Pharmaceuticals) |
| 5267 | Prednisolone | 37941611000001100 | Prednisolone 10mg/ml oral solution sugar free (Alliance Healthcare (Distribution) Ltd) |
| 5267 | Prednisolone | 38721711000001107 | Prednisolone 5mg tablets (NorthStar Healthcare Unlimited Company) |
| 5267 | Prednisolone | 39169011000001106 | Prednisolone 1mg tablets (Medihealth (Northern) Ltd) |
| 5267 | Prednisolone | 39169811000001100 | Prednisolone 10mg tablets (Medihealth (Northern) Ltd) |
| 5267 | Prednisolone | 39172911000001107 | Prednisolone 2.5mg tablets (Medihealth (Northern) Ltd) |
| 5267 | Prednisolone | 39173511000001107 | Prednisolone 30mg tablets (Medihealth (Northern) Ltd) |
| 5267 | Prednisolone | 39174011000001102 | Prednisolone 10mg/ml oral solution sugar free (Medihealth (Northern) Ltd) |
| 5267 | Prednisolone | 39179711000001104 | Prednisolone 5mg soluble tablets (Medihealth (Northern) Ltd) |
| 5267 | Prednisolone | 39180111000001100 | Prednisolone 25mg tablets (Medihealth (Northern) Ltd) |
| 5267 | Prednisolone | 39204911000001106 | Prednisolone 5mg gastro-resistant tablets (Medihealth (Northern) Ltd) |
| 5267 | Prednisolone | 39300111000001105 | Prednisolone 2.5mg gastro-resistant tablets (Medihealth (Northern) Ltd) |
| 5267 | Prednisolone | 39722711000001103 | Prednisolone 5mg soluble tablets |
| 5267 | Prednisolone | 39888311000001108 | Prednisolone 20mg tablets (Medihealth (Northern) Ltd) |
| 5267 | Prednisolone | 40021511000001101 | Prednisolone 5mg tablets (Relonchem Ltd) |
| 5267 | Prednisolone | 40435311000001104 | Prednisolone 10mg/ml oral solution sugar free (Aerona Clinical Ltd) |

**Table S4: The RECORD statement – checklist of items, extended from the STROBE statement, for observational studies using routinely collected health data**

| | Item No. | STROBE items | Location in manuscript where items are reported | RECORD items | Location in manuscript where items are reported |
|---|---|---|---|---|---|
| **Title and abstract** | | | | | |
| | 1 | (a) Indicate the study's design with a commonly used term in the title or the abstract<br>(b) Provide in the abstract an informative and balanced summary of what was done and what was found | Page 1, 2<br><br>Page 2 | RECORD 1.1: The type of data used should be specified in the title or abstract. When possible, the name of the databases used should be included.<br><br>RECORD 1.2: If applicable, the geographic region and timeframe within which the study took place should be reported in the title or abstract.<br><br>RECORD 1.3: If linkage between databases was conducted for the study, this should be clearly stated in the title or abstract. | Page 1, 2<br><br>Page 1, 2<br><br>NA |
| **Introduction** | | | | | |
| Background rationale | 2 | Explain the scientific background and rationale for the investigation being reported | Page 4 | | |
| Objectives | 3 | State specific objectives, including any prespecified hypotheses | Page 4 | | |
| **Methods** | | | | | |
| Study Design | 4 | Present key elements of study design early in the paper | Page 4 | | |
| Setting | 5 | Describe the setting, locations, and relevant dates, including periods of recruitment, exposure, follow-up, and data collection | Page 4 | | |
| Participants | 6 | *(a) Cohort study* - Give the eligibility criteria, and the sources and methods of selection of participants. Describe methods of follow-up<br>*Case-control study* - Give the eligibility criteria, and the sources and methods of case ascertainment and control selection. Give the rationale for the choice of cases and controls<br>*Cross-sectional study* - Give the eligibility criteria, and the sources and methods of selection of participants<br><br>*(b) Cohort study* - For matched studies, give matching criteria and number of exposed and unexposed<br>*Case-control study* - For matched studies, give matching criteria and the number of controls per case | Page 5<br><br>Page 4, 5 | RECORD 6.1: The methods of study population selection (such as codes or algorithms used to identify subjects) should be listed in detail. If this is not possible, an explanation should be provided.<br><br>RECORD 6.2: Any validation studies of the codes or algorithms used to select the population should be referenced. If validation was conducted for this study and not published elsewhere, detailed methods and results should be provided.<br><br>RECORD 6.3: If the study involved linkage of databases, consider use of a flow diagram or other graphical display to demonstrate the data linkage process, including the number of individuals with linked data at each stage. | Page 5<br><br>Page 5<br><br>NA |
| Variables | 7 | Clearly define all outcomes, exposures, predictors, potential confounders, and effect modifiers. Give diagnostic criteria, if applicable. | Page 4 | RECORD 7.1: A complete list of codes and algorithms used to classify exposures, outcomes, confounders, and effect modifiers should be provided. If these cannot be reported, an explanation should be provided. | Supplement Tables S1-S3, Ref 30, page 6 |

| | | | | | |
|---|---|---|---|---|---|
| Data sources/ measurement | 8 | For each variable of interest, give sources of data and details of methods of assessment (measurement). Describe comparability of assessment methods if there is more than one group | Page 5 | | |
| Bias | 9 | Describe any efforts to address potential sources of bias | NA | | |
| Study size | 10 | Explain how the study size was arrived at | Page 4-6 | | |
| Quantitative variables | 11 | Explain how quantitative variables were handled in the analyses. If applicable, describe which groupings were chosen, and why | Page 6-7 | | |
| Statistical methods | 12 | (a) Describe all statistical methods, including those used to control for confounding<br>(b) Describe any methods used to examine subgroups and interactions<br>(c) Explain how missing data were addressed<br>(d) *Cohort study* - If applicable, explain how loss to follow-up was addressed<br>*Case-control study* - If applicable, explain how matching of cases and controls was addressed<br>*Cross-sectional study* - If applicable, describe analytical methods taking account of sampling strategy<br>(e) Describe any sensitivity analyses | Page 6-7 | | |
| Data access and cleaning methods | | .. | | RECORD 12.1: Authors should describe the extent to which the investigators had access to the database population used to create the study population.<br><br>RECORD 12.2: Authors should provide information on the data cleaning methods used in the study. | Page 12-13 Contributions<br><br>Page 6-7 |
| Linkage | | .. | | RECORD 12.3: State whether the study included person-level, institutional-level, or other data linkage across two or more databases. The methods of linkage and methods of linkage quality evaluation should be provided. | NA |
| **Results** | | | | | |
| Participants | 13 | (a) Report the numbers of individuals at each stage of the study (*e.g.*, numbers potentially eligible, examined for eligibility, confirmed eligible, included in the study, completing follow-up, and analysed)<br>(b) Give reasons for non-participation at each stage.<br>(c) Consider use of a flow diagram | Page 6 | RECORD 13.1: Describe in detail the selection of the persons included in the study (*i.e.,* study population selection) including filtering based on data quality, data availability and linkage. The selection of included persons can be described in the text and/or by means of the study flow diagram. | Page 8 |
| Descriptive data | 14 | (a) Give characteristics of study participants (*e.g.*, demographic, clinical, social) and information on exposures and potential confounders | Page 8-9 | | |

| | | | | | |
|---|---|---|---|---|---|
| | | (b) Indicate the number of participants with missing data for each variable of interest<br>(c) *Cohort study* - summarise follow-up time (*e.g.*, average and total amount) | | | |
| Outcome data | 15 | *Cohort study* - Report numbers of outcome events or summary measures over time<br>*Case-control study* - Report numbers in each exposure category, or summary measures of exposure<br>*Cross-sectional study* - Report numbers of outcome events or summary measures | Page 8-10 | | |
| Main results | 16 | (a) Give unadjusted estimates and, if applicable, confounder-adjusted estimates and their precision (e.g., 95% confidence interval). Make clear which confounders were adjusted for and why they were included<br>(b) Report category boundaries when continuous variables were categorized<br>(c) If relevant, consider translating estimates of relative risk into absolute risk for a meaningful time period | Page 8-9 | | |
| Other analyses | 17 | Report other analyses done—e.g., analyses of subgroups and interactions, and sensitivity analyses | NA | | |
| **Discussion** | | | | | |
| Key results | 18 | Summarise key results with reference to study objectives | Page 10 | | |
| Limitations | 19 | Discuss limitations of the study, taking into account sources of potential bias or imprecision. Discuss both direction and magnitude of any potential bias | Page 11, 12 | RECORD 19.1: Discuss the implications of using data that were not created or collected to answer the specific research question(s). Include discussion of misclassification bias, unmeasured confounding, missing data, and changing eligibility over time, as they pertain to the study being reported. | Page 11, 12 |
| Interpretation | 20 | Give a cautious overall interpretation of results considering objectives, limitations, multiplicity of analyses, results from similar studies, and other relevant evidence | Page 10-12 | | |
| Generalisabilit y | 21 | Discuss the generalisability (external validity) of the study results | Page 12 | | |
| **Other Information** | | | | | |
| Funding | 22 | Give the source of funding and the role of the funders for the present study and, if applicable, for the original study on which the present article is based | Page 2, 13 | | |
| Accessibility of protocol, raw data, and programming code | | .. | | RECORD 22.1: Authors should provide information on how to access any supplemental information such as the study protocol, raw data, or programming code. | Page 13 |

*Reference: Benchimol EI, Smeeth L, Guttmann A, Harron K, Moher D, Petersen I, Sørensen HT, von Elm E, Langan SM, the RECORD Working Committee. The REporting of studies Conducted using Observational Routinely-collected health Data (RECORD) Statement. *PLoS Medicine* 2015; in press.

*Checklist is protected under Creative Commons Attribution (CC BY) license.
